# Safety and Immunogenicity of an Accelerated Ebola Vaccination Schedule in People with and without Human Immunodeficiency Virus: A Randomized Clinical Trial

**DOI:** 10.3390/vaccines12050497

**Published:** 2024-05-04

**Authors:** Julie A. Ake, Kristopher Paolino, Jack N. Hutter, Susan Biggs Cicatelli, Leigh Anne Eller, Michael A. Eller, Margaret C. Costanzo, Dominic Paquin-Proulx, Merlin L. Robb, Chi L. Tran, Lalaine Anova, Linda L. Jagodzinski, Lucy A. Ward, Nicole Kilgore, Janice Rusnak, Callie Bounds, Christopher S. Badorrek, Jay W. Hooper, Steven A. Kwilas, Ine Ilsbroux, Dickson Nkafu Anumendem, Auguste Gaddah, Georgi Shukarev, Viki Bockstal, Kerstin Luhn, Macaya Douoguih, Cynthia Robinson

**Affiliations:** 1 U.S. Military HIV Research Program, Center for Infectious Disease Research, Walter Reed Army Institute of Research, Silver Spring, MD 20910, USA; 2 Clinical Trials Center, Center for Enabling Capabilities, Walter Reed Army Institute of Research, Silver Spring, MD 20910, USA; 3 ICON Government and Public Health Solutions, Silver Spring, MD 20901, USA; 4 Henry M. Jackson Foundation for the Advancement of Military Medicine, Bethesda, MD 20817, USA; 5 Diagnostics and Countermeasures Branch, Center for Infectious Diseases Research, Walter Reed Army Institute of Research, Silver Spring, MD 20910, USA; 6 Joint Project Manager for Chemical, Biological, Radiological, and Nuclear Medical, U.S. Department of Defense Joint Program Executive Office for Chemical, Biological, Radiological and Nuclear Defense, Fort Detrick, MD 21702, USA; 7 Virology Division, U.S. Army Medical Research Institute of Infectious Diseases (USAMRIID), Fort Detrick, MD 21702, USA; 8 Janssen Research & Development, 2340 Beerse, Belgium; 9 Janssen Vaccines & Prevention B.V., 2333 Leiden, The Netherlands

**Keywords:** Ebola virus, vaccine safety, immunogenicity

## Abstract

The safety and immunogenicity of the two-dose Ebola vaccine regimen MVA-BN-Filo, Ad26.ZEBOV, 14 days apart, was evaluated in people without HIV (PWOH) and living with HIV (PLWH). In this observer-blind, placebo-controlled, phase 2 trial, healthy adults were randomized (4:1) to receive MVA-BN-Filo (dose 1) and Ad26.ZEBOV (dose 2), or two doses of saline/placebo, administered intramuscularly 14 days apart. The primary endpoints were safety (adverse events (AEs)) and immunogenicity (Ebola virus (EBOV) glycoprotein-specific binding antibody responses). Among 75 participants (n = 50 PWOH; n = 25 PLWH), 37% were female, the mean age was 44 years, and 56% were Black/African American. AEs were generally mild/moderate, with no vaccine-related serious AEs. At 21 days post-dose 2, EBOV glycoprotein-specific binding antibody responder rates were 100% among PWOH and 95% among PLWH; geometric mean antibody concentrations were 6286 EU/mL (n = 36) and 2005 EU/mL (n = 19), respectively. A total of 45 neutralizing and other functional antibody responses were frequently observed. Ebola-specific CD4+ and CD8+ T-cell responses were polyfunctional and durable to at least 12 months post-dose 2. The regimen was well tolerated and generated robust, durable immune responses in PWOH and PLWH. Findings support continued evaluation of accelerated vaccine schedules for rapid deployment in populations at immediate risk. Trial registration: NCT02598388 (submitted 14 November 2015).

## 1. Introduction

The frequency and size of Ebola disease (ED) outbreaks have increased since its discovery in 1976, including two large outbreaks from 2014 to 2016 and 2018 to 2020 and, more recently, three outbreaks in 2021, two in 2022, and one spanning 2022–2023 [1,2,3,4]. A two-dose vaccine regimen with an adenovirus type 26 (Ad26) vaccine encoding the Zaire Ebola virus (EBOV) glycoprotein (GP; Ad26.ZEBOV, Zabdeno, Janssen Vaccines & Prevention B.V., Leiden, The Netherlands) and a multivalent modified vaccinia Ankara filovirus vaccine (MVA-BN-Filo, Mvabea, Bavarian Nordic, Hellerup, Denmark) has been safe and immunogenic in clinical trials, inducing robust and durable antibody and T-cell responses [5,6,7,8,9,10,11,12,13,14,15,16,17]. The 56-day Ad26.ZEBOV, MVA-BN-Filo regimen received marketing authorization under exceptional circumstances from the European Medicines Agency (EMA) [18,19], was recommended by the World Health Organization (WHO) Strategic Advisory Group of Experts on Immunization for prophylactic use in people with lower risk of Ebola infection during the 2018–2020 outbreak [20] and was granted WHO prequalification in April 2021 [21]. While this Ad26.ZEBOV, MVA-BN-Filo regimen has been well studied [5,6,7,8,9,10,11,12,13,14,15,16,17], regimens reversing the product order are less robustly characterized in terms of numbers of participants and types of populations studied.

Compressed ED vaccine schedules are similarly less well studied. Safe accelerated vaccination schedules rapidly inducing peak immune responses could be advantageous for immunizing outbreak responders, similar to accelerated pretravel vaccination schedules for the prevention of hepatitis A and B, rabies, and tick-borne encephalitis [22]. In a phase 1 study, the MVA-BN-Filo, Ad26.ZEBOV vaccine regimen induced higher and more frequent cellular immune responses and lower but similarly frequent humoral responses when administered over a 14-day period compared to a 56-day period in healthy adults [10].

As ED outbreaks primarily occur in regions of high HIV prevalence, and antibody responses to vaccines for other pathogens (e.g., hepatitis B) are lower in people living with HIV (PLWH), a thorough evaluation of ED vaccines in PLWH is relevant to the product’s intended use [23]. We, therefore, included a cohort of PLWH in a clinical trial to assess the safety and immunogenicity of the 14-day MVA-BN-Filo, Ad26.ZEBOV regimen in this population. Safety data from this pilot study (Part 1) informed progression to a larger, subsequent evaluation of accelerated Ebola vaccine regimens (Part 2).

## 2. Methods

### 2.1. Study Design

Part 1 of EBL2003/RV456 (NCT02598388) was conducted at a single center in the U.S., specifically the Walter Reed Army Institute of Research Clinical Trials Center, and Part 2 of the trial was conducted at six centers in sub-Saharan Africa; Part 1 results are presented here, with Part 2 results presented separately. Part 1 included people without HIV (PWOH) and PLWH, evaluating MVA-BN-Filo with Ad26.ZEBOV 14 days later. Randomization was conducted with a 4:1 ratio of vaccine to placebo, and study groups were enrolled in parallel. The study was conducted in accordance with the Helsinki Declaration and was approved by the Walter Reed Army Institute of Research Institutional Review Board. All participants provided written informed consent. The study protocol and statistical analysis plan are available in Supplements S1 and S2.

### 2.2. Participants

Participants were healthy and aged 18–70 years at randomization. PLWH were required to have chronic infection treated with stable antiretroviral therapy and CD4+ T-cell count > 200 cells/μL. Exclusion criteria included breastfeeding or pregnancy, prior ED, vaccination with a candidate Ebola vaccine, vaccination with a live-attenuated vaccine in the previous 30 days, inactivated vaccine receipt in the previous 14 days, and previous severe adverse reactions to vaccination.

### 2.3. Randomization and Masking

Participants were centrally randomized using computer-generated randomization with a block size of five. Study personnel (except those responsible for vaccine preparation) and participants were blinded to study vaccine allocation until all participants had completed at least the Day 380 visit (or discontinued earlier) and the database locked. Dispensing syringes were covered with masking tape.

### 2.4. Objectives

The primary objectives were to assess the safety and immunogenicity of the MVA-BN-Filo, Ad26.ZEBOV regimen, as expressed by the number of participants with adverse events (AEs), and anti-EBOV GP antibody responses as measured by Filovirus Animal Non-Clinical Group (FANG) EBOV GP enzyme-linked immunosorbent assay (ELISA) at Day 36. A secondary objective compared the safety and tolerability of the regimen in PWOH and PLWH. Exploratory objectives included assessment of binding antibody responses at other time points, EBOV GP-specific CD4+ and CD8+ T-cell responses measured by intracellular cytokine staining (ICS), cross-strain neutralizing antibody responses, Fc-mediated EBOV GP-specific antibody effector functions, and changes in viral load among PLWH.

### 2.5. Vaccines and Vaccinations

Ad26.ZEBOV is a recombinant, replication-incompetent, Ad26-vectored vaccine that encodes EBOV Mayinga GP [5]. MVA-BN-Filois a recombinant, nonreplicating, modified vaccinia Ankara-vectored vaccine that encodes the EBOV Mayinga GP, Sudan virus Gulu GP, Marburg virus Musoke GP, and the Taï Forest virus nucleoprotein [5]. Consistent with the approved dose levels and volumes for the 56-day regimen [18,19], all doses were administered via a single 0.5 mL intramuscular deltoid injection. Participants randomized to active vaccine received MVA-BN-Filo (1 × 10^8^ infectious units) on Day 1, followed by Ad26.ZEBOV (5 × 10^10^ viral particles) on Day 15; placebo recipients received 0.9% saline at these time points.

### 2.6. Safety Evaluations

Participants were observed for ≥30 min post-vaccination. Local and systemic solicited AEs were recorded for seven days following each vaccination. Safety blood tests were performed seven days after each vaccination. Unsolicited AEs and serious AEs (SAEs) were recorded from informed consent until Day 57 and study end, respectively. AEs were graded as 1—mild, 2—moderate, or 3—severe, according to the adapted Division of Microbiology and Infectious Diseases Toxicity Tables [24]. Toxicity scales for clinical laboratory assessments were based on the U.S. FDA toxicity grading scale for healthy adults/adolescents enrolled in preventive vaccine trials [25]. For PLWH, viral loads and CD4+ T-cell counts were measured at prespecified time points (Supplementary methods, Supplement S3).

### 2.7. Immunogenicity Assessments

Peripheral blood mononuclear cells (PBMCs) and serum specimens were cryopreserved from time points prior to each vaccination on Days 1, 15, 36 (21 days post-dose 2), 57 (42 days post-dose 2), 195 (6 months post-dose 2), and 380 (12 months post-dose 2). Total immunoglobulin G (IgG) EBOV GP (Kikwit)-specific binding antibody concentrations were assessed using the EBOV GP (Kikwit) FANG anti-EBOV GP IgG ELISA at Q^2^ Solutions Laboratories (San Juan Capistrano, California, USA) [11,26]. EBOV GP- specific neutralizing antibody titers specific for Zaire strains Kikwit and Makona, as well as for the Bundibugyo EBOV, were measured by pseudovirion neutralization assay (Supplementary methods, Supplement S3) at the U.S. Army Research Institute of Infectious Diseases (Fort Detrick, Maryland, USA). PBMCs were stimulated with peptide pools covering the GP from the EBOV Mayinga strain and analyzed to establish the percentage of CD4+ and CD8+ T-cells producing interferon γ (IFN-γ) and/or interleukin-2 (IL-2; qualified markers) by ICS (Supplementary methods, Supplement S3) [27].

Additionally, IL-4, IL-21, tumor necrosis factor-α (TNF-α), and CD154 were measured by ICS at the same time points and were used for combinatorial polyfunctionality analysis of antigen-specific T-cell subsets using the computational COMPASS package in R (version 4.2.3) (Supplementary methods, Supplement S3) [28]. EBOV GP-specific antibody-dependent cellular phagocytosis (ADCP), antibody-dependent natural killer cell activation (ADNKA), as well as antibody-dependent complement deposition (ADCD) were measured (Supplementary methods, Supplement S3) [29]. EBOV GP-specific antibody polyfunctionality was defined by positivity for all three Fc-mediated effector functions and neutralization.

### 2.8. Statistical Analysis and Sample Size

The sample of 75 participants, which included 60 who received active vaccine, was not based on formal statistical hypothesis testing. Nevertheless, if a specific AE was not observed, the one-sided 97.5% upper confidence limit of the true rate of this AE was <16.8% and <8.8% for a sample size of 20 (PLWH) and 40 (PWOH) active vaccine recipients, respectively. Additionally, this sample size permitted a thorough immunologic assessment of the accelerated regimen. Data were analyzed when all participants completed the study or discontinued prior to its end. Safety analyses for unsolicited events were performed on the full analysis set (those receiving ≥ 1 study vaccine dose). Analyses of solicited adverse events were based on participants in a full analysis set with recorded reactogenicity data in the database. The primary immunogenicity analysis set included all vaccinated participants who received both doses within the protocol-specified window and who had ≥1 evaluable post-vaccination immunogenicity sample. Data were analyzed descriptively without formal hypothesis testing. Spearman’s correlation coefficients for binding antibody concentrations and neutralizing antibody titers were calculated on Day 36. Spearman’s correlation coefficients for binding antibody concentrations and HIV viral load or CD4+ T-cell count were calculated on Days 15 and 36. PRISM version 9.4.1 (GraphPad Software, Boston, MA, USA) was used for ICS graphs. All other statistical analyses employed SAS version 9.2 (SAS Institute, Cary, NC, USA).

## 3. Results

**Figure 1:**
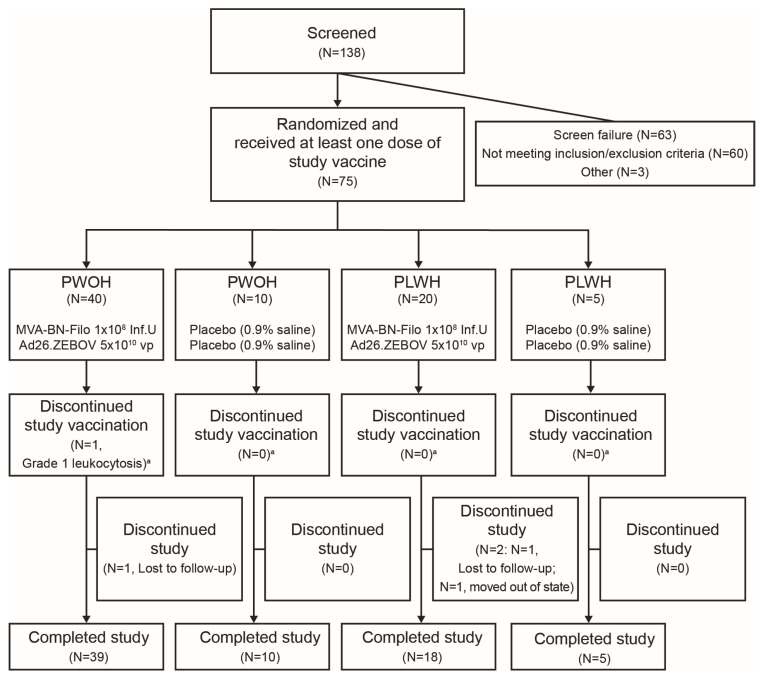
**CONSORT diagram.** Inf.U, infectious units; N, number of participants; PLWH, people living with HIV; PWOH, people without HIV; vp, viral particles. ^a^ Participants who discontinued vaccination could still complete the study.

The study was performed from 14 December 2015 to 14 December 2017. Of the 138 individuals screened, 63 were not eligible (60 did not meet inclusion/exclusion criteria and 3 were due to other reasons), and 75 participants were randomized and received ≥1 vaccine dose; 40 PWOH and 20 PLWH received the active vaccine, and 10 PWOH and 5 PLWH received placebo (Figure 1). The mean age was 44 years, and 37% were female. Most participants were Black/African American (56%) or White (41%; Table 1). Overall, 72/75 (96%) participants completed the study; three discontinued in the active vaccine group (one PWOH and one PLWH were lost to follow-up; one PLWH moved out of state; Figure 1).

**Figure 2:**
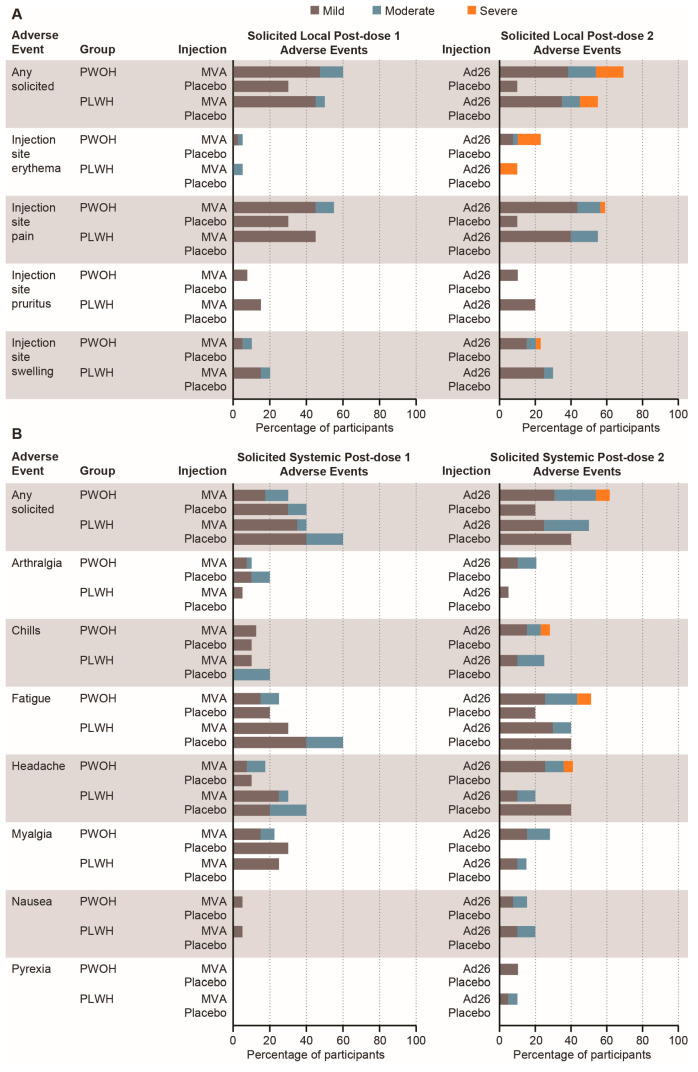
**Solicited adverse events.** (**A**) Solicited local adverse events; (**B**) Solicited systemic adverse events. Percentages reflect n/N, where n is the number of participants with one or more adverse events and N is the number of participants with available reactogenicity data after the given dose. Ad26, Ad26.ZEBOV; MVA, MVA-BN-Filo; PLWH, people living with HIV; PWOH, people without HIV.

Solicited AEs were predominantly mild-to-moderate (Figure 2; Supplementary Tables S1 and S2, Supplement S3). The most frequently reported local AE across groups was injection-site pain. Eight participants reported a total of nine grade 3 solicited local AEs, seven of which were erythema, all occurring after Ad26.ZEBOV vaccination. Headache, myalgia, and fatigue were the most frequently reported solicited systemic AEs after any vaccination/placebo. No grade 3 solicited systemic AEs were observed following MVA-BN-Filo or placebo, and no grade 3 fevers were reported following any vaccination/placebo. Three participants (all PWOH) reported grade 3 solicited systemic AEs following Ad26.ZEBOV receipt. No remarkable trends were noted in unsolicited AE reporting. One grade 3 unsolicited anemia AE following MVA-BN-Filo administration occurred in a participant without HIV (Supplementary Table S3, Supplement S3) and was considered unrelated to the study vaccine.

Five SAEs were reported by four participants, all of which occurred >28 days post-vaccination (Supplementary Table S4, Supplement S3) and were considered unrelated to the study vaccine. No deaths were reported. One participant did not receive dose 2 due to grade 1 leukocytosis. The event occurred in a participant without HIV post-vaccination with MVA-BN-Filo and was considered nonserious and possibly related to the study vaccine by the investigator.

All PLWH had HIV viral loads < 200 copies/mL at screening and at the final visit, with a single transient post-vaccination blip (Supplementary Table S5, Supplement S3).

Fifty-seven participants who received the MVA-BN-Filo, Ad26.ZEBOV active regimen (38 PWOH; 19 PLWH) and 15 placebo recipients fulfilled the criteria for inclusion in the per-protocol immunogenicity analyses set.

**Figure 3:**
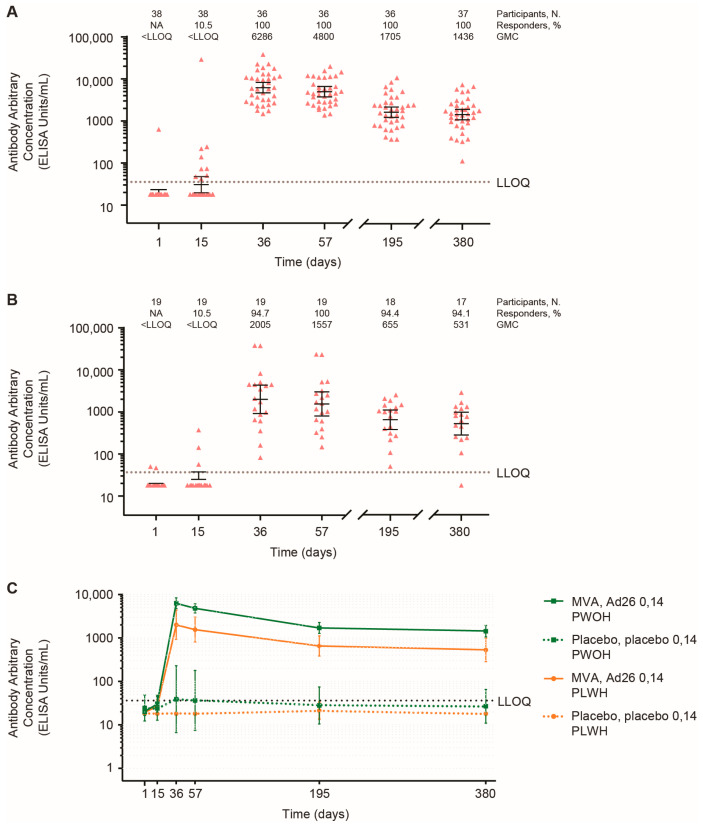
**FANG anti-EBOV GP IgG ELISA measurements of EBOV GP-specific binding antibody responses.** (**A**) In PWOH; (**B**) In PLWH; (**C**) All PWOH and PLWH active vaccine regimen and placebo recipients. The points (symbols) represent individual GMCs, and error bars denote 95% CIs. Ad26, Ad26.ZEBOV; CI, confidence interval; EBOV, Ebola virus; ELISA, enzyme-linked immunosorbent assay; FANG, Filovirus Animal Non-Clinical Group; GMC, geometric centration; GP glycoprotein; IgG, immunoglobulin G; LLOQ, lower limit of quantification; MVA, MVA-BN-Filo; N, number of participants; NA, not applicable; PLWH, people living with HIV; PWOH, people without HIV.

On Day 15, 4/38 (11%) and 2/19 (11%) PWOH and PLWH, respectively, displayed an EBOV GP-specific binding antibody response following the first active vaccination (Figure 3A–C; Supplementary Table S6, Supplement 3). On Day 36, responder rates increased to 36/36 (100%) and 18/19 (95%), with geometric mean concentrations (GMCs) of 6286 ELISA units (EU)/mL (95% confidence interval (CI), 4730–8355) and 2005 EU/mL (95% CI, 923–4353) for PWOH and PLWH, respectively. Binding antibody response magnitude (GMC) for both populations decreased by Days 195 and 380. However, responder rates remained high at 100% in PWOH and 94% in PLWH on Day 380. In nearly all placebo recipients, binding antibody responses were low or not quantifiable over the course of the study. The negligible impact of HIV parameters on EBOV GP-specific binding antibody responses is illustrated in Supplementary Figure S1, Supplement S3.

**Figure 4:**
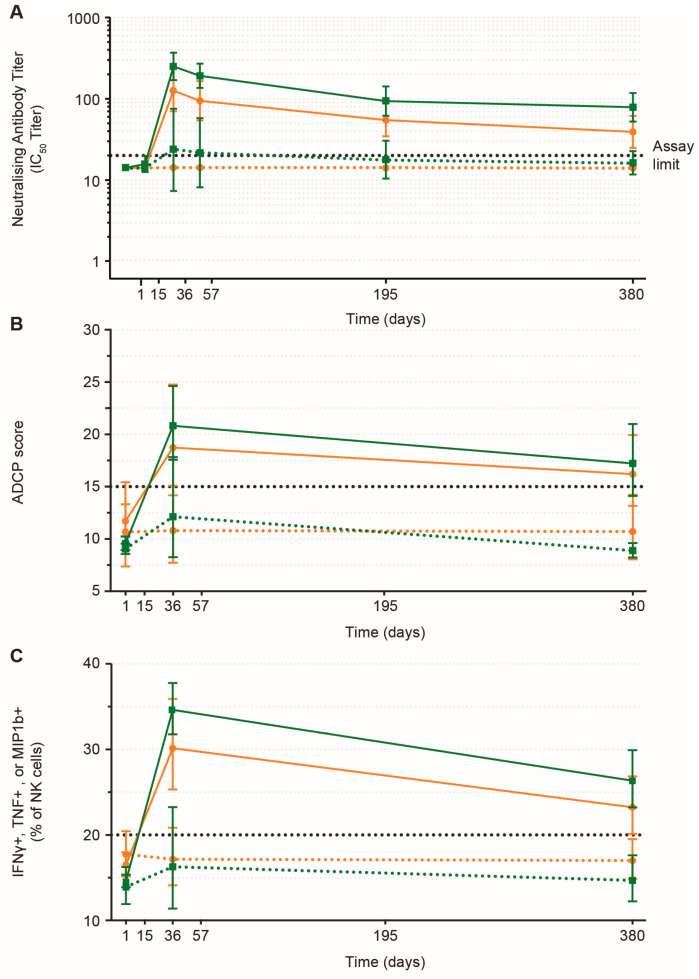

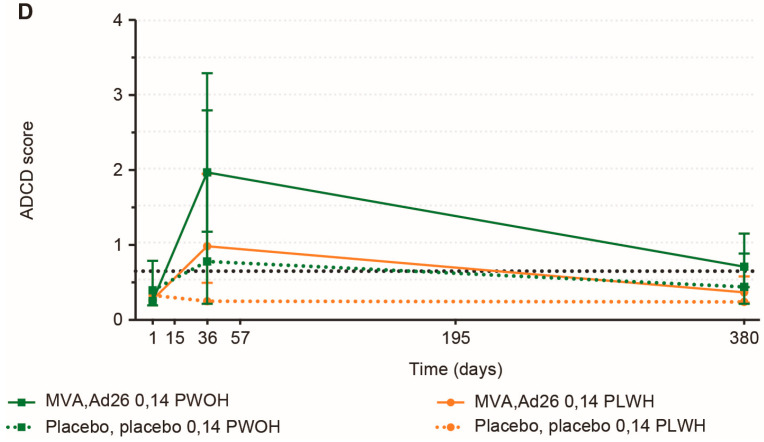
**EBOV GP-specific functional antibody responses.** (**A**) Neutralizing antibody responses to EBOV GP Zaire 95 Kikwit (psVNA); (**B**) Antibody-dependent cellular phagocytosis (ADCP); (**C**) Antibody-dependent natural killer cell activation (ADNKA); (**D**) Antibody-dependent complement deposition (ADCD). Specimens from PWOH or PLWH who were vaccinated with MVA, Ad26, or placebo were evaluated by (**A**) psVNA to measure the cross-neutralizing antibody response against EBOV Zaire 95 Kikwit strain. Blood was collected on Days 0, 15, 36, 57, 195, and 360 and ran in the assays. (**B**) Antibody-dependent cellular phagocytosis, (**C**) NK cell activation as measured by ICS, and (**D**) complement deposition were evaluated on Days 0, 36, and 360. For (**A**), data are plotted as geometric mean IC_50_ titers ± 95% CI, and the lower assay limit was 20 (dotted line). For (**B**–**D**), data are plotted as geometric mean ± 95% CI, and the threshold for positivity was determined using plasma from controls (dotted line). Ad26, Ad26.ZEBOV; CI, confidence interval; EBOV, Ebola virus; GP glycoprotein; IC50, 50% inhibitory concentration; ICS, intracellular cytokine staining; IFN-γ, interferon γ; MVA, MVA-BN-Filo; NK, natural killer; PLWH, people living with HIV; psVNA, pseudovirion neutralization assay; PWOH, people without HIV; TNF, tumor necrosis factor.

On Day 15, neutralizing antibody responses against the Kikwit strain were observed in 1/40 (3%) PWOH and 0/20 (0%) PLWH (Figure 4A). On Day 36, Kikwit-specific neutralizing antibody responses were observed in 37/40 (93%; geometric mean titer (GMT): 251 50% inhibitory concentration (IC_50_) titer) PWOH and 16/20 (80%; GMT: 126 IC_50_ titer) PLWH. A strong positive correlation between EBOV GP binding antibody and neutralizing antibody responses was observed for both study populations (pooled PWOH and PLWH Day 36 Spearman correlation factor: 0.728; Supplementary Figure S2, Supplement S3). Ninety-eight percent (39/40) of PWOH and eighty-five percent (17/20) of PLWH developed cross-strain neutralizing responses to the Makona strain, while thirty-five percent (14/40) of PWOH and ten percent (2/20) of PLWH developed cross-neutralizing responses to Bundibugyo (Supplementary Figures S3 and S4, Supplement S3). Neutralization declined after Day 36 but remained detectable to Day 380, except for the Bundibugyo strain among PLWH. Correlation plots of cross-strain neutralization and binding antibody responses are shown in Supplementary Figure S5, Supplement S3.

EBOV GP-specific ADCP responses were highest in vaccine recipients on Day 36, with 27/40 (68%) responders in PWOH and 12/20 (60%) responders among PLWH (Figure 4B; Supplementary Figure S6, Supplement S3). Response magnitude declined by Day 380 but remained quantifiable in both groups at the end of the study, at which point 23/39 (59%) PWOH and 12/18 (67%) PLWH had detectable EBOV GP-specific ADCP responses. On Day 36, 39/40 (98%) PWOH and 15/20 (75%) PLWH displayed an ADNKA response (Figure 4C; Supplementary Figure S7, Supplement S3). ADNKA responses persisted in 29/39 (74%) and 13/19 (68%) PWOH and PLWH, respectively, to Day 380. ADCD response rates were greater in PWOH on Day 36, manifesting in 63% (25/40) of PWOH and 47% (9/19) of PLWH (Figure 4D). ADCD responses, in contrast to the other effector responses, were less durable, with 14/39 (36%) PWOH and 4/18 (22%) PLWH demonstrating responses on Day 380 (Supplementary Figure S8, Supplement S3). At the Day 36 immune response peak, polyfunctional EBOV GP-specific antibody responses were observed in 60% (24/40) of PWOH and 32% (6/19) of PLWH (Supplementary Figure S9, Supplement S3). By Day 380, antibody polyfunctionality had declined to 31% (12/39) of PWOH and 11% (2/18) of PLWH. In almost all placebo recipients, EBOV GP-specific functional antibody responses were low or not quantifiable.

**Figure 5:**
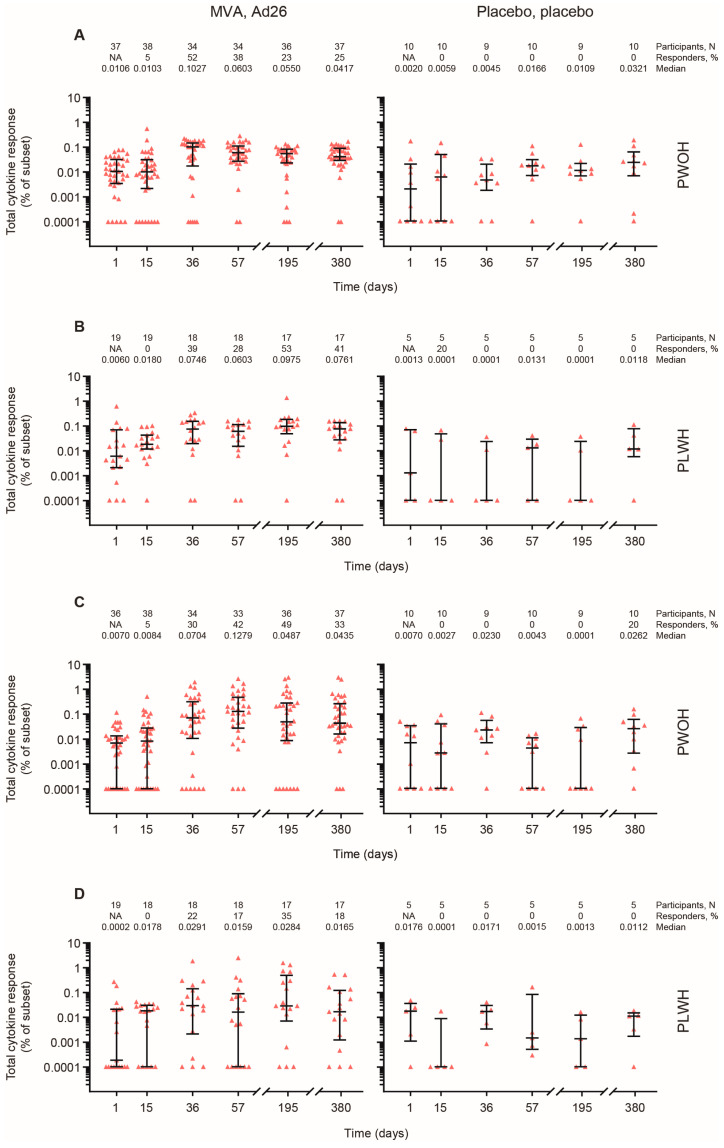
**T-cell–mediated analysis of EBOV-specific vaccine response.** (**A**) CD4+ T-cell responses in PWOH; (**B**) CD4+ T-cell responses in PLWH; (**C**) CD8+ T-cell responses in PWOH; (**D**) CD8+ T-cell responses in PLWH. EBOV GP-specific T-cell responses were measured by intracellular cytokine staining. Total cytokine response, identified by qualified cytokines, was assessed on Days 1, 15, 36, 57, 195, and 380. Participants (N), responders (%), and median responses are listed in rows above each corresponding graph. Data are plotted as individual points with bars indicating median and interquartile range. Ad26, Ad26.ZEBOV; EBOV, Ebola virus; GP, glycoprotein; IFN-γ, interferon γ; IL-2, interleukin-2; MVA, MVA-BN-Filo; NA, not applicable; PLWH, people living with HIV; PWOH, people without HIV.

In vaccine recipients at Day 36, EBOV GP-specific CD4+ T-cells expressing IFN and/or IL-2 were detected in 17/33 (52%) PWOH (median value: 0.1027%) and in 7/18 (39%) PLWH (median: 0.0746%; Figure 5A,B). By Day 380, the response among PWOH had declined to 9/36 responders (25%, median: 0.0417%), whereas for PLWH, there were 7/17 responders (41%, median: 0.0761%).

EBOV GP-specific CD8+ T-cells expressing IFN-γ and/or IL-2 were detected in 10/33 (30%) PWOH (median: 0.0704%) and 4/18 (22%) PLWH (median: 0.0291%) on Day 36 in the active vaccine group (Figure 5C,D). On Day 380, EBOV GP-specific CD8+ T-cells expressing IFN-γ and/or IL-2 were detected in 12/36 (33%) PWOH (median: 0.0435%) and 3/17 (18%) PLWH (median: 0.0165%).

COMPASS analysis revealed that most Ebola responders developed polyfunctional Ebola-specific CD4+ T-cells that produced IFN-γ, CD154, and TNF-α with or without IL-2. CD8+ T-cell responses included mainly IFN-γ and TNF-α (Supplementary Figure S10, Supplement S3).

## 4. Discussion

This study illustrates an accelerated two-dose heterologous Ebola vaccination schedule comprised of MVA-BN-Filo followed by Ad26.ZEBOV 14 days later is well tolerated and can elicit durable humoral and cellular responses against the EBOV GP in both PWOH and PLWH. Solicited AEs were generally mild-to-moderate with limited duration in both populations. Among PLWH, vaccination had no appreciable clinically significant impact on HIV viral suppression.

In the absence of efficacy data, immunobridging is used to infer a vaccine’s protective effect by comparing immunogenicity in humans to the relationship between immunogenicity and survival after challenge in nonhuman primates (NHP). Although a mechanistic correlate of protection for this vaccine has not yet been identified, binding antibodies against the EBOV surface GP strongly correlate with survival post-challenge in a fully lethal EBOV Kikwit NHP challenge model [30,31]. The Ad26.ZEBOV, MVA-BN-Filo 56-day regimen provided nearly 100% protection against infection in this model. The protective efficacy of this vaccine regimen was demonstrated via immunobridging, facilitating marketing authorization approval under exceptional circumstances by the EMA [18,19].

Compared to the 56-day regimen of the Ad26.ZEBOV, MVA-BN-Filo vaccines, the accelerated reverse regimen in this study elicited lower binding antibody concentrations 21 days post-dose 2; however, response rates were high, and antibody concentrations at 6 and 12 months post-dose 2 were similar [5,7,8,9,11,12,13]. As the immunobridging model is only informative for the 56-day regimen, the degree of protection against infection induced by this trial’s accelerated schedule cannot be extrapolated. Also, the protective efficacy of these vaccine regimens may be underestimated in this highly stringent animal model [30].

A cross-strain evaluation of neutralizing antibody responses demonstrated robust and durable titers to the Ebola Zaire Kikwit and Makona strains with strong correlations to the binding antibody responses measured in the FANG anti-EBOV GP IgG ELISA employing the Kikwit EBOV GP. Cross-strain neutralizing antibody responses against the Bundibugyo virus were detectable in some participants but at low titers. Animal model results suggest that antibody-dependent cell-mediated cytotoxicity and ADCP could confer protection against EBOV [32,33,34]. Therefore, effector antibody functions such as ADCP, ADNKA, and ADCD were evaluated, and these responses were generally durable and polyfunctional. An HIV vaccine regimen based on the same Ad26 platform has also been shown to induce polyfunctional antibody responses in both humans and NHP [35], and ADCP activity has been linked to protection against infection in a relevant simian-human immunodeficiency virus challenge model [36]. Cellular responses have also been linked to protection in NHP models [37], and in this study, we observed both CD4+ and CD8+ T-cell responses.

This trial’s data are aligned with previous observations that vaccines can stimulate functional immune responses in well-controlled PLWH, even if the magnitude of response is decreased [38]. Protection with this accelerated schedule is not yet established; however, recent data characterizing immune responses elicited by a Day 360 boost with either Ad26.ZEBOV or MVA-BN-Filo have shown a significant anamnestic response across heterologous regimens irrespective of the vaccination order or dosing interval of the primary regimen [10]. Indeed, we observe that this accelerated vaccine regimen induced a polyfunctional antibody, as well as T-cell response, in both PWOH and PLWH, often persisting to 12 months. Taken together, these data suggest that a heterologous two-dose vaccination regimen with an accelerated MVA-BN-Filo, Ad26.ZEBOV schedule may establish immune memory that can be rapidly recalled by subsequent boosting or even pathogen exposure.

Study limitations include the relatively small sample size, which limited statistical power to compare HIV status groups. Additionally, women were slightly under-represented in the enrollment (37%). Study results would have greater utility if the approved 56-day regimen was included for direct comparison. Finally, the specimen collection schedule was not optimized for defining early innate immune responses.

## 5. Conclusions

In summary, this is the first study to demonstrate safety and EBOV GP-specific immunogenicity in PLWH with an Ebola vaccination regimen of MVA-BN-Filo followed by Ad26.ZEBOV 14 days later. These results show that an accelerated vaccination schedule is well tolerated in both PWOH and PLWH and induces a broad array of durable humoral and cellular immune responses. These findings support continued consideration for accelerated regimen development for rapid deployment among outbreak responders and relevant global populations at immediate risk.

## Figures and Tables

**Table 1:** Participants’ demographic and baseline characteristics; full analysis set.

PWOH			PLWH		All Participants
	MVA, Ad26	Placebo, Placebo	MVA, Ad26	Placebo, Placebo	
Analysis set: Full analysis set, N	40	10	20	5	75
Age (years) at screening					
Mean (SD)	42.0 (14.4)	47.3 (11.8)	46.7 (12.9)	46.0 (8.6)	44.2 (13.4)
Body mass index (kg/m^2^)					
Mean (SD)	26.0 (4.9)	29.4 (7.7)	26.3 (4.8)	27.7 (4.4)	26.7 (5.3)
95% CI	(24.5–27.6)	(23.9–34.9)	(24.0–28.5)	(22.2–33.2)	(25.4–27.9)
Age group					
18–50 years, n (%)	25 (63)	6 (60)	12 (60)	3 (60)	46 (61)
51–70 years, n (%)	15 (38)	4 (40)	8 (40)	2 (40)	29 (39)
Sex					
Female, n (%)	18 (45)	7 (70)	2 (10)	1 (20)	28 (37)
Male, n (%)	22 (55)	3 (30)	18 (90)	4 (80)	47 (63)
Race					
American Indian or Alaska Native, n (%)	0	0	2 (10)	0	2 (3)
Black/African American, n (%)	19 (48)	6 (60)	13 (65)	4 (80)	42 (56)
White, n (%)	21 (53)	4 (40)	5 (25)	1 (20)	31 (41)

Ad26, Ad26.ZEBOV; CI, confidence interval; MVA, MVA-BN-Filo; N, number of participants; PLWH, people living with HIV; PWOH, people without HIV; SD, standard deviation.

## Data Availability

The data sharing policy of Janssen Pharmaceutical Companies of Johnson & Johnson is available at https://www.janssen.com/clinical-trials/transparency. As noted on this site, requests for access to the study data can be submitted through the Yale Open Data Access (YODA) Project site at http://yoda.yale.edu.

## Supplementary Materials

The following supporting information can be downloaded at https://www.mdpi.com/article/10.3390/vaccines12050497/s1, Supplement S1: Study protocol, Supplement S2: Statistical analysis plan, Supplement S3: Supplementary material (Supplementary methods, Supplementary results, Supplementary Table S1: Solicited local AEs by worst severity grade [full analysis set], Supplementary Table S2: Solicited systemic AEs by worst severity grade [full analysis set], Supplementary Table S3: Unsolicited AEs [full analysis set], Supplementary Table S4: SAEs [full analysis set], Supplementary Table S5: HIV viral load: categorization of HIV viral loads in PLWH, Supplementary Table S6: EBOV GP-specific binding antibody responses in each study group, from baseline to study completion as measured by FANG anti-EBOV GP IgG ELISA, Supplementary Figure S1: Correlation between HIV viral load at baseline [panel A] and CD4+ T-cells at baseline [panel B] and EBOV GP binding antibody in active vaccine recipients at Day 36, Supplementary Figure S2: Correlation between EBOV GP binding antibody [Kikwit] and neutralizing antibody responses at Day 36 in PWOH and PLWH [active vaccine recipients], Supplementary Figure S3: Cross-strain EBOV GP-specific neutralizing antibody responses as measured by psVNA, Supplementary Figure S4: Cross-strain EBOV GP neutralizing antibody responses as measured by psVNA, Supplementary Figure S5: Correlation between cross-strain neutralizing antibody responses and EBOV GP-specific binding antibody responses at Day 36 in active vaccine recipients, Supplementary Figure S6: EBOV GP-specific ADCP responses, Supplementary Figure S7: EBOV GP-specific antibody-dependent natural killer cell activation [ADNKA] responses, Supplementary Figure S8: EBOV GP-specific ADCD responses, Supplementary Figure S9: Antibody effector polyfunctionality, Supplementary Figure S10: Combinatorial polyfunctionality analysis of antigen-specific T-cell subsets [COMPASS], Members of the VAC52150/EBL2003/RV456 study team [in addition to authors]).

## References

[1] Malvy D., McElroy A.K., de Clerck H., Günther S., van Griensven J. (2019). Ebola virus disease. Lancet.

[2] Centers for Disease Control and Prevention 2014–2016 Ebola Outbreak in West Africa. https://www.cdc.gov/vhf/ebola/history/2014-2016-outbreak/index.html.

[3] World Health Oranization Ebola Outbreak—Democratic Republic of the Congo. North Kivu, Ituri 2018–2020. https://www.who.int/emergencies/situations/Ebola-2019-drc-.

[4] Centers for Disease Control and Prevention History of Ebola Disease Outbreaks. https://www.cdc.gov/vhf/ebola/history/chronology.html.

[5] Milligan I.D., Gibani M.M., Sewell R., Clutterbuck E.A., Campbell D., Plested E., Nuthall E., Voysey M., Silva-Reyes L., McElrath M.J. (2016). Safety and immunogenicity of novel adenovirus type 26- and modified vaccinia Ankara-vectored Ebola vaccines: A randomized clinical trial. JAMA.

[6] Shukarev G., Callendret B., Luhn K., Douoguih M., EBOVAC1 consortium (2017). A two-dose heterologous prime-boost vaccine regimen eliciting sustained immune responses to Ebola Zaire could support a preventive strategy for future outbreaks. Hum. Vaccin. Immunother..

[7] Winslow R.L., Milligan I.D., Voysey M., Luhn K., Shukarev G., Douoguih M., Snape M.D. (2017). Immune responses to novel adenovirus type 26 and modified vaccinia virus Ankara-vectored Ebola vaccines at 1 year. JAMA.

[8] Anywaine Z., Whitworth H., Kaleebu P., Praygod G., Shukarev G., Manno D., Kapiga S., Grosskurth H., Kalluvya S., Bockstal V. (2019). Safety and immunogenicity of a 2-dose heterologous vaccination regimen with Ad26.ZEBOV and MVA-BN-Filo ebola vaccines: 12-month data from a phase 1 randomized clinical trial in Uganda and Tanzania. J. Infect. Dis..

[9] Mutua G., Anzala O., Luhn K., Robinson C., Bockstal V., Anumendem D., Douoguih M. (2019). Safety and immunogenicity of a 2-dose heterologous vaccine regimen with Ad26.ZEBOV and MVA-BN-Filo Ebola vaccines: 12-month data from a phase 1 randomized clinical trial in Nairobi, Kenya. J. Infect. Dis..

[10] Goldstein N., Bockstal V., Bart S., Luhn K., Robinson C., Gaddah A., Callendret B., Douoguih M. (2022). Safety and immunogenicity of heterologous and homologous 2-dose regimens of adenovirus serotype 26- and modified vaccinia Ankara-vectored Ebola vaccines: A randomized, controlled phase 1 study. J. Infect. Dis..

[11] Pollard A.J., Launay O., Lelievre J.D., Lacabaratz C., Grande S., Goldstein N., Robinson C., Gaddah A., Bockstal V., Wiedemann A. (2021). Safety and immunogenicity of a two-dose heterologous Ad26.ZEBOV and MVA-BN-Filo Ebola vaccine regimen in adults in Europe (EBOVAC2): A randomised, observer-blind, participant-blind, placebo-controlled, phase 2 trial. Lancet Infect. Dis..

[12] Ishola D., Manno D., Afolabi M.O., Keshinro B., Bockstal V., Rogers B., Owusu-Kyei K., Serry-Bangura A., Swaray I., Lowe B. (2022). Safety and long-term immunogenicity of the two-dose heterologous Ad26.ZEBOV and MVA-BN-Filo Ebola vaccine regimen in adults in Sierra Leone: A combined open-label, non-randomised stage 1, and a randomised, double-blind, controlled stage 2 trial. Lancet Infect. Dis..

[13] Afolabi M.O., Ishola D., Manno D., Keshinro B., Bockstal V., Rogers B., Owusu-Kyei K., Serry-Bangura A., Swaray I., Lowe B. (2022). Safety and immunogenicity of the two-dose heterologous Ad26.ZEBOV and MVA-BN-Filo Ebola vaccine regimen in children in Sierra Leone: A randomised, double-blind, controlled trial. Lancet Infect. Dis..

[14] Barry H., Mutua G., Kibuuka H., Anywaine Z., Sirima S.B., Meda N., Anzala O., Eholie S., Bétard C., Richert L. (2021). Safety and immunogenicity of 2-dose heterologous Ad26.ZEBOV, MVA-BN-Filo Ebola vaccination in healthy and HIV-infected adults: A randomised, placebo-controlled phase II clinical trial in Africa. PLoS Med..

[15] Anywaine Z., Barry H., Anzala O., Mutua G., Sirima S.B., Eholie S., Kibuuka H., Bétard C., Richert L., Lacabaratz C. (2022). Safety and immunogenicity of 2-dose heterologous Ad26.ZEBOV, MVA-BN-Filo Ebola vaccination in children and adolescents in Africa: A randomised, placebo-controlled, multicentre phase II clinical trial. PLoS Med..

[16] Kieh M., Richert L., Beavogui A.H., Grund B., Leigh B., D’Ortenzio E., Doumbia S., Lhomme E., Sow S., PREVAC Study Team (2022). Randomized trial of vaccines for Zaire Ebola virus disease. N. Engl. J. Med..

[17] Manno D., Bangura A., Baiden F., Kamara A.B., Ayieko P., Kallon J., Foster J., Conteh M., Connor N.E., Koroma B. (2023). Safety and immunogenicity of an Ad26.ZEBOV booster dose in children previously vaccinated with the two-dose heterologous Ad26.ZEBOV and MVA-BN-Filo Ebola vaccine regimen: An open-label, non-randomised, phase 2 trial. Lancet Infect. Dis..

[18] Zabdeno EPAR—Product Information. https://www.ema.europa.eu/en/documents/product-information/zabdeno-epar-product-information_en.pdf.

[19] Mvabea EPAR—Product Information. https://www.ema.europa.eu/en/documents/product-information/mvabea-epar-product-information_en.pdf.

[20] World Health Organization Strategic Advisory Group of Experts (SAGE) on Immunization Interim Recommendations on Vaccination against Ebola Virus Disease (EVD). https://cdn.who.int/media/docs/default-source/immunization/ebola/interim-ebola-recommendations-may-2019.pdf?sfvrsn=c54ce264_9.

[21] Johnson & Johnson Johnson & Johnson Joins World Health Organization in Efforts to Prevent Spread of Ebola in West Africa. https://www.jnj.com/johnson-johnson-joins-world-health-organization-in-efforts-to-prevent-spread-of-ebola-in-west-africa.

[22] Freedman D.O., Chen L.H. (2019). Vaccines for international travel. Mayo Clin. Proc..

[23] Collier A.C., Corey L., Murphy V.L., Handsfield H.H. (1988). Antibody to human immunodeficiency virus (HIV) and suboptimal response to hepatitis B vaccination. Ann. Intern. Med..

[24] National Institute of Allergy and Infectious Diseases Division of Microbiology and Infectious Diseases (DMID) Adult Toxicity Table July 2017 Draft. https://rsc.niaid.nih.gov/sites/default/files/daidsgradingcorrectedv21.pdf.

[25] US Food and Drug Administration Guidance for Industry: Toxicity Grading Scale for Healthy Adult and Adolescent Volunteers Enrolled in Preventive Vaccine Clinical Trials. https://www.fda.gov/media/73679/download.

[26] Logue J., Tuznik K., Follmann D., Grandits G., Marchand J., Reilly C., Sarro Y.D.S., Pettitt J., Stavale E.J., Fallah M. (2018). Use of the Filovirus Animal Non-Clinical Group (FANG) Ebola virus immuno-assay requires fewer study participants to power a study than the Alpha Diagnostic International assay. J. Virol. Methods.

[27] Moncunill G., Dobaño C., McElrath M.J., De Rosa S.C. (2015). OMIP-025: Evaluation of human T- and NK-cell responses including memory and follicular helper phenotype by intracellular cytokine staining. Cytometry A.

[28] Lin L., Finak G., Ushey K., Seshadri C., Hawn T.R., Frahm N., Scriba T.J., Mahomed H., Hanekom W., Bart P.A. (2015). COMPASS identifies T-cell subsets correlated with clinical outcomes. Nat. Biotechnol..

[29] Paquin-Proulx D., Gunn B.M., Alrubayyi A., Clark D.V., Creegan M., Kim D., Kibuuka H., Millard M., Wakabi S., Eller L.A. (2021). Associations between antibody Fc-mediated effector functions and long-term sequelae in Ebola virus survivors. Front. Immunol..

[30] Roozendaal R., Hendriks J., van Effelterre T., Spiessens B., Dekking L., Solforosi L., Czapska-Casey D., Bockstal V., Stoop J., Splinter D. (2020). Nonhuman primate to human immunobridging to infer the protective effect of an Ebola virus vaccine candidate. NPJ Vaccines.

[31] Bockstal V., Leyssen M., Heerwegh D., Spiessens B., Robinson C., Stoop J.N., Roozendaal R., Van Effelterre T., Gaddah A., Van Roey G.A. (2022). Non-human primate to human immunobridging demonstrates a protective effect of Ad26.ZEBOV, MVA-BN-Filo vaccine against Ebola. NPJ Vaccines.

[32] Liu Q., Fan C., Li Q., Zhou S., Huang W., Wang L., Sun C., Wang M., Wu X., Ma J. (2017). Antibody-dependent-cellular-cytotoxicity-inducing antibodies significantly affect the post-exposure treatment of Ebola virus infection. Sci. Rep..

[33] Gunn B.M., Yu W.H., Karim M.M., Brannan J.M., Herbert A.S., Wec A.Z., Halfmann P.J., Fusco M.L., Schendel S.L., Gangavarapu K. (2018). A role for Fc function in therapeutic monoclonal antibody-mediated protection against Ebola virus. Cell Host Microbe.

[34] Saphire E.O., Schendel S.L., Fusco M.L., Gangavarapu K., Gunn B.M., Wec A.Z., Halfmann P.J., Brannan J.M., Herbert A.S., Qiu X. (2018). Systematic analysis of monoclonal antibodies against Ebola virus GP defines features that contribute to protection. Cell.

[35] Barouch D.H., Tomaka F.L., Wegmann F., Stieh D.J., Alter G., Robb M.L., Michael N.L., Peter L., Nkolola J.P., Borducchi E.N. (2018). Evaluation of a mosaic HIV-1 vaccine in a multicentre, randomised, double-blind, placebo-controlled, phase 1/2a clinical trial (APPROACH) and in rhesus monkeys (NHP 13-19). Lancet.

[36] Barouch D.H., Stephenson K.E., Borducchi E.N., Smith K., Stanley K., McNally A.G., Liu J., Abbink P., Maxfield L.F., Seaman M.S. (2013). Protective efficacy of a global HIV-1 mosaic vaccine against heterologous SHIV challenges in rhesus monkeys. Cell.

[37] Sullivan N.J., Hensley L., Asiedu C., Geisbert T.W., Stanley D., Johnson J., Honko A., Olinger G., Bailey M., Geisbert J.B. (2011). CD8+ cellular immunity mediates rAd5 vaccine protection against Ebola virus infection of nonhuman primates. Nat. Med..

[38] Madhi S.A., Moodley D., Hanley S., Archary M., Hoosain Z., Lalloo U., Louw C., Fairlie L., Fouche L.F., Masilela M.S.L. (2022). Immunogenicity and safety of a SARS-CoV-2 recombinant spike protein nanoparticle vaccine in people living with and without HIV-1 infection: A randomised, controlled, phase 2A/2B trial. Lancet HIV.

