# Supplementary material for: Safety and Immunogenicity of an Accelerated Ebola Vaccination Schedule in People with and without Human Immunodeficiency Virus: A Randomized Clinical Trial
Source: Vaccines (Basel). 2024 May 4;12(5):497. doi: 10.3390/vaccines12050497 (PMC11125575; doi:10.3390/vaccines12050497)
Supplement: Supplementary file 1 [file vaccines-12-00497-s001.zip › Supplement/Supplement S1. Study protocol.pdf]

Janssen Vaccines & Prevention B.V.

Clinical Protocol

A Randomized, Observer-blind, Placebo-controlled, Two-part, Phase 2 Study to Evaluate the Safety, Tolerability and Immunogenicity of Two Prime-boost Regimens of the Candidate Prophylactic Vaccines for Ebola Ad26.ZEBOV and MVA-BN-Filo

Protocol VAC52150EBL2003; Phase 2
AMENDMENT 5

Walter Reed Army Institute of Research and Janssen Vaccines & Prevention B.V.

IND Number: 16280

VAC52150 (Ad26.ZEBOV/MVA-BN-Filo [MVA-mBN226B])

*Janssen Vaccines & Prevention B.V. (formerly known as Crucell Holland B.V.) is a Janssen pharmaceutical company of Johnson & Johnson and is hereafter referred to as the sponsor of the study. The sponsor is identified on the Contact Information page that accompanies the protocol.

This study will be conducted under US Food & Drug Administration IND regulations (21 CFR Part 312).

**Status:**	Approved
**Date:**	18 September 2017
**EDMS number:**	EDMS-ERI-108856107, 13.0

GCP Compliance: This study will be conducted in compliance with Good Clinical Practice, and applicable regulatory requirements.

Confidentiality Statement
The information in this document contains trade secrets and commercial information that are privileged or confidential and may not be disclosed unless such disclosure is required by applicable law or regulations. In any event, persons to whom the information is disclosed must be informed that the information is privileged or confidential and may not be further disclosed by them. These restrictions on disclosure will apply equally to all future information supplied to you that is indicated as privileged or confidential.

**Protocol History VAC52150EBL2003_Protocol**		
Document Type and *File Name*	Issued Date	Comments
Initial Clinical Protocol *VAC52150EBL2003_Protocol*	21 September 2015	-
Protocol Amendment 1 *VAC52150EBL2003_Protocol_Amend_1*	6 November 2015	For details, see Section Amendment_1
Protocol Amendment 2 *VAC52150EBL2003_Protocol_Amend_2*	8 March 2016	For details, see Section Amendment_2
Protocol Amendment 3 *VAC52150EBL2003_Protocol_Amend_3*	3 November 2016	For details, see Section Amendment_3
Protocol Amendment 4 *VAC52150EBL2003_Protocol_Amend_4*	20 April 2017	For details, see Section Amendment_4
Protocol Amendment 5 *VAC52150EBL2003_Protocol_Amend_5*	This document	For details, see Section Amendment_5

TABLE OF CONTENTS

TABLE OF CONTENTS 3

LIST OF ATTACHMENTS 6

LIST OF IN-TEXT TABLES AND FIGURES 6

PROTOCOL AMENDMENT 7

SYNOPSIS 20

TIME AND EVENTS SCHEDULE 27

ABBREVIATIONS 33

DEFINITIONS OF TERMS 34

1. INTRODUCTION 35
1.1. Background 35
1.2. Benefit/Risk Section 42
1.2.1. Known Benefits 42
1.2.2. Potential Benefits 42
1.2.3. Known Risks 42
1.2.4. Potential Risks 42
1.2.5. Overall Benefit/Risk Assessment 43
1.3. Overall Rationale for the Study 45

2. OBJECTIVES AND HYPOTHESIS 46
2.1. Objectives 46
2.2. Hypothesis 48

3. STUDY DESIGN AND RATIONALE 48
3.1. Overview of Study Design 48
3.2. Study Design Rationale 53

4. SUBJECT POPULATION 54
4.1. Inclusion Criteria 55
4.1.1. Inclusion Criteria for Healthy Adult Subjects 55
4.1.2. Additional Inclusion Criteria for HIV-infected Subjects 57
4.2. Exclusion Criteria 58
4.2.1. Exclusion Criteria for Healthy Adult Subjects 58
4.2.2. Additional Exclusion Criteria for HIV-infected Subjects 60
4.3. Prohibitions and Restrictions 60

5. TREATMENT ALLOCATION AND BLINDING 60

6. DOSAGE AND ADMINISTRATION 63
6.1. General Instructions and Procedures 63
6.2. Criteria for Postponement of Vaccination 64
6.3. Contraindications to Boost Vaccination 64

7. TREATMENT COMPLIANCE 65

8. PRESTUDY AND CONCOMITANT THERAPY 65

9. STUDY EVALUATIONS 66
9.1. Study Procedures 66
9.1.1. Overview 66

9.1.2. Screening Phase 67
9.1.3. Vaccination Phase 69
9.1.4. Post-boost Follow-up 70
9.1.5. VAC52150 Vaccine Development Roll-over Study 71
9.2. Endpoints 71
9.2.1. Primary Endpoints 71
9.2.2. Secondary Endpoint 72
9.2.3. Exploratory Endpoints 72
9.3. Safety Evaluations 73
9.3.1. Safety Assessments 73
9.3.2. Pausing Rules 77
9.4. Immunogenicity Evaluations 78
9.5. Virologic Evaluations 79
9.6. Pharmacogenomic (DNA) Evaluations 79
9.7. Vaccine-induced Seropositivity 80
9.8. Sample Collection and Handling 80
9.9. Optional Procedures 80
9.9.1. Apheresis for Part 1 Subjects 80
9.9.2. Mucosal Secretions for Part 2 Subjects 81

10. SUBJECT COMPLETION/DISCONTINUATION OF STUDY VACCINE/WITHDRAWAL FROM THE STUDY 81
10.1. Completion 81
10.2. Discontinuation of Study Vaccine/Withdrawal From the Study 81
10.3. Withdrawal From the Use of Research Samples 83

11. STATISTICAL METHODS 83
11.1. Sample Size Determination 83
11.2. Analysis Sets 85
11.3. Subject Information 85
11.4. Safety Analyses 85
11.5. Immunogenicity Analyses 86
11.6. Interim Analyses 87
11.7. Independent Data Monitoring Committee 87
11.8. Protocol Safety Review Team and Protocol Safety Review Team Reviews 87

12. ADVERSE EVENT REPORTING 88
12.1. Definitions 88
12.1.1. Adverse Event Definitions and Classifications 88
12.1.2. Attribution Definitions 90
12.1.3. Severity Criteria 91
12.2. Special Reporting Situations 91
12.3. Procedures 91
12.3.1. All Adverse Events 91
12.3.2. Serious Adverse Events 93
12.3.3. Immediate Reportable Events 94
12.3.4. Pregnancy 94
12.4. Contacting Sponsor Regarding Safety 95

13. PRODUCT QUALITY COMPLAINT HANDLING 95
13.1. Procedures 95
13.2. Contacting Sponsor Regarding Product Quality 95

14. STUDY VACCINE INFORMATION 96
14.1. Description of Study Vaccines 96
14.2. Packaging and Labeling 96
14.3. Preparation, Handling, and Storage 96
14.4. Study Vaccine Accountability 97

15. STUDY-SPECIFIC MATERIALS ... 98

16. ETHICAL ASPECTS ... 98
16.1. Study-specific Design Considerations ... 98
16.2. Regulatory Ethics Compliance ... 99
16.2.1. Investigator Responsibilities ... 99
16.2.2. Independent Ethics Committee or Institutional Review Board ... 99
16.2.3. DoD Research Monitor ... 100
16.2.4. Informed Consent ... 101
16.2.5. Privacy of Personal Data ... 102
16.2.6. Long-term Retention of Samples for Additional Future Research ... 102
16.2.7. Country Selection ... 103

17. ADMINISTRATIVE REQUIREMENTS ... 103
17.1. Protocol Amendments ... 103
17.2. Regulatory Documentation ... 104
17.2.1. Regulatory Approval/Notification ... 104
17.2.2. Required Prestudy Documentation ... 104
17.3. Subject Identification, Enrollment, and Screening Logs ... 105
17.4. Source Documentation ... 105
17.5. Case Report Form Completion ... 105
17.6. Data Quality Assurance/Quality Control ... 106
17.7. Record Retention ... 106
17.8. Monitoring ... 107
17.9. Regulatory Audits ... 107
17.10. Study Completion/Termination ... 108
17.10.1. Study Completion/End of Study ... 108
17.10.2. Study Termination ... 108
17.11. On-site Audits ... 108
17.12. Site-specific addendum ... 108
17.13. Use of Information and Publication ... 109

REFERENCES ... 111

ATTACHMENTS ... 113

INVESTIGATOR AGREEMENT ... 120

LAST PAGE ... 120

LIST OF ATTACHMENTS

Attachment 1: Test of Understanding (TOU) ... 113
Attachment 2: Toxicity Tables for Use in Trials Enrolling Healthy Adults ... 114
Attachment 3: Hemoglobin Cut-off Values ... 119

LIST OF IN-TEXT TABLES AND FIGURES

TABLES

Table 1: Study Vaccination Schedules Parts 1 and 2 ... 63
Table 2: Visit Windows ... 67
Table 3: Summary of Immunologic Assessments in Serum and PBMC ... 79
Table 4: Probability of Observing at Least One Adverse Event Given a True Adverse Event Incidence ... 84
Table 5: Magnitude of Pairwise Difference to be Detected Between Vaccine Regimens for a Given Sample Size ... 84

FIGURES

Figure 1: Schematic Overview of the Study ... 23
Figure 2: Schematic Overview of the Study ... 51

PROTOCOL AMENDMENT

Amendment_5 (18 September 2017)

The overall reason for the amendment: This amendment was created to increase the number of mucosal sample participants in Part 2 of the study, and to remove 2 planned statistical analyses.

The changes made to the clinical protocol VAC52150EBL2003 are listed below, including the rationale of each change and a list of all applicable sections.

Rationale: As requested by the Walter Reed Army Institute of Research (WRAIR) the number of mucosal sampling participants was increased up to approximately 40% (n= 200) to allow for more robust comparisons between study populations.

SYNOPSIS

3.1 Overview of Study Design

Rationale: The note on subjects who have recently received treatment for acute, uncomplicated malaria was reworded as the previous wording was confusing.

4.2.1 Exclusion Criteria for Healthy Adult Subjects

Rationale: The protocol states that highly active antiretroviral therapy (HAART) must be recorded in the concomitant medication form as a prior medication and any changes to the subject's HAART regimen must also be recorded. This is not how this is currently captured. It needs to be entered on the HIV information page in the eCRF and not in the concomitant medications page.

8 PRESTUDY AND CONCOMITANT THERAPY

Rationale: Details on the immunogenicity analyses will be provided in the Statistical Analysis Plan, and were removed from the protocol.

11.5 Immunogenicity Analyses

Rationale: The planned interim analysis when all subjects in Part 1 have completed the 6-month post-boost visit or discontinued earlier and the primary analysis when all subjects have completed the 6-month post-boost visit or discontinued earlier, have been removed as unblinding has been delayed. In addition, an interim analysis when all subjects in Part 1 have completed the 1-year post-boost visit or discontinued earlier has been added.

SYNOPSIS

3.1 Overview of Study Design

5 TREATMENT ALLOCATION AND BLINDING

11 STATISTICAL METHODS

11.4 Safety Analyses

11.5 Immunogenicity Analyses

11.6 Interim Analyses

Rationale: The statistical methods for physical examinations were revised. Only listings will be generated.

SYNOPSIS

11.4 Safety Analyses

Rationale: Minor editorial changes have been made.

Throughout the document

Amendment_4 (20 April 2017)

The overall reason for the amendment: This amendment is developed in response to changes to the global clinical development plan. As of this amendment, the enrollment of subjects from VAC52150EBL2003 to the VAC52150EBL4001 Roll-over study is limited to female subjects in Part 2 who became pregnant with estimated conception within 28 days after vaccination with MVA-BN-Filo (or placebo) or within 3 months after vaccination with Ad26.ZEBOV (or placebo) and children born to those vaccinated female subjects, unless local regulations have additional requirements for follow up. After implementation of this amendment, no subjects of Part 1 will be approached anymore in this study to roll-over to the VAC52150EBL4001 study.

See also below for a more extensive rationale regarding this change.

The changes made to the clinical protocol VAC52150EBL2003 are listed below, including the rationale of each change and a list of all applicable sections.

Rationale: The original development plan (at the time of the ongoing Ebola epidemic in Africa) was an accelerated plan with the anticipation of conducting Phase 3 efficacy studies (with limited safety data collection) shortly after Phase 1 and in parallel with Phase 2. The sponsor designed the VAC52150EBL4001 study for the extended follow-up of SAEs to enhance the ability for signal detection of rare events. Since there is no longer an ongoing Ebola epidemic, it is not currently possible to conduct a parallel Phase 3 efficacy study as part of an accelerated development plan. More controlled safety data will have to become available for all vaccinated subjects, prior to any potential future efficacy study. In this study, the requirement for entrance to the VAC52150EBL4001 Roll-over study will be limited to female subjects in Part 2 who became pregnant with estimated conception within 28 days after vaccination with MVA-BN-Filo (or placebo) or within 3 months after vaccination with Ad26.ZEBOV (or placebo) and children born to those vaccinated female subjects, unless local regulations have additional requirements for follow up. After implementation of this amendment, no subjects of Part 1 will be approached anymore in this study to roll-over to the VAC52150EBL4001 study.

SYNOPSIS

3.1 Overview of Study Design

9.1.5 VAC52150 Vaccine Development Roll-over Study

12.3.4 Pregnancy

Amendment_3 (3 November 2016**)**

The overall reason for the amendment: The sponsor halted vaccinations in the clinical program following a case of Miller Fisher syndrome after receipt of MVA-BN-Filo or placebo in study VAC52150EBL2001 that required further assessment to rule out a neurologic and autoimmune event. The current study was paused until the updated study-specific informed consent form had been approved to inform the study participants on the additional safety information. Based on the request of the Agence Nationale de Sécurité du Médicament et des produits de santé (ANSM) for study VAC52150EBL2001 in response to the Miller Fisher case, the sponsor has decided to implement the collection of neurologic and autoimmune events ("Immediate Reportable Events") throughout the entire clinical development plan.

The changes made to the clinical protocol VAC52150EBL2003 are listed below, including the rationale of each change and a list of all applicable sections.

Rationale: As requested by the ANSM, wording on the collection of "Immediate Reportable Events" was added after one subject in the study VAC52150EBL2001 experienced a serious and very rare condition called "Miller Fisher syndrome" about a month after boost vaccination with either MVA-BN-Filo or placebo. Although the event was considered to be unrelated to the vaccine, measures were implemented for the entire clinical development plan.

SYNOPSIS

Time and Events Schedule

ABBREVIATIONS

1.1 Background

1.2.5 Overall Benefit/Risk Assessment

3.1 Overview of Study Design

8 PRESTUDY AND CONCOMITANT THERAPY

9.1.2 Screening Phase

9.1.3 Vaccination Phase

9.1.4 Post-boost Follow-up

9.2.1 Primary Endpoints

9.3.1 Safety Assessments

11.4 Safety Analyses

12.1.1 Adverse Event Definitions and Classifications

12.2 Special Reporting Situations

12.3.1 All Adverse Events

12.3.3 Immediate Reportable Events

Rationale: As a consequence of the pause, delays in unblinding are expected. Further details regarding enrollment into the VAC52150 roll-over study have been added, such as the inclusion of placebo subjects before unblinding of the current study.

SYNOPSIS

3.1 Overview of Study Design

9.1.5 VAC52150 Vaccine Development Roll-over Study

Rationale: Adverse events of special interest (cardiovascular events) will no longer be collected as no cardiovascular events have been associated with the current MVA-BN-Filo vaccine. Information was added on the procedure that needs to be followed in case any cardiac event develops.

1.2.5 Overall Benefit/Risk Assessment

9.3.1 Safety Assessments

12.2 Special Reporting Situations

12.3.1 All Adverse Events

Rationale: The secondary immunogenicity objectives and endpoints were shifted to exploratory objectives and endpoints.

SYNOPSIS

2.1 Objectives

9.2.1 Primary Endpoints

9.2.2 Secondary Endpoint

9.2.3 Exploratory Endpoints

9.4 Immunogenicity Evaluations

Rationale: Information regarding rescreening of subjects was added.

SYNOPSIS

Time and Events Schedule

3.1 Overview of Study Design

4.1.2 Additional Inclusion Criteria for HIV-infected Subjects

9.1.2 Screening Phase

Rationale: Information was added to clarify the procedures that need to be followed for subjects who are affected by a study pause in the future.

SYNOPSIS

3.1 Overview of Study Design

Rationale: Name change from Crucell Holland B.V. to Janssen Vaccines & Prevention B.V.

Title Page

1 INTRODUCTION

14.1 Description of Study Vaccines

16.2.3 DoD Research Monitor

REFERENCES

INVESTIGATOR AGREEMENT

Rationale: Safety information following MVA-BN-Filo vaccine administration based on the pooled safety data from studies VAC52150EBL1001 and VAC52150EBL1002 has been included.

1.1 Background

1.2.3 Known Risks

1.2.4 Potential Risks

1.3 Overall Rationale for the Study

Rationale: As requested by the Walter Reed Army Institute of Research (WRAIR), a question in the Test of Understanding was changed to avoid confusion in the number of visits.

Attachment 1: Test of Understanding (TOU)

Rationale: Clarification was added with regard to which information can be considered as source documentation.

17.4 Source Documentation

Rationale: Information with regard to the stability of highly active antiretroviral therapy was added.

4.1.2 Additional Inclusion Criteria for HIV-infected Subjects

---

Rationale: The statistical methods for physical examination, vital signs, and clinical laboratory tests were revised. For these parameters, only abnormalities will be tabulated by worst abnormality grade.

SYNOPSIS

11.4 Safety Analyses

Rationale: The protocol has been updated in line with the current protocol template (version 6 June 2016).

4.1.1 Inclusion Criteria for Healthy Adult Subjects

10.2 Discontinuation of Study Vaccine/Withdrawal From the Study

16.2.7 Country Selection

17.1 Protocol Amendments

17.5 Case Report Form Completion

Rationale: Minor textual changes have been made, in addition to modifications for clarity and updates to be in line with other current protocols and the current Investigator Brochures.

SYNOPSIS

Time and Events Schedule

ABBREVIATIONS

1.1 Background

1.2.5 Overall Benefit/Risk Assessment

2.1 Objectives

3.1 Overview of Study Design

3.2 Study Design Rationale

5 TREATMENT ALLOCATION AND BLINDING

9.1.4 Post-boost Follow-up

10.1 Completion

10.2 Discontinuation of Study Vaccine/Withdrawal From the Study

11.1 Sample Size Determination

11.2 Analysis Sets

12.1.1 Adverse Event Definitions and Classifications

14.1 Description of Study Vaccines

REFERENCES

Amendment_2 (8 March 2016)

The overall reason for the amendment: This amendment was created to be in alignment with the VAC52150EBL2001 and VAC52150EBL2002 protocols, to remove a planned interim analysis that was erroneously added, and to add an additional planned interim analysis.

The changes made to the clinical protocol VAC52150EBL2003 are listed below, including the rationale of each change and a list of all applicable sections.

Rationale: The planned interim analysis in Part 1 and Part 2 (when all subjects in that part have completed the 42-day post-boost visit or discontinued earlier), has been removed since the primary analysis will be performed on unblinded data at the 6 months post-boost database lock. It has been clarified that the interim analysis in Part 1 (when all subjects in Part 1 have completed the 21-day post-boost visit or discontinued earlier) will be evaluated only from a safety perspective. An additional planned interim analysis will be done when all subjects in Part 1 have completed the 6-month post-boost visit or discontinued earlier.

SYNOPSIS

3.1 Overview of Study Design

11.5 Immunogenicity Analyses

11.6 Interim Analyses

Rationale: The blood volume for the CD4+ cell count (only for HIV-infected subjects) has been increased to 4 mL. The blood volume for HIV viral load quantification and sequencing (only for HIV-infected subjects) at screening has been increased to 6 mL. Daily and cumulative blood volumes have been updated accordingly. Maximum daily and cumulative blood draws have been changed to approximate daily and cumulative blood draws to allow for retesting in the event of sample hazard.

Time and Events Schedule

9.1.1 Overview

9.3.1 Safety Assessments

9.5 Virologic Evaluations

16.1 Study-specific Design Considerations

Rationale: The statement that a male subject's study vaccine should be permanently discontinued if his partner becomes pregnant, has been removed. The current biodistribution and reprotoxicity data support the recommendation that there is a negligible risk to the partner of a male vaccinated subject if she becomes pregnant.

10.2 Discontinuation of Study Vaccine

Rationale: The statement that the birth control method used by the female partner of a male subject should be documented, has been removed. The current biodistribution data suggest that there is no vaccine present in tissues other than the inoculated muscle and regional lymph nodes. Therefore, the risk to the partner of a male participant is negligible and it is unnecessary to confirm additional contraceptive use by the partner. Consistent condom use in the male participant will continue to be emphasized.

4.1.1 Inclusion Criteria for Healthy Adult Subjects

4.3 Prohibitions and Restrictions

Rationale: Immunogenicity objectives, endpoints and assessments have been revised and corrected. The secondary objective 'to assess the kinetics and durability of cellular immune response in healthy and HIV-infected adults for EBOV GP insert' has been moved to the exploratory objectives and the corresponding secondary endpoint has been moved to the exploratory endpoints.

SYNOPSIS

2.1 Objectives

9.2.2 Secondary Endpoint

9.2.3 Exploratory Endpoints

9.4 Immunogenicity Evaluations

Rationale: **S**ubjects who received placebo and reach the 1-year post-boost visit (Part 1 and Part 2, Group 2: Day 380; Part 2, Group 1: Day 394) prior to unblinding will be required to attend the 1-year post-boost visit.

SYNOPSIS

Time and Events Schedule

3.1 Overview of Study Design

5 TREATMENT ALLOCATION AND BLINDING

9.1.4 Post-boost Follow-up

Rationale: To guarantee a sufficient number of subjects, up to 20 subjects (10 HIV-infected, 10 healthy) from Part 1 will be asked to enroll for optional apheresis for collection of peripheral blood mononuclear cell and plasma, given that they have provided specific consent for this procedure. Up to a total of 10 Part 1 subjects (irrespective of HIV status) with robust immune responses post-boost may also be enrolled for an optional apheresis procedure.

Time and Events Schedule

9.9.1 Apheresis for Part 1 Subjects

Rationale: The description of the doubtful case of pericarditis in the MVA-BN clinical trial program was updated.

1.2.4 Potential Risks

Rationale: Fainting was added as a risk from blood draws.

1.2.4 Potential Risks

Rationale: Inclusion criterion 4 has been modified to specify that subjects with hemoglobin lower than the institutional normal reference range should not be included in the study.

4.1.1 Inclusion Criteria for Healthy Adult Subjects

Rationale: The note to inclusion criterion 12 has been modified to clarify that if subjects fail the test of understanding (TOU) on the third attempt, they should not continue with the consenting and screening processes.

4.1.1 Inclusion Criteria for Healthy Adult Subjects

Rationale: Inclusion criteria 5 and 7 have been modified to clarify that birth control methods should be used until at least 3 months after the boost vaccination.

4.1.1 Inclusion Criteria for Healthy Adult Subjects

Rationale: The 21-day post-boost visit has been added as time point when differences between the vaccination schedules will be described.

SYNOPSIS

11.5 Immunogenicity Analyses

Rationale: The supine position while resting before ECG collection is not required and has therefore been removed. The order of safety procedures (vital signs, ECG[s], blood draw) has been removed.

9.3.1 Safety Assessments

Rationale: Further details regarding the VAC52150 Vaccine Development Roll-over study have been added. The statement '(non-participating) partners of male subjects on active vaccine who become pregnant during the study and up to 3 months after the prime vaccination will be approached to consent for enrollment of their children into the registry' has been removed since these children are not part of this roll-over study.

SYNOPSIS
3.1 Overview of Study Design
9.1.5 VAC52150 Vaccine Development Roll-over Study
12.3.4 Pregnancy

Rationale: The name of the participating subject, date, reviewer of the test, and the score has been added to the TOU.

Attachment 1: Test of Understanding (TOU)

Rationale: Minor textual changes have been made, in addition to modifications for clarity and updates to be in line with other current protocols.

SYNOPSIS
Time and Events Schedule
1 INTRODUCTION
2.1 Objectives
3.1 Overview of Study Design
4 SUBJECT POPULATION
5 TREATMENT ALLOCATION AND BLINDING
6.3 Contraindications to Boost Vaccination
9.1.1 Overview
9.1.2 Screening Phase
9.1.3 Vaccination Phase
9.1.4 Post-boost Follow-up
9.3 Safety Evaluations
9.7 Vaccine-induced Seropositivity
9.9.2 Mucosal Secretions for Part 2 Subjects
10.2 Discontinuation of Study Vaccine
10.3 Withdrawal From the Use of Research Samples
12.1.1 Adverse Event Definitions and Classifications
12.3.1 All Adverse Events
12.3.4 Pregnancy
16.1 Study-specific Design Considerations
16.2.4 Informed Consent
16.2.6 Long-term Retention of Samples for Additional Future Research
16.2.7 Country Selection
17.5 Case Report Form Completion
17.6 Data Quality Assurance/Quality Control
17.7 Record Retention

17.8 Monitoring
REFERENCES
Attachment 2: Toxicity Tables for Use in Trials Enrolling Healthy Adults

Amendment_1 (6 November 2015)

The overall reason for the amendment: This amendment includes the request of the Center for Biologics Evaluation and Research (CBER, a division of US Food and Drug Administration [FDA]) to extend the safety follow-up to 6 months post-boost. This request was originally made for protocols VAC52150EBL3002 and VAC52150EBL3003 and has now also been implemented for this protocol. In addition, specific clarifications and corrections to the protocol as requested by the Walter Reed Army Institute of Research (WRAIR) Institutional Review Board (IRB), Human Subjects Protection Branch (HSPB), and Human Research Protection Office (HRPO) have been included.

The changes made to the clinical protocol VAC52150EBL2003, dd. 21-Sep-2015, are listed below, including the rationale of each change and a list of all applicable sections.

Rationale: As requested by the CBER (US FDA), the 6-month and 1-year visits have been changed to 6-month post-boost and 1-year post-boost visits.

SYNOPSIS
Time and Events Schedule
1.2.5 Overall Benefit/Risk Assessment
3.1 Overview of Study Design
3.2 Study Design Rationale
4.3 Prohibitions and Restrictions
5 TREATMENT ALLOCATION AND BLINDING
9.1.1 Overview
9.1.4 Post-boost Follow-up
9.3.1 Safety Assessments
10.1 Completion
11 STATISTICAL METHODS
11.5 Immunogenicity Analyses
16.1 Study-specific Design Considerations

Rationale: As requested by the CBER (US FDA), the safety laboratory assessments at screening are to be performed within 28 days prior to the prime vaccination and may be repeated if they fall outside this time window.

Time and Events Schedule
4.1.1 Inclusion Criteria for Healthy Adult Subjects
9.1.2 Screening Phase

Rationale: The blood sampling volume for cellular assays has been updated due to a switch to Heparin tubes.

Time and Events Schedule
9.1.1 Overview
9.4 Immunogenicity Evaluations
16.1 Study-specific Design Considerations

Rationale: Footnote "d" related to apheresis for collection of peripheral blood mononuclear cell (PBMC) and plasma in a subset of subjects in Part 1 has been added to the blood sampling for cellular assays assessment in the Time and Events Schedule. In addition, it has been clarified that the optional apheresis procedure in up to a total of 10 Part 1 subjects (irrespective of human immunodeficiency virus [HIV] status) with robust immune responses post-boost will be performed after unblinding at a predefined visits or at an unscheduled visit.

Time and Events Schedule

9.9.1 Apheresis for Part 1 Subjects

Rationale: As requested by the WRAIR HSPB, the description of the vaccination schedule has been reworded.

SYNOPSIS

3.1 Overview of Study Design

Rationale: It has been added that adherence to highly active antiretroviral therapy (HAART) will be assessed at every visit in HIV-infected subjects.

Time and Events Schedule

8 PRESTUDY AND CONCOMITANT THERAPY

9.1.3 Vaccination Phase

9.1.4 Post-boost Follow-up

Rationale: The optional consent for mucosal collection in Part 2 has been added to the Time and Events Schedule.

Time and Events Schedule

Rationale: It has been added that stored serum or plasma of HIV-infected subjects may be used to assess antiretroviral drug levels.

Time and Events Schedule

9.5 Virologic Evaluations

Rationale: As requested by the WRAIR IRB, text has been added regarding the identification of an estimate of the number of subjects that may be screened in the site-specific addendum.

9.1.2 Screening Phase

Rationale: Because urinalysis is only performed at screening, the reference to unexplained hematuria has been removed from the pausing rules.

9.3.2 Pausing Rules

Rationale: Details about the collection of oral secretion samples have been added.

9.9.2.2 Collection of Oral Secretion Samples

Rationale: As requested by the WRAIR HSPB, it has been clarified that the interim analysis conducted when all subjects in Part 1 have completed the 21-day post-boost visit will be reviewed by the Protocol Safety Review Team (PSRT).

SYNOPSIS

3.1 Overview of Study Design

11.6 Interim Analyses

**Rationale:** As requested by the WRAIR HSPB, it has been clarified that there will be a single PSRT in which all of the Department of Defense (DoD) Research Monitors from each participating site will participate. It has been made clear that the list of additional participants in the PSRT is non exhaustive and that the PSRT will also review summaries for the Independent Data Monitoring Committee (IDMC) and make clarifications for the IDMC as needed.
11.8 Protocol Safety Review Team and Protocol Safety Review Team Reviews
**Rationale:** As requested by the WRAIR HRPO, the site-specific addendum and site-specific addendum amendments have been added to the list of documents that will be provided to the Independent Ethics Committee or Institutional Review Board before the start of the study and during the study, respectively.
16.2.2 Independent Ethics Committee or Institutional Review Board
**Rationale:** As requested by the WRAIR HSPB, the DoD Research Monitor's responsibilities have been revised.
16.2.3 DoD Research Monitor
**Rationale:** One possible albeit doubtful case of pericarditis observed in the MVA-BN clinical trial program has been added to the Potential Risks section.
1.2.4 Potential Risks
**Rationale:** Information regarding the marketing authorization of MVA-BN and the Phase 3 clinical study POX-MVA-013 has been updated.
1.1 Background<br>REFERENCES
**Rationale:** The note on abstinence or natural family planning under inclusion criterion 5 has been revised.
4.1.1 Inclusion Criteria for Healthy Adult Subjects
**Rationale:** It has been clarified that in study VAC52150EBL1002 no serious adverse events related to vaccination have been reported to date.
1.1 Background
**Rationale:** The protocol has been updated to be in line with the current protocol template (version 14 October 2015).
4 SUBJECT POPULATION<br>9.3.1 Safety Assessments<br>10 SUBJECT COMPLETION/DISCONTINUATION OF STUDY VACCINE/WITHDRAWAL FROM THE STUDY<br>12.2 Special Reporting Situations<br>12.4 Contacting Sponsor Regarding Safety<br>13.2 Contacting Sponsor Regarding Product Quality<br>16.2.2 Independent Ethics Committee or Institutional Review Board<br>17.1 Protocol Amendments<br>17.4 Source Documentation<br>17.5 Case Report Form Completion<br>17.8 Monitoring<br>17.10.1 Study Completion/End of Study<br>17.11 On-site Audits

17.13 Use of Information and Publication
**Rationale:** Minor errors have been corrected and minor textual changes have been made.
SYNOPSIS<br>Time and Events Schedule<br>1.2.4 Potential Risks<br>3.1 Overview of Study Design<br>4.1.2 Additional Inclusion Criteria for HIV-infected Subjects<br>5 TREATMENT ALLOCATION AND BLINDING<br>8 PRESTUDY AND CONCOMITANT THERAPY<br>9.1.1 Overview<br>9.1.2 Screening Phase<br>9.9.2.1 Collection of Genital and Rectal Secretion Samples<br>11.7 Independent Data Monitoring Committee<br>11.8 Protocol Safety Review Team and Protocol Safety Review Team Reviews<br>16.2.3 DoD Research Monitor

SYNOPSIS

A Randomized, Observer-blind, Placebo-controlled, Two-part, Phase 2 Study to Evaluate the Safety, Tolerability and Immunogenicity of Two Prime-boost Regimens of the Candidate Prophylactic Vaccines for Ebola Ad26.ZEBOV and MVA-BN-Filo.

The sponsor, in collaboration with Bavarian Nordic (BN) and Walter Reed Army Institute of Research (WRAIR), is investigating the potential of a prophylactic Ebola vaccine regimen comprised of the following 2 candidate Ebola vaccines:

Ad26.ZEBOV is a monovalent vaccine expressing the full length Ebola virus (EBOV, formerly known as *Zaire ebolavirus*) Mayinga glycoprotein (GP), and is produced in the human cell line PER.C6®.

MVA-mBN226B, further referred to as Modified Vaccinia Ankara (MVA)-BN-Filo®, is a multivalent vaccine expressing the Sudan virus (SUDV) GP, the EBOV GP, the Marburg virus (MARV) Musoke GP, and the Tai Forest virus (TAFV, formerly known as Côte d'Ivoire ebolavirus) nucleoprotein (NP), and is produced in chicken embryo fibroblast cells. The EBOV GP expressed by MVA-BN-Filo has 100% homology to the one expressed by Ad26.ZEBOV.

Two populations will be studied in this protocol: healthy adults and human immunodeficiency virus (HIV)-infected adults, ages 18-70 years inclusive, will be enrolled.

Ad26.ZEBOV and MVA-BN-Filo will be evaluated as a heterologous prime-boost regimen. The first schedule to be evaluated is prime vaccination with MVA-BN-Filo followed by a boost with Ad26.ZEBOV 14 days later. In the second schedule, Ad26.ZEBOV is used to prime a filovirus-specific immune response and MVA-BN-Filo is used to boost the immune response 28 days later. The EBOV GP that circulated in West Africa has 97% homology to the EBOV GP used in this vaccine regimen.

STUDY RATIONALE

- Enlarge the safety and immunogenicity database for the Ad26.ZEBOV/MVA-BN-Filo 1, 29-day prime-boost regimen in healthy adults.
- Assess the safety and immunogenicity of Ad26.ZEBOV/MVA-BN-Filo 1, 29-day prime-boost regimen in HIV-infected subjects and determine the effect of vaccination on HIV disease.
- Enlarge the safety and immunogenicity database for MVA-BN-Filo/Ad26.ZEBOV 1, 15-day prime-boost regimen in healthy adults and HIV-infected adults.

OBJECTIVES AND HYPOTHESIS

Primary Objectives

- To assess the safety and tolerability of different vaccination schedules of Ad26.ZEBOV and MVA-BN-Filo administered intramuscularly (IM) as heterologous prime-boost regimens in healthy adults and in HIV-infected adults, with Ad26.ZEBOV prime and MVA-BN-Filo boost vaccination on Days 1 and 29, respectively and MVA-BN-Filo prime and Ad26.ZEBOV boost vaccination on Days 1 and 15, respectively.
- To assess the immune responses to the EBOV GP (as measured by [enzyme-linked immunosorbent assay] ELISA antibody concentration) of different vaccination schedules of Ad26.ZEBOV and MVA-BN-Filo administered IM as heterologous prime-boost regimens in healthy adults and in HIV-infected adults, with Ad26.ZEBOV prime and MVA-BN-Filo boost vaccination on Days 1 and 29, respectively and MVA-BN-Filo prime and Ad26.ZEBOV boost vaccination on Days 1 and 15, respectively.

Secondary Objective

To compare safety and tolerability of both Ad26.ZEBOV/MVA-BN-Filo and MVA-BN-Filo/Ad26.ZEBOV regimens between healthy and HIV-infected adults.

Exploratory Objectives

The exploratory objectives include the following. Some exploratory objectives may not be performed if reagents or assays are not available.

- To assess the humoral immune responses further regarding kinetics and durability as well as neutralizing antibody responses directed against EBOV GP induced by the heterologous prime-boost regimen as measured by a virus neutralization assay, at relevant time points of different vaccination schedules of Ad26.ZEBOV and MVA-BN-Filo.
- To assess the kinetics and durability of cellular immune responses in healthy and HIV-infected adults for EBOV GP, by vaccine regimen.
- To compare kinetics and durability of humoral and cellular immune responses to EBOV GP of Ad26.ZEBOV/MVA-BN-Filo and MVA-BN-Filo/Ad26.ZEBOV regimens between healthy and HIV-infected adults.
- To assess the kinetics and durability of humoral immune responses, including the development of neutralizing antibodies, in healthy and HIV-infected adults for other filovirus GP.
- To assess the kinetics and durability of cellular immune responses in healthy and HIV-infected adults for other filovirus GP.
- To compare kinetics and durability of humoral and cellular immune responses to other filovirus GP, of Ad26.ZEBOV/MVA-BN-Filo and MVA-BN-Filo/Ad26.ZEBOV regimens between healthy and HIV-infected adults.
- To assess the frequency, magnitude, and durability of anti-vector responses in healthy and HIV-infected adults.
- To compare kinetics and durability of humoral and cellular immune responses to the EBOV and other filovirus GP of Ad26.ZEBOV/MVA-BN-Filo and MVA-BN-Filo/Ad26.ZEBOV regimens between adults aged 18-50 and aged 51-70.
- To assess the impact of CD4+ count on safety and immunogenicity of Ad26.ZEBOV/MVA-BN-Filo and MVA-BN-Filo/Ad26.ZEBOV regimens in HIV-infected adults.
- To assess changes in HIV ribonucleic acid (RNA) and HIV specific immune response (using HIV antigens in place of Ebola GP) associated with vaccination with Ad26.ZEBOV/MVA-BN-Filo and MVA-BN-Filo/Ad26.ZEBOV regimens in HIV-infected adults.
- To assess humoral immune responses in genital, rectal and oral secretions in a subset of healthy and HIV-infected adults at selected sites for EBOV and other filovirus GP.
- To explore the impact of host genetics on immune responses to the Ad26.ZEBOV/MVA-BN-Filo and MVA-BN-Filo/Ad26.ZEBOV prime-boost regimens in healthy and HIV-infected adults.
- To assess immune epitope breadth elicited by the Ad26.ZEBOV/MVA-BN-Filo and MVA-BN-Filo/Ad26.ZEBOV prime-boost regimens in healthy and HIV-infected adults.
- To assess the immunoglobulin subclass, glycosylation and effector functions of humoral responses to EBOV and other filovirus GP.

- To describe B cell, helper T cell, and cytotoxic T cell responses elicited by the Ad26.ZEBOV/MVA-BN-Filo and MVA-BN-Filo/Ad26.ZEBOV prime-boost regimens in healthy and HIV-infected adults.
- To explore immune inflammatory responses elicited by the Ad26.ZEBOV/MVA-BN-Filo and MVA-BN-Filo/Ad26.ZEBOV regimens in healthy and HIV-infected adults on a multiplex array platform to evaluate cytokine and soluble factor responses to vaccination.

Hypothesis

No formal statistical hypothesis testing is planned for this study. The primary purpose of the study is to provide descriptive information regarding safety and immunogenicity of the 2 vaccination regimens within cohorts of healthy and HIV-infected subjects.

OVERVIEW OF STUDY DESIGN

This is a randomized, observer-blind, placebo-controlled, parallel-group, multicenter, 2-part, Phase 2 study to evaluate the safety, tolerability and immunogenicity of different vaccination regimens using Ad26.ZEBOV at a dose of $5x10^{10}$ viral particles (vp) and MVA-BN-Filo at a dose of $1x10^{8}$ infectious units (Inf U, nominal titer), administered IM. The first regimen to be evaluated is prime vaccination with MVA-BN-Filo followed by a boost vaccination with Ad26.ZEBOV 14 days later. The second regimen to be evaluated is prime vaccination with Ad26.ZEBOV followed by a boost vaccination with MVA-BN-Filo 28 days later. These regimens will be evaluated in healthy and HIV-infected adults.

A planned total of 575 subjects will be enrolled, with approximately 300 healthy subjects and 275 HIV-infected subjects. Eligible subjects are those who have never received a candidate Ebola vaccine and have no prior exposure to EBOV (including travel to epidemic Ebola areas less than 1 month prior to screening) or a diagnosis of Ebola virus disease.

There are 2 parts to this study that will run in parallel. Part 1 features 2 cohorts consisting of 50 healthy adults and 25 HIV-infected adults, aged 18-70 years inclusive. Within each cohort, subjects will be randomized in a 4:1 ratio to receive Ad26.ZEBOV and MVA-BN-Filo or placebo: MVA-BN-Filo prime vaccination followed by Ad26.ZEBOV boost vaccination or placebo prime and boost vaccination 14 days later. Randomization will be done separately for healthy subjects and HIV-infected subjects. Part 1 will be conducted exclusively in the US at the WRAIR Clinical Research Center.

Part 2 of the study will investigate 2 vaccination regimens: Ad26.ZEBOV prime vaccination followed by MVA-BN-Filo boost vaccination 28 days later (Group 1) and MVA-BN-Filo prime vaccination followed by Ad26.ZEBOV boost vaccination 14 days later (Group 2). Group 1 includes 2 cohorts consisting of 200 healthy adults and 200 HIV-infected adults, aged 18-70 years inclusive, randomized in a 4:1 ratio to receive Ad26.ZEBOV and MVA-BN-Filo or placebo: Ad26.ZEBOV as prime vaccination followed by MVA-BN-Filo as boost vaccination or placebo prime and boost vaccination 28 days later. Group 2 includes 2 cohorts consisting of 50 healthy adults and 50 HIV-infected adults, aged 18-70 years inclusive, randomized in a 4:1 ratio to receive Ad26.ZEBOV and MVA-BN-Filo or placebo: MVA-BN-Filo prime vaccination followed by Ad26.ZEBOV boost vaccination or placebo prime and boost vaccination 14 days later, as shown in Figure 1 and Table 1. Randomization will be done separately for healthy subjects and HIV-infected subjects. Randomization of HIV-infected subjects in Part 2 will be contingent upon acceptable safety data from HIV-infected subjects from Part 1. An overview of the study design is provided below:

Figure 1: Schematic Overview of the Study

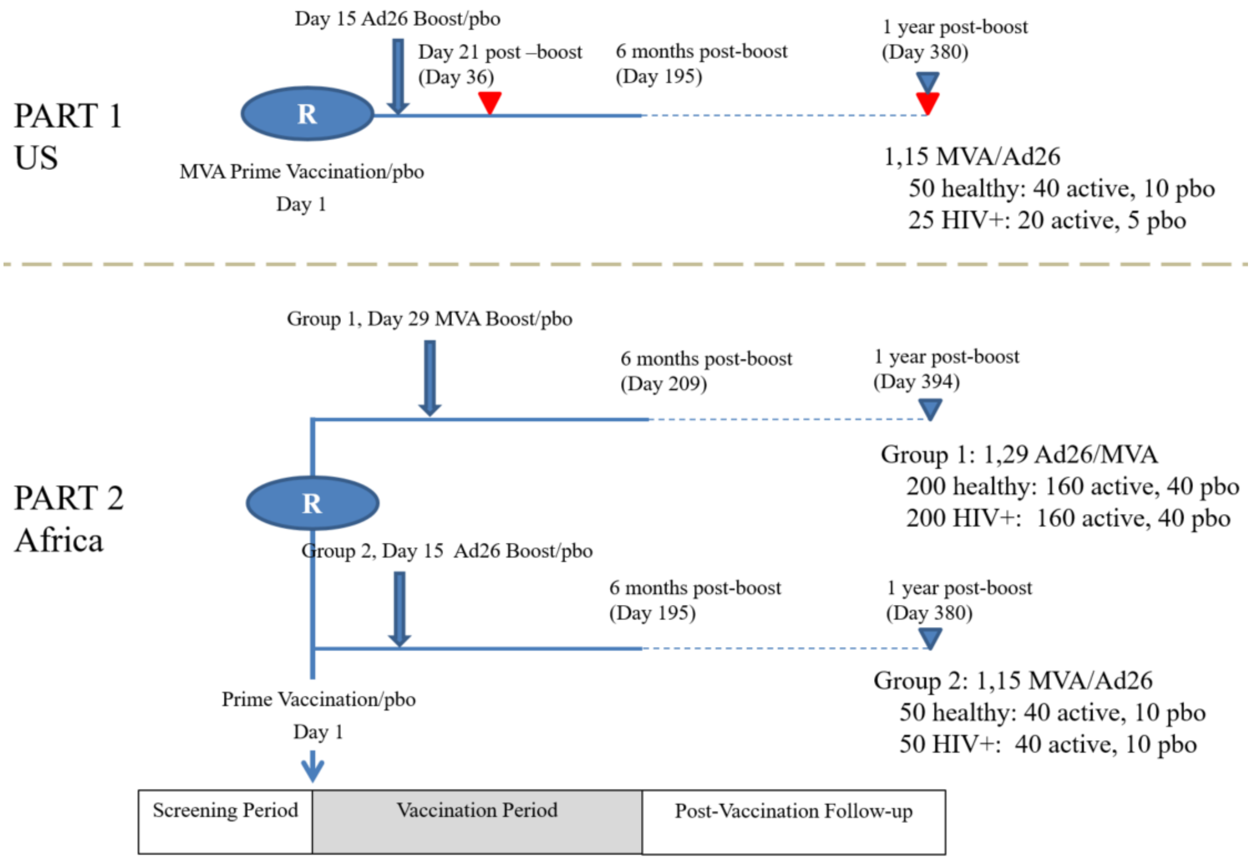

HIV+: HIV-infected; pbo: placebo; ▼: time of interim analysis; ▼: time of final analysis

An Independent Data Monitoring Committee (IDMC) will be commissioned for this study. Refer to Section 11.7 for details.

Within each part, study-site personnel (except for those with primary responsibility for study vaccine preparation and dispensing), sponsor personnel and subjects will be blinded to the study vaccine allocation until all subjects in that part have completed the 6-month post-boost visit or discontinued earlier and the database has been locked for that part. For any interim analyses performed in a part before database lock, study-site personnel (except for those with primary responsibility for study vaccine preparation and dispensing), the sponsor (except for specifically designated sponsor personnel who are independent of the study) and subjects will remain blinded to study vaccine allocation.

All subjects will receive the study vaccine (Ad26.ZEBOV, MVA-BN-Filo or placebo) IM in the deltoid muscle. In Part 1 and Part 2, Group 2, all subjects will receive MVA-BN-Filo ($1x10^8$ Inf U, nominal titer) or placebo on Day 1 followed by Ad26.ZEBOV ($5x10^{10}$ vp) or placebo on Day 15. In Part 2, Group 1, all subjects will receive Ad26.ZEBOV ($5x10^{10}$ vp) or placebo on Day 1, followed by MVA-BN-Filo ($1x10^8$ Inf U, nominal titer) or placebo on Day 29.

The study consists of a screening phase of up to 8 weeks (starting from the moment the subject signs the informed consent form [ICF]), a vaccination phase, in which subjects will be vaccinated at baseline (Day 1) followed by a boost vaccination on Day 15 or 29, and a post-boost follow-up phase of maximum 1 year post-boost vaccination. When all subjects in a part have completed the 6-month post-boost visit or discontinued earlier, and the database for that part has been locked, the part will be unblinded. At that time, the subjects who received placebo will be contacted by the site to communicate that they have completed the study and no further follow-up is required. The subjects who received Ad26.ZEBOV and

MVA-BN-Filo will continue the study until the 1-year post-boost vaccination visit to assess long-term safety and immunogenicity. However, subjects who received placebo and reach the 1-year post-boost visit (Part 1 and Part 2, Group 2: Day 380; Part 2, Group 1: Day 394) prior to unblinding will be required to attend the 1-year post-boost visit.

While Part 1 was enrolling, the sponsor halted all vaccinations in this study due to the occurrence of a serious and very rare condition, Miller Fisher syndrome, reported in study VAC52150EBL2001, until a thorough medical review was performed to ensure safety of the subjects, a revised ICF was prepared, and approval to restart the study was granted by the relevant competent authority. As a result of the delay, some subjects in Part 1 may reach the Day 380 visit prior to unblinding of Part 1. Female subjects in Part 2 who became pregnant with estimated conception within 28 days after vaccination with MVA-BN-Filo (or placebo) or within 3 months after vaccination with Ad26.ZEBOV (or placebo) and children born to vaccinated female subjects in Part 2 who became pregnant with estimated conception within 28 days after vaccination with MVA-BN-Filo (or placebo) or within 3 months after vaccination with Ad26.ZEBOV (or placebo) (unless local regulations have additional requirements for follow up) will be eligible for enrollment into the VAC52150EBL4001 Vaccine Development Roll-over study for long-term surveillance (for a total of up to 60 months after the prime vaccination). After unblinding, only female subjects and the children born to female subjects (with estimated conception within the period stated above) in Part 2 who received Ad26.ZEBOV and/or MVA-BN-Filo will be approached to consent for enrollment in the VAC52150 Vaccine Development Roll-over study for long-term surveillance. After unblinding, female subjects and the children born to female subjects (with estimated conception within the period stated above) in Part 2 who received placebo and had already been enrolled into the VAC52150 Vaccine Development Roll-over study will be discontinued from further participation in the roll-over study.

As of amendment 4 implementation, no subjects of Part 1 will be approached anymore in this study to roll-over to the VAC52150EBL4001 study.

In the event that the study is paused in the future, subjects who were primed and awaiting boost will be followed every 3 months until 1 year for ascertainment of serious adverse events and immediate reportable events (IREs). Subjects who received both prime and boost will continue to follow the protocol as written. Subjects who are awaiting prime vaccination may need to be rescreened under a new subject number, repeating all screening procedures except the test of understanding (TOU) (unless the TOU has been revised in the meantime, in which case it needs to be taken again).

SUBJECT POPULATION

Screening of subjects for eligibility will be performed within 8 weeks before administration of study vaccine on Day 1. The study population will consist of a planned total number of 575 subjects, including healthy and HIV-infected subjects (aged 18 to 70 years inclusive).

DOSAGE AND ADMINISTRATION

Study vaccines (Ad26.ZEBOV, MVA-BN-Filo or placebo) will be administered as 0.5-mL IM injections into the deltoid muscle. The boost vaccination should be administered in the opposite deltoid from the prime vaccination.

All subjects will receive a vaccination, according to randomization, on Day 1 and on Day 15 (Part 1 and Part 2, Group 2) or on Day 29 (Part 2, Group 1) at the following dose levels:

- Ad26.ZEBOV: $5x10^{10}$ vp, supplied in a single use vial (0.5 mL extractable)
- MVA-BN-Filo: $1x10^{8}$ Inf U (nominal titer; target fill is $1.9x10^{8}$ Inf U per dose, range: $1.27\text{-}2.67x10^{8}$ Inf U), supplied in a single use vial (0.5 mL extractable)
- Placebo: 0.9% saline (0.5 mL extractable)

After each vaccination, subjects will remain at the site for a total of 30 (±10) minutes post-vaccination to monitor for the development of any acute reactions, or longer if deemed necessary by the investigator (eg, in case of Grade 3 adverse events).

Criteria for postponement of vaccination at the scheduled time for vaccine administration and contraindications to boost vaccination have been defined and will be applied by the investigator.

SAFETY EVALUATIONS

Safety evaluations will be performed as specified in the Time and Events Schedule.

Safety will be assessed by collection of solicited local and systemic adverse events (reactogenicity), unsolicited adverse events, serious adverse events, and IREs. The subjects will be closely observed by study-site personnel for 30 (±10) minutes after each vaccination to monitor for the development of any acute reactions, or longer if deemed necessary by the investigator (eg, in case of grade 3 adverse events). Any unsolicited, solicited local or systemic adverse events occurring while on site will be documented. Upon discharge from the site, subjects will receive a diary, a thermometer and a ruler to measure body temperature and solicited local reactions. Subjects will be instructed to record solicited local and systemic adverse events in the diary in the evening after each vaccination and then daily for the next 7 days at approximately the same time each day. Diaries should be completed at home by the subject. For those subjects having difficulty completing the diary independently, the sites will make arrangements to have the diary completed according to their local practice. The investigator will document unsolicited adverse events from signing of the ICF onwards until 42 days post-boost, and serious adverse events and IREs from signing of the ICF onwards until the end of the study. The primary endpoints are adverse events, serious adverse events, IREs and solicited local and systemic adverse events. Adverse events that are ongoing at 42 days post-boost vaccination will be followed until resolution or stabilization. Other safety assessments include physical examination, vital signs (blood pressure, pulse/heart rate, body temperature), clinical laboratory and pregnancy testing.

The investigators, together with the sponsor's study responsible physician, will be responsible for the safety monitoring of the study, and will halt vaccination of further subjects in case any of the pre-specified pausing rules have been met or in case of other severe safety concerns.

IMMUNOGENICITY EVALUATIONS

Samples will be collected for immunogenicity assessments as specified in the Time and Events Schedule. Serum samples to assess humoral immune response and peripheral blood mononuclear cell (PBMC) samples to assess cellular immune response will be taken from all subjects. In Part 2, a subset of approximately 40% will also participate in the optional mucosal assessments. Subjects giving informed consent for the study will be informed that their leftover blood and secretion samples will be stored for potential future research.

PHARMACOGENOMIC EVALUATIONS

The human leukocyte antigen (HLA) type will be determined (where local regulations permit) to explore the impact of host genetics on immune responses to the Ad26.ZEBOV/MVA-BN-Filo and MVA-BN-Filo/Ad26.ZEBOV prime-boost regimens. No additional venous blood samples need to be collected for this evaluation, samples collected on the day of the prime vaccination will be used. Subject participation in the pharmacogenomic research is optional and will require additional consent.

STATISTICAL METHODS

An interim analysis will be conducted when all subjects in Part 1 have completed the 21-day post-boost visit, or discontinued earlier. The purpose of this interim analysis is to evaluate the effect of vaccination on the HIV viral load of HIV-infected subjects from a safety perspective. This interim analysis will be reviewed by the Protocol Safety Review Team (PSRT) and forwarded to the IDMC for review. An

additional interim analysis will be done when all subjects in Part 1 have completed the 1-year post-boost visit or discontinued earlier. Additional (ad hoc) interim analyses may be performed during the study for the purpose of informing future vaccine-related decisions in a timely manner. The results will not influence the conduct of the study in terms of early termination or later safety or immunogenicity endpoint assessments unless a significant safety concern has been identified.

For any interim analyses performed in a part before database lock, study-site personnel (except for those with primary responsibility for study vaccine preparation and dispensing), the sponsor (except for specifically designated sponsor personnel who are independent of the study) and subjects will remain blinded to study vaccine allocation.

The final analysis will be performed when all subjects have completed the last study-related visit or discontinued earlier.

Specific details will be provided in the Statistical Analysis Plan (SAP).

Safety Analyses

Safety data will be analyzed descriptively (including 95% confidence intervals, if applicable). For each adverse event, the number and percentage of subjects who experience at least 1 occurrence of the given event will be summarized by group. Summaries, listings, datasets, or subject narratives may be provided, as appropriate, for those subjects who die, who discontinue study vaccine due to an adverse event, or who experience a severe or a serious adverse event. Abnormalities in vital signs and clinical laboratory tests will be tabulated by worst abnormality grade. Physical examination abnormalities will be listed. Exploratory statistical testing of safety data may be performed.

Immunogenicity Analyses

Descriptive statistics (actual values and changes from baseline, including 95% confidence intervals, if applicable) will be calculated for continuous immunologic parameters at each time point. Graphical representations of changes in immunologic parameters will be prepared, as applicable. Frequency tabulations will be calculated for discrete (qualitative) immunologic parameters at each time point. Response patterns over time for the immunologic parameters will be analyzed, taking into account within-subject correlations, to describe differences between the vaccination schedules at 21 days post-boost, 42 days post-boost, 6 months post-boost and 1 year post-boost. Exploratory statistical testing of immunogenicity data may be performed.

TIME AND EVENTS SCHEDULE

Part 1

	Screening Phase[a] (≤ 8 weeks)	Vaccination Phase and Post-boost Follow-up[b,c]									
		D1	D2-D7	D8	D15	D16-D21	D22	D36	D57	D195	D380
**Study Procedures**											
Screening/Administrative											
Informed consent	X										
Consent for apheresis (optional)[d]	X										
Inclusion/exclusion criteria	X										
Medical history and demographics	X										
Prestudy therapies[e]	X										
Test of Understanding (TOU)[f]	X										
Review Screening Data[g]		X									
Randomization		X									
Study Vaccine Administration[h,i]		▼			▲						
Antiretroviral therapy adherence check, if applicable[u]	X	X		X	X		X	X	X	X	X
Safety Assessments											
Electrocardiogram	X[j]										
Urine pregnancy test[k,l]	X	X			X						
Physical examination[m]	X	X		X	X		X	X	X	X	X
Vital signs[n]	X	X		X	X		X	X	X	X	X
Distribution of subject diary[o]		X			X						
Completion of diary at home[p]		X	X		X	X					
Review of diary by site staff				X			X				
Adverse events[q]	Continuous										
Serious adverse events and immediate reportable events[r]	Continuous										
Concomitant medications	X	X	X	X	X	X	X	X	X[s]	X[s]	X[s]
Clinical Lab Assessments											
Chemistry (ALT, creatinine) (4 mL)	X	X		X	X		X				
Hematology[t] (3 mL)	X	X		X	X		X				
Urinalysis	X										

	Screening Phase[a] (≤ 8 weeks)	Vaccination Phase and Post-boost Follow-up[b,c]									
		D1	D2-D7	D8	D15	D16-D21	D22	D36	D57	D195	D380
HIV-1 or HIV-1/2 serology (6 mL)	X										
CD4+ cell count[u] (4 mL)	X	X			X			X	X	X	X
Immunogenicity and Virologic Assessments[v]											
Blood sampling for humoral assays (16 mL)		X[w]			X[w]			X	X	X	X
Blood sampling for cellular assays (80 mL)[d]		X[w]			X[w]			X	X	X	X
HIV viral load quantification and sequencing[u,x] (6 mL at screening, 4 mL at other visits)	X	X			X			X	X	X	X
Approximate daily blood draws[y] (mL)	23	111	0	7	111	0	7	104	104	104	104
Approximate cumulative blood draws[y] (mL)	23	134	134	141	252	252	259	363	467	571	675

▲ *Ad26.ZEBOV 5x10^{10} vp or placebo* ▼ *MVA-BN-Filo 1x10^{8} Inf U or placebo*

NOTE: In case of early withdrawal from the study, early withdrawal assessments (ie, physical examination, vital signs, CD4+ cell count [as applicable], [serious] adverse events and concomitant medications review, immunogenicity, and virologic assessments) should be obtained. Subjects who wish to withdraw consent will be offered an optional visit for safety follow-up (before the formal withdrawal of consent); the subject has the right to refuse.

a. Screening may be split into multiple days or visits. Retesting of values (eg, safety laboratory) that lead to exclusion is allowed only once using an unscheduled visit during the screening phase. The safety laboratory assessments at screening are to be performed within 28 days prior to the prime vaccination and may be repeated if they fall outside this time window. If rescreening is required, all screening procedures (except TOU) should be repeated (unless the TOU has been revised in the meantime, in which case it needs to be taken again) (see Section 9.1.2).
b. In addition to the assessments scheduled for the 42-day post-boost visit, subjects will be instructed to contact the investigator before the next visit if they experience any adverse event or intercurrent illness that they perceive as relevant and/or can be possibly related to study vaccine in their opinion. After unblinding a part (ie, when the last subject in that part completed the 6-month post-boost visit or discontinued earlier), subjects who received placebo will be contacted by the site to communicate that they have completed the study and no further follow-up is required. However, subjects who received placebo and reach the 1-year post-boost visit (Part 1 and Part 2, Group 2: Day 380; Part 2, Group 1: Day 394) prior to unblinding will be required to attend the 1-year post-boost visit.
c. Only subjects who received Ad26.ZEBOV or MVA-BN-Filo will be followed up for collection of serious adverse event and immediate reportable event information post unblinding.
d. Up to 20 subjects (10 HIV-infected, 10 healthy) from Part 1 will be asked to enroll for optional apheresis for collection of peripheral blood mononuclear cell (PBMC) and plasma on the Day 36 and Day 57 visit, given that they have provided specific consent for this procedure. Up to a total of 10 Part 1 subjects (irrespective of HIV status) with robust immune responses post-boost may also be enrolled for an optional apheresis procedure performed after unblinding at a predefined visits or at an unscheduled visit for better characterization of immune responses.

e. Prestudy therapies up to 30 days prior to the start of screening and previous vaccinia/smallpox vaccination at any time prior to study entry must be recorded in the case report form (CRF).
f. After reading but before signing the informed consent form (ICF), the TOU will be conducted (or as required per local regulations and practice). The ICF needs to be signed before any other study-related activities occur.
g. The investigators should ensure that all study enrollment criteria have been met at the end of the screening phase. Minimum criteria for the availability of documentation supporting the eligibility criteria are described in Section 17.4.
h. After each vaccination, subjects will remain at the site for a total of 30 (±10) minutes post-vaccination to monitor for the development of any acute reactions, or longer if deemed necessary by the investigator. Solicited and unsolicited adverse events emerging during the observation period at the site will be recorded in the CRF.
i. Phlebotomy, indicated pregnancy tests, physical examination and measurements of vital signs will occur prior to study vaccine administration.
j. Only for subjects >50 years. A single, 12-lead electrocardiogram (ECG) (supine) after at least 5 minutes rest will be performed and interpreted locally. Additional ECG monitoring may be done at other time points during the study if clinically indicated based on signs and symptoms.
k. Only for women of childbearing potential. A urine pregnancy test should be performed prior to each vaccination administration.
l. Pregnancies will be reported from signing of the ICF until the end of the study.
m. A full physical examination, including height and body weight, will be carried out at screening. A genitourinary examination is not required. At other visits, an abbreviated, symptom-directed examination will be performed as indicated by the investigator.
n. Includes blood pressure, pulse/heart rate (at rest) and body temperature (oral or axillary). The site of body temperature measurement must be captured in the CRF.
o. Diary cards will be used as memory aids to document solicited adverse events by the subject.
p. Subjects will use the subject diary to document solicited local and systemic adverse events (reactogenicity) in the evening after each vaccination and then daily for the next 7 days at approximately the same time each day. Diaries should be completed at home by the subject. For subjects having difficulty completing the diary independently, the sites will make arrangements to have the diary completed according to their local practice.
q. Solicitation of any cardiac sign or symptom must be performed at every visit and if indicated, an ECG and/or troponin I levels and/or echocardiogram should be obtained.
r. For reporting of immediate reportable events, refer to Section 12.3.3.
s. After the 42-day post-boost visit, concomitant therapies should only be recorded if given in conjunction with serious adverse events and immediate reportable events.
t. Complete blood count with differential.
u. Only for HIV-infected subjects.
v. Stored serum or plasma of HIV-infected subjects may be used to assess antiretroviral drug levels.
w. Prior to study vaccine administration.
x. Viral sequencing may be performed if the HIV viral load is sufficiently high.
y. Additional blood may be drawn to confirm and follow-up on adverse events.

Part 2, Groups 1 and 2

	Screening Phase[a] (≤ 8 weeks)	Vaccination Phase and Post-boost Follow-up[b,c]									
Group 1 n=400		**D1**	**D2-D7**	**D8**	**D29**	**D30-D35**	**D36**	**D50**	**D71**	**D209**	**D394**
Group 2 n=100					**D15**	**D16-D21**	**D22**	**D36**	**D57**	**D195**	**D380**
**Study Procedures**											
Screening/Administrative											
Informed consent	X										
Consent for mucosal secretions collections (optional)	X										
Inclusion/exclusion criteria	X										
Medical history and demographics	X										
Prestudy therapies[d]	X										
Test of Understanding (TOU)[e]	X										
Review Screening Data[f]		X									
Randomization		X									
Study Vaccine Administration[g,h]		▲<br>▼			▼<br>▲						
Antiretroviral therapy adherence check, if applicable[t]	X	X		X	X		X	X	X	X	X
Safety Assessments											
Electrocardiogram	X[i]										
Urine pregnancy test[j,k]	X	X			X						
Physical examination[l]	X	X		X	X		X	X	X	X	X
Vital signs[m]	X	X		X	X		X	X	X	X	X
Distribution of subject diary[n]		X			X						
Completion of subject diary at home[o]		X	X		X	X					
Review of diary by site staff				X			X				
Adverse events[p]	Continuous										
Serious adverse events and immediate reportable events[q]	Continuous										
Concomitant medications	X	X	X	X	X	X	X	X	X[r]	X[r]	X[r]
Clinical Lab assessments											
Chemistry (ALT, Creatinine) (4 mL)	X	X		X	X		X				
Hematology[s] (3 mL)	X	X		X	X		X				

	**Screening Phase[a] (≤ 8 weeks)**	**Vaccination Phase and Post-boost Follow-up[b,c]**									
Group 1 n=400		**D1**	**D2-D7**	**D8**	**D29**	**D30-D35**	**D36**	**D50**	**D71**	**D209**	**D394**
Group 2 n=100					**D15**	**D16-D21**	**D22**	**D36**	**D57**	**D195**	**D380**
Urinalysis	X										
HIV-1 or HIV-1/2 serology (6 mL)	X										
CD4+ cell count[t] (4 mL)[u]	X	X			X			X	X	X	X
Immunogenicity and Virologic Assessments[v]											
Mucosal secretions collections (optional)[w]		X[x]			X[x]			X	X	X	X
Blood sampling for humoral assays (16 mL)		X[x]			X[x]			X	X	X	X
Blood sampling for cellular assays (80 mL)		X[x]			X[x]			X	X	X	X
HIV viral load quantification and sequencing[t,y] (6 mL at screening, 4 mL at other visits)	X	X			X			X	X	X	X
Approximate daily blood draws[z](mL)	23	111	0	7	111	0	7	104	104	104	104
Approximate cumulative blood draws[z] (mL)	23	134	134	141	252	252	259	363	467	571	675

▲ *Ad26.ZEBOV 5x10^{10} vp or placebo* ▼ *MVA-BN-Filo 1x10^{8} Inf U or placebo*

NOTE: In case of early withdrawal from the study, early withdrawal assessments (ie, physical examination, vital signs, CD4+ cell count [as applicable], [serious] adverse events and concomitant medications review, immunogenicity, and virologic assessments) should be obtained. Subjects who wish to withdraw consent will be offered an optional visit for safety follow-up (before the formal withdrawal of consent); the subject has the right to refuse.

a. Screening may be split into multiple days or visits. Retesting of values (eg, safety laboratory) that lead to exclusion is allowed only once using an unscheduled visit during the screening phase. The safety laboratory assessments at screening are to be performed within 28 days prior to the prime vaccination and may be repeated if they fall outside this time window. If rescreening is required, all screening procedures (except TOU) should be repeated (unless the TOU has been revised in the meantime, in which case it needs to be taken again) (see Section 9.1.2).

b. In addition to the assessments scheduled for the 42-day post-boost visit, subjects will be instructed to contact the investigator before the next visit if they experience any adverse event or intercurrent illness that they perceive as relevant and/or can be possibly related to study vaccine in their opinion. After unblinding in a part (ie, when the last subject in that part completed the 6-month post-boost visit or discontinued earlier), subjects who received placebo will be contacted by the site to communicate that they have completed the study and no further follow-up is required. However, subjects who received placebo and reach the 1-year post-boost visit (Part 1 and Part 2, Group 2: Day 380; Part 2, Group 1: Day 394) prior to unblinding will be required to attend the 1-year post-boost visit.

c. Only subjects who received Ad26.ZEBOV or MVA-BN-Filowill be followed up for collection of serious adverse event and immediate reportable event information post unblinding.
d. Prestudy therapies up to 30 days prior to the start of screening and previous vaccinia/smallpox vaccination at any time prior to study entry must be recorded in the CRF.
e. After reading but before signing the ICF, the TOU will be conducted (or as required per local regulations and practice). The ICF needs to be signed before any other study-related activities occur.
f. The investigators should ensure that all study enrollment criteria have been met at the end of the screening phase. Minimum criteria for the availability of documentation supporting the eligibility criteria are described in Section 17.4.
g. After each vaccination, subjects will remain at the site for a total of 30 (±10) minutes post-vaccination to monitor for the development of any acute reactions, or longer if deemed necessary by the investigator. Solicited and unsolicited adverse events emerging during the observation period at the site will be recorded in the CRF.
h. Phlebotomy, indicated pregnancy test, physical examination and measurements of vital signs will occur prior to study vaccine administration.
i. Only for subjects >50 years. A single, 12-lead ECG (supine) after at least 5 minutes rest will be performed and interpreted locally. Additional ECG monitoring may be done at other time points during the study if clinically indicated based on signs and symptoms.
j. Only for women of childbearing potential. A urine pregnancy test should be performed prior to each vaccination administration.
k. Pregnancies will be reported from signing of the ICF until the end of the study.
l. A full physical examination, including height and body weight, will be carried out at screening. A genitourinary examination is not required. At other visits, an abbreviated, symptom-directed examination will be performed as indicated by the investigator.
m. Includes blood pressure, pulse/heart rate (at rest) and body temperature (oral or axillary). The site of body temperature measurement must be captured in the CRF.
n. Diary cards will be used as memory aids to document solicited adverse events by the subject.
o. Subjects will use the subject diary to document solicited local and systemic adverse events (reactogenicity) in the evening after each vaccination and then daily for the next 7 days at approximately the same time each day. Diaries should be completed at home by the subject. For subjects having difficulty completing the diary independently, the sites will make arrangements to have the diary completed according to their local practice.
p. Solicitation of any cardiac sign or symptom must be performed at every visit and if indicated, an ECG and/or troponin I levels and/or echocardiogram should be obtained.
q. For reporting of immediate reportable events, refer to Section 12.3.3.
r. After the 42-day post-boost visit, concomitant therapies should only be recorded if given in conjunction with serious adverse events and immediate reportable events.
s. Complete blood count with differential.
t. Only for HIV-infected subjects.
u. The site's Standard Operating Procedure will be followed with regard to the blood volumes for CD4+ cell count.
v. Stored serum or plasma of HIV-infected subjects may be used to assess antiretroviral drug levels.
w. Includes collection of genital, rectal and oral secretions.
x. Prior to study vaccine administration.
y. Viral sequencing may be performed if the HIV viral load is sufficiently high.
z. Additional blood may be drawn to confirm and follow-up on adverse events.

ABBREVIATIONS

Adxx	adenovirus serotype xx (vector)
Ad26.ZEBOV	adenovirus serotype 26 expressing the Ebola virus Mayinga glycoprotein
AIDS	acquired immunodeficiency syndrome
ART	antiretroviral therapy
β-hCG	β-human chorionic gonadotropin
BMI	body mass index
BN	Bavarian Nordic
CBER	Center for Biologics Evaluation and Research
(e)CRF	(electronic) case report form
DMID	Division of Microbiology and Infectious Diseases
DNA	deoxyribonucleic acid
DoD	Department of Defense
EBOV	Ebola virus
eDC	electronic data capture
ECG	electrocardiogram
EDTA	ethylenediaminetetraacetic acid
ELISA	enzyme-linked immunosorbent assay
ELISpot	enzyme-linked immunospot
EU	European Union
FDA	Food and Drug Administration
GCP	Good Clinical Practice
GMP	Good Manufacturing Practice
GP	glycoprotein
HAART	highly active antiretroviral therapy
HIV	human immunodeficiency virus
HRPO	Human Research Protection Office
HSPB	Human Subjects Protection Branch
ICF	informed consent form
ICH	International Council for Harmonisation
ICS	intracellular cytokine staining
IDMC	Independent Data Monitoring Committee
IEC	Independent Ethics Committee
IFN-γ	interferon-gamma
Ig (X)	immunoglobulin (X)
IL	interleukin
IM	intramuscular(ly)
Inf U	infectious units
IRB	Institutional Review Board
IRE	immediate reportable event
IWRS	interactive web response system
kb	kilobase
MARV	Marburg virus
MedDRA	Medical Dictionary for Regulatory Activities
MVA	Modified Vaccinia Ankara
MVA-BN-Filo	Modified Vaccinia Ankara Bavarian Nordic vector expressing multiple filovirus proteins
NHP	nonhuman primate
NP	nucleoprotein
PBMC	peripheral blood mononuclear cell
PCR	polymerase chain reaction
PI	Principal investigator
PQC	Product Quality Complaint
PSRT	Protocol Safety Review Team
RNA	ribonucleic acid
SAP	Statistical Analysis Plan
SUDV	Sudan virus

SUSAR	suspected unexpected serious adverse reaction
TAFV	Tai Forest virus
$TCID_{50}$	50% tissue culture infective dose
THAM	tris (hydroxymethyl)-amino methane
TNF-α	tumor necrosis factor-α
TOU	test of understanding
US	United States
USAMRMC	United States Army Medical Research and Materiel Command
VISP	vaccine-induced seropositivity
VNA	virus neutralization assay
vp	viral particles
WHO	World Health Organization
WRAIR	Walter Reed Army Institute of Research

DEFINITIONS OF TERMS

Study vaccine	Ad26.ZEBOV, MVA-BN-Filo or placebo.
Blinded study vaccine administrator	A blinded trained study nurse, medical doctor, or otherwise qualified health care provider responsible for administration of study vaccine.
Independent study vaccine monitor	An unblinded study vaccine monitor assigned to the study who is responsible for the unblinded interface between the sponsor and the investigational site pharmacy.
Solicited adverse events (reactogenicity)	Local and systemic adverse events that are common and known to occur after vaccination and that are usually collected in a standard, systematic format in vaccine clinical studies. For the list of solicited adverse events in this study, see Section 9.3. For the purpose of vaccine clinical studies, all other adverse events are considered unsolicited; however, this definition should be distinguished from definitions based on pharmacovigilance guidelines.

1. INTRODUCTION

Janssen Vaccines & Prevention B.V. (formerly known as Crucell Holland B.V., hereafter referred to as the sponsor), in collaboration with Bavarian Nordic (BN), and in conjunction with Walter Reed Army Institute of Research (WRAIR), is investigating the potential of a prophylactic Ebola vaccine regimen comprised of the following 2 candidate Ebola vaccines:

Ad26.ZEBOV is a monovalent vaccine expressing the full length Ebola virus (EBOV, formerly known as *Zaire ebolavirus*) Mayinga glycoprotein (GP), and is produced in the human cell line PER.C6®.

MVA-mBN226B, further referred to as Modified Vaccinia Ankara (MVA)-BN®-Filo, is a multivalent vaccine expressing the Sudan virus (SUDV) GP, the EBOV GP, the Marburg virus (MARV) Musoke GP, and the Tai Forest virus (TAFV, formerly known as *Côte d'Ivoire ebolavirus*) nucleoprotein (NP), and is produced in chicken embryo fibroblast cells. The EBOV GP expressed by MVA-BN-Filo has 100% homology to the one expressed by Ad26.ZEBOV.

For the most up-to-date nonclinical and clinical information regarding Ad26.ZEBOV and MVA-BN-Filo, refer to the latest versions of the Investigator's Brochures and Addenda (if applicable).[11,12] A brief summary of the nonclinical and clinical information is provided below.

The term "sponsor" used throughout this document refers to the entities listed in the Contact Information page(s), which will be provided as a separate document.

1.1. Background

Ebola viruses belong to the Filoviridae family and cause Ebola virus disease, which can induce severe hemorrhagic fever in humans and nonhuman primates (NHPs). Case fatality rates in Ebola disease range from 25% to 90% (average: 50%), according to the World Health Organization (WHO).[22] These viruses are highly prioritized by the United States (US) Government, who has defined them as 'Category A' agents, due to the high mortality rate of infected individuals and the potential use as a bioweapon.

Currently, no licensed vaccine, treatment or cure exists for this disease.

Filoviruses are named for their long, filamentous shape. Within this filamentous virus, a single 19-kilobase (kb) negative-sense ribonucleic acid (RNA) genome encodes 7 proteins: the GP, the polymerase, the NP, the secondary matrix protein, the transcriptional activator, the polymerase cofactor, and the matrix protein. The virion surface is covered by homotrimers of the viral GP, which is believed to be the sole host attachment factor for filoviruses. Following cell entry, the viruses replicate their genomes and viral proteins in the cytoplasm using an RNA-dependent RNA polymerase, which is carried into the cell together with the virus.[9]

Nonclinical Studies

Immunogenicity and Efficacy

Immunogenicity and efficacy of the vaccine combination Ad26.ZEBOV and MVA-BN-Filo was evaluated in an NHP model (ie, Cynomolgus macaques, *Macaca fascicularis*). The combination was assessed in a multivalent filovirus setting in a small number (2 per regimen) of animals and the study included heterologous prime-boost regimens of adenovirus serotype 26 (Ad26), Ad35 and MVA-BN-Filo vectors expressing different Ebola and Marburg proteins. Full protection from Ebola virus disease and death after wild-type EBOV Kikwit 1995 challenge was obtained with all heterologous regimens, including the Ad26 and MVA vaccine regimen. All heterologous prime-boost regimens induced comparable immune responses against the EBOV Mayinga GP. Independently of the vaccine regimen, a strong boost effect was seen after heterologous prime-boost immunization. Two additional studies involving more animals are ongoing, to strengthen the robustness of the nonclinical efficacy data, and also to optimize the prime-boost schedule so as to obtain induction of protective immunity as quickly as possible, to specifically respond to the Ebola virus disease outbreak in West Africa.

Toxicology

A repeated-dose toxicity study in rabbits was performed with prime-boost combinations of Ad26.ZEBOV and MVA-BN-Filo. The different dose regimens were well tolerated when administered twice by intramuscular (IM) injection to New Zealand White rabbits with a 14-day interval period. Additionally, the objective was to assess the persistence, reversibility or delayed onset of any effects after a 14-day treatment-free period. In the heterologous prime-boost regimen, either vector or both were used to prime a filovirus-specific immune response and the other/same vector or both were used to boost the immune response 2 weeks later. All vaccine dosing regimens resulted in detectable EBOV GP-specific antibody titers. No significant toxicological effects (no adverse effects) were observed. The immune response was associated with transient increases in fibrinogen, C-reactive protein, globulin, decreases in hematocrit and hemoglobin, and microscopic findings in draining iliac lymph nodes, spleen and at the injection sites. The findings were noted to be recovering over a 2-week treatment-free period and were considered to reflect a physiological response associated with vaccination. There were no effects noted that were considered to be adverse.

Biodistribution

Single-dose biodistribution studies in rabbits were performed using the MVA-BN vector or the Ad26 vector in combination with another insert (Ad26.ENVA.01: an experimental, prophylactic Ad26 vector expressing the human immunodeficiency virus [HIV] type 1, Clade A envelope protein). MVA-BN distributed to the skin, muscle, blood, spleen, lung, liver, and pooled lymph nodes and was rapidly cleared (within 48 hours following vaccination). Ad26.ENVA.01 was primarily localized in the injection site muscle, the regional lymph nodes and the spleen. Three months after the single IM injection of Ad26.ENVA.01, the vaccine was cleared from most of the examined tissues. As biodistribution is dependent on the vector platform (MVA or Ad26) and not on the insert, it can be assumed that recombinant MVA-BN-Filo or Ad26.ZEBOV is distributed in the same way as the MVA-BN vector or Ad26.ENVA.01 vector, respectively.

Clinical Studies

To date, no clinical studies have been completed with Ad26.ZEBOV or MVA-BN-Filo. However, 4 Phase 1 studies (VAC52150EBL1001, VAC52150EBL1002, VAC52150EBL1003 and VAC52150EBL1004), 2 Phase 2 studies (VAC52150EBL2001 and VAC52150EBL2002) and 1 Phase 3 study (VAC52150EBL3001) are ongoing where monovalent Ad26.ZEBOV vaccine and multivalent MVA-BN-Filo vaccine are combined in homologous or heterologous prime-boost regimens in which each vector is used to prime a filovirus-specific immune response followed by a boost immunization with the same or the other vector 2 to 12 weeks later. Most subjects in the 4 Phase 1 studies have been vaccinated and are in follow-up. Two additional Phase 1 studies investigating MVA-BN-Filo are also ongoing (EBL01 and CVD-Mali Ebola Vaccine #1000). Refer to the latest versions of the Ad26.ZEBOV and MVA-BN-Filo Investigator's Brochures and Addenda (if applicable) for more details.[11,12]

Limited data from the ongoing Phase 1 studies with Ad26.ZEBOV and MVA-BN-Filo are available.

VAC52150EBL1001, a first-in-human study, is a randomized, placebo-controlled, observer-blind study in healthy adults evaluating the safety, tolerability and immunogenicity of 4 regimens using MVA-BN-Filo at a dose of $1x10^8$ 50% tissue culture infective dose ($TCID_{50}$) and Ad26.ZEBOV at a dose of $5x10^{10}$ viral particles (vp): 2 regimens with MVA-BN-Filo as prime and Ad26.ZEBOV as boost at a 28- or 56-day interval, and 2 regimens with Ad26.ZEBOV as prime and MVA-BN-Filo as boost at a 28- or 56-day interval. A fifth regimen, with Ad26.ZEBOV at a dose of $5x10^{10}$ vp as prime, and MVA-BN-Filo at a dose of $1x10^8$ $TCID_{50}$ at a 14-day interval was evaluated in the open-label, uncontrolled, substudy in healthy adults. VAC52150EBL1001 has enrolled 87 subjects for which 7-day post-prime safety data, blinded on a treatment group level, on 72 subjects (36 per treatment group) are available. Most of the adverse events reported were grade 1 or grade 2 in severity. Local injection site reactions were reported in 18 (50%) MVA/placebo subjects (all grade 1) and 28 (78%) Ad26/placebo subjects (grade 1 [22], grade 2 [5], grade 3 [1]). The most frequent local reaction was injection site pain, in 17 (47%) MVA/placebo subjects and 28 (78%) Ad26/placebo subjects, with 1 grade 3 case occurring in the Ad26/placebo group. Solicited systemic reactions were reported in 25 (69%) MVA/placebo subjects (grade 1 [24] and grade 2 [1]) and 31 (86%) Ad26/placebo subjects (grade 1 [22], grade 2 [7], grade 3 [1], unknown [1]). The most frequent systemic reactions were fatigue (50% overall), followed by headache (46%) and myalgia (35%). One Ad26/placebo subject experienced 3 grade 3 solicited systemic reactions (headache, myalgia and nausea). None of the subjects reported fever; however, 2 subjects had 1 temperature measurement missing. The most frequent unsolicited adverse events were decreased neutrophils, in 3 (8%) MVA/placebo subjects and 6 (17%) Ad26/placebo subjects, followed by activated partial thromboplastin time prolongation and hypokalemia, in 3 (8%) MVA/placebo subjects and 5 (14%) Ad26/placebo subjects each. Two subjects in the open-label substudy who experienced grade 3 neutropenia did not receive the boost vaccination due to contraindications to the boost (as specified in the protocol), but continued scheduled assessments as planned. All of these events were transient in nature and resolved without intervention. No deaths or serious adverse events related to the vaccine were reported during the blinded portion of the study.

VAC52150EBL1002 is a randomized, placebo-controlled, observer-blind study in healthy adults evaluating the safety, tolerability and immunogenicity of heterologous and homologous prime-boost regimens using MVA-BN-Filo and Ad26.ZEBOV administered in different doses, sequences and schedules: MVA-BN-Filo ($1x10^8$ $TCID_{50}$) as prime followed by a Ad26.ZEBOV ($5x10^{10}$ vp) as boost at 14, 28, or 56 days after prime; Ad26.ZEBOV ($5x10^{10}$ vp) as prime followed by MVA-BN-Filo ($1x10^8$ $TCID_{50}$) as boost at 28 days after prime; Ad26.ZEBOV ($5x10^{10}$ vp) as prime followed by a high dose of MVA-BN-Filo ($4.4x10^8$ $TCID_{50}$) as boost at 14 days after the prime; a high dose of Ad26.ZEBOV ($1x10^{11}$ vp) as prime followed by a high dose of MVA-BN-Filo ($4.4x10^8$ $TCID_{50}$) as boost at 28 days after the prime. VAC52150EBL1002 completed enrollment of 92 subjects; the blinded phase of the study is ongoing. No serious adverse events related to study vaccine have been reported and no safety issues have been identified to date.

VAC52150EBL1003 and VAC52150EBL1004 are randomized, placebo-controlled, observer-blind studies in healthy adults evaluating the safety, tolerability and immunogenicity of 4 regimens using MVA-BN-Filo at a dose of $1x10^8$ $TCID_{50}$ and Ad26.ZEBOV at a dose of $5x10^{10}$ vp: 2 regimens had MVA-BN-Filo as prime and Ad26.ZEBOV as boost at a 28- or 56-day interval, and 2 regimens had Ad26.ZEBOV as prime and MVA-BN-Filo as boost at a 28- or 56-day interval. The blinded phase of studies VAC52150EBL1003 and 1004 are ongoing with enrollment complete in 1003. The blinded safety profile is similar to that reported in VAC52150EBL1001 and 1002 studies with so far, fewer reports of solicited local and systemic adverse events. No vaccine-related serious adverse events have been reported in either study.

Safety data generated with the 2 vectors containing different inserts are provided below:

Safety Profile of Ad26.ZEBOV

Ad26.ZEBOV is a monovalent recombinant, replication-incompetent Ad26-based vector. Only limited clinical data are available for Ad26.ZEBOV. However, adenovirus vaccine programs with other gene inserts revealed no significant safety issues. The data described below are based on the evaluation of the prototype vaccine Ad26.ENVA.01, which expresses the HIV envelope gene.[13]

Three randomized, placebo-controlled, Phase 1 studies (IPCAVD-001, IPCAVD-003, IPCAVD-004) have evaluated the safety and immunogenicity of the prototype vaccine Ad26.ENVA.01. This prototype vaccine has been administered to more than 200 healthy, HIV-negative subjects between the ages of 18 and 50 years in the US and Africa.[13,14,15]

- In the dose-escalation study IPCAVD-001 (n=60), 2 or 3 IM doses of Ad26.ENVA.01 ($1x10^9$, $1x10^{10}$, $5x10^{10}$, $1x10^{11}$ vp) were given to Ad26 seronegative subjects. There were no deaths or vaccine-related serious adverse events. Ad26.ENVA.01 was generally well tolerated at all 4 dose levels with minimal reactogenicity observed in the $1x10^9$ and $1x10^{10}$ vp dose groups. Moderate to severe malaise, myalgia, fatigue and chills occurred in the majority of subjects 12 to 18 hours after the first dose of $1x10^{11}$ vp, but were resolved within 24 to 36 hours and were not seen after the second injection at this dose level. Two subjects in the $1x10^{11}$ vp dose group chose not to have the second injection, however, 1 of them decided to have the 6-month injection. Envelope-specific humoral and cell-mediated immune responses were induced at all 4 dose levels of vaccine.[2,4]
- In the single-dose study IPCAVD-003 (n=24), an IM dose of Ad26.ENVA.01 ($5x10^{10}$ vp) or placebo was given to subjects, who were stratified according to baseline Ad26 immune status, to evaluate the safety, mucosal immunogenicity and innate immune responses. Local reactogenicity comprised moderate injection site pain/tenderness and/or moderate to severe erythema which resolved within 3 days of vaccination. Transient systemic reactogenicity comprised headache, chills, joint pain, myalgia, malaise/fatigue, and fever. No deaths or vaccine-related serious adverse events were observed. Vaccination elicited both systemic and mucosal envelope-specific humoral and cellular immune responses. No increased activated total or vector-specific mucosal CD4+ T-lymphocytes following vaccination were detected in the colorectal mucosa, indicating that vaccination with Ad26 did not increase mucosal inflammation.[1]
- In study IPCAVD-004 (n=217), the safety and immunogenicity of IM doses of Ad26.ENVA.01 and Ad35.ENV (an Ad35 vector expressing an HIV envelope GP used in that study at a dose of $5x10^{10}$ vp), given in heterologous and homologous prime-boost regimens at 3- versus 6-month intervals, was evaluated. There were 452 adverse events reported by 84 of 176 Ad26-vaccine recipients (47.7%), the majority being mild (75.5%) in severity. The proportion of subjects with moderate or severe symptoms was not statistically significantly different between vaccine and placebo. There were 3 serious adverse events: 2 serious adverse events in placebo recipients (grade 3 peritonsillar abscess and grade 4 migraine headache, both resolved with no residual effects) and 1 serious adverse event in an Ad35/Ad26 vaccine recipient (grade 4 acute myelogenous leukemia, resolved with sequelae). No deaths or vaccine-related serious adverse events were reported. Overall, 97% to 100% of subjects developed anti-envelope binding antibodies (enzyme-linked immunosorbent assay [ELISA]) after a second dose, with heterologous and homologous regimens being comparable. Immune responses in groups who received 3- and 6-month schedules were comparable. Four weeks post-vaccination, interferon (IFN)-γ enzyme-linked immunospot (ELISpot) assay showed response rates between 44% and 100%. The heterologous and homologous regimens were comparable. There was induction of Ad26-neutralizing antibodies in the majority of vaccine recipients after 2 immunizations with Ad26.ENVA.01.[16]

In addition, the sponsor performed a Phase 1/2a double-blind, randomized, placebo-controlled, dose-escalation study (MAL-V-A001) to evaluate the safety, tolerability and immunogenicity of 2 dose levels ($1x10^{10}$ and $5x10^{10}$ vp) of Ad35.CS.01/Ad26.CS.01 (both expressing the malaria *Plasmodium falciparum* circumsporozoite antigen) prime-boost regimens in healthy subjects. The dose-escalation phase was followed by an evaluation of efficacy of the higher dose level in an experimental malaria challenge. A total of 42 subjects were enrolled and were vaccinated. The

analysis of adverse events did not show any consistent pattern suggestive of an association of Ad35.CS.01 or Ad26.CS.01 with specific adverse events. There were no serious adverse events reported during the study. No subject discontinued during a study phase (vaccination or challenge) due to adverse events. One subject in the high-dose group completed the vaccination phase and the final safety follow-up visit but did not take part in any challenge phase activities because of ongoing dyspnea. The most common related adverse events after each vaccination were injection site pain, malaise, headache, myalgia and chills. The incidence of vaccine-related adverse events was generally higher in the high-dose group than in the low-dose group. In general, incidence of malaise, headache, and myalgia were higher after the third dose (Ad26) than after the first or second doses (Ad35). Injection site pain was more commonly reported in the low and high-dose groups than by placebo subjects. There were no clinically significant changes in laboratory test parameters or vital signs data.[5]

Based on the previous clinical experience of Ad26 vector with different inserts, there has been no impact of Ad26 seropositivity on subjects' safety and only limited impact on immunogenicity results. Therefore, there are no safety concerns with regard to the inclusion of Ad26 seropositive subjects in the study, and the study subjects will not be screened for Ad26 seropositivity as part of the study eligibility criteria. The purpose of the Ad26 seropositivity assessment at baseline is to evaluate its impact, if any, on vaccine immunogenicity.

Safety Profile of MVA-BN

MVA-BN is a further attenuated version of the MVA virus, which in itself is a highly attenuated strain of the poxvirus Chorioallantois Vaccinia Virus Ankara. MVA-BN induces strong cellular activity as well as a humoral (antibody) immune response and has demonstrated an ability to stimulate a response even in individuals with pre-existing immunity against Vaccinia. One of the advantages of MVA-BN is the virus' inability to replicate in a vaccinated individual. The replication cycle is blocked at a very late stage, which ensures that new viruses are not generated and released. This means that the virus cannot spread in the vaccinated person and none of the serious side effects normally associated with replicating Vaccinia viruses have been seen with MVA-BN.

MVA-BN (MVA-BN®, trade name IMVAMUNE® outside the European Union [EU], invented name IMVANEX® in the EU) has received marketing authorization in the EU for active immunization against smallpox in adults and in Canada for persons 18 years of age and older who have a contraindication to the first or second generation smallpox vaccines including individuals with immune deficiencies and skin disorders.[10] A Phase 3 clinical study (POX-MVA-013) has been completed (ClinicalTrials.gov Identifier: NCT01144637)[6]. Results of completed and ongoing clinical studies of MVA-BN-based vaccines in more than 8,100 individuals, including elderly subjects (up to age of 80 years), children and subjects in whom replicating vaccines are contraindicated (eg, individuals with HIV infection or diagnosed with atopic dermatitis), have shown that the platform displays high immunogenicity and a favorable safety profile.[17] Across all clinical studies, no trends for unexpected or serious adverse reactions due to the product were detected.

Safety information was pooled from the first 2 studies of MVA-BN-Filo (VAC52150EBL1001 and VAC52150EBL1002). In general, MVA-BN-Filo has been shown to be well tolerated.[12]

Three fifths of the subjects reported at least one local site reaction (injection site pain, tenderness, warmth, redness, swelling and/or itching) following administration of MVA-BN-Filo; mostly of mild severity. The most common reported local site reaction was pain at the injection site. All the local reactions resolved to normal without any lasting effects.

At least one general symptom was reported in two fifths of the subjects following MVA-BN-Filo administration. The most common general symptoms were fatigue, headache, myalgia (muscle pain) and nausea. All general symptoms were transient and resolved without lasting effects.

Changes in laboratory tests were reported following MVA-BN-Filo administration which included hypokalemia and decreased numbers of neutrophils (neutropenia). Both changes in laboratory tests were seen in similar numbers of participants following MVA-BN-Filo and the dummy (placebo) vaccine. Less frequently, events of decreased hemoglobin levels were reported. The changes in laboratory tests were not associated with any complaints or symptoms.

Extensive nonclinical studies support the safety profile of the MVA-BN strain.[19,20]

Relevant Safety Information from Ongoing VAC52150 Studies

One subject in the study VAC52150EBL2001 experienced a serious and very rare condition called “Miller Fisher syndrome”. This condition consists of double vision, pain on moving the eye, and difficulty with balance while walking. Miller Fisher syndrome most commonly occurs following a recent infection. The subject experienced these symptoms about a week after suffering from a common cold and fever. The event happened about a month after boost vaccination with either MVA-BN-Filo or placebo. This subject had to go to the hospital for treatment and has recovered. After an extensive investigation, the event has been considered to be doubtfully related to vaccine and most likely related to the previous common cold.

In the ongoing clinical studies with more than 2,000 participants, there have been a few reports of mild to moderate tingling especially in the hands and feet or a sensation of mild to moderate muscle weakness in subjects vaccinated with Ad26.ZEBOV or placebo. These symptoms have been observed to last no more than 24 to 48 hours in the majority of cases but can last for several weeks before going away on their own. These types of symptoms have also been reported following administration of other licensed vaccines and following acute viral infections of various types. One serious case of probable peripheral sensory neuropathy of moderate severity has occurred and has been ongoing for several months, interfering with some of the subject’s daily activities.

1.2. Benefit/Risk Section

1.2.1. Known Benefits

The clinical benefit of prime-boost combinations of Ad26.ZEBOV and MVA-BN-Filo is to be established.

1.2.2. Potential Benefits

Subjects may benefit from clinical testing and physical examination; others may benefit from the knowledge that they may aid in the development of an Ebola vaccine. There is no direct individual benefit from vaccination for the subjects at the current development stage.

1.2.3. Known Risks

To date, there are only limited data from the Phase 1 studies with Ad26.ZEBOV and MVA-BN-Filo available. However, Ad26- and Ad35-based vaccines with other gene inserts have been administered to a limited number of human volunteers in clinical studies. These other vaccines mainly elicited some solicited local and systemic reactions, as expected with injectable vaccines, and no serious safety concerns in study participants. MVA-BN-based vaccines have been administered to more than 8,100 individuals without unexpected or serious adverse reactions reported. For details, see the safety data presented in Section 1.1.

1.2.4. Potential Risks

The following potential risks for Ad26.ZEBOV and MVA-BN-Filo will be monitored during the study:

Risks Related to Vaccines

Subjects may exhibit general signs and symptoms associated with administration of a vaccine, or a placebo vaccination, including nausea/vomiting, headache, myalgia, arthralgia, fever, fatigue/malaise and chills. In addition, subjects may experience local (injection site) reactions such as pain/tenderness, erythema, induration/swelling and itching at the injection site. These events will be monitored, but are generally short-term and do not require treatment.

Subjects may have an allergic reaction to the vaccination. An allergic reaction may cause a rash, hives or even difficulty breathing (anaphylaxis). Severe reactions are rare. Medications must be available in the clinic to treat serious allergic reactions.

The risks related to vaccine-induced seropositivity (VISP) are discussed in Section 9.7.

Risk of Myo/Pericarditis

While replicating smallpox vaccines have been associated with an increased risk to develop myo/pericarditis,[18] this has not been observed with MVA-BN and is not expected with this highly attenuated, non-replicating vaccine. Based on observations with first- and second-generation replication-competent smallpox vaccines, particular attention has been placed on the monitoring for cardiac signs and symptoms in all clinical studies using MVA-BN. Despite the close cardiac monitoring, no event indicating a symptomatic case of myo/pericarditis has been

observed in any completed MVA-BN study. There has been 1 case of chest pain that might be indicative of pericarditis (consisting of chest pain only with no other cardiac findings suggestive of pericarditis) with previous MVA use although this diagnosis was not finally confirmed and the subject fully recovered. In a review of prospective surveillances for cardiac adverse events in 6 different clinical studies in 382 subjects receiving MVA vaccines, only 1 subject (0.3%) met the criteria for vaccine-induced myocarditis and eventually the subject was found to suffer from exercise-induced palpitations. Self-limited mild elevations in troponin I were recorded in 3 subjects (0.8%) without evidence of myo/pericarditis.[7] Based on the current exposure data in more than 8,100 subjects vaccinated with MVA-BN and other MVA-BN recombinant products, the safety profile of MVA-BN has shown to be comparable with other licensed, live attenuated vaccines.

Pregnancy and Birth Control

The effect of the study vaccines on a fetus, nursing baby, or semen is unknown, so female subjects of childbearing potential, and male subjects having sexual intercourse with females, must agree to practice adequate birth control measures for sexual intercourse from at least 28 days before the prime vaccination (or prior to prime vaccination for men) until at least 3 months after the boost vaccination (see Section 4.3). Women who are pregnant or breast-feeding, or are planning to become pregnant while enrolled in the study until 3 months after the boost vaccination, will be excluded from enrollment into the study.

Risks from Blood Draws

Blood draws may cause pain/tenderness, bruising, bleeding, fainting, and, rarely, infection at the site where the blood is taken.

Unknown Risks

There are no clinical data on the use of either vaccine (Ad26.ZEBOV or MVA-BN-Filo) in:

- Pediatric subjects (<18 years)
- Pregnant or nursing women
- Adults >50 years
- Immunocompromised subjects (including those with HIV infection)

There may be other serious risks that are not known.

1.2.5. Overall Benefit/Risk Assessment

Based on the available data and proposed safety measures, the overall benefit/risk assessment for this clinical study is considered acceptable for the following reasons:

- Preliminary safety data from the ongoing Phase 1 studies and safety data generated with the 2 vaccines with different inserts revealed no significant safety issues (see Sections 1.1 and 1.2.3). Further experience from Ad26.ZEBOV or MVA-BN-Filo will be gained from currently ongoing clinical studies.

- Only subjects who meet all inclusion criteria and none of the exclusion criteria (specified in Section 4) will be allowed to participate in this study. The selection criteria include adequate provisions to minimize the risk and protect the well-being of subjects in the study.
- Safety will be closely monitored throughout the study:
  - After each vaccination, subjects will remain at the site for a total of 30 (±10) minutes post-vaccination to monitor the development of any acute reactions, or longer if deemed necessary by the investigator (eg, in case of grade 3 adverse events). Refer to Section 6.1 for more information on emergency care. Any unsolicited, solicited local or systemic adverse events occurring while on site will be documented. Subjects will use a diary memory aid to document solicited local and systemic adverse events in the evening after each vaccination and then daily for the next 7 days at approximately the same time each day. Diaries should be completed at home by the subject. For subjects having difficulty completing the diary independently, the sites will make arrangements to have the diary completed according to their local practice.
  - The investigator or the designee will document unsolicited adverse events from signing of the informed consent form (ICF) onwards until 42 days post-boost, and serious adverse events and immediate reportable events (IREs) from signing of the ICF onwards until the end of the study.
  - Safety measures, including an electrocardiogram (ECG, performed at screening in subjects >50 years and at other time points during the study if clinically indicated based on signs and symptoms), physical examinations, vital sign measurements, clinical laboratory and pregnancy testing, will be performed at scheduled visits during the study, which lasts up to 1 year after the boost vaccination in subjects who received Ad26.ZEBOV or MVA-BN-Filo.
  - Any clinically significant abnormalities (including those persisting at the end of the study/early withdrawal) will be followed by the investigator until resolution or until a clinically stable endpoint is reached.
- Several safety measures are included in this protocol to minimize the potential risk to subjects, including the following:
  - The neuroinflammatory disorders listed in Section 12.1.1 should be categorized as IREs and should be reported to the sponsor as described in Section 12.3.3.
  - There are pre-specified pausing rules that would result in pausing of further vaccination if predefined conditions occur, preventing exposure of new subjects to study vaccine until an Independent Data Monitoring Committee (IDMC) reviews all safety data (see Section 9.3.2).
  - Subjects will discontinue study vaccine for the reasons included in Section 10.2.
  - If acute illness (excluding minor illnesses such as diarrhea or mild upper respiratory tract infection) or fever (body temperature ≥38.0 °C) occur at the scheduled time for vaccination, the subject may be vaccinated up to 10 days beyond the window allowed for the scheduled vaccination, or be withdrawn from that vaccination at the discretion of the investigator and after consultation with the sponsor (see Section 6.2).
  - Contraindications to boost vaccinations are included in Section 6.3.

– If a subject withdraws from the study (withdrawal of consent), he/she maintains the option to participate in the safety follow-up (see Section 10.2).

1.3. Overall Rationale for the Study

In nonclinical studies in the Cynomolgus macaque model, heterologous prime-boost regimens of a multivalent mixture of Ad26 vectors (each expressing EBOV Mayinga, SUDV or MARV GP) and MVA-BN-Filo provided complete protection against the highly pathogenic wild-type EBOV Kikwit 1995 variant (report pending). Further nonclinical studies are ongoing to evaluate the protection of the multivalent vaccine regimen in additional animals and to assess the protective efficacy of a combination regimen of Ad26.ZEBOV and MVA-BN-Filo (either a simultaneous administration or as prime-boost regimen).

In humans, both Ad26- and MVA-based vaccines containing various antigenic inserts have been shown to be safe and immunogenic (see Section 1.1). To date, more than 230 subjects have received the sponsor's Ad26-based vaccines in completed clinical studies (based on the adenoviral vaccine safety database report [dated 20 March 2015]). Up to 28 October 2015, 227 subjects received Ad26.ZEBOV in ongoing studies. The MVA-BN platform is the basis of the non-replicating smallpox vaccine registered in Canada and Europe, and has been safely used in more than 7,600 humans.[17] Although routinely used by the subcutaneous route, MVA-BN at a dose of $1x10^8$ $TCID_{50}$ has been demonstrated to be as safe and immunogenic when used by the IM route.[8,21] The IM route has been chosen for the present study.

The unprecedented size and scale of the ongoing Ebola disease outbreak that started in December 2013 in Guinea and subsequently spread to Sierra Leone, Nigeria and Liberia, led to the outbreak being declared a public health emergency of international concern in August 2014 by the WHO. This study is one of a series of studies to evaluate the heterologous combination of Ad26.ZEBOV and MVA-BN-Filo as a possible vaccine regimen to prevent Ebola disease. It will test schedules that are being evaluated in ongoing NHP challenge and Phase 1 clinical studies.

In this Phase 2 study, the sponsor's Ad26 vector expressing the EBOV Mayinga GP (Ad26.ZEBOV) and the MVA-BN vector with EBOV, SUDV and MARV GP inserts and TAFV NP insert (MVA-BN-Filo) will be evaluated as a heterologous prime-boost regimen. The first schedule to be evaluated is prime vaccination with MVA-BN-Filo followed by a boost with Ad26.ZEBOV 14 days later. In the second schedule, Ad26.ZEBOV is used to prime a filovirus-specific immune response and MVA-BN-Filo is used to boost the immune response 28 days later. The EBOV GP that circulated in West Africa has 97% homology to the EBOV GP used in this vaccine regimen.[3]

For the prevention of Ebola virus disease, short vaccination schedules may be relevant in the context of the epidemic and suitable for use during acute outbreaks of Ebola. When the current outbreak is under control, longer vaccination schedules may be more relevant than the shorter prime-boost intervals provided a more persistent immune response is observed.

The 2 different time intervals (of 14 or 28 days) between the prime and the boost vaccination will be evaluated for safety and tolerability as well as for immunogenicity. These prime-boost

regimens will differ in the sequence of vaccination and timing of the boost vaccination depending on the sequence, while the dose of each vaccine will be identical. The vaccine consists of Ad26.ZEBOV at a dose of $5x10^{10}$ vp and MVA-BN-Filo at a dose of $1x10^{8}$ infectious units (Inf U, nominal titer). The MVA-BN-Filo dose to be used corresponds to the dose of $1x10^{8}$ $TCID_{50}$ that is used in the current Phase 1 studies.

The 14-, 28-day prime-boost intervals are being evaluated in the current Phase 1 studies. Preliminary data from a small group of healthy subjects in the VAC52150EBL1001 and 1002 studies showed that both regimens, namely 14-day MVA prime/Ad26 boost regimen and 28-day Ad26 prime/MVA boost regimen, were immunogenic.

This Phase 2 study will be conducted to enlarge the safety and immunogenicity database for the Ad26.ZEBOV and MVA-BN-Filo prime-boost regimens in healthy subjects and to extend the population studied in Phase 1 to include subjects up to and including 70 years of age and HIV-infected subjects. HIV-infected subjects are included in this study because they represent a significant minority population whose response to vaccination needs to be understood in order to develop an effective prophylactic vaccine against Ebola.

2. OBJECTIVES AND HYPOTHESIS

2.1. Objectives

Primary Objectives

The primary objectives are:

- To assess the safety and tolerability of different vaccination schedules of Ad26.ZEBOV and MVA-BN-Filo administered IM as heterologous prime-boost regimens in healthy and in HIV-infected adults, with Ad26.ZEBOV prime and MVA-BN-Filo boost vaccination on Days 1 and 29, respectively and MVA-BN-Filo prime and Ad26.ZEBOV boost vaccination on Days 1 and 15, respectively.
- To assess the immune responses to the EBOV GP (as measured by ELISA antibody concentration) of different vaccination schedules of Ad26.ZEBOV and MVA-BN-Filo administered IM as heterologous prime-boost regimens in healthy and in HIV-infected adults, with Ad26.ZEBOV prime and MVA-BN-Filo boost vaccination on Days 1 and 29, respectively and MVA-BN-Filo prime and Ad26.ZEBOV boost vaccination on Days 1 and 15, respectively.

Secondary Objective

To compare safety and tolerability of both Ad26.ZEBOV/MVA-BN-Filo and MVA-BN-Filo/Ad26.ZEBOV regimens between healthy and HIV-infected adults.

Exploratory Objectives

The exploratory objectives include the following. Some exploratory objectives may not be performed if reagents or assays are not available.

- To assess the humoral immune responses further regarding kinetics and durability as well as neutralizing antibody responses directed against EBOV GP induced by the heterologous prime-boost regimen as measured by a virus neutralization assay, at relevant time points of different vaccination schedules of Ad26.ZEBOV and MVA-BN-Filo.
- To assess the kinetics and durability of cellular immune responses in healthy and HIV-infected adults for EBOV GP, by vaccine regime.
- To compare kinetics and durability of humoral and cellular immune responses to EBOV GP of Ad26.ZEBOV/MVA-BN-Filo and MVA-BN-Filo/Ad26.ZEBOV regimens between healthy and HIV-infected adults.
- To assess the kinetics and durability of humoral immune responses, including the development of neutralizing antibodies, in healthy and HIV-infected adults for other filovirus GP.
- To assess the kinetics and durability of cellular immune responses in healthy and HIV-infected adults for other filovirus GP.
- To compare kinetics and durability of humoral and cellular immune responses to other filovirus GP, of Ad26.ZEBOV/MVA-BN-Filo and MVA-BN-Filo/Ad26.ZEBOV regimens between healthy and HIV-infected adults.
- To assess the frequency, magnitude, and durability of anti-vector responses in healthy and HIV-infected adults.
- To compare kinetics and durability of humoral and cellular immune responses to the EBOV and other filovirus GP of Ad26.ZEBOV/MVA-BN-Filo and MVA-BN-Filo/Ad26.ZEBOV regimens between adults aged 18-50 and aged 51-70.
- To assess the impact of CD4+ count on safety and immunogenicity of Ad26.ZEBOV/MVA-BN-Filo and MVA-BN-Filo/Ad26.ZEBOV regimens in HIV-infected adults.
- To assess changes in HIV RNA and HIV specific immune response (using HIV antigens in place of Ebola GP) associated with vaccination with Ad26.ZEBOV/MVA-BN-Filo and MVA-BN-Filo/Ad26.ZEBOV regimens in HIV-infected adults.
- To assess humoral immune responses in genital, rectal and oral secretions in a subset of healthy and HIV-infected adults at selected sites for EBOV and other filovirus GP.
- To explore the impact of host genetics on immune responses to the Ad26.ZEBOV/MVA-BN-Filo and MVA-BN-Filo/Ad26.ZEBOV prime-boost regimens in healthy and HIV-infected adults.
- To assess immune epitope breadth elicited by the Ad26.ZEBOV/MVA-BN-Filo and MVA-BN-Filo/Ad26.ZEBOV prime-boost regimens in healthy and HIV-infected adults.
- To assess the immunoglobulin subclass, glycosylation and effector functions of humoral responses to EBOV and other filovirus GP.

- To describe B cell, helper T cell, and cytotoxic T cell responses elicited by the Ad26.ZEBOV/MVA-BN-Filo and MVA-BN-Filo/Ad26.ZEBOV prime-boost regimens in healthy and HIV-infected adults.
- To explore immune inflammatory responses elicited by the Ad26.ZEBOV/MVA-BN-Filo and MVA-BN-Filo/Ad26.ZEBOV regimens in healthy and HIV-infected adults on a multiplex array platform to evaluate cytokine and soluble factor responses to vaccination.

2.2. Hypothesis

No formal statistical hypothesis testing is planned for this study. The primary purpose of the study is to provide descriptive information regarding safety and immunogenicity of the 2 vaccination regimens within cohorts of healthy and HIV-infected subjects.

3. STUDY DESIGN AND RATIONALE

3.1. Overview of Study Design

This is a randomized, observer-blind, placebo-controlled, parallel-group, multicenter, 2-part, Phase 2 study to evaluate the safety, tolerability and immunogenicity of different vaccination regimens using Ad26.ZEBOV at a dose of $5x10^{10}$ vp and MVA-BN-Filo at a dose of $1x10^{8}$ Inf U (nominal titer), administered IM. The first regimen to be evaluated is prime vaccination with MVA-BN-Filo followed by a boost vaccination with Ad26.ZEBOV 14 days later. The second regimen to be evaluated is prime vaccination with Ad26.ZEBOV followed by a boost vaccination with MVA-BN-Filo 28 days later. These regimens will be evaluated in healthy and HIV-infected adults.

A planned total of 575 subjects will be enrolled, with approximately 300 healthy subjects and 275 HIV-infected subjects. Eligible subjects are those who have never received a candidate Ebola vaccine and have no prior exposure to EBOV (including travel to epidemic Ebola areas less than 1 month prior to screening) or a diagnosis of Ebola virus disease.

There are 2 parts to this study that will run in parallel. Part 1 features 2 cohorts consisting of 50 healthy adults and 25 HIV-infected adults, aged 18-70 years inclusive. Within each cohort, subjects will be randomized in a 4:1 ratio to receive Ad26.ZEBOV and MVA-BN-Filo or placebo: MVA-BN-Filo prime vaccination followed by Ad26.ZEBOV boost vaccination or placebo prime and boost vaccination 14 days later. Randomization will be done separately for healthy subjects and HIV-infected subjects. Part 1 will be conducted exclusively in the US at the WRAIR Clinical Research Center.

Part 2 of the study will investigate 2 vaccination regimens: Ad26.ZEBOV prime vaccination followed by MVA-BN-Filo boost vaccination 28 days later (Group 1) and MVA-BN-Filo prime vaccination followed by Ad26.ZEBOV boost vaccination 14 days later (Group 2). Group 1 includes 2 cohorts consisting of 200 healthy adults and 200 HIV-infected adults, aged 18-70 years inclusive, randomized in a 4:1 ratio to receive Ad26.ZEBOV and MVA-BN-Filo or placebo: Ad26.ZEBOV as prime vaccination followed by MVA-BN-Filo as boost vaccination or placebo prime and boost vaccination 28 days later. Group 2 includes 2 cohorts consisting of 50 healthy adults and 50 HIV-infected adults, aged 18-70 years inclusive, randomized in a

4:1 ratio to receive Ad26.ZEBOV and MVA-BN-Filo or placebo: MVA-BN-Filo prime vaccination followed by Ad26.ZEBOV boost vaccination or placebo prime and boost vaccination 14 days later, as shown in Figure 2 and Table 1. Randomization will be done separately for healthy subjects and HIV-infected subjects. Randomization of HIV-infected subjects in Part 2 will be contingent upon acceptable safety data from HIV-infected subject from Part 1.

An IDMC will be commissioned for this study. Refer to Section 11.7 for details.

Within each part, study-site personnel (except for those with primary responsibility for study vaccine preparation and dispensing), sponsor personnel and subjects will be blinded to the study vaccine allocation until all subjects in that part have completed the 6-month post-boost visit or discontinued earlier and the database has been locked for that part. For any interim analyses performed in a part before database lock, study-site personnel (except for those with primary responsibility for study vaccine preparation and dispensing), the sponsor (except for specifically designated sponsor personnel who are independent of the study) and subjects will remain blinded to study vaccine allocation. Refer to Section 5 for further details on blinding in case of interim analyses.

All subjects will receive the study vaccine (Ad26.ZEBOV, MVA-BN-Filo or placebo) IM in the deltoid muscle. In Part 1 and Part 2, Group 2, all subjects will receive MVA-BN-Filo ($1x10^8$ Inf U, nominal titer) or placebo on Day 1 followed by Ad26.ZEBOV ($5x10^{10}$ vp) or placebo on Day 15. In Part 2, Group 1, all subjects will receive Ad26.ZEBOV ($5x10^{10}$ vp) or placebo on Day 1, followed by MVA-BN-Filo ($1x10^8$ Inf U, nominal titer) or placebo on Day 29.

The study consists of a screening phase of up to 8 weeks (starting from the moment the subject signs the ICF), a vaccination phase, in which subjects will be vaccinated at baseline (Day 1) followed by a boost vaccination on Day 15 or 29, and a post-boost follow-up phase of maximum 1 year post-boost vaccination. When all subjects in a part have completed the 6-month post-boost visit or discontinued earlier, and the database for that part has been locked, the part will be unblinded. At that time, the subjects who received placebo in that part will be informed that they have completed the study and no further follow-up is required. However, subjects who received placebo and reach the 1-year post-boost visit (Part 1 and Part 2, Group 2: Day 380; Part 2, Group 1: Day 394) prior to unblinding will be required to attend the 1-year post-boost visit. The subjects who received Ad26.ZEBOV and MVA-BN-Filo will continue the study until the 1-year post-boost vaccination visit to assess long-term safety and immunogenicity.

While Part 1 was enrolling, the sponsor halted all vaccinations in this study due to the occurrence of a serious and very rare condition, Miller Fisher syndrome, reported in study VAC52150EBL2001, until a revised ICF was prepared, and approval to restart the current study was granted by the relevant competent authority. As a result of the delay, some subjects in Part 1 may reach the Day 380 visit prior to unblinding of Part 1. Female subjects in Part 2 who became pregnant with estimated conception within 28 days after vaccination with MVA-BN-Filo (or placebo) or within 3 months after vaccination with Ad26.ZEBOV (or placebo) (unless local regulations have additional requirements for follow up) and children born to vaccinated female subjects in Part 2 who became pregnant with estimated conception within 28 days after

vaccination with MVA-BN-Filo (or placebo) or within 3 months after vaccination with Ad26.ZEBOV (or placebo) will be eligible for enrollment into the VAC52150EBL4001 Vaccine Development Roll-over study for long-term surveillance (for a total of up to 60 months after the prime vaccination). After unblinding, only female subjects and children born to female subjects (with estimated conception within the period stated above) in Part 2 who received Ad26.ZEBOV and/or MVA-BN-Filo will be approached to consent for enrollment in the VAC52150 Vaccine Development Roll-over study for long-term surveillance. After unblinding, female subjects and children born to female subjects (with estimated conception within the period stated above) in Part 2 who received placebo and had already been enrolled into the VAC52150 Vaccine Development Roll-over study will be discontinued from further participation in the roll-over study.

As of amendment 4 implementation, no subjects in Part 1 will be approached anymore in this study to roll-over to the VAC52150EBL4001 study.

In the event that the study is paused in the future, subjects who were primed and awaiting boost will be followed every 3 months until 1 year for ascertainment of serious adverse events and IREs. Subjects who received both prime and boost will continue to follow the protocol as written. Subjects who are awaiting prime vaccination may need to be rescreened under a new subject number, repeating all screening procedures except the test of understanding (TOU) (unless the TOU has been revised in the meantime, in which case it needs to be taken again).

A schematic overview of the study design is presented in Figure 2.

Figure 2: Schematic Overview of the Study

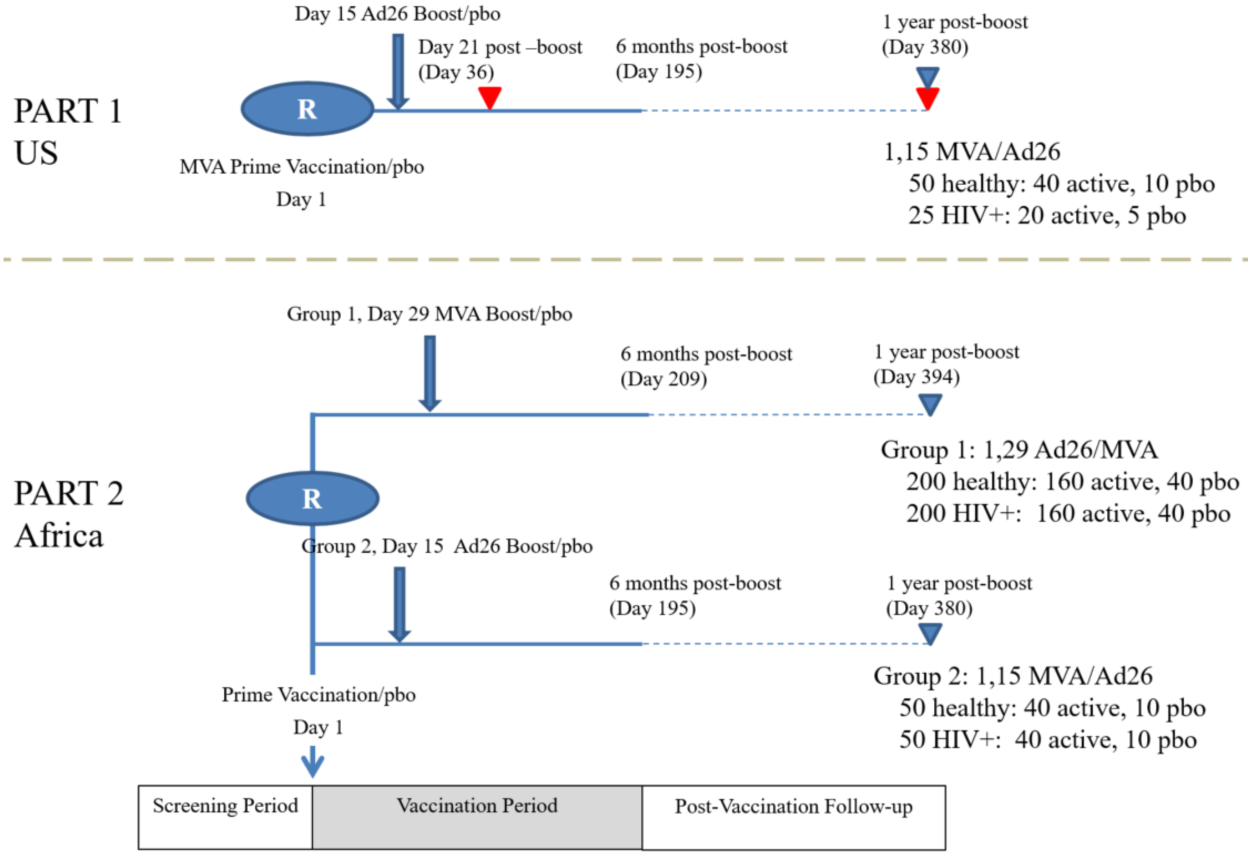

HIV+: HIV-infected; pbo: placebo; ▼: time of interim analysis; ▼: time of final analysis

The baseline visit may be scheduled as soon as the results of all screening assessments are known (but should occur within 8 weeks from screening, see Section 9.1.2) and show that the subject is eligible for inclusion. The administration of the prime vaccination will occur on Day 1 (baseline), after the completion of all baseline assessments.

All subjects will receive the study vaccine (Ad26.ZEBOV, MVA-BN-Filo or placebo) IM (0.5 mL) in the deltoid muscle:

- Ad26.ZEBOV ($5x10^{10}$ vp) on Day 1, followed by a boost vaccination of MVA-BN-Filo ($1x10^{8}$ Inf U) on Day 29 (Part 2, Group 1) *OR*
- MVA-BN-Filo ($1x10^{8}$ Inf U) on Day 1, followed by a boost vaccination of Ad26.ZEBOV ($5x10^{10}$ vp) on Day 15 (Part 1 and Part 2, Group 2)
- Placebo (0.9% saline) on Day 1, followed by a boost vaccination of placebo (0.9% saline) on Day 15 or 29.

Refer to Section 6 for further details on dosage and administration. After each vaccination, subjects will remain at the site for a total of 30 (±10) minutes post-vaccination to monitor for the

development of any acute reactions, or longer if deemed necessary by the investigator (eg, in case of grade 3 adverse events).

Safety will be assessed by collection of solicited local and systemic adverse events (reactogenicity), unsolicited adverse events, serious adverse events, and IREs. The subjects will be closely observed by study-site personnel for 30 (±10) minutes after each vaccination to monitor for the development of any acute reactions, or longer if deemed necessary by the investigator (eg, in case of grade 3 adverse events). Any unsolicited, solicited local or systemic adverse events occurring while on site will be documented. Upon discharge from the site, subjects will receive a diary, a thermometer and a ruler to measure body temperature and solicited local reactions. Subjects will be instructed to record solicited local and systemic adverse events in the diary in the evening after each vaccination and then daily for the next 7 days at approximately the same time each day. Diaries should be completed at home by the subject. For subjects having difficulty completing the diary independently, the sites will make arrangements to have the diary completed according to their local practice. The investigator will document unsolicited adverse events from signing of the ICF onwards until 42 days post-boost, and serious adverse events and IREs from signing of the ICF onwards until the end of the study. The primary endpoints are adverse events, serious adverse events, IREs and solicited local and systemic adverse events (see Section 9.2.1). Adverse events that are ongoing at 42 days post-boost vaccination will be followed until resolution or stabilization. Other safety assessments include physical examination, vital signs (blood pressure, pulse/heart rate, body temperature), clinical laboratory and pregnancy testing.

The investigators, together with the sponsor's study responsible physician, will be responsible for the safety monitoring of the study, and will halt vaccination of further subjects in case any of the pre-specified pausing rules described in Section 9.3.2 have been met or in case of other severe safety concerns. Criteria for postponement of vaccination at the scheduled time for vaccine administration and contraindications to boost vaccination have been defined and will be applied by the investigator (see Sections 6.2 and 6.3 respectively). Discontinuation of study vaccine should occur in any subject meeting the criteria outlined in Section 10.2.

In addition, the investigator will collect samples for immunogenicity assessments at the time points indicated in the Time and Events Schedule. Samples to assess humoral responses and samples to assess cellular immune responses will be taken from all subjects. In Part 2, a subset of approximately 40% will also participate in the optional mucosal assessments. Subjects giving informed consent for the study will be informed that their leftover blood and secretion samples will be stored for potential future research. Subjects can withdraw consent for their samples to be used for future research at any time (see Section 16.2.6).

An interim analysis will be conducted when all subjects in Part 1 have completed the 21-day post-boost visit, or discontinued earlier. The purpose of this interim analysis is to evaluate the effect of vaccination on the HIV viral load of HIV-infected subjects from a safety perspective. This interim analysis will be reviewed by the Protocol Safety Review Team (PSRT) and forwarded to the IDMC for review. An additional interim analysis will be done when all subjects in Part 1 have completed the 1-year post-boost visit or discontinued earlier.

Additional (ad hoc) interim analyses may be performed during the study for the purpose of informing future vaccine-related decisions in a timely manner. The results will not influence the conduct of the study in terms of early termination or later safety or immunogenicity endpoint assessments unless a significant safety concern has been identified.

The final analysis will be performed when all subjects have completed the last study-related visit or discontinued earlier.

After completing the present study, female subjects who became pregnant with estimated conception within timelines as indicated before and children born to female subjects (with estimated conception within the period stated above) in Part 2 will be approached to consent for enrollment into the VAC52150 Vaccine Development Roll-over study for long-term surveillance (see Section 9.1.5 for details).

3.2. Study Design Rationale

The study design is largely driven by the accelerated development approach of the Ad26.ZEBOV and MVA-BN-Filo prime-boost regimen to provide a robust safety and immunogenicity database to support registration. Further, preliminary data on the 14-day MVA prime/Ad26 boost regimen from ongoing Phase 1 studies have shown this regimen to be immunogenic and thus appropriate to consider for use in an outbreak setting. This study proposes to satisfy these development objectives: enlarge the safety and immunogenicity database in adults, contribute to the safety and immunogenicity data in HIV-infected adults and enhance the experience of the 14-day MVA/Ad26 prime/boost regimen.

Control and Blinding

Randomization will be used to minimize bias in the assignment of subjects to vaccination schedules (groups), to increase the likelihood that known and unknown subject attributes (eg, demographic and baseline characteristics) are evenly balanced across groups, and to enhance the validity of possible statistical comparisons across groups.

In addition, randomization will be used to minimize bias in the assignment of subjects to study vaccine (Ad26.ZEBOV and MVA-BN-Filo versus placebo). Placebo recipients are included for blinding purposes and safety analyses, and will provide control specimens for immunogenicity assays. A placebo control will be used up to 6 months post-boost to establish the frequency and magnitude of changes in clinical and immunologic endpoints that may occur in the absence of the Ad26.ZEBOV and MVA-BN-Filo. The nature of the study vaccine (active versus placebo) will be blind to reduce potential bias during data collection and evaluation of clinical safety endpoints. Blinding will be guaranteed by preparation of study vaccine by unblinded qualified study-site personnel not involved in any other study-related procedure, and by the administration of vaccine in a masked syringe by a blinded vaccine administrator (see Definitions of Terms). No additional placebo injections will be administered to mask the vaccination schedule across groups.

Study Groups

There are two vaccination schedules being investigated in this study. One schedule, MVA-BN-Filo prime followed by Ad26.ZEBOV 14 days later, is being studied in Part 1 and Part 2, Group 2. The other schedule, Ad26.ZEBOV prime followed by MVA-BN-Filo 28 days later, is being studied in Part 2, Group 1. The dose of each vaccine, regardless of the order of administration, remains the same. The dose of Ad26.ZEBOV is $5x10^{10}$ vp and the dose of MVA-BN-Filo is $1x10^8$ Inf U, which corresponds to the dose of $1x10^8$ $TCID_{50}$ that is used in the current Phase 1 studies. The safety, tolerability and immunogenicity results for the 2 vaccination schedules will be evaluated in the study.

The study population consists of healthy subjects and HIV-infected subjects, both allocated to the different vaccination schedules in Part 2. This will allow for evaluation of the vaccine schedules in both populations.

Future Research

Subjects giving informed consent for the study will be informed that their leftover blood and secretion samples (serum and/or peripheral blood mononuclear cells [PBMC]) will be stored for potential future research (see Section 16.2.6). Future scientific research may be conducted to further investigate Ebola vaccine- and disease-related questions. This may include the development of new or the improvement of existing techniques to characterize EBOV-directed immune responses or diagnostic tests. Subjects can withdraw consent for their samples to be used for future research at any time.

4. SUBJECT POPULATION

Screening of subjects for eligibility will be performed within 8 weeks before administration of study vaccine on Day 1. Subjects must sign an ICF indicating that he or she understands the purpose of and procedures required for the study and is willing to participate in the study. Signing of the ICF needs to be done before the first study-related activity.

The inclusion and exclusion criteria for enrolling subjects in this study are described in the following 2 subsections. If there is a question about the inclusion or exclusion criteria below, the investigator must consult with the appropriate sponsor representative and resolve any issues before enrolling a subject in the study. Waivers are not allowed.

For a discussion of the statistical considerations of subject selection, refer to Section 11.1, Sample Size Determination.

NOTE: The investigator should ensure that all study enrollment criteria have been met at the end of the screening period. If a subject’s clinical status changes (including any available laboratory results or the receipt of additional medical records) after screening but before Day 1 so the subject no longer meets the eligibility criteria, then the subject should be excluded from further participation in the study. Section 17.4 describes the required documentation to support meeting the enrollment criteria.

4.1. Inclusion Criteria

4.1.1. Inclusion Criteria for Healthy Adult Subjects

Each potential subject must satisfy all of the following criteria to be enrolled in the study.

1. Each subject must sign an ICF indicating that he or she understands the purpose of and procedures required for the study and is willing to participate in the study. In case the subject cannot read or write, the procedures must be explained and informed consent must be witnessed by a literate third party not involved with the conduct of the study.
2. Subject must be a man or woman aged 18 to 70 years of age, inclusive at screening.
3. Subject must be healthy in the investigator's clinical judgment on the basis of medical history, physical examination and vital signs performed at screening.
4. Subject must be healthy on the basis of clinical laboratory tests and ECG (only in subjects >50 years) performed at screening. If the results of the laboratory screening tests and ECG are outside the institutional normal reference ranges, the subject may be included only if the investigator judges the abnormalities or deviations from normal to be not clinically significant or to be appropriate and reasonable for the population under study. This determination must be recorded in the subject's source documents and initialed by the investigator. However, the subject should not be included when hemoglobin is lower than the institutional normal reference range (or below the values in Attachment 3 when that range is not available).

   Note: *The safety laboratory assessments at screening are to be performed within 28 days prior to the prime vaccination and may be repeated if they fall outside this time window.*

   Note: *In case of menstruation, urinalysis must be postponed but a result should be available before the prime vaccination. All urinalyses should be collected as freshly voided, mid-stream, clean catch samples obtained after proper cleansing.*

   Note: *If laboratory screening tests are out of range and deemed clinically significant, repeat of screening tests is permitted only once using an unscheduled visit during the screening period to assess eligibility.*

5. Criterion modified per Amendment 3.

5.1 Contraceptive use by women should be consistent with local regulations regarding the use of contraceptive methods for subjects participating in clinical studies.

Before randomization (on Day 1), subjects who were born female must be either:

a. Of childbearing potential and practicing a highly effective method of birth control consistent with local regulations regarding the use of birth control methods for subjects participating in clinical studies, beginning at least 28 days prior to vaccination until at least 3 months after the boost vaccination. The sponsor considers the following methods of birth control to be highly effective: established use of oral, injected or implanted hormonal methods of contraception; placement of an intrauterine device or intrauterine system; barrier methods (condom or occlusive cap [diaphragm or cervical/vault caps] with spermicidal foam/gel/film/cream/suppository); male partner sterilization (the vasectomized

partner should be the sole partner for that subject). More restrictive measures may be required by the site.

Note: Reliance on abstinence or natural family planning are not acceptable birth control methods for sexually active women of child-bearing potential.

b. Not of childbearing potential defined as:

- postmenopausal
  A postmenopausal state is defined as >45 years of age with amenorrhea for at least 2 years
- permanently sterile
  Permanent sterilization methods include bilateral tubal occlusion (which includes tubal ligation procedures as consistent with local regulations), hysterectomy, bilateral salpingectomy, bilateral oophorectomy
- incapable of pregnancy.

Note: If the social situation of a woman changes after start of the study (eg, woman who is not heterosexually active becomes active), she must begin a highly effective method of birth control, as described above.

6. A woman of childbearing potential must have a negative urine β-human chorionic gonadotropin (β-hCG) pregnancy test at screening and a negative urine β-hCG pregnancy test immediately prior to each study vaccine administration.

   Note: If the pregnancy test result is positive, in order to maintain subject confidentiality, the investigator will ensure adequate counseling and follow-up will be made available.

7. A man who is sexually active with a woman of childbearing potential must be willing to use condoms for sexual intercourse beginning prior to prime vaccination until at least 3 months after the boost vaccination, unless a vasectomy was performed more than 1 year prior to screening.
8. Subject must be available and willing to participate for the duration of the study visits and follow-up.
9. Subject must be willing and able to comply with the protocol requirements, including the Prohibitions and Restrictions specified in Section 4.3.
10. Subject must be willing to provide verifiable identification.
11. Subject must have a means to be contacted.
12. Subject must pass the TOU (passing score: ≥90%) (Attachment 1).

    Note: If subjects fail the TOU test on the first attempt, subjects must be retrained on the purpose of the study and must take the test again (2 repeats are allowed). If subjects fail on the third attempt, they should not continue with the consenting and screening processes.

4.1.2. Additional Inclusion Criteria for HIV-infected Subjects

All of the inclusion criteria for healthy adult subjects (see Section 4.1.1) must be met by adults who are HIV-infected with the following additions/clarifications:

13. Subjects must have a positive HIV-1 and/or -2 serology test within 6 months of screening, including the day of screening.
14. Subjects must have a screening CD4+ cell count >200 cells/µL
15. Criterion modified per Amendment 3.

15.1 Investigators should use their best clinical judgment when determining stability of highly active antiretroviral therapy (HAART). The occasional missed dose would not make a subject ineligible for the study. In uncertain cases, the subject's eligibility should be discussed with the sponsor.

Part 1: All subjects must be on a stable HAART regimen for 4 weeks prior to screening, taking into account the following criteria.

Part 2: Subjects with screening CD4+ cell count <350 cells/µL must be on a stable HAART regimen for 4 weeks prior to screening, taking into account the following criteria:

- Antiretroviral therapy (ART) is considered stable if subjects did not change their antiretroviral drugs within the last 4 consecutive weeks prior to their first screening visit. Changes in formulations are allowed.
- Subject must be willing to continue their ART throughout the study as directed by their local physician.

Subjects newly diagnosed with HIV infection at screening must be referred for continuing care and if started on therapy, they must be rescreened using a new subject number with repeat laboratory testing (see Section 9.1.2) after they have been on that regimen for 4 weeks.

16. Subject must be in an otherwise reasonably good medical condition (absence of acquired immunodeficiency syndrome [AIDS]-defining illnesses or clinically significant disease), diagnosed on the basis of physical examination, medical history and the investigator's clinical judgment at the time of screening (refer to Section 4.1.1 for details).

Note for Part 2 subjects: Otherwise healthy HIV-infected individuals with CD4+ cell count ≥350 cells/µL who are not taking ART may be eligible for the study if they are engaged in ongoing HIV care and meet all other enrollment criteria.

4.2. Exclusion Criteria

4.2.1. Exclusion Criteria for Healthy Adult Subjects

Any potential subject who meets any of the following criteria will be excluded from participating in the study.

1. Has received any candidate Ebola vaccine.
2. Diagnosed with Ebola virus disease, or prior exposure to EBOV, including travel to epidemic Ebola areas less than 1 month prior to screening.

   Note: Participation of international volunteers to Ebola operations is allowed, but they should comply with the prohibitions and restrictions as specified in Section 4.3.
3. Has received any experimental candidate Ad26- or MVA-based vaccine in the past or received any other investigational drug or investigational vaccine or used an invasive investigational medical device within 3 months prior to screening.

   Note: Receipt of any approved vaccinia/smallpox vaccine or Ad-based candidate vaccine other than Ad26 at any time prior to study entry is allowed.
4. Known allergy or history of anaphylaxis or other serious adverse reactions to vaccines or vaccine products (including any of the constituents of the study vaccines [eg, polysorbate 80, ethylenediaminetetraacetic acid (EDTA) or L-histidine for Ad26.ZEBOV vaccine; tris (hydroxymethyl)-amino methane (THAM) for MVA-BN-Filo vaccine]), including known allergy to egg, egg products and aminoglycosides.
5. Subject with acute illness (this does not include minor illnesses such as diarrhea or mild upper respiratory tract infection) or body temperature ≥38.0ºC on Day 1 will be excluded from prime vaccination at that time, but may be rescheduled for prime vaccination at a later date.
6. HIV type 1 or type 2 infection.

   Note: If the HIV test result is positive, in order to maintain subject confidentiality, the investigator will ensure adequate counseling and follow-up will be made available.
7. A woman who is pregnant, breast-feeding or planning to become pregnant while enrolled in the study or within at least 3 months after the boost vaccination.
8. Presence of significant conditions (eg, history of seizure disorders, (auto)immune disease or deficiency, any spleen disease, active malignancy, ongoing tuberculosis treatment, other systemic infections) or clinically significant findings during screening of medical history, ECG (only in subjects >50 years), physical examination, vital signs or laboratory testing for which, in the opinion of the investigator, participation would not be in the best interest of the subject (eg, compromise the safety or well-being) or that could prevent, limit, or confound the protocol-specified assessments.

Note: Subjects who have recently received treatment for acute, uncomplicated malaria are eligible for randomization if at least 3 days have elapsed from the conclusion of a standard, recommended course of therapy for malaria; subjects who are acutely ill with malaria at the time of the initial screening should complete therapy and wait an additional 3 days after completion before they can enter the screening phase for the study.

Note: Subjects with sickle cell trait can be included.

9. History of or underlying liver or renal insufficiency, or significant cardiac, vascular, pulmonary (eg, persistent asthma), gastrointestinal, endocrinic, neurologic, hematologic, rheumatologic, psychiatric or metabolic disturbances or eczema.

10. History of malignancy other than squamous cell or basal cell skin cancer, unless there has been surgical excision that is considered cured. Subjects with malignancies who are currently being treated or are not surgical cures are excluded.

11. Major surgery (per the investigator's judgment) within the 4 weeks prior to screening or planned major surgery through the course of the study (from screening until completion of the study, see Section 10.1).

12. Post-organ and/or stem cell transplant whether or not with chronic immunosuppressive therapy.

13. Receipt of any disallowed therapies as noted in Section 8 before the planned administration of the prime vaccine on Day 1.

14. Is currently participating in or planning to participate in another clinical study during the study.

    Note: Participation in an observational clinical study is allowed.

15. Donation of a unit of blood within 8 weeks before Day 1 or plan to donate blood until 42-days post-boost.

16. Receipt of blood products or immunoglobulin within 3 months prior to screening and during participation in the study.

17. Current or past abuse of alcohol, recreational or narcotic drugs, which in the investigator's opinion would compromise the subject's safety and/or compliance with the study procedures.

18. History of chronic urticaria (recurrent hives).

19. Subject cannot communicate reliably with the investigator.

20. Subject who, in the opinion of the investigator, is unlikely to adhere to the requirements of the study.

21. Employee of the investigator or study site, with direct involvement in the proposed study or other studies under the direction of that investigator or study site, as well as family members of the employees or the investigator.

4.2.2. Additional Exclusion Criteria for HIV-infected Subjects

HIV-infected subjects must not meet any of the exclusion criteria for healthy adult subjects (see Section 4.2.1) with the exception of exclusion criterion 6: HIV-1 or HIV-1/2 infection. HIV-infected subjects must have a positive HIV-1 or HIV-1/2 test within 6 months of screening, including the day of screening.

4.3. Prohibitions and Restrictions

Potential subjects must be willing and able to adhere to the following prohibitions and restrictions during the course of the study to be eligible for participation:

1. Female subjects of childbearing potential must remain on a highly effective method of birth control consistent with local regulations regarding the use of birth control methods for subjects participating in clinical studies (see inclusion criteria) until at least 3 months after the boost vaccination. If the social situation of a female subject changes after start of the study (eg, female subject who is not heterosexually active becomes active), she must begin a highly effective method of birth control, as described above in Sections 4.1 and 4.2, until at least 3 months after the boost vaccination.

   Note: Prior to each study vaccine administration, a urine pregnancy test should be performed for female subjects of childbearing potential.

2. Male subjects who did not have a vasectomy performed more than 1 year prior to screening and who are sexually active with a female subject of childbearing potential must use condoms for sexual intercourse until at least 3 months after the boost vaccination.

3. Women should not breast-feed while enrolled in the study until at least 3 months after the boost vaccination.

4. Traveling to epidemic Ebola areas is prohibited while enrolled in the study from the start of screening onwards until the 6-month post-boost visit. Subjects who subsequently travel to these areas should have returned at least 1 month before the 1-year post-boost visit. Any traveling to epidemic Ebola areas should be documented in the case report form (CRF).

   Note: Subjects travelling to epidemic Ebola areas will be excluded from follow-up collection of blood for immunogenicity assessments if they contract Ebola virus disease (see also exclusion criterion #2 in Section 4.2.1).

5. Subjects should not use any disallowed therapies as described in Section 8.

5. TREATMENT ALLOCATION AND BLINDING

Vaccination Schedule Allocation

Central randomization will be implemented in this study. The randomization within each group will be balanced by using randomly permuted blocks. There will be stratification by age group (subjects ≥18 to ≤50 years versus subjects >50 years). The interactive web response system (IWRS) will assign a unique code, which will dictate the assignment and matching vaccination

schedule for the subject. The requestor must use his or her own user identification and personal identification number when contacting the IWRS, and will then give the relevant subject details to uniquely identify the subject.

For Part 1, subjects will be randomized in a 4:1 ratio to either receive MVA-BN-Filo or placebo on Day 1 followed by a boost vaccination of Ad26.ZEBOV or placebo 14 days later. Randomization will be done separately for healthy subjects (N=50) and HIV-infected subjects (N=25).

Part 2 will be conducted in parallel to Part 1 but has a different randomization scheme. In Part 2, a subject will be randomly enrolled in 1 of 2 groups at study entry in a 4:1 ratio of Group 1 versus Group 2. Within each group, subjects will be randomized in a 4:1 ratio to receive Ad26.ZEBOV and MVA-BN-Filo versus placebo. Randomization will be done separately for healthy subjects (N=250) and HIV-infected subjects (N=250). Randomization of HIV-infected subjects in Part 2 will be contingent upon acceptable safety data from HIV-infected subjects from Part 1.

Blinding

For each part, subjects and study-site personnel will be blinded to the study vaccine allocation until the last subject in that part has completed the 6-month post-boost visit or discontinued earlier and the database has been locked for that part, except for unblinded qualified study-site personnel with primary responsibility for study vaccine preparation and dispensing, and not involved in any other study-related procedure. The study vaccines will be administered by a blinded study vaccine administrator (see Definitions of Terms).

When all subjects in a part have completed the 6-month post-boost visit or discontinued earlier and the database for that part has been locked the part will be unblinded and the subjects who received placebo will be contacted by the site to communicate that they have completed the study and no further follow-up is required. However, subjects who received placebo and reach the 1-year post-boost visit (Part 1 and Part 2, Group 2: Day 380; Part 2, Group 1: Day 394) prior to unblinding will be required to attend the 1-year post-boost visit. Subjects who received Ad26.ZEBOV and MVA-BN-Filo will be followed up in an open-label fashion until the 1-year post-boost visit. For any interim analyses performed in a part before database lock, study-site personnel (except for those with primary responsibility for study vaccine preparation and dispensing), the sponsor (except for specifically designated sponsor personnel who are independent of the study) and subjects will remain blinded to study vaccine allocation (see below). Refer to Sections 3.1 and 11 for further details on unblinding and interim analyses.

The investigator will not be provided with randomization codes. The codes will be maintained within the IWRS, which has the functionality to allow the investigator to break the blind for an individual subject.

Data that may potentially unblind the study vaccine assignment (ie, study vaccine preparation/accountability data, or other specific laboratory data) will be handled with special care to ensure that the integrity of the blind is maintained and the potential for bias is minimized.

This can include making special provisions, such as segregating the data in question from view by the investigators, clinical team, or others as appropriate until the time of database lock and unblinding. The pharmacy and preparation of study vaccines will be monitored by an independent study vaccine monitor (see Section 17.8).

Under normal circumstances, the blind should not be broken in a part, until all subjects in that part completed the 6-month post-boost visit or discontinued earlier and the database has been locked for that part. Otherwise, the blind should be broken only if specific emergency treatment/course of action would be dictated by knowing the treatment status of the subject. In such cases, the investigator may in an emergency determine the identity of the treatment by contacting the IWRS. It is recommended that the investigator contacts the sponsor or its designee if possible to discuss the particular situation, before breaking the blind. Telephone contact with the sponsor or its designee will be available 24 hours per day, 7 days per week. In the event the blind is broken, the sponsor must be informed as soon as possible. The date and reason for the unblinding must be documented by the IWRS, in the appropriate section of the CRF, and in the source document. The documentation received from the IWRS indicating the code break must be retained with the subject's source documents in a secure manner.

In general, randomization codes will be disclosed fully for a part if all subjects in that part completed the 6-month post-boost visit or discontinued earlier. For any interim analysis performed in a part before database lock, the randomization codes and if required the translation of randomization codes into treatment and control groups, will be disclosed to those authorized and only for those subjects included in the interim analysis.

If the randomization code is broken by the investigator or the study-site personnel, the subject must discontinue further study vaccine administration (only applicable for subjects in the vaccination phase) and must be followed as appropriate (see Section 10.2 for details). If the randomization code is broken by the sponsor for safety reporting purposes, the subject should not discontinue further study vaccine administration and may remain in the study (if the randomization code is still blinded to the study-site personnel and the subject).

6. DOSAGE AND ADMINISTRATION

An overview of the study vaccination schedules is provided in Table 1.

Table 1: Study Vaccination Schedules Parts 1 and 2

**Part 1: US, n=75**			
**MVA-BN-Filo prime day 1, Ad26.ZEBOV boost day 15**			
Active		Placebo	
Healthy Adult	HIV+ Adult	Healthy Adult	HIV+ Adult
40	20	10	5
**Part 2: Africa, n=500**			
**Group 1: Ad26.ZEBOV prime day 1, MVA-BN-Filo boost day 29**			
Active		Placebo	
Healthy Adult	HIV+ Adult	Healthy Adult	HIV+ Adult
160	160	40	40
**Group 2: MVA-BN-Filo prime day 1, Ad26.ZEBOV boost day 15**			
Active		Placebo	
Healthy Adult	HIV+ Adult	Healthy Adult	HIV+ Adult
40	40	10	10

6.1. General Instructions and Procedures

All subjects will receive a vaccination, according to randomization, on Day 1 and on Day 15 (Part 1 and Part 2, Group 2), or Day 29 (Part 2, Group 1) at the following dose levels:

- Ad26.ZEBOV: $5x10^{10}$ vp, supplied in a single use vial (0.5 mL extractable)
- MVA-BN-Filo: $1x10^{8}$ Inf U (nominal titer; target fill is $1.9x10^{8}$ Inf U per dose, range: $1.27\text{-}2.67x10^{8}$ Inf U), supplied in a single use vial (0.5 mL extractable)
- Placebo: 0.9% saline (0.5 mL extractable)

Study vaccines (Ad26.ZEBOV, MVA-BN-Filo or placebo) will be administered as 0.5-mL IM injections into the deltoid muscle by a blinded study vaccine administrator. In each subject, the boost vaccination should preferably be administered in the opposite deltoid from the prime vaccination (unless local site reaction cannot be assessed reliably in the opposite arm) and it should be recorded in the CRF in which arm the vaccination has been administered. The injection site should be free from any injury, local skin problem, significant tattoo or other issue that might interfere with evaluating the arm after injection (eg, subjects with a history of skin cancer must not be vaccinated at the previous tumor site). No local or topical anesthetic will be used prior to the injection.

Discontinuation of study vaccine administration should occur in any subject meeting the criteria outlined in Section 10.2. Criteria for postponement of vaccination and contraindications to boost vaccination have been defined in Sections 6.2 and 6.3, respectively. Refer to Section 9.3.2 for details on the pre-specified pausing rules to halt vaccination of further subjects.

After each vaccination, subjects will remain at the site for a total of 30 (±10) minutes post-vaccination to monitor for the development of any acute reactions, or longer if deemed

necessary by the investigator (eg, in case of grade 3 adverse events). As with any vaccine, allergic reactions following vaccination with the study vaccine are possible. Therefore, appropriate drugs and medical equipment to treat acute anaphylactic reactions must be immediately available and a medically qualified member of study-site personnel trained to recognize and treat anaphylaxis must be present in the clinic during the entire vaccination procedure and post-vaccination monitoring period.

The investigator must provide emergency care as needed for any subject who experiences a life-threatening event. All sites will have facilities, equipment and the ability to manage an anaphylactic reaction. If additional therapy is required, the investigator will arrange for transport to the closest appropriate facility for continuing care.

The Site Investigational Product Procedures Manual specifies the maximum time that will be allowed between preparation and administration of the study vaccine.

6.2. Criteria for Postponement of Vaccination

A subject will not be given the prime or boost vaccination if he/she experiences any of the following events at the scheduled time for vaccination:

- Acute illness at the time of vaccination (this does not include minor illnesses such as diarrhea or mild upper respiratory tract infection)

  Note: If a subject is acutely ill with malaria at the time of either prime or boost vaccination, at least 3 days must elapse from the conclusion of a standard, recommended course of therapy for malaria before vaccination.

- Fever (body temperature ≥38.0°C) at the time of vaccination

Subjects experiencing any of the events described above may be vaccinated up to 10 days beyond the window allowed for the scheduled vaccination, or be withdrawn from that vaccination at the discretion of the investigator and after consultation with the sponsor (see Section 10.2).

Note: In case the boost vaccination is postponed, the timing of the post-boost visits will be planned relative to the actual vaccination day (see Section 9.1.1).

6.3. Contraindications to Boost Vaccination

A subject will not be given the boost vaccination if he or she experiences any of the following events at any time after the prime vaccination:

1. Any anaphylaxis that occurs between prime and boost vaccination either attributable to study vaccine or not *OR*
2. Generalized urticaria within 72 hours of vaccination considered to be at least possibly related to study vaccine *OR*
3. A serious adverse event considered to be at least possibly related to study vaccine *OR*

4. Injection site ulceration, abscess or necrosis considered to be at least possibly related to the study vaccine *OR*

5. Any other safety concern threatening the subject's safety or persisting clinically significant abnormality considered to be related to prime vaccination

Subjects experiencing any of the events described above must not receive any further study vaccine, but should be monitored for safety and for immunogenicity according to the protocol as described in Section 10.2.

An ad hoc IDMC meeting may be requested via the sponsor for any single event or combination of multiple events which are considered to jeopardize the safety of the subjects.

7. TREATMENT COMPLIANCE

All study vaccines will be administered on site by a blinded study vaccine administrator (see Definitions of Terms). The date and time of each study vaccine administration will be recorded in the CRF.

8. PRESTUDY AND CONCOMITANT THERAPY

Prestudy therapies administered up to 30 days prior to the start of screening and previous vaccinia/smallpox vaccination at any time prior to study entry must be recorded in the CRF.

Concomitant therapies must be recorded from screening onwards until the 42-day post-boost visit. Concomitant therapies should also be recorded after the 42-day post-boost visit but only if given in conjunction with serious adverse events and IREs that meet the criteria outlined in Sections 12.3.2 and 12.3.3, respectively. Any vaccines received during the study should be recorded.

All therapies (prescription or over-the-counter medications, including vaccines, vitamins, herbal supplements; non-pharmacologic therapies such as electrical stimulation, acupuncture, special diets, exercise regimens) must be recorded in the CRF. Recorded information will include a description of the type of the drug, treatment period, dosing regimen, route of administration, and its indication.

Subjects must use adequate birth control measures prior to randomization as described in Section 4.

Subjects are not allowed to have received any experimental candidate Ad26- or MVA-based vaccine in the past or received any other investigational drug or investigational vaccine or used an invasive investigational medical device within 3 months prior to screening. Receipt of any approved vaccinia/smallpox vaccine or Ad-based candidate vaccine other than Ad26 at any time prior to study entry is allowed.

Subjects are allowed to receive all routine immunizations according to local vaccination schedules, taking into account the following restrictions:

- Routine immunizations with inactivated vaccines should be administered at least 15 days before or after administration of any study vaccine to avoid any potential interference in efficacy of the routine immunizations or the interpretation of immune responses to study vaccines, as well as to avoid potential confusion with regard to attribution of adverse reactions.
- Routine immunizations with live attenuated vaccines are prohibited in the period from 30 days before baseline (Day 1) to 30 days after the boost vaccination.

However, if a vaccine is indicated in a post-exposure setting (eg, rabies or tetanus), it must take priority over the study vaccine.

Analgesic/antipyretic medications and nonsteroidal anti-inflammatory drugs may be used post-vaccination only in case of medical need (eg, body temperature ≥38.0 °C or pain) and their use must be documented. Use of these medications as routine prophylaxis within 24 hours prior to study vaccine administration is prohibited.

Chronic or recurrent use of medications that modify the host immune response (eg, cancer chemotherapeutic agents, systemic corticosteroids, immunomodulators) are prohibited.

HAART must be documented as a prior medication and any changes to the subject's HAART regimen must also be recorded. HIV-infected subjects who begin the study untreated may begin an HAART regimen during the course of the study as indicated by local guidelines. Adherence to HAART will be assessed at every visit.

The sponsor must be notified in advance (or as soon as possible thereafter) of any instances in which prohibited therapies are administered. Prohibited therapies will be captured as protocol deviations.

9. STUDY EVALUATIONS

9.1. Study Procedures

9.1.1. Overview

The Time and Events Schedule summarizes the frequency and timing of safety, tolerability and immunogenicity measurements and evaluations applicable to this study. Details for all study procedures are provided in the following sections. Additional unscheduled study visits may be required if in the investigator's opinion, further clinical or laboratory evaluation is needed.

Visit Windows

The screening visit has to be performed within 8 weeks prior to the baseline visit (ie, the day of the subject's prime vaccination, Day 1). If a subject did not receive study vaccine on the planned day of vaccination, the timings of the next visits post-vaccination (see Time and Events Schedule) will be determined relative to the actual day of vaccination. Visit windows that

will be allowed are summarized in Table 2. The subject should be encouraged to come on the exact day planned and use the visit window only if absolutely necessary.

Table 2: Visit Windows

Visit description	Day	Window
7 Days Post-prime Vaccination	Day 8 for all groups	±2 days
Boost Vaccination	Day 15 for Part 1 and Part 2, Group 2	±3 days
	Day 29 for Part 2, Group 1	
7 Days Post-boost Vaccination	Day 22 for Part 1 and Part 2, Group 2	±2 days
	Day 36 for Part 1 and Part 2, Group 1	
21 Days Post-boost Vaccination	Day 36 for Part 1 and Part 2, Group 2	±3 days
	Day 50 for Part 2, Group 1	
42 Days Post-boost Vaccination	Day 57 for Part 1 and Part 2, Group 2<br>Day 71 for Part 2, Group 1	±3 days
Follow-up 6 Months Post-boost	Day 195 for Part 1 and Part 2, Group 2	±15 days
	Day 209 for Part 2, Group 1	
Follow-up 1 Year Post-boost	Day 380 for Part 1 and Part 2, Group 2	±30 days
	Day 394 for Part 2, Group 1	

Blood Sampling Volumes

Approximately 571 to 675 mL of blood (including PBMC samples) will be drawn over a period of around 1 year, and remains well below the limits of standard blood donation. Additional blood may be drawn to confirm and follow-up on adverse events.

For details on the approximate blood sampling volumes collected by visit and the cumulative blood volumes, refer to the Time and Events Schedule.

Additional urine pregnancy tests may be performed, as determined necessary by the investigator or required by local regulation, to establish the absence of pregnancy at any time during the subject's participation in the study.

Repeat or unscheduled samples may be taken for safety reasons or for technical issues with the samples.

9.1.2. Screening Phase

Up to 8 weeks before baseline (the day of the subject's prime vaccination, Day 1) and after signing and dating the ICF (see Section 16.2.4), screening assessments will be performed as indicated in the Time and Events Schedule. Screening may be split into multiple days or visits. In exceptional cases, the screening phase can be extended if discussed with and approved (documented) by the sponsor, eg, if not all the test results become available during the allocated 8 weeks; this will be evaluated on a case-by-case basis. Each site-specific addendum will identify an estimate of the number of subjects that may be screened, as this will vary across all sites (Section 17.12). If a subject is a screen failure, the subject may be rescreened on 1 occasion after consultation and agreement with the sponsor medical monitor and after a new informed consent has been obtained. Subjects who are rescreened will be assigned a new subject number

and will repeat all screening procedures, except TOU (unless the TOU has been revised in the meantime, in which case it needs to be taken again).

For male subjects and female subjects of non-childbearing potential, there will be no minimum duration of the screening period and it will last only for the time required to verify eligibility criteria. For female subjects of childbearing potential, it should be confirmed that adequate birth control measures were used from at least 28 days before the prime vaccination with a negative urine β-hCG pregnancy test at screening and a negative urine pregnancy test immediately prior to each study vaccination (see Section 4). All male and female subjects, except for female subjects of non-childbearing potential, will be asked to use adequate birth control for sexual intercourse until at least 3 months after the boost vaccination (see Section 4.3).

Only subjects complying with the criteria specified in Section 4 will be included in the study. The investigator will provide detailed information on the study to the subject and will obtain written informed consent prior to each subject's study participation. The procedures indicated in the Time and Events Schedule will only be performed after the subject's written informed consent has been obtained.

After reading but before signing the ICF, the TOU will be conducted (or as required per local regulations and practices). Subjects who fail may repeat the test twice (and have to pass the third time to be eligible). Subjects must pass the TOU before starting the screening procedures. The TOU is a questionnaire to document the subject's understanding of the study (for details, see Section 16.1).

The following is performed to determine eligibility requirements as specified in the inclusion and exclusion criteria:

- Review of all inclusion and exclusion criteria
- Review of medical history (including concomitant diseases) and demographics
- Review of prestudy therapies (up to 30 days prior to the start of screening), previous vaccinia/smallpox vaccination if known (at any time prior to study entry) and concomitant therapies
- ECG recording (for subjects >50 years)
- Urine pregnancy test (for female subjects of childbearing potential)
- Full physical examination (including height and body weight; a genitourinary examination is not required)
- Measurement of vital signs (blood pressure, pulse/heart rate, body temperature)
- Blood sampling for hematology and chemistry (fasting or non-fasting)
- Urinalysis
- Serology testing: HIV type 1 or HIV type 1 and 2

- CD4+ cell count for HIV-infected subjects

All adverse events and pregnancies will be collected starting at the time a signed and dated ICF is obtained. Serious adverse events and IREs will be collected from signing of the ICF onwards until the end of the study.

The overall eligibility of the subject to participate in the study will be assessed once all screening values and results of any other required evaluations are available. Retesting of values (eg, safety laboratory) that lead to exclusion is allowed only once using an unscheduled visit during screening to assess eligibility. The safety laboratory assessments are to be performed within 28 days prior to the prime vaccination and may be repeated once if they fall outside this time window. Study subjects who qualify for inclusion will be contacted and scheduled for enrollment and prime vaccination within 8 weeks.

9.1.3. Vaccination Phase

If eligible, the subject will come for the baseline visit (Day 1). The investigator should ensure that all enrollment criteria have been met during screening. If a subject's clinical status changes (including any available laboratory results or receipt of additional medical records) after screening but before the prime vaccination (Day 1) such that the subject no longer meets all enrollment criteria, then the subject should be excluded from further participation in the study. If the initial laboratory sampling for hematology, chemistry, urinalysis and urine pregnancy test occurred more than 28 days before baseline (Day 1), sampling will need to be repeated.

Eligible subjects will be allocated (by central randomization) to a vaccination schedule as described in Section 5.

The subjects will be vaccinated as described in Section 6, with prime vaccinations administered at Day 1 for all groups, and boost vaccinations at Day 15 (Part 1 and Part 2, Group 2), or Day 29 (Part 2, Group 1) unless any of the pre-specified criteria not to proceed with vaccination are met (refer to Sections 6.2, 6.3 and 10.2 for details) or if a pause for vaccination of further subjects has been installed (see Section 9.3.2).

Prior to each vaccination, a urine pregnancy test (for female subjects of childbearing potential), a targeted physical examination and measurements of vital signs will be performed and blood will be drawn for safety, immunogenicity and virologic (only for HIV-infected subjects) assessments. Refer to Sections 9.3, 9.4 and 9.5 for further details on the safety, immunogenicity and virologic evaluations.

Study vaccine will be prepared on-site by unblinded qualified study-site personnel not involved in any other study-related procedure who will place a blinding tape on the syringe to mask its content and send the vaccine to a blinded study vaccine administrator (see Definitions of Terms) for administration to the subject (see Section 14.3 for details). Refer to Section 6 for further details on dosage and administration and post-vaccination monitoring.

All adverse events, serious adverse events, IREs and pregnancies will be collected and documented on the CRF, together with the information on any concomitant medications.

Adherence to HAART will be assessed at every visit in HIV-infected subjects. For reporting of IREs, refer to Section 12.3.3.

Upon discharge from the site, subjects will be provided with a diary, a thermometer, and a ruler to measure and record local solicited adverse events and body temperature. Subjects will also record solicited local and systemic adverse events (reactogenicity) in the diary in the evening after vaccination and then daily for the next 7 days at approximately the same time each day. Diaries should be completed at home by the subject. For subjects having difficulty completing the diary independently, the sites will make arrangements to have the diary completed according to their local practice. Subjects will be instructed to contact the investigator immediately in case they experience an unsolicited adverse event (not listed on the diary card) or for any severe (grade 3) solicited adverse event (listed on the diary card).

In all parts, subjects will come to the site at 7 days after each vaccination as indicated in the Time and Events Schedule. The subject's diary will be reviewed by study-site personnel. The investigator will examine the injection site for occurrences of erythema, induration/swelling, pain/tenderness or itching at these visits in order to complete the relevant parts of the CRF. Blood samples will be taken for safety evaluations. In addition, vital signs will be assessed at the visits 7 days after the vaccination.

9.1.4. Post-boost Follow-up

In all groups, subjects will come to the site at 21 and 42 days after the boost vaccination as indicated in the Time and Events Schedule. The investigator will examine the injection site for occurrences of erythema, induration/swelling, pain/tenderness or itching and will assess vital signs at these visits in order to complete the relevant parts of the CRF.

All adverse events, serious adverse events, IREs and pregnancies will be collected and documented on the CRF during these visits, together with the information on any concomitant medications. Subjects will also have blood drawn for CD4+ cell count determination (HIV-infected subjects only) and immunogenicity and virologic (only for HIV-infected subjects) assessments at both visits as specified in the Time and Events Schedule. For reporting of IREs, refer to Section 12.3.3.

Subjects will be instructed to contact the investigator before the next visit (ie, on Day 195 or 209) if they experience any adverse event or intercurrent illness that they perceive as relevant and/or can be possibly related to study vaccine in their opinion.

When all subjects in a part have come to the site at 6 months after the boost vaccination, or discontinued earlier and the database for that part has been locked, that part will be unblinded. Subjects who received placebo will be contacted by the site to communicate that they have completed the study and no further follow-up is required. However, subjects who received placebo and reach the 1-year post-boost visit (Part 1 and Part 2, Group 2: Day 380; Part 2, Group 1: Day 394) prior to unblinding will be required to attend the 1-year post-boost visit. All subjects who received Ad26.ZEBOV and MVA-BN-Filo will continue the study with a visit 1 year after the boost vaccination.

Serious adverse event information and IREs will be collected until the end of the study. Concomitant therapies should be recorded up to the 42-day post-boost visit and thereafter, concomitant therapies will be recorded only if given in conjunction with serious adverse events and IREs. Pregnancies will be reported until the end of the study. Adherence to HAART will be assessed at every visit in HIV-infected subjects. Subjects' vital signs will be assessed, a physical examination will be performed and blood will be drawn for CD4+ cell count determination (HIV-infected subjects only), immunogenicity and virologic (only HIV-infected subjects) assessments as specified in the Time and Events Schedule at the 6-month post-boost visit and 1-year post-boost visit (only for subjects on Ad26.ZEBOV and MVA-BN-Filo). Refer to Sections 9.3, 9.4 and 9.5 for further details on the CD4+ cell count determination, immunogenicity and virologic evaluations.

9.1.5. VAC52150 Vaccine Development Roll-over Study

Female subjects in Part 2 who became pregnant with estimated conception within 28 days after vaccination with MVA-BN-Filo (or placebo) or within 3 months after vaccination with Ad26.ZEBOV (or placebo) and children born to vaccinated female subjects in Part 2 who became pregnant with estimated conception within 28 days after vaccination with MVA-BN-Filo (or placebo) or within 3 months after vaccination with Ad26.ZEBOV (or placebo) will be eligible for enrollment into the VAC52150EBL4001 Vaccine Development Roll-over study for long-term surveillance (for a total of up to 60 months after the prime vaccination). After unblinding, only female subjects and the children born to female subjects (with estimated conception within the period stated above) in Part 2 who received Ad26.ZEBOV and/or MVA-BN-Filo will be approached to consent for enrollment in the VAC52150 Vaccine Development Roll-over study for long-term surveillance. After unblinding, female subjects and the children born to female subjects (with estimated conception within the period stated above) in Part 2 who received placebo and had already been enrolled into the VAC52150 Vaccine Development Roll-over study will be discontinued from further participation in the roll-over study.

As of amendment 4 implementation, no subjects in Part 1 will be approached anymore in this study to roll-over to the VAC52150EBL4001 study.

9.2. Endpoints

9.2.1. Primary Endpoints

The primary endpoints are:

- Safety as determined by:
  - Adverse events, collected until the 42-day post-boost visit.
  - Serious adverse events and IREs, collected until the end of the study.
  - Solicited local and systemic adverse events (reactogenicity), collected until 7 days after each vaccination.

- Magnitude of humoral immune response against the EBOV GP by determining binding antibody levels using ELISA (in ELISA units/mL and/or endpoint-titers) until the 21-day post-boost visit.

9.2.2. Secondary Endpoint

Comparison of safety and tolerability of Ad26.ZEBOV/MVA-BN-Filo and MVA-BN-Filo/Ad26.ZEBOV regimens between healthy and HIV-infected adults.

9.2.3. Exploratory Endpoints

Additional exploratory analyses may be performed to further investigate study vaccine-elicited immune responses. The exploratory endpoints might include but are not limited to the following:

- Magnitude, kinetics and durability of humoral immune responses to EBOV GP, including neutralizing antibody response measured in titers that inhibit viral infection by a certain percentage (IC_{50}, $_{80}$ or $_{90}$).
- Magnitude, kinetics and durability of cellular immune responses for EBOV GP (EBOV GP specific T cell responses will be measured as the number of IFN-γ producing cells by ELISpot analysis).
- Comparison between healthy and HIV-infected adults of the kinetics and durability of humoral and cellular immune responses to EBOV GP of Ad26.ZEBOV/MVA-BN-Filo and MVA-BN-Filo/Ad26.ZEBOV regimens.
- Kinetics and durability of humoral immune responses, including the development of neutralizing antibodies, in healthy and HIV-infected adults for other filovirus GP (binding antibody response measured in ELISA units/mL and/or endpoint-titers; neutralizing antibody response measured as titers that inhibit viral infection by a certain percentage [$IC_{50,\ 80\ \mathrm{or}\ 90}$]).
- Kinetics and durability of cellular immune responses in healthy and HIV-infected adults for other filovirus GP (EBOV GP specific T cell responses will be measured as the number of IFN-γ producing cells by ELISpot analysis or the percentage of CD4+ or CD8+ T cells producing IFN-γ, tumor necrosis factor- α (TNF-α) and/or interleukin (IL)-2 by intracellular cytokine staining [ICS]).
- Comparison of kinetics and durability of humoral and cellular immune responses to the other filovirus GP, of Ad26.ZEBOV/MVA-BN-Filo and MVA-BN-Filo/Ad26.ZEBOV regimens between healthy and HIV-infected adults.
- Comparison of kinetics and durability of humoral and cellular immune responses to the EBOV other filovirus GP of Ad26.ZEBOV/MVA-BN-Filo and MVA-BN-Filo/Ad26.ZEBOV regimens between adults aged 18-50 and aged 51-70.
- Frequency, magnitude, and durability of anti-vector responses in healthy and HIV-infected adults (measured by ELISA, virus neutralization assay [VNA] and/or plaque reduction neutralization test [PRNT].
- Time course of CD4+ count in HIV-infected adults.

- Changes in HIV RNA and HIV specific immune response (using HIV antigens in place of Ebola GP) associated with vaccination with Ad26.ZEBOV/MVA-BN-Filo and MVA-BN-Filo/Ad26.ZEBOV regimens in HIV-infected adults.
- Humoral immune responses in genital, rectal and oral secretions in a subset of healthy and HIV-infected adults for EBOV and other filovirus GP at selected sites.
- Impact of host genetics on immune responses to the Ad26.ZEBOV/MVA-BN-Filo and MVA-BN-Filo/Ad26.ZEBOV prime-boost regimens in healthy and HIV-infected adults.
- Immune epitope breadth elicited by the Ad26.ZEBOV/MVA-BN-Filo and MVA-BN-Filo/Ad26.ZEBOV prime-boost regimens in healthy and HIV-infected adults.
- Immunoglobulin subclass, glycosylation and effector functions of humoral responses to EBOV and other filovirus GP.
- B cell, helper T cell, and cytotoxic T cell responses elicited by the Ad26.ZEBOV/MVA-BN-Filo and MVA-BN-Filo/Ad26.ZEBOV prime-boost regimens in healthy and HIV-infected adults.
- Immune inflammatory responses elicited by the Ad26.ZEBOV/MVA-BN-Filo and MVA-BN-Filo/Ad26.ZEBOV regimens in healthy and HIV-infected adults on a multiplex array platform to evaluate cytokine and soluble factor responses to vaccination.

9.3. Safety Evaluations

9.3.1. Safety Assessments

The study will include the following evaluations of safety and tolerability as described below and according to the time points provided in the Time and Events Schedule. Any clinically significant abnormalities occurring from signing of the ICF onwards until 42 days after the boost vaccination must be recorded on the Adverse Event section of the CRF. Thereafter, reporting will be limited to all serious adverse events and IREs. Any clinically significant abnormalities (including those persisting at the end of the study/early withdrawal) will be followed by the investigator until resolution or until a clinically stable endpoint is reached (see Section 12).

The investigators, together with the sponsor's study responsible physician, will be responsible for the safety monitoring of the study, and will halt vaccination of further subjects in case any of the pre-specified pausing rules described in Section 9.3.2 have been met or in case of other severe safety concerns. Further safety measures with regards to vaccination are described in Sections 6.2 and 6.3.

An IDMC will be appointed by the sponsor before the start of the study to perform regular review of the safety data during the study. Details regarding the IDMC are provided in Section 11.7.

Adverse Events

All adverse events, whether serious or non-serious, will be collected at all visits from signing of the ICF onwards until 42 days post-boost. Thereafter, reporting will be limited to all serious adverse events and IREs up to the subject’s last study-related procedure. Solicited local and

systemic adverse events (reactogenicity, see below) will be reported by the subject until 7 days after each administration of study vaccine. Adverse events will be followed by the investigator as specified in Section 12.

Additional blood may be drawn to confirm and follow up on adverse events.

Solicited Adverse Events

Solicited adverse events (see Definitions of Terms) are precisely defined events that subjects are specifically asked about and which are noted by subjects in the diary. The subjects will be closely observed by study-site personnel for 30 (±10) minutes after each vaccination to monitor for the development of any acute reactions, or longer if deemed necessary by the investigator (eg, in case of grade 3 adverse events). Any unsolicited, solicited local or systemic adverse events occurring while on site will be documented. Upon discharge from the site, subjects will receive a diary, a thermometer and a ruler to measure body temperature and solicited local reactions. Subjects will be instructed to record solicited local and systemic adverse events in the diary in the evening after each administration of study vaccine and then daily for the next 7 days (until Day 8) at approximately the same time each day to serve as a reminder to the subject for the next visit. On Day 8, the diary will be reviewed on site before the subject leaves the site. The investigator should discuss the information from the diary with the subject, document the relevant information in the clinic chart, and complete the relevant parts of the CRF as described in the CRF Completion Guidelines.

On-site and diary reported solicited adverse events will be captured on a separate CRF page as described in the CRF Completion Guidelines, in contrast to the unsolicited adverse events which will be reported on the Adverse Event page of the CRF. The investigator must record in the CRF his/her opinion concerning the relationship of the adverse event to study vaccine.

Solicited Local (Injection Site) Adverse Events

Subjects will also be instructed on how to note occurrences of erythema, induration/swelling (measured using the ruler supplied), pain/tenderness and itching at the injection site in the evening after each administration of study vaccine and then daily for the next 7 days in the diary at approximately the same time each day.

Solicited Systemic Adverse Events

Subjects will be instructed on how to record daily body temperature using a thermometer provided for home use. Subjects should record the body temperature in the evening after each vaccination and then daily for the next 7 days in the diary at approximately the same time each day. If >1 measurement is made on any given day, the highest value will be recorded in the CRF.

Subjects will also be instructed on how to note the following symptoms in the evening after each administration of study vaccine and then daily for the next 7 days in the diary at approximately the same time each day:

- Nausea/vomiting
- Headache
- Myalgia
- Arthralgia
- Fatigue/malaise
- Chills
- Fever

If a ***solicited local or systemic adverse event*** is not resolved at Day 8, the follow-up will be captured on the diary. The subject will be instructed to record the date of last symptoms and maximum severity in the diary after resolution.

Suspected Cardiac Events

In case of a cardiac event, an ECG will be obtained and the subject will undergo cardiac enzyme testing and/or be referred to a local cardiologist as clinically indicated.

Clinical Laboratory Tests

Blood samples for serum chemistry and hematology, and for other tests (see below) and a urine sample for urinalysis will be collected at the time points indicated in the Time and Events Schedule. The investigator must review the laboratory results, document this review, and record any clinically relevant changes occurring during the study in the Adverse Event section of the CRF up to the 42-day post-boost visit. Thereafter, only serious adverse events and IREs need to be recorded. The laboratory reports must be filed with the source documents. For reporting of IREs, refer to Section 12.3.3.

Approximate blood volume expected to be drawn per visit for clinical laboratory assessments:

- 13 mL at screening
- 7 mL at other visits

Note: for HIV-infected subjects, an additional 4 mL will be taken for monitoring of CD4+ cell count at time points indicated in the Time and Events Schedule.

The following tests will be performed by the local laboratory, unless otherwise specified:

- Hematology Panel
  - hemoglobin
  - hematocrit
  - red blood cell count
  - white blood cell count with differential
  - platelet count
- Serum Chemistry Panel
  - alanine aminotransferase
  - creatinine

- Urinalysis – Dipstick
  - specific gravity
  - pH
  - glucose
  - protein
  - blood
  - ketones

  In case of positive urinalysis dipstick results for >1+ protein or blood, the sediment will be examined microscopically (only red blood cells (RBC) will be documented).

Additional clinical laboratory assessments to be performed are as follows:

- Urine pregnancy test for female subjects of childbearing potential at screening and prior to each study vaccination.
- CD4+ cell count for HIV-infected subjects at screening, on Day 1, on the day of the boost vaccination, at 21 and 42 days after the boost vaccination and at 6 months and 1 year after the boost vaccination.
- Serology: HIV type 1 or HIV type 1/2 at screening.

Laboratory abnormalities will be determined according to the Toxicity Table for Use in Trials Enrolling Healthy Adults (Attachment 2).

Electrocardiogram

A single, 12-lead ECG will be performed at screening for subjects >50 years and interpreted locally. Additional ECG monitoring may be done at other time points during the study if clinically indicated based on signs and symptoms.

During the collection of ECGs, subjects should be in a quiet setting without distractions (eg, television, cell phones). Subjects should rest for at least 5 minutes before ECG collection and should refrain from talking or moving arms or legs.

Vital Signs (blood pressure, pulse/heart rate, body temperature)

Vital sign measurements will be performed at the time points indicated in the Time and Events Schedule. Blood pressure and pulse/heart rate measurements will be assessed (at rest) with a completely automated device. Manual techniques will be used only if an automated device is not available. Confirmatory measurements can be performed if inconsistent with a prior measurement.

Either oral or axillary body temperatures may be measured and the site of measurement must be captured in the CRF.

Physical Examination

A full physical examination including height and body weight will be performed at screening. Calculation of the body mass index (BMI), will be recorded in the CRF. At other visits, an abbreviated, symptom-directed physical examination will be performed as indicated based on any clinically relevant issues, clinically relevant symptoms and medical history. The symptom-directed physical examination may be repeated if deemed necessary by the investigator. Physical examinations will be performed by the investigator or by a designated medically-trained clinician.

A full physical examination includes the following: general appearance, eyes, ears, nose, throat, cardiovascular system, respiratory system, gastrointestinal system, and skin and mucous membranes. A genitourinary exam is not required. A neurological and musculoskeletal examination as well as an examination of the lymph nodes will also be performed. An abbreviated, symptom-directed physical examination includes examination of the injection site(s), heart, lungs and abdomen.

The height should be measured at the screening visit. To obtain the actual body weight, subjects should be weighed without bulky outer clothing.

9.3.2. Pausing Rules

The investigators and the sponsor's study responsible physician will review the safety of enrolled subjects on an ongoing basis. The sponsor's study responsible physician will be involved in all discussions and decisions.

If any of the following events occur in any subject in any part who received at least 1 dose of study vaccine in the study (at any site), the site investigator will halt the vaccination of further subjects and the sponsor's study responsible physician will be notified immediately. The sponsor's study responsible physician will inform all the other investigators to halt further vaccination as well.

- Death in any subject, considered at least possibly related to the study vaccine *OR*
- An anaphylactic reaction within 24 hours of vaccination or the presence of generalized urticaria within 72 hours of vaccination in any subject, considered at least possibly related to the study vaccine *OR*
- A life-threatening or other serious adverse event in any subject, considered at least possibly related to the study vaccine

If any of the following events occur in any subject who received at least 1 dose of study vaccine in the study (across all sites), the sponsor's study responsible physician will notify all investigators to halt vaccination of further subjects:

- Three or more subjects experience a severe (grade 3) (non-serious) unsolicited adverse event (of the same type) considered to be related to any of the study vaccines that persists for 3 or more days *OR*

- Three or more subjects experience a persistent (upon repeat testing) severe (grade 3) (non-serious) abnormality related to the same laboratory parameter and considered to be related to any of the study vaccines *OR*
- Three or more subjects experience the same severe (grade 3) (non-serious) solicited systemic reaction considered to be related to any of the study vaccines that persists for 3 or more days (subjective systemic reaction corroborated by study personnel).

In case of occurrence of any of the events described above, the sponsor's study responsible physician will notify the IDMC immediately. Dosing will be halted and all applicable health authorities will be notified. Within 3 business days, the IDMC will convene to review the available safety data as outlined in the charter and to discuss study suspension or discontinuation of further vaccination or to decide that vaccination may resume. The sites will be allowed to resume activities upon receipt of a written notification from the sponsor. The criteria for pausing will be re-set each time and the same criteria have to be met again to halt further vaccination. The communications from the IDMC will be forwarded by the investigator to the Independent Ethics Committee/ Institutional Review Board (IEC/IRB) according to local standards and regulations and by the sponsor to relevant health authorities.

9.4. Immunogenicity Evaluations

Venous blood samples will be collected for the determination of immune responses at the time points indicated in the Time and Events Schedule. Samples for assessment of humoral and cellular immune responses will be taken from all subjects; a list of assessments can be found in Table 3. At selected sites, genital, rectal and oral secretions will be taken for assessment in a subset of subjects of humoral immune responses for EBOV, SUDV and MARV GP, dependent on assay availability.

Sample collection and processing will be performed by the study-site personnel according to current versions of approved standard operating procedures. The laboratory manual contains further details regarding the collection, handling, labeling, and shipment of blood samples and secretions to the respective laboratories.

Approximate blood volume expected to be drawn per visit for immunogenicity evaluations:

- 96 mL

The immunologic assessments and purposes are summarized in Table 3. The exploratory assay package may include, but might not be limited to, the listed assays.

Table 3: Summary of Immunologic Assessments in Serum and PBMC

Sample	Purpose (non-exhaustive)
Serum	- Analysis of antibodies binding to EBOV GP, possibly using different EBOV GPs and/or other filovirus GP (ELISA)<br>- Analysis of neutralizing antibodies to EBOV GP, possibly using different EBOV GPs and/or other filovirus GP (VNA)<br>- Analysis of binding and/or neutralizing antibodies to adenovirus and/or MVA (ELISA and/or neutralization test)<br>- Analysis of anti-EBOV GP, or other filovirus GP antibody characteristics and functionality, including IgG subtyping (molecular antibody characterization)<br>- In HIV-infected subjects, analysis of antibodies to HIV antigens (ELISA and/or neutralization assays)
PBMC	- Analysis of T cell IFN-γ responses to EBOV GP and possibly other filovirus GP (ELISpot)<br>- Analysis of T cell responses to EBOV GP, and/or other filovirus GP (including CD4/8, IL-2, IFN-γ, TNF-α and/or activation markers) (ICS)<br>- In HIV-infected subjects: analysis of T cell IFN-γ responses to HIV antigens (ELISpot)<br>- In HIV-infected subjects: analysis of T cell responses to HIV antigens (including CD4/8, IL-2, IFN-γ, TNF-α and/or activation markers) (ICS)

IgG: immunoglobulin G; ICS: intracellular cytokine staining; IFN-γ: interferon-γ; IL: interleukin; TNF: tumor necrosis factor; VNA: virus neutralization assay

9.5. Virologic Evaluations

Venous blood samples will be collected from HIV-infected subjects for the determination of HIV viral load (assayed by RNA polymerase chain reaction [PCR]) at the time points indicated in the Time and Events Schedule. Viral sequencing may be performed on these samples if the HIV viral load is sufficiently high.

In addition, stored serum or plasma of HIV-infected subjects may be used to assess antiretroviral drug levels.

Sample collection and processing will be performed by the study-site personnel according to current versions of approved standard operating procedures. The laboratory manual contains further details regarding the collection, handling, labeling, and shipment of blood samples to the respective laboratories.

Approximate blood volume expected to be drawn per visit for viral load determination and sequencing:

- 6 mL at screening, 4 mL at other visits

9.6. Pharmacogenomic (DNA) Evaluations

DNA samples will be analyzed for human leukocyte antigen (HLA) type determination (where local regulations permit) to explore the impact of host genetics on immune responses to the Ad26.ZEBOV/MVA-BN-Filo and MVA-BN-Filo/Ad26.ZEBOV prime-boost regimens. No additional venous blood samples need to be collected for this evaluation, samples collected on the day of the prime vaccination will be used. Subject participation in the pharmacogenomic research is optional and will require additional consent.

9.7. Vaccine-induced Seropositivity

In general, uninfected subjects who participate in Ebola vaccine studies may develop Ebola-specific antibodies as a result of an immune response to the candidate Ebola vaccine, referred to as VISP. These antibodies may be detected in Ebola serologic tests, causing the test to appear positive even in the absence of actual Ebola infection. VISP may become evident during the study, or after the study has been completed. The potential of a study participant becoming PCR-positive after vaccination is being assessed in a Phase 1 study (VAC52150EBL1002).

Subjects should not donate blood within 8 weeks before Day 1 or plan to donate blood until 42-days post-boost (see Section 4.2.1).

Subjects will be provided with a letter describing the study if requested by the subject. Subjects participating in the study will be provided with a "site information card" and instructed to carry this card with them for the duration of the study (see Section 12.3.1).

9.8. Sample Collection and Handling

The actual dates and times of sample collection must be recorded in the CRF or laboratory requisition form.

Refer to the Time and Events Schedule for the timing and frequency of all sample collections.

Instructions for the collection, handling, storage, and shipment of samples are found in the laboratory manual that will be provided. Collection, handling, storage, and shipment of samples must be under the specified, and where applicable, controlled temperature conditions as indicated in the laboratory manual.

9.9. Optional Procedures

Subjects will have the option of participating in separately consented collections and procedures in addition to the main protocol procedures as detailed below. Participation of sites in these collection and procedures varies across sites, and is detailed in each site-specific addendum. If any of the procedures provides evidence of an abnormal finding, the participant will be referred for diagnosis and care. Specifics on referral are provided in the site-specific addendum.

9.9.1. Apheresis for Part 1 Subjects

Up to 20 subjects (10 HIV-infected, 10 healthy) from Part 1 will be enrolled for optional apheresis for collection of PBMC and plasma on the Day 36 and Day 57 visits, given they have provided specific consent for this procedure. Up to a total of 10 Part 1 subjects (irrespective of HIV status) with robust immune responses post-boost may also be enrolled for an optional apheresis procedure performed after unblinding at a predefined visits or at an unscheduled visit for better characterization of immune responses. PBMCs and plasma will be obtained using automated apheresis techniques conducted by a qualified apheresis nurse/technician. Approximately 25 mL of red blood cell volume will be lost as residual loss in the machine during the procedure.

9.9.2. Mucosal Secretions for Part 2 Subjects

Collection of genital, rectal and oral secretion samples will be offered at Part 2 sites (Kenya, Tanzania, and Uganda) at time points indicated in the Time and Events Schedule.

9.9.2.1. Collection of Genital and Rectal Secretion Samples

Female Subjects

Consenting women will be instructed on how to use a Softcup device to collect cervical and vaginal secretions. The cup will remain in place for 4-12 hours. Pregnant women and those with a history of toxic shock syndrome will be excluded. Date of last menstrual period will be recorded at each collection visit. Collection will not take place if the woman is menstruating or has symptoms of active inflammation or infection of the vagina or cervix. Rectal sponge secretions will also be collected however, will be deferred if there are signs or symptoms of perianal inflammation. Subjects may participate in cervicovaginal secretion collection if they choose to defer rectal sponge secretion collections.

Male Subjects

Consenting men will be asked to ejaculate into a sterile container for semen collection. Rectal sponge secretions will also be collected. Semen and rectal sponge collections will be deferred from men if there are signs or symptoms of urethral or perianal inflammation, respectively. Subjects may participate in semen collection if they choose to defer rectal sponge secretion collections.

9.9.2.2. Collection of Oral Secretion Samples

Consenting men and women will be instructed to provide oral secretion samples by expectorating into a cup.

10. SUBJECT COMPLETION/DISCONTINUATION OF STUDY VACCINE/WITHDRAWAL FROM THE STUDY

10.1. Completion

A subject will be considered to have completed the study if he or she has completed all assessments at the 6-month post-boost visit for subjects who received placebo or at the 1-year post-boost visit for subjects who received Ad26.ZEBOV and MVA-BN-Filo.

10.2. Discontinuation of Study Vaccine/Withdrawal From the Study

Discontinuation of Study Vaccine

If a subject's study vaccine must be discontinued before the end of the vaccination schedule, this will not result in automatic withdrawal of the subject from the study.

A subject’s study vaccine (prime or boost) must be discontinued at the discretion of the investigator and after consultation with the sponsor for any of the events in Section 6.2.

A subject's study vaccine should be **permanently** discontinued if:

- The investigator believes that for safety reasons (eg, adverse event) it is in the best interest of the subject to discontinue study vaccine
- The subject becomes pregnant
- The subject has confirmed Ebola virus disease through natural exposure to the virus (eg, by travel to an affected country)
- The subject experiences any of the events described in Section 6.3
- The randomization code is broken by the investigator or the study-site personnel

Subjects meeting any of the reasons listed above must not receive any further study vaccine, but should continue to be monitored for safety and for immunogenicity according to the protocol if this does not result in safety risks for the subject. In case of early discontinuation of study vaccine due to an adverse event, the investigator will collect all information relevant to the adverse event and safety of the subject, and will follow the subject to resolution, or until reaching a clinically stable endpoint.

Withdrawal From the Study

Each subject has the right to withdraw from the study at any time for whatever reason. The investigator should make an attempt to contact subjects who did not return for scheduled visits or follow-up. Although the subject is not obliged to give reason(s) for withdrawing early, the investigator should make a reasonable effort to ascertain the reason(s) while fully respecting the subject's rights. The measures taken to follow up must be documented.

A subject will be withdrawn from the study for any of the following reasons:

- Decision by the investigator to withdraw a subject for repeated failure to comply with protocol requirements
- Decision by the sponsor to stop or cancel the study
- Decision by local regulatory authorities and IEC/IRB to stop or cancel the study
- Lost to follow-up
- Withdrawal of consent
- Death
- Receipt of blood products or immunoglobulin during the study.

If a subject withdraws early from the study for any of the reasons listed above (except in case of death), early withdrawal assessments (ie, physical examination, vital signs, CD4+ cell count [as applicable], [serious] adverse events and concomitant medications review, immunogenicity, and virologic assessments) should be obtained. A subject who wishes to withdraw consent from participation in the study will be offered an optional visit for safety follow-up (before formal withdrawal of consent), but the subject has the right to refuse.

When a subject withdraws before completing the study, the reason for withdrawal is to be documented in the CRF and in the source document. Study vaccine assigned to the withdrawn subject may not be assigned to another subject. For subjects who withdraw from the study after randomization but before the prime vaccination, an additional subject will be enrolled who will receive the same vaccination regimen as the withdrawn subject. No additional subject will be enrolled in case a subject withdraws from the study after receiving the prime vaccination.

10.3. Withdrawal From the Use of Research Samples

A subject who withdraws from the study will have the following options for storage of samples for potential future use:

- The collected samples will be retained and used in accordance with the subject's original informed consent for storage of samples for future use.
- The subject may withdraw consent for storage of samples for potential future use (see Section 16.2.6), in which case the samples will be destroyed and no further testing will take place. To initiate the sample destruction process, the investigator must notify the sponsor study site contact of withdrawal of consent for the storage of leftover samples for future research and request sample destruction. The sponsor study site contact will, in turn, contact the biomarker representative to execute sample destruction. If requested, the investigator will receive written confirmation from the sponsor that the samples have been destroyed. Details of the sample retention for research are presented in the ICF.

11. STATISTICAL METHODS

Statistical analysis will be done by the sponsor or under the authority of the sponsor. A general description of the analytic approach for the safety and immunogenicity data is outlined below. Specific details on methods will be provided in the Statistical Analysis Plan (SAP).

Interim analyses may be performed as described in Section 11.6.

The final analysis will be performed when all subjects have completed the last study-related visit or discontinued earlier.

A general description of the statistical methods to be used to analyze the safety and immunogenicity data is outlined below.

11.1. Sample Size Determination

An overall planned sample size of 575 subjects includes 460 subjects who will receive active prime-boost vaccination to substantially contribute to an overall safety database of the Ad26.ZEBOV and MVA-BN-Filo prime-boost regimen.

In Part 1, a total of 60 subjects (40 healthy and 20 HIV-infected) are planned to be vaccinated with MVA-BN-Filo prime vaccination followed by Ad26.ZEBOV 14 days later.

In Part 2, Group 1, a total of 320 subjects (160 healthy and 160 HIV-infected) will be vaccinated with Ad26.ZEBOV on Day 1, followed by MVA-BN-Filo 28 days later and in Group 2, a total of

80 subjects (40 healthy and 40 HIV-infected) will be given MVA-BN-Filo prime vaccination followed by Ad26.ZEBOV 14 days later.

The sample size is based on the probability of observing an adverse event given the true underlying incidence and is described in Table 4. In case a specific adverse event is not observed, the one-sided 97.5% upper confidence limit of the true incidence rate of this adverse event is less than 16.8%, 8.8%, 6.0%, 4.5%, 2.3%, 1.5%, 1.1%, and 0.8% for a total of 20, 40, 60, 80, 160, 240, 320, and 460 subjects on Ad26.ZEBOV and MVA-BN-Filo, respectively. Note that this does include some scenarios based on combining the data from Part 1 and Part 2. An analysis that combines data from Part 1 and Part 2 is appropriate for estimating safety parameters although any formal comparisons between vaccination regimens or HIV cohorts would likely only consider data from Part 2 of the study.

Table 4: Probability of Observing at Least One Adverse Event Given a True Adverse Event Incidence

True Adverse Event Incidence	N=20	N=40	N=60	N=80	N=160	N=240	N=320	N=460
0.1%	2.0%	3.9%	5.8%	7.7%	14.8%	21.4%	27.4%	36.9%
0.5%	9.5%	18.2%	26.0%	33.0%	55.2%	70.0	79.9%	90.0%
1%	18.2%	33.1%	45.3%	55.3%	80.0	91.0%	96.0%	99.0%
2.5%	39.7%	63.7%	78.1%	86.8%	98.3%	99.8%	100%	100%
5%	64.2%	87.2%	95.4%	98.4%	100%	100%	100%	100%
10%	87.8%	98.5%	99.8%	100%	100%	100%	100%	100%

Table 5 below shows the detectable differences for a 2-sided 5% level t-test with 90% power for comparing immune response (ELISA antibody levels against EBOV GP) at Day 42 post-boost. The 4 scenarios provided focus on the likely comparisons of interest (healthy versus HIV-infected within each vaccination regimen, across vaccination regimens regardless of HIV status, and HIV status exclusive of vaccination regimen).

Table 5: Magnitude of Pairwise Difference to be Detected Between Vaccine Regimens for a Given Sample Size

Variability ELISA antibody levels (SD, $\log_{10}$ scale)	Detectable difference, $\log_{10}$ scale *			
	n1=160, n2=40	n1=320, n2=80	n1=160, n2=160	n1=40, n2=40
0.3	0.18	0.12	0.11	0.22
0.4	0.23	0.16	0.15	0.29
0.5	0.29	0.2	0.18	0.37

SD: standard deviation
* 90% power, 5% significant level for 2-sided hypothesis

As the study has adequate power to detect differences in active vaccination regimens, the study will also likely be suitably powered to detect differences between active and placebo subjects.

11.2. Analysis Sets

The Full Analysis set includes all subjects who were randomized and received at least 1 dose of study vaccine, regardless of the occurrence of protocol deviations. Safety data will be analyzed based on the Full Analysis set.

The Immunogenicity Analysis set includes all randomized and vaccinated subjects, who have data from baseline and at least 1 post-vaccination immunogenicity blood draw.

The Per Protocol Analysis set includes all randomized and vaccinated subjects, who received both the prime and boost vaccinations (administered not more than 10 days outside the visit window), have immunogenicity data from baseline and at least 1 post-vaccination evaluable immunogenicity sample, and have no major protocol violations influencing the immune response.

11.3. Subject Information

For all subjects, demographic characteristics (eg, age, height, weight, BMI, race, and sex) and screening/baseline characteristics (eg, physical examination, medical history) will be tabulated and summarized with descriptive statistics.

11.4. Safety Analyses

The primary safety endpoint in Part 1 and Part 2 are Adverse events, collected until the 42-day post-boost visit, serious adverse events and IREs, collected until the end of the study and solicited local and systemic adverse events (reactogenicity), collected until 7 days after each vaccination. Safety analyses will be conducted during the planned interim analyses, primary analysis and final analysis. In addition, safety analyses may be conducted during ad hoc interim analyses. For more details about the interim and final analyses, see Sections 11 and 11.6.

Safety data will be analyzed descriptively (including 95% confidence intervals, if applicable). In general, the primary safety plan will analyze each part separately and secondarily analyze the combined safety data from Part 1 with Part 2, Group 2 (MVA prime/Ad26 boost 0, 14-day regimen). In detail, safety data will be separately described by part, HIV infection status, and vaccination regimen (in Part 2 only). For purposes of estimating rates and confidence intervals, various combinations of those factors will be considered (including collapsing across parts for analyzing the adverse event profile of the MVA/Ad26 regimen).

Baseline for all safety parameters will be defined as the last value before the prime vaccination.

Exploratory statistical analyses may be considered comparing safety endpoints (such as proportion of subjects experiencing systemic reactogenicity) between active and placebo subjects within part, group and cohort or across groups and cohorts among active subjects (eg, comparing rates between healthy and HIV-infected subjects with a similar vaccination regimen). Such comparisons will be described in the SAP and will be considered exploratory as the study is not definitively powered to detect small differences in safety rates between vaccination regimens or infection status or formally prove equivalence between regimens. Instead the purpose of such a

comparison would be to identify any potential safety issue which could then be formally assessed for equivalence in a future study or considered in a meta-analysis combining data across multiple studies.

Adverse Events (Including Reactogenicity)

The verbatim terms used in the CRF by investigators to report adverse events will be coded using the Medical Dictionary for Regulatory Activities (MedDRA). All reported adverse events (solicited local, solicited systemic, and unsolicited) will be included in the analysis. For each adverse event, the number and percentage of subjects who experience at least 1 occurrence of the given event will be summarized by group. Summaries, listings, datasets and/or subject narratives may be provided as appropriate, for those subjects who die, discontinue study vaccinations due to an adverse event, or experience a severe or serious adverse event. The analysis for solicited adverse events will be done on those subjects in the Full Analysis set for whom reactogenicity assessments are available in the database. The analysis of unsolicited adverse events will be done based on the Full Analysis set.

Physical Examination

Physical examination abnormalities will be listed. BMI will be calculated using the recording of height at screening.

Vital signs

Vital signs abnormalities will be tabulated by worst abnormality grade.

Clinical Laboratory Tests

Laboratory abnormalities will be determined in accordance with Toxicity Table for Use in Trials Enrolling Healthy Adults (Attachment 2). Laboratory abnormalities will be tabulated per treatment group by worst abnormality grade.

11.5. Immunogenicity Analyses

The primary immunogenicity endpoint in Part 1 and Part 2 is the Day 21 post-boost visit. Immunogenicity analyses will be conducted during the planned interim analyses and the final analysis. In addition, immunogenicity analyses may be conducted during ad hoc interim analyses. For more details about the interim and final analyses, see Sections 11 and 11.6. Within each part, the HIV-infected groups will be analyzed separately and in comparison with the healthy groups.

Descriptive statistics (actual values and changes from baseline, including 95% confidence intervals, if applicable) will be calculated for continuous immunologic parameters by time point. Graphical representations of changes in immunologic parameters will be prepared, as applicable. Frequency tabulations will be calculated for discrete (qualitative) immunologic parameters by time point. Response patterns over time for the immunologic parameters will be analyzed, taking into account within-subject correlations, to describe differences between the vaccination schedules at 21 days post-boost, 42 days post-boost, 6 months post-boost and 1 year post-boost.

Immunogenicity endpoints will be compared between treatment groups, in particular ELISA levels for Part 2. Immunogenicity data will be compared between vaccine groups for healthy and HIV-infected groups separately and combined. The immunogenicity data will also be compared between the healthy and HIV-infected groups within each vaccination group and across vaccination groups.

Details on the statistical analysis of the immunogenicity data will be provided in the SAP.

11.6. Interim Analyses

An interim analysis will be conducted when all subjects in Part 1 have completed the 21-day post-boost visit, or discontinued earlier. The purpose of this interim analysis is to evaluate the effect of vaccination on the HIV viral load of HIV-infected subjects from a safety perspective. This interim analysis will be reviewed by the PSRT and forwarded to the IDMC for review. An additional interim analysis will be done when all subjects in Part 1 have completed the 1-year post-boost visit or discontinued earlier.

Additional (ad hoc) interim analyses may be performed during the study for the purpose of informing future vaccine-related decisions in a timely manner. The results will not influence the conduct of the study in terms of early termination or later safety or immunogenicity endpoint assessments unless a significant safety concern has been identified.

A separate interim SAP will be prepared before the conduct of an interim analysis.

11.7. Independent Data Monitoring Committee

An IDMC will be established to monitor data on an ongoing basis to ensure the continuing safety of the subjects enrolled in this study. The committee will meet periodically to review interim data. Ad hoc IDMC meetings may be requested via the sponsor for any single event or combination of multiple events which are considered to jeopardize the safety of the subjects. After the review, the IDMC will make recommendations regarding the continuation of the study. The details will be provided in a separate IDMC charter.

The IDMC will be appointed by the sponsor with recommendations from the PSRT (see Section 11.8) before the start of the study. The IDMC will consist of at least 1 medical expert in the relevant therapeutic area and at least 1 statistician. The IDMC responsibilities, authorities, and procedures will be documented in its charter. Any safety reports/interim data from the parts in this study reviewed by the IDMC and the recommendations of the IDMC will be shared with the local health authorities and the IECs/IRBs.

11.8. Protocol Safety Review Team and Protocol Safety Review Team Reviews

The PSRT will review all adverse events (including reportable adverse events) on a regular and expedited basis as needed. In addition, the PSRT will review aggregate safety data reports from all sites on a regular basis as dictated by study progress. This team includes the following: Study Chair, site principal investigators (PIs), all Department of Defense (DoD) Research Monitors from each participating site, and the sponsor's study physician or their designees. Additional

participants could include but is not limited to associate investigators and senior clinical research nursing staff. A quorum is established with the Protocol Chair, 4 of 7 site PIs and the sponsor's study physician or their designee.

The PSRT will review summaries for the IDMC and make clarifications for the IDMC as needed.

12. ADVERSE EVENT REPORTING

Timely, accurate, and complete reporting and analysis of safety information from clinical studies are crucial for the protection of subjects, investigators, and the sponsor, and are mandated by regulatory agencies worldwide. The sponsor has established Standard Operating Procedures in conformity with regulatory requirements worldwide to ensure appropriate reporting of safety information; all clinical studies conducted by the sponsor or its affiliates will be conducted in accordance with those procedures.

12.1. Definitions

12.1.1. Adverse Event Definitions and Classifications

Adverse Event

An adverse event is any untoward medical occurrence in a clinical study subject administered a medicinal (investigational or non-investigational) product. An adverse event does not necessarily have a causal relationship with the treatment. An adverse event can therefore be any unfavorable and unintended sign (including an abnormal finding), symptom, or disease temporally associated with the use of a medicinal (investigational or non-investigational) product, whether or not related to that medicinal (investigational or non-investigational) product. (Definition per International Council for Harmonisation [ICH]).

This includes any occurrence that is new in onset or aggravated in severity or frequency from the baseline condition, or abnormal results of diagnostic procedures, including laboratory test abnormalities.

<u>Note</u>: *The sponsor collects adverse events starting with the signing of the ICF (refer to Section 12.3.1, for time of last adverse event recording).*

Serious Adverse Event

A serious adverse event based on ICH and EU Guidelines on Pharmacovigilance for Medicinal Products for Human Use is any untoward medical occurrence that at any dose:

- Results in death
- Is life-threatening (The subject was at risk of death at the time of the event. It does not refer to an event that hypothetically might have caused death if it were more severe.)
- Requires inpatient hospitalization or prolongation of existing hospitalization
- Results in persistent or significant disability/incapacity

- Is a congenital anomaly/birth defect
- Is a suspected transmission of any infectious agent via a medicinal product
- Is Medically Important*

*Medical and scientific judgment should be exercised in deciding whether expedited reporting is also appropriate in other situations, such as important medical events that may not be immediately life-threatening or result in death or hospitalization but may jeopardize the subject or may require intervention to prevent one of the other outcomes listed in the definition above. These should usually be considered serious.

If a serious and unexpected adverse event occurs for which there is evidence suggesting a causal relationship between the study vaccine and the event (eg, death from anaphylaxis), the event must be reported as a suspected unexpected serious adverse reaction (SUSAR) (even after the study is over, if the sponsor, IDMC or investigator becomes aware of them) by the sponsor to the applicable health authorities and by the investigator to the IEC/IRB according to regulatory and local requirements.

Unlisted (Unexpected) Adverse Event/Reference Safety Information

An adverse event is considered unlisted if the nature or severity is not consistent with the applicable product reference safety information. For Ad26.ZEBOV and MVA-BN-Filo, the expectedness of an adverse event will be determined by whether or not it is listed in the Investigator's Brochures and Addenda, if applicable.[11,12]

Immediate Reportable Events

The following list of neuroinflammatory disorders are categorized as IREs, and should be reported to the sponsor within 24 hours of becoming aware of the event using the IRE Form. Relevant data from the IRE Form will be captured in the clinical database.

- Cranial nerve disorders, including paralyses/paresis (eg, Bell’s palsy)
- Optic neuritis
- Multiple sclerosis
- Transverse myelitis
- Guillain-Barré syndrome, including Miller Fisher syndrome, Bickerstaff’s encephalitis and other variants
- Acute disseminated encephalomyelitis, including site-specific variants: eg, non-infectious encephalitis, encephalomyelitis, myelitis, myeloradiculomyelitis
- Myasthenia gravis and Lambert-Eaton myasthenic syndrome
- Immune-mediated peripheral neuropathies and plexopathies (including chronic inflammatory demyelinating polyneuropathy, multifocal motor neuropathy and polyneuropathies associated with monoclonal gammopathy)
- Narcolepsy

- Isolated paresthesia of >7 days duration

Symptoms, signs or conditions which might (or might not) represent the above diagnoses, should be recorded and reported as IREs even if the final or definitive diagnosis has not yet been determined, and alternative diagnoses have not yet been eliminated or shown to be less likely. Follow up information and final diagnoses, if applicable, should be submitted as soon as they become available.

If the IRE is also serious (serious adverse event), it will be reported using the same process as for other serious adverse events.

Note: IREs are to be reported from the signing of the ICF onwards until the end of the study.

Adverse Event Associated with the Use of the Study Vaccine

An adverse event is considered associated with the use of the study vaccine if the attribution is possibly, probably, or very likely by the definitions listed in Section 12.1.2, Attribution Definitions.

An adverse event is considered not associated with the use of the study vaccine if the attribution is not related or doubtful by the definitions listed in Section 12.1.2, Attribution Definitions.

12.1.2. Attribution Definitions

Every effort should be made by the investigator to explain any adverse event and to assess its potential causal relationship, ie, to administration of the study vaccine or to alternative causes, eg, natural history of underlying disease(s), concomitant drug(s). This applies to all adverse events, whether serious or non-serious. Assessment of causality must be done by a licensed study physician (the investigator or designee).

The investigator will use the following guidelines to assess the causal relationship of an adverse event to study vaccine:

Not Related

An adverse event that is not related to the use of the vaccine.

Doubtful

An adverse event for which an alternative explanation is more likely, eg, concomitant drug(s), concomitant disease(s), or the relationship in time suggests that a causal relationship is unlikely.

Possible

An adverse event that might be due to the use of the vaccine. An alternative explanation, eg, concomitant drug(s), concomitant disease(s), is inconclusive. The relationship in time is reasonable; therefore, the causal relationship cannot be excluded.

Probable

An adverse event that might be due to the use of the vaccine. The relationship in time is suggestive. An alternative explanation is less likely, eg, concomitant drug(s), concomitant disease(s).

Very Likely

An adverse event that is listed as a possible adverse reaction and cannot be reasonably explained by an alternative explanation, eg, concomitant drug(s), concomitant disease(s). The relationship in time is very suggestive.

12.1.3. Severity Criteria

Adverse events and laboratory data will be coded for severity using the toxicity grading tables in Attachment 2. For adverse events not identified in the table, the following guidelines will apply:

**Mild**	Grade 1	Symptoms causing no or minimal interference with usual social and functional activities
**Moderate**	Grade 2	Symptoms causing greater than minimal interference with usual social and functional activities
**Severe**	Grade 3	Symptoms causing inability to perform usual social and functional activities

Note: Only clinically significant abnormalities in laboratory data occurring from signing of the ICF onwards will be reported as adverse events and graded using the tables above.

12.2. Special Reporting Situations

Safety events of interest on a sponsor study vaccine that may require expedited reporting or safety evaluation include, but are not limited to:

- Administration of an overdose of study vaccine
- Accidental or occupational exposure to a study vaccine
- Administration error involving a study vaccine (with or without subject/patient exposure to the study vaccine, eg, name confusion)
- IREs

Special reporting situations should be recorded in the CRF. For reporting of IREs, refer to Section 12.3.3. Any special reporting situation that meets the criteria of a serious adverse event should be recorded on the Serious Adverse Event page of the CRF.

12.3. Procedures

12.3.1. All Adverse Events

All adverse events and special reporting situations, whether serious or non-serious, will be reported from the time a signed and dated ICF is obtained until 42 days post-boost, and serious

adverse events and IREs will be collected from signing of the ICF onwards until the end of the study. Subjects will be instructed to record solicited local and systemic adverse events (reactogenicity) in the diary in the evening after each vaccination and then daily for the next 7 days at approximately the same time each day.

Serious adverse events must be reported by the investigator using the Serious Adverse Event Form. SUSARs will be reported even after the study is over, if the sponsor, the IDMC or the investigator becomes aware of them. The sponsor will evaluate any safety information that is spontaneously reported by an investigator beyond the time frame specified in the protocol.

The investigator will monitor and analyze the study data including all adverse events and clinical laboratory data as they become available and will make determinations regarding the severity of the adverse experiences and their relation to study vaccine. All adverse events will be deemed related to study vaccine or not related to study vaccine, according to the terminology used in Section 12.1.2. To ensure that all adverse events are captured in a timely manner, the CRF will be entered in real-time and subjected to review to identify adverse events which may invoke pausing rules.

The investigator must review both post-injection reactogenicity and other adverse event CRF to insure the prompt and complete identification of all events that require expedited reporting as serious adverse events, invoke pausing rules or are other serious and unexpected events.

All adverse events, regardless of seriousness, severity, or presumed relationship to study vaccine, must be recorded using medical terminology in the source document and the CRF. Whenever possible, diagnoses should be given when signs and symptoms are due to a common etiology (eg, cough, runny nose, sneezing, sore throat, and head congestion should be reported as "upper respiratory infection"). Investigators must record in the CRF their opinion concerning the relationship of the adverse event to vaccine. All measures required for adverse event management must be recorded in the source document and reported according to sponsor instructions. For reporting of IREs, refer to Section 12.3.3.

The sponsor assumes responsibility for appropriate reporting of adverse events to the regulatory authorities. The sponsor will also report to the investigator (and the head of the investigational institute where required) all SUSARs. The investigator (or sponsor where required) must report SUSARs to the appropriate IEC/IRB that approved the protocol unless otherwise required and documented by the IEC/IRB. A SUSAR will be reported to regulatory authorities unblinded. Participating investigators and IEC/IRB will receive a blinded SUSAR summary, unless otherwise specified.

For all studies with an outpatient phase, including open-label studies, the subject must be provided with a "wallet (study) card" and instructed to carry this card with them for the duration of the study indicating the following:

- Study number

- Statement, in the local language(s), that the subject is participating in a clinical study and that results from Ebola virus serological screening tests should be interpreted with caution as the subject could have a false positive test (not truly infected with EBOV) as a result of an immune response to the candidate Ebola vaccine.
- Investigator's name and 24-hour contact telephone number
- Local sponsor's representative (name) and 24-hour contact telephone number (for medical staff only)
- Site number
- Subject number
- Any other information that is required to do an emergency breaking of the blind

12.3.2. Serious Adverse Events

All serious adverse events occurring during the study must be reported to the appropriate sponsor contact person by study-site personnel within 24 hours of their knowledge of the event.

Information regarding serious adverse events will be transmitted to the sponsor using the Serious Adverse Event Form, which must be completed and signed by a physician from the study site, and transmitted to the sponsor within 24 hours. The initial and follow-up reports of a serious adverse event should be made by facsimile (fax).

All serious adverse events that have not resolved by the end of the study, or that have not resolved upon discontinuation of the subject's participation in the study, must be followed until any of the following occurs:

- The event resolves
- The event stabilizes
- The event returns to baseline, if a baseline value/status is available
- The event can be attributed to agents other than the study vaccine or to factors unrelated to study conduct
- It becomes unlikely that any additional information can be obtained (subject or health care practitioner refusal to provide additional information, lost to follow-up after demonstration of due diligence with follow-up efforts)

Suspected transmission of an infectious agent by a medicinal product will be reported as a serious adverse event. Any event requiring hospitalization (or prolongation of hospitalization) that occurs during the course of a subject's participation in a study must be reported as a serious adverse event, except hospitalizations for the following:

- Hospitalizations not intended to treat an acute illness or adverse event (eg, social reasons such as pending placement in long-term care facility)
- Surgery or procedure planned before entry into the study (must be documented in the CRF).

Note: Hospitalizations that were planned before the signing of the ICF, and where the underlying condition for which the hospitalization was planned has not worsened, will not be considered serious adverse events. Any adverse event that results in a prolongation of the originally planned hospitalization is to be reported as a new serious adverse event.

The cause of death of a subject in a study, whether or not the event is expected or associated with the study vaccine, is considered a serious adverse event.

12.3.3. Immediate Reportable Events

One subject in the study VAC52150EBL2001 experienced a serious and very rare condition called "Miller Fisher syndrome". This condition consists of double vision, pain on moving the eye, and difficulty with balance while walking. Miller Fisher syndrome most commonly occurs following a recent infection. The subject experienced these symptoms about a week after suffering from a common cold and fever. The event happened about a month after boost vaccination with either MVA-BN-Filo or placebo. This subject had to go to the hospital for treatment and has recovered. After an extensive investigation, the event has been considered to be doubtfully related to vaccine and most likely related to the previous common cold.

Any events of neuroimmunologic significance (listed in Section 12.1.1) should be categorized as IREs and should be reported throughout the study (from signing of the ICF onwards until the end of the study) using the IRE Form provided **within 24 hours to the sponsor**. Events suggestive of the disorders considered IREs should be reported even if the final diagnosis has not been yet determined, and follow-up information and final diagnosis should be submitted to the sponsor as soon as they become available.

If an event meets serious adverse event criteria (see above), it should be documented as such using the Serious Adverse Event Form, as well as the relevant CRF Adverse Event page and the complete IRE Form page 3 to be included as part of the Serious Adverse Event report.

In addition, all serious adverse events that are considered related or possibly related, and all deaths, will be promptly (within 48 hours) reported to the WRAIR IRB and local IRB.

The principal investigator will then submit written reports within 10 working days to the WRAIR IRB. Follow-up reports will be submitted as additional information becomes available. A summary of the non-serious adverse events and serious adverse events (both related and unrelated) that occurred during the reporting period will be included in the continuing review report to the WRAIR IRB.

12.3.4. Pregnancy

Pregnancies will be reported from signing of the ICF until the end of the study.

All initial reports of pregnancy in female subjects or partners of male subjects must be reported to the sponsor by the study-site personnel within 24 hours of their knowledge of the event using the appropriate pregnancy notification form. Abnormal pregnancy outcomes (eg, spontaneous abortion, fetal death, stillbirth, congenital anomalies, ectopic pregnancy) are considered serious adverse events and must be reported using the Serious Adverse Event Form. Any subject who

becomes pregnant during the study must be promptly withdrawn from further study vaccination but should continue participation in the study for safety follow-up.

Because the effect of the study vaccine on sperm is unknown, pregnancies in partners of male subjects included in the study will be reported as noted above.

Follow-up information regarding the outcome of the pregnancy and any postnatal sequelae in the infant will be required to be sent to the sponsor.

The parent(s)/legal guardian of children born to vaccinated female subjects in Part 2 who become pregnant with estimated conception within 28 days after vaccination with MVA-BN-Filo (or placebo) or within 3 months after vaccination with Ad26.ZEBOV (or placebo), will also be approached to consent for enrollment of their offspring into the VAC52150EBL4001 Vaccine Development Roll-over study (see Section 9.1.5).

12.4. Contacting Sponsor Regarding Safety

The names (and corresponding telephone numbers) of the individuals who should be contacted regarding safety issues or questions regarding the study are listed in the Contact Information page(s), which will be provided as a separate document.

13. PRODUCT QUALITY COMPLAINT HANDLING

A product quality complaint (PQC) is defined as any suspicion of a product defect related to manufacturing, labeling, or packaging, ie, any dissatisfaction relative to the identity, quality, durability, or reliability of a product, including its labeling or package integrity. A PQC may have an impact on the safety and efficacy of the product. Timely, accurate, and complete reporting and analysis of PQC information from studies are crucial for the protection of subjects, investigators, and the sponsor, and are mandated by regulatory agencies worldwide. The sponsor has established procedures in conformity with regulatory requirements worldwide to ensure appropriate reporting of PQC information; all studies conducted by the sponsor or its affiliates will be conducted in accordance with those procedures.

13.1. Procedures

All initial PQCs must be reported to the sponsor by the study-site personnel within 24 hours after being made aware of the event.

If the defect is combined with a serious adverse event, the study-site personnel must report the PQC to the sponsor according to the serious adverse event reporting timelines (refer to Section 12.3.2). A sample of the suspected product should be maintained for further investigation if requested by the sponsor.

13.2. Contacting Sponsor Regarding Product Quality

The names (and corresponding telephone numbers) of the individuals who should be contacted regarding product quality issues are listed in the Contact Information page(s), which will be provided as a separate document.

14. STUDY VACCINE INFORMATION

14.1. Description of Study Vaccines

Ad26.ZEBOV

Ad26.ZEBOV is a monovalent, replication-incompetent Ad26-based vector that expresses the full length EBOV Mayinga GP and is produced in the human cell line PER.C6®.

The Ad26.ZEBOV vaccine will be supplied at a concentration of $1x10^{11}$ vp/mL in 2-mL single-use glass vials as a frozen liquid suspension to be thawed before use. Each vial contains an extractable volume of 0.5 mL. Refer to the Investigator's Brochure for a list of excipients.[11]

The drug substance used in Ad26.ZEBOV drug product is manufactured, according to the 2x10 L scale process, in Bern, Switzerland manufacturing facility.

MVA-BN-Filo

MVA-BN-Filo is a recombinant multivalent vaccine intended for active immunization against Ebola and Marburg virus infection. MVA-BN-Filo is strongly attenuated; the vaccine is propagated in primary chicken embryo fibroblast cells and does not replicate in human cells.

The MVA-BN-Filo vaccine is supplied at a concentration of $2x10^{8}$ Inf U/mL (nominal titer) in 2-mL glass vials as a frozen liquid suspension to be thawed before use. Each vial contains an extractable volume of 0.5 mL. Refer to the Investigator's Brochure for a list of excipients.[12]

The MVA-BN-Filo vaccine is manufactured by IDT Biologika GmbH for Janssen Vaccines & Prevention B.V., The Netherlands.

Placebo

The placebo supplied for this study will be formulated as a sterile 0.9% saline for injection (as commercially available).

14.2. Packaging and Labeling

All study vaccines will be manufactured and packaged in accordance with Good Manufacturing Practice (GMP). All study vaccines will be packaged and labeled under the responsibility of the sponsor. No study vaccine can be repacked or relabeled on site without prior approval from the sponsor.

Further details for study vaccine packaging and labeling can be found in the Site Investigational Product Procedures Manual.

14.3. Preparation, Handling, and Storage

Study vaccine must be stored at controlled temperatures: Ad26.ZEBOV vials must be stored at ≤-60°C and MVA-BN-Filo vials must be stored at ≤–20°C.

Vials must be stored in a secured location with no access for unauthorized personnel. All study product storage equipment (including refrigerators, freezers), must be equipped with a continuous temperature monitor and alarm, and with back-up power systems. In the event that study vaccine is exposed to temperatures outside the specified temperature ranges, all relevant data will be sent to the sponsor to determine if the affected study vaccine can be used or will be replaced. The affected study vaccine must be quarantined and not used until further instruction from the sponsor is received.

Blinding will be achieved by preparation of study vaccine by unblinded qualified study-site personnel not involved in any other study-related procedure, and by the administration of vaccine in a masked syringe by a blinded study vaccine administrator (see Definitions of Terms).

Details on the preparation, the holding time and storage conditions from the time of preparation to administration of Ad26.ZEBOV and MVA-BN-Filo are provided in the Site Investigational Product Procedures Manual.

14.4. Study Vaccine Accountability

The investigator is responsible for ensuring that all study vaccine received at the site is inventoried and accounted for throughout the study. The study vaccine administered to the subject must be documented on the study vaccine accountability form. All study vaccine will be stored and disposed of according to the sponsor's instructions. Study-site personnel must not combine contents of the study vaccine containers.

Study vaccine must be handled in strict accordance with the protocol and the container label, and must be stored at the study site in a limited-access area or in a locked cabinet under appropriate environmental conditions. Unused study vaccine must be available for verification by the sponsor's study site monitor during on-site monitoring visits. The return to the sponsor of unused study vaccine will be documented on the study vaccine return form. When the study site is an authorized destruction unit and study vaccine supplies are destroyed on-site, this must also be documented on the study vaccine return form.

Potentially hazardous materials such as used ampules, needles, syringes and vials containing hazardous liquids, should be disposed of immediately in a safe manner and therefore will not be retained for study vaccine accountability purposes.

Study vaccine should be dispensed under the supervision of the investigator or a qualified member of the study-site personnel, or by a qualified staff member. Study vaccine will be supplied only to subjects participating in the study. Returned study vaccine must not be dispensed again, even to the same subject. Study vaccine may not be relabeled or reassigned for use by other subjects. The investigator agrees neither to dispense the study vaccine from, nor store it at, any site other than the study sites agreed upon with the sponsor.

15. STUDY-SPECIFIC MATERIALS

The investigator will be provided with the following supplies:

- Investigator's Brochures and addenda (if applicable) for Ad26.ZEBOV and MVA-BN-Filo
- Site investigational product procedures manual
- Laboratory manual
- IWRS manual
- Electronic Data Capture (eDC) manual/electronic CRF completion guidelines and randomization instructions
- Sample ICF
- Subject diaries
- TOU
- Rulers, thermometers
- Site information card
- Recruitment tools, as applicable

16. ETHICAL ASPECTS

16.1. Study-specific Design Considerations

Potential subjects will be fully informed of the risks and requirements of the study and, during the study, subjects will be given any new information that may affect their decision to continue participation. They will be told that their consent to participate in the study is voluntary and may be withdrawn at any time with no reason given and without penalty or loss of benefits to which they would otherwise be entitled. Only subjects who are fully able to understand the risks, benefits, and potential adverse events of the study, and provide their consent voluntarily will be enrolled.

Approximately 571 to 675 mL of blood (including PBMC samples) will be drawn from adults over a period of around 1 year, and remains well below the limits of standard blood donation. Additional blood may be drawn to confirm and follow-up on adverse events.

Test of Understanding

The TOU is a short assessment of the subject's understanding of key aspects of the study (Attachment 1). The test will help the study staff to determine how well the subject understands the study and the requirements for participation.

The TOU must be completed after reading but before signing the ICF by all subjects (or as required per local regulations and practice, see Section 4), prior to enrollment into the study. The TOU is reviewed one-on-one with the subject and a member of the study team. Subjects are allowed to retake the test twice to achieve the passing score (≥90%) required for participation in the study. If a subject fails to achieve the passing score, further information and counseling will be provided by the study team member.

Any subject not capable of understanding the key aspects of the study, and their requirements for participation, should not be enrolled.

16.2. Regulatory Ethics Compliance

16.2.1. Investigator Responsibilities

The investigator is responsible for ensuring that the study is performed in accordance with the protocol, current ICH guidelines on Good Clinical Practice (GCP), and applicable regulatory and country-specific requirements.

GCP is an international ethical and scientific quality standard for designing, conducting, recording, and reporting studies that involve the participation of human subjects. Compliance with this standard provides public assurance that the rights, safety, and well-being of study subjects are protected, consistent with the principles that originated in the Declaration of Helsinki, and that the study data are credible.

16.2.2. Independent Ethics Committee or Institutional Review Board

Before the start of the study, the investigator (or sponsor where required) will provide the IEC/IRB with current and complete copies of the following documents (as required by local regulations):

- Final protocol and, if applicable, amendments
- Site-specific addendum
- Sponsor-approved ICF (and any other written materials to be provided to the subjects)
- Investigator's Brochure (or equivalent information) and amendments/addenda
- TOU
- Sponsor-approved subject recruiting materials
- Information on compensation for study-related injuries or payment to subjects for participation in the study, if applicable
- Investigator's curriculum vitae or equivalent information (unless not required, as documented by the IEC/IRB)
- Information regarding funding, name of the sponsor, institutional affiliations, other potential conflicts of interest, and incentives for subjects
- Any other documents that the IEC/IRB requests to fulfill its obligation

This study will be undertaken only after the IEC/IRB has given full approval of the final protocol, amendments (if any, excluding the ones that are purely administrative, with no consequences for subjects, data or study conduct, unless required locally), the ICF, applicable recruiting materials, and subject compensation programs, and the sponsor has received a copy of this approval. This approval letter must be dated and must clearly identify the IEC/IRB and the documents being approved.

During the study the investigator (or sponsor where required) will send the following documents and updates to the IEC/IRB for their review and approval, where appropriate:

- Protocol amendments (excluding the ones that are purely administrative, with no consequences for subjects, data or study conduct)
- Site-specific addendum amendments
- Revision(s) to ICF and any other written materials to be provided to subjects
- If applicable, new or revised subject recruiting materials approved by the sponsor
- Revisions to compensation for study-related injuries or payment to subjects for participation in the study, if applicable
- New edition(s) of the Investigator's Brochure and amendments/addenda
- Summaries of the status of the study at intervals stipulated in guidelines of the IEC/IRB (at least annually)
- Reports of adverse events that are serious, unlisted/unexpected, and associated with the study vaccine
- New information that may adversely affect the safety of the subjects or the conduct of the study
- Deviations from or changes to the protocol to eliminate immediate hazards to the subjects
- Report of deaths of subjects under the investigator's care
- Notification if a new investigator is responsible for the study at the site
- Development Safety Update Report and Line Listings, where applicable
- Any other requirements of the IEC/IRB

For all protocol amendments (excluding the ones that are purely administrative, with no consequences for subjects, data or study conduct), the amendment and applicable ICF revisions must be submitted promptly to the IEC/IRB for review and approval before implementation of the change(s).

At least once a year, the IEC/IRB will be asked to review and reapprove this study, where required.

At the end of the study, the investigator (or sponsor where required) will notify the IEC/IRB about the study completion.

16.2.3. DoD Research Monitor

Each site will identify a DoD Research Monitor. The role and qualifications of the DoD Research Monitor are identified as follows:

In their role as a full member of the PSRT, the DoD Research Monitor is required to review all unanticipated problems involving risk to volunteers or others, serious adverse events reports, and all volunteer deaths. The DoD Research Monitor will provide an unbiased written report of all

unanticipated problems involving risks to participants or others, and related serious adverse events and deaths occurring at their site to the sponsor and the WRAIR IRB. At a minimum, the research monitor should comment on the outcomes of the event or problem, and in the case of a death, comment on the relationship to participation in the study. The research monitor should also indicate whether he/she concurs with the details of the report provided by the study investigator. The research monitor will also in the context of the PSRT:

- Discuss research progress with the PI and study team, interview subjects at their site, consult on individual cases, or evaluate suspected adverse reaction reports on behalf of the WRAIR IRB.
- At the direction of the WRAIR IRB, be involved in oversight functions (eg, observe recruitment, enrollment procedures, and the consent process for individuals, groups or units; oversee study interventions and interactions; review monitoring plans and unanticipated problems involving risks to subjects or others (UPIRTSO) reports; and oversee data matching, data collection, and analysis).
- Promptly report discrepancies or problems to the WRAIR IRB.
- Have the authority, in the context of PSRT deliberations, to stop a research study in progress, remove individual subjects from a study, and take whatever steps are necessary to protect the safety and well-being of research subjects until the WRAIR IRB can assess the research monitor's report.

Per ICH, Janssen Vaccines & Prevention B.V. as the sponsor has ultimate responsibility for the safety and well-being of participating subjects.

16.2.4. Informed Consent

Each subject (or a legally acceptable representative) must give written consent according to local requirements after the nature of the study has been fully explained. The ICF(s) must be signed before performance of any study-related activity. The ICF(s) used must be approved by both the sponsor and by the reviewing IEC/IRB and be in a language that the subject can understand. The informed consent should be in accordance with principles that originated in the Declaration of Helsinki, current ICH and GCP guidelines, applicable regulatory requirements, and sponsor policy.

Before enrollment in the study, the investigator or an authorized member of the study-site personnel must explain to potential subjects or their legally acceptable representatives the aims, methods, reasonably anticipated benefits, and potential hazards of the study, and any discomfort participation in the study may entail. Subjects will be informed that their participation is voluntary and that they may withdraw consent to participate at any time. They will be informed that choosing not to participate will not affect the care the subject will receive. Finally, they will be told that the investigator will maintain a subject identification register for the purposes of long-term follow-up if needed and that their records may be accessed by health authorities and authorized sponsor personnel without violating the confidentiality of the subject, to the extent permitted by the applicable law(s) or regulations. By signing the ICF the subject or legally acceptable representative is authorizing such access. It also denotes that the subject agrees to

allow his or her study physician to recontact the subject for the purpose of obtaining consent for additional safety evaluations, if needed.

The subject or legally acceptable representative will be given sufficient time to read the ICF and the opportunity to ask questions. After this explanation and before entry into the study, consent should be appropriately recorded by means of either the subject's or his or her legally acceptable representative's personally dated signature. After having obtained the consent, a copy of the ICF must be given to the subject.

If the subject or legally acceptable representative is unable to read or write, an impartial witness should be present for the entire informed consent process (which includes reading and explaining all written information) and should personally date and sign the ICF after the oral consent of the subject or legally acceptable representative is obtained.

16.2.5. Privacy of Personal Data

The collection and processing of personal data from subjects enrolled in this study will be limited to those data that are necessary to fulfill the objectives of the study.

These data must be collected and processed with adequate precautions to ensure confidentiality and compliance with applicable data privacy protection laws and regulations. Appropriate technical and organizational measures to protect the personal data against unauthorized disclosures or access, accidental or unlawful destruction, or accidental loss or alteration must be put in place. Sponsor personnel whose responsibilities require access to personal data agree to keep the identity of subjects confidential. The informed consent obtained from the subject (or his or her legally acceptable representative) includes explicit consent for the processing of personal data and for the investigator/institution to allow direct access to his or her original medical records (source data/documents) for study-related monitoring, audit, IEC/IRB review, and regulatory inspection. This consent also addresses the transfer of the data to other entities and to other countries.

The subject has the right to request through the investigator access to his or her personal data and the right to request rectification of any data that are not correct or complete. Reasonable steps will be taken to respond to such a request, taking into consideration the nature of the request, the conditions of the study, and the applicable laws and regulations.

Exploratory immunogenicity research is not conducted under standards appropriate for the return of data to subjects. In addition, the sponsor cannot make decisions as to the significance of any findings resulting from exploratory research. Therefore, exploratory research data will not be returned to subjects or investigators, unless required by law or local regulations. Privacy and confidentiality of data generated in the future on stored samples will be protected by the same standards applicable to all other clinical data.

16.2.6. Long-term Retention of Samples for Additional Future Research

Samples collected in this study, for which consent has been obtained and for which additional material is available after study-specific testing is complete, may be stored for up to 15 years (or

according to local regulations) for potential future research. Samples will only be used to understand Ebola vaccine- and disease-related questions and to develop tests/assays related to the characterization of EBOV-directed immune responses or diagnostic tests. The research may begin at any time during the study or the post-study storage period.

Stored samples will be coded throughout the sample storage and analysis process and will not be labeled with personal identifiers. Subjects may withdraw consent for their samples to be stored for potential future research at any time (see Section 10.3, Withdrawal From the Use of Research Samples). In such case, their blood samples will be destroyed after all the tests specified for this study have been concluded.

The sponsor will be responsible for the overall management of the sample inventory, shipping plan, allocation and storage of samples. The sponsor will establish a Material Transfer Agreement between all relevant parties (site, sponsor and the central laboratory charged with shipment and distribution of the samples). These Material Transfer Agreements will take into account national regulations and will be submitted for ethics approval and be in place before a site will be activated. Samples will be stored securely at facilities as directed by the sponsor.

16.2.7. Country Selection

This study will only be conducted in those countries where the intent is to launch or otherwise help ensure access to the developed product if the need for the product persists, unless explicitly addressed as a specific ethical consideration in Section 16.1, Study-Specific Design Considerations.

17. ADMINISTRATIVE REQUIREMENTS

17.1. Protocol Amendments

Neither the investigator nor the sponsor will modify this protocol without a formal amendment by the sponsor. All protocol amendments must be issued by the sponsor, and signed and dated by the investigator. Protocol amendments must not be implemented without prior IEC/IRB approval, or when the relevant competent authority has raised any grounds for non-acceptance, except when necessary to eliminate immediate hazards to the subjects, in which case the amendment must be promptly submitted to the IEC/IRB and relevant competent authority. Documentation of amendment approval by the investigator and IEC/IRB must be provided to the sponsor. When the change(s) involve(s) only logistic or administrative aspects of the study, the IEC/IRB (where required) only needs to be notified.

During the course of the study, in situations where a departure from the protocol is unavoidable, the investigator or other physician in attendance will contact the appropriate sponsor representative listed in the Contact Information page(s), which will be provided as a separate document). Except in emergency situations, this contact should be made before implementing any departure from the protocol. In all cases, contact with the sponsor must be made as soon as possible to discuss the situation and agree on an appropriate course of action. The data recorded in the CRF and source documents will reflect any departure from the protocol, and the source documents will describe this departure and the circumstances requiring it.

17.2. Regulatory Documentation

17.2.1. Regulatory Approval/Notification

This protocol and any amendment(s) must be submitted to the appropriate regulatory authorities in each respective country, if applicable. A study may not be initiated until all local regulatory requirements are met.

17.2.2. Required Prestudy Documentation

The following documents must be provided to the sponsor before shipment of study vaccine to the study site:

- Protocol and amendment(s), if any, signed and dated by the PI
- A copy of the dated and signed (or sealed, where appropriate per local regulations), written IEC/IRB approval of the protocol, amendments, ICF, any recruiting materials, and if applicable, subject compensation programs. This approval must clearly identify the specific protocol by title and number and must be signed (or sealed, where appropriate per local regulations) by the chairman or authorized designee.
- Name and address of the IEC/IRB, including a current list of the IEC/IRB members and their function, with a statement that it is organized and operates according to GCP and the applicable laws and regulations. If accompanied by a letter of explanation, or equivalent, from the IEC/IRB, a general statement may be substituted for this list. If an investigator or a member of the study-site personnel is a member of the IEC/IRB, documentation must be obtained to state that this person did not participate in the deliberations or in the vote/opinion of the study.
- Regulatory authority approval or notification, if applicable.
- Signed and dated statement of investigator (eg, Form FDA 1572), if applicable.
- Documentation of investigator qualifications (eg, curriculum vitae).
- Completed investigator financial disclosure form from the PI, where required.
- Signed and dated clinical trial agreement, which includes the financial agreement.
- Any other documentation required by local regulations.

The following documents must be provided to the sponsor before enrollment of the first subject:

- Completed investigator financial disclosure forms from all subinvestigators.
- Documentation of subinvestigator qualifications (eg, curriculum vitae).
- Name and address of any local laboratory conducting tests for the study, and a dated copy of current laboratory normal ranges for these tests, if applicable.
- Local laboratory documentation demonstrating competence and test reliability (eg, accreditation/license), if applicable.

17.3. Subject Identification, Enrollment, and Screening Logs

The investigator agrees to complete a subject identification and enrollment log to permit easy identification of each subject during and after the study. This document will be reviewed by the sponsor study-site contact for completeness.

The subject identification and enrollment log will be treated as confidential and will be filed by the investigator in the study file. To ensure subject confidentiality, no copy will be made. All reports and communications relating to the study will identify subjects by subject identification and date of birth. In cases where the subject is not randomized into the study, the date seen and date of birth will be used.

The investigator must also complete a subject screening log, which reports on all subjects who were seen to determine eligibility for inclusion in the study.

17.4. Source Documentation

At a minimum, source documents consistent in the type and level of detail with that commonly recorded at the study site as a basis for standard medical care, must be available for the following: subject identification, eligibility, and study identification; study discussion and date of signed informed consent; dates of visits; results of safety and immunogenicity parameters as required by the protocol; record of all adverse events and follow-up of adverse events; concomitant medication; study vaccine receipt/dispensing/return records; study vaccine administration information; and date of study completion and reason for early discontinuation of study vaccine or withdrawal from the study, if applicable.

The author of an entry in the source documents should be identifiable.

Specific details required as source data for the study and source data collection methods will be reviewed with the investigator before the study and will be described in the monitoring guidelines (or other equivalent document).

The information contained in the subject diary is primary data. Either the physical diary or diary information collected in the subject's medical record will be considered a source document. Information from the diary provided to subjects to record solicited local and systemic adverse events until 7 days after each vaccination, will be reviewed by the investigator or designee to transcribe into the relevant parts of the CRF as described in the CRF Completion Guidelines.

17.5. Case Report Form Completion

Case report forms are prepared and provided by the sponsor for each subject in electronic format. All CRF entries, corrections, and alterations must be made by the investigator or authorized study-site personnel. The investigator must verify that all data entries in the CRF are accurate and correct.

The study data will be transcribed by study-site personnel from the source documents onto an electronic CRF, if applicable. Study-specific data will be transmitted in a secure manner to the sponsor.

Data must be entered into CRF in English. The CRF must be completed as soon as possible after a subject visit and the forms should be available for review at the next scheduled monitoring visit.

If necessary, queries will be generated in the eDC tool. If corrections to a CRF are needed after the initial entry into the CRF, this can be done in either of the following ways:

- Investigator and study-site personnel can make corrections in the eDC tool at their own initiative or as a response to an auto query (generated by the eDC tool).
- Sponsor or sponsor delegate can generate a query for resolution by the investigator and study-site personnel.

17.6. Data Quality Assurance/Quality Control

Steps to be taken to ensure the accuracy and reliability of data include the selection of qualified investigators and appropriate study sites, review of protocol procedures with the investigator and study-site personnel before the study, and periodic monitoring visits by the sponsor. Written instructions will be provided for collection, handling, storage, and shipment of samples.

Guidelines for CRF completion will be provided and reviewed with study-site personnel before the start of the study. The sponsor will review CRF for accuracy and completeness during on-site monitoring visits and after transmission to the sponsor; any discrepancies will be resolved with the investigator or designee, as appropriate. After upload of the data into the study database they will be verified for accuracy and consistency with the data sources.

17.7. Record Retention

In compliance with the ICH/GCP guidelines, the investigator/institution will maintain all CRF and all source documents that support the data collected from each subject, as well as all study documents as specified in ICH/GCP Section 8, Essential Documents for the Conduct of a Clinical Trial, and all study documents as specified by the applicable regulatory requirement(s). The investigator/institution will take measures to prevent accidental or premature destruction of these documents.

Essential documents must be retained until at least 2 years after the last approval of a marketing application in an ICH region and until there are no pending or contemplated marketing applications in an ICH region or until at least 2 years have elapsed since the formal discontinuation of clinical development of the investigational product. These documents will be retained for a longer period if required by the applicable regulatory requirements or by an agreement with the sponsor. It is the responsibility of the sponsor to inform the investigator/institution as to when these documents no longer need to be retained.

If the responsible investigator retires, relocates, or for other reasons withdraws from the responsibility of keeping the study records, custody must be transferred to a person who will accept the responsibility. The sponsor must be notified in writing of the name and address of the new custodian. Under no circumstance shall the investigator relocate or dispose of any study documents before having obtained written approval from the sponsor.

If it becomes necessary for the sponsor, representatives from the WRAIR, United States Army Medical Research and Materiel Command (USAMRMC), the FDA, or the appropriate regulatory authority to review any documentation relating to this study, the investigator/institution must permit access to such reports.

17.8. Monitoring

The sponsor will perform on-site monitoring visits as frequently as necessary. The monitor will record dates of the visits in a study site visit log that will be kept at the study site. The first post-initiation visit will be made as soon as possible after enrollment has begun. At these visits, the monitor will compare the data entered into the CRF with the source documents (eg, hospital/clinic/physician's office medical records). The nature and location of all source documents will be identified to ensure that all sources of original data required to complete the CRF are known to the sponsor and study-site personnel and are accessible for verification by the sponsor study-site contact. If electronic records are maintained at the study site, the method of verification must be discussed with the study-site personnel.

Direct access to source documents (medical records) must be allowed for the purpose of verifying that the recorded data are consistent with the original source data. Findings from this review will be discussed with the study-site personnel. The sponsor expects that, during monitoring visits, the relevant study-site personnel will be available, the source documents will be accessible, and a suitable environment will be provided for review of study-related documents. The monitor will meet with the investigator on a regular basis during the study to provide feedback on the study conduct.

There will be independent monitoring of the pharmacy and preparation of study vaccines by an unblinded monitor (independent study vaccine monitor); regular monitors will be blinded.

In addition to on-site monitoring visits, remote contacts can occur. It is expected that during these remote contacts, study-site personnel will be available to provide an update on the progress of the study at the site.

17.9. Regulatory Audits

The knowledge of any pending compliance inspection/visit by the US FDA, Office for Human Research Protections (OHRP), or other government agency concerning clinical investigation or research, the issuance of Inspection Reports, FDA Form 483, warning letters or actions taken by any regulatory agencies including legal or medical actions and any instances of serious or continuing noncompliance with the regulations or requirements will be reported immediately to the sponsor and WRAIR Human Subjects Protection Branch (HSPB). The WRAIR HSPB will report knowledge of any pending inspections/audits by regulatory agencies to the USAMRMC Office of Research Protections (ORP) Human Research Protection Office (HRPO).

17.10. Study Completion/Termination

17.10.1. Study Completion/End of Study

The study is considered completed with the last visit for the last subject participating in the study. The final data from the study site will be sent to the sponsor (or designee) after completion of the final subject visit at that study site, in the time frame specified in the Clinical Trial Agreement.

17.10.2. Study Termination

The sponsor reserves the right to close the study site or terminate the study at any time for any reason at the sole discretion of the sponsor. Study sites will be closed upon study completion. A study site is considered closed when all required documents and study supplies have been collected and a study-site closure visit has been performed.

The investigator may initiate study-site closure at any time, provided there is reasonable cause and sufficient notice is given in advance of the intended termination.

Reasons for the early closure of a study site by the sponsor or investigator may include but are not limited to:

- Failure of the investigator to comply with the protocol, the requirements of the IEC/IRB or local health authorities, the sponsor's procedures, or GCP guidelines
- Inadequate recruitment of subjects by the investigator
- Discontinuation of further study vaccine development

17.11. On-site Audits

Representatives of the sponsor's clinical quality assurance department may visit the study site at any time during or after completion of the study to conduct an audit of the study in compliance with regulatory guidelines and company policy. These audits will require access to all study records, including source documents, for inspection. Subject privacy must, however, be respected. The investigator and study-site personnel are responsible for being present and available for consultation during routinely scheduled study-site audit visits conducted by the sponsor or its designees.

Similar auditing procedures may also be conducted by agents of any regulatory body, either as part of a national GCP compliance program or to review the results of this study in support of a regulatory submission. The investigator should immediately notify the sponsor if he or she has been contacted by a regulatory agency concerning an upcoming inspection.

17.12. Site-specific addendum

A site-specific addendum will be employed. The core protocol will be followed by all sites except for the differences noted in the site-specific addendum, which must be approved by the sponsor prior to submission to the IRBs and regulatory authorities.

17.13. Use of Information and Publication

All information, including but not limited to information regarding Ad26.ZEBOV and MVA-BN-Filo or the sponsor's operations (eg, patent application, formulas, manufacturing processes, basic scientific data, prior clinical data, formulation information) supplied by the sponsor to the investigator and not previously published, and any data, including exploratory research data, generated as a result of this study, are considered confidential and remain the sole property of the sponsor. The investigator agrees to maintain this information in confidence and use this information only to accomplish this study, and will not use it for other purposes without the sponsor's prior written consent.

The investigator understands that the information developed in the study will be used by the sponsor in connection with the continued development of Ad26.ZEBOV and MVA-BN-Filo, and thus may be disclosed as required to other clinical investigators or regulatory agencies. To permit the information derived from the clinical studies to be used, the investigator is obligated to provide the sponsor with all data obtained in the study.

The results of the study will be reported in a Clinical Study Report generated by the sponsor and will contain data from all study sites that participated in the study as per protocol. Recruitment performance or specific expertise related to the nature and the key assessment parameters of the study will be used to determine a coordinating investigator. Results of exploratory analyses performed after the Clinical Study Report has been issued will be reported in a separate report and will not require a revision of the Clinical Study Report. Study subject identifiers will not be used in publication of results. Any work created in connection with performance of the study and contained in the data that can benefit from copyright protection (except any publication by the investigator as provided for below) shall be the property of the sponsor as author and owner of copyright in such work.

Consistent with Good Publication Practices and International Committee of Medical Journal Editors guidelines, the sponsor and consortium members shall have the right to publish such primary (multicenter) data and information without approval from the individual investigators. The individual investigators have the right to publish study site-specific data after the primary data are published. If an investigator wishes to publish information from the study, a copy of the manuscript must be provided to the sponsor for review at least 60 days before submission for publication or presentation. Expedited reviews will be arranged for abstracts, poster presentations, or other materials. If requested by the sponsor in writing, the investigator will withhold such publication for up to an additional 60 days to allow for filing of a patent application. In the event that issues arise regarding scientific integrity or regulatory compliance, the sponsor will review these issues with the investigator. The sponsor will not mandate modifications to scientific content and does not have the right to suppress information. For multicenter study designs and substudy approaches, secondary results generally should not be published before the primary endpoints of a study have been published. Similarly, investigators will recognize the integrity of a multicenter study by not submitting for publication data derived from the individual study site until the combined results from the completed study have been submitted for publication, within 12 months of the availability of the final data (tables, listings,

graphs), or the sponsor confirms there will be no multicenter study publication. WRAIR recognizes the importance of communicating medical study data and therefore encourages their publication in reputable scientific journals and at seminars or conferences. Any results of medical investigations and or publication/lecture/manuscripts based thereon, shall be exchanged and discussed by the investigator and the USAMRMC prior to submission for publication or presentation.

Results from investigations shall not be made available to any third party by the investigating team outside the publication procedure as outlined previously. WRAIR will not quote from publications by investigators in its scientific information and/or promotional material without full acknowledgment of the source (i.e., author and reference). All publications written by WRAIR investigators must be reviewed and approved through the WRAIR publications approval process.

Registration of Clinical Studies and Disclosure of Results

The sponsor will register and disclose the existence of and the results of clinical studies as required by law.

REFERENCES

1. Baden LR, Liu J, Li H, et al. Induction of HIV-1-specific mucosal immune responses following intramuscular recombinant adenovirus serotype 26 HIV-1 vaccination of humans. J Infect Dis. 2015;211(4):518-528.
2. Baden LR, Walsh SR, Seaman MS, et al. First-in-human evaluation of the safety and immunogenicity of a recombinant adenovirus serotype 26 HIV-1 Env vaccine (IPCAVD 001). J Infect Dis. 2013;207:240-247.
3. Baize S, Pannetier D, Oestereich L, et al. Emergence of Zaire Ebola virus disease in Guinea. N Engl J Med. 2014;371(15):1418-1425.
4. Barouch DH, Liu J, Peter L, et al. Characterization of humoral and cellular immune responses elicited by a recombinant adenovirus serotype 26 HIV-1 Env vaccine in healthy adults (IPCAVD 001). J Infect Dis. 2013;207:248-256.
5. Clinical Study Report MAL-V-A001. A Phase I/IIa, double-blind, randomized, placebo-controlled, dose-escalation clinical study evaluating safety, tolerability and immunogenicity of two dose levels of recombinant adenoviral serotype Ad35 and serotype Ad26 vectors expressing the malaria *Plasmodium falciparum* circumsporozoite antigen administered as heterologous prime-boost regimen, and assessing protective efficacy of the higher dose in a malaria challenge model in unblinded conditions. Crucell Holland B.V. (Aug 2014).
6. Clinical Study Report POX-MVA-013. A randomized, double-blind, placebo-controlled Phase III trial to evaluate immunogenicity and safety of three consecutive production lots of IMVAMUNE® (MVA-BN®) smallpox vaccine in healthy, Vaccinia-naïve subjects. Bavarian Nordic. (Aug 2015).
7. Elizaga ML, Vasan S, Marovich MA, et al.; MVA Cardiac Safety Working Group. Prospective surveillance for cardiac adverse events in healthy adults receiving modified vaccinia Ankara vaccines: a systematic review. PLoS One. 2013;8(1):e54407.
8. Frey SE, Newman FK, Kennedy JS, et al. Clinical and immunologic responses to multiple doses of IMVAMUNE (Modified Vaccinia Ankara) followed by Dryvax challenge. Vaccine. 2007;25(51):8562-8573.
9. Friedrich B, Trefry J, Biggins J, et al. Potential vaccines and post-exposure treatments for filovirus infections. Viruses. 2012;4(9):1619-1650.
10. IMVANEX suspension for injection. UK Summary of Product Characteristics. Available at: http://www.ema.europa.eu/ema/index.jsp?curl=pages/medicines/human/medicines/002596/human_med_001666.jsp&mid=WC0b01ac058001d124. Accessed 24 March 2015.
11. Investigator's Brochure: JNJ-61210474 (Ad26.ZEBOV), Edition 2.1. Crucell Holland B.V. (June 2015). For updated information, refer to Edition 4 (Janssen Vaccines & Prevention, 23 June 2016) and Addendum 1 for this edition (30 August 2016).
12. Investigator's Brochure: MVA-BN-Filo (MVA-mBN226B), Edition 5. Bavarian Nordic A/S (Mar 2015). For updated information, refer to Edition 10 (11 July 2016).
13. Investigator's Brochure: Ad26.ENVA.01 HIV-1 Vaccine, Edition 3. Crucell Holland B.V. (June 2013).
14. Investigator's Brochure: JNJ-55471468-AAA, JNJ-55471494-AAA, JNJ-55471520-AAA (Ad26.Mos.HIV) HIV 1 vaccine, Edition 2. Crucell Holland B.V. (Nov 2014).
15. Investigator's Brochure: JNJ-55471468-AAA, JNJ-55471494-AAA, JNJ-55471520-AAA (Ad26.Mos.HIV) HIV 1 vaccine, Addendum to Edition 2. Crucell Holland B.V. (Dec 2014).
16. Karita E, Mutua G, Bekker LG, et al. Safety in a Phase 1 randomized, double-blind, placebo-controlled trial evaluating two adenovirus hiv vaccines in three different geographic regions (IAVI-B003/IPCAVD-004/HVTN091 trial). Poster presented at AIDS Vaccine 2013.
17. MVA-BN® (Modified Vaccinia Ankara - Bavarian Nordic). Available at: http://id.bavarian-nordic.com/pipeline/technology-platform/mva-bn.aspx. Accessed 24 March 2015.

18. Neff J, Modlin J, Birkhead GS, et al. Monitoring the safety of a smallpox vaccination program in the United States: report of the joint Smallpox Vaccine Safety Working Group of the advisory committee on immunization practices and the Armed Forces Epidemiological Board. Clin Infect Dis. 2008;46 Suppl.3:S258-S270.

19. Stittelaar KJ, Kuiken T, de Swart RL, et al. Safety of Modified Vaccinia Virus Ankara (MVA) in immune-suppressed macaques. Vaccine. 2001;19(27):3700-3709.

20. Verheust C, Goossens M, Pauwels K, Breyer D. Biosafety aspects of Modified Vaccinia Virus Ankara (MVA)-based vectors used for gene therapy or vaccination. Vaccine. 2012;30(16):2623-2632.

21. Vollmar J, Arndtz N, Eckl KM, et al. Safety and immunogenicity of IMVAMUNE, a promising candidate as a third generation smallpox vaccine. Vaccine. 2006;24(12):2065-2070.

22. WHO Child Growth Standards (birth to 24 months). Available at: http://www.who.int/childgrowth/en. Accessed 10 March 2015.

ATTACHMENTS

Attachment 1: Test of Understanding (TOU)

Note: A culturally appropriate translation will be made available to participating subjects.

Name participating subject:
Date:
Reviewer of the test:
Score:

Please read each question and answer whether the statement is **True** or **False**.

True	False	
True ☐	False ☐	1. The vaccines you will receive in this study will definitely protect against Ebola.
True ☐	False ☐	2. In this study you will receive 2 injections of either vaccine or salt water.
True ☐	False ☐	3. The vaccines in this study can give you Ebola.
True ☐	False ☐	4. One purpose of this study is to determine if these vaccines are safe to administer to humans.
True ☐	False ☐	5. Participants in this study will need to avoid engaging in activities that may expose them to Ebola virus.
True ☐	False ☐	6. You may take other experimental (test) products while you are taking part in this study.
True ☐	False ☐	7. You may withdraw from the study at any time if you choose.
True ☐	False ☐	8. Women participating in this study are permitted to become pregnant during the study.
True ☐	False ☐	9. A participant in this study may experience side effects after vaccination.
True ☐	False ☐	10. Some participants in this study may develop a positive Ebola test result, despite the fact that they do not have Ebola disease.

Attachment 2: Toxicity Tables for Use in Trials Enrolling Healthy Adults

The abbreviations used in the following tables are:

ALT: alanine aminotransferase; aPTT: activated partial thromboplastin time; AST: aspartate aminotransferase; AV block: atrioventricular block; bpm: beats per minute; CK: creatine kinase; FEV_1: forced expiratory volume in 1 second; g: gram; HI: high; HPF: high power field; INR: international normalized ratio; IV: intravenous; LO: low; mEq: milliequivalent; mm Hg: millimeter of mercury; N: not graded; PT: prothrombin time; PTT: partial thromboplastin time; QTc: QT-interval corrected for heart rate; QTcB: Bazett's corrected QT interval; QTcF: Fridericia's corrected QT interval; RBC: red blood cell; Rx: therapy; ULN: upper limit of normal

CLINICAL ADVERSE EVENTS

Grading scale used for clinical adverse events is adapted from the Division of Microbiology and Infectious Diseases (DMID) Toxicity Tables (2014). For adverse events not included in the tables below, refer to the severity criteria guidelines in Section 12.1.3.

**Cardiovascular**	**Grade 1**	**Grade 2**	**Grade 3**
Arrhythmia		Asymptomatic, transient signs, no Rx required	Recurrent/persistent; symptomatic Rx required
Hemorrhage, blood loss	Estimated blood loss ≤100 mL	Estimated blood loss >100 mL, no transfusion required	Transfusion required
QTcF (Fridericia's correction) [1] or QTcB (Bazett's correction)	Asymptomatic, QTc interval 450-479 ms, *OR* Increase in interval <30 ms above baseline	Asymptomatic, QTc interval 480-499 ms, *OR* Increase in interval 30-60 ms above baseline[2]	Asymptomatic, QTc interval ≥500 ms, *OR* Increase in interval ≥60 ms above baseline
PR interval (prolonged)	PR interval 0.21-0.25 s	PR interval >0.25 s	Type II 2nd degree AV block *OR* Ventricular pause >3.0 s
**Respiratory**	**Grade 1**	**Grade 2**	**Grade 3**
Cough	Transient; no treatment	Persistent cough	Interferes with daily activities
Bronchospasm, acute	Transient; no treatment; FEV_1 71%-80% of peak flow	Requires treatment; normalizes with bronchodilator; FEV_1 60%-70% (of peak flow)	No normalization with bronchodilator; FEV_1 <60% of peak flow
Dyspnea	Does not interfere with usual and social activities	Interferes with usual and social activities, no treatment	Prevents daily and usual social activity or requires treatment
**Gastrointestinal**	**Grade 1**	**Grade 2**	**Grade 3**
Nausea/vomiting	Minimal symptoms; caused minimal or no interference with work, school or self-care activities	Notable symptoms; required modification in activity or use of medications; did not result in loss of work or cancellation of social activities	Incapacitating symptoms; required bed rest and/or resulted in loss of work or cancellation of social activities

[1] Inclusion dependent upon protocol requirements.

[2] The Grade 2 increase in interval is changed from 30-50 ms to 30-60 ms since the original DMID Toxicity Tables (2014) did not cover the increase in interval between 50 and 60 ms.

Diarrhea	2-3 loose or watery stools or <400 g/24 hours	4-5 loose or watery stools or 400-800 g/24 hours	6 or more loose or watery stools or >800 g/24 hours or requires IV hydration
**Reactogenicity**	**Grade 1**	**Grade 2**	**Grade 3**
Local reactions			
Pain/tenderness at injection site	Aware of symptoms but easily tolerated; does not interfere with activity; discomfort only to touch	Notable symptoms; required modification in activity or use of medications; discomfort with movement	Incapacitating symptoms; inability to do work or usual activities; significant discomfort at rest
Erythema/redness[3]	2.5-5 cm	5.1-10 cm	>10 cm
Induration/swelling[4]	2.5-5 cm and does not interfere with activity	5.1-10 cm or interferes with activity	>10 cm or prevents daily activity
Itching at the injection site	Minimal symptoms; caused minimal or no interference with work, school or self-care activities	Notable symptoms; required modification in activity or use of medications; did not result in loss of work or cancellation of social activities	Incapacitating symptoms; required bed rest and/or resulted in loss of work or cancellation of social activities
Systemic reactions			
Allergic reaction	Pruritus without rash	Localized urticaria	Generalized urticaria; angioedema or anaphylaxis
Headache	Minimal symptoms; caused minimal or no interference with work, school or self-care activities	Notable symptoms; required modification in activity or use of medications; did not result in loss of work or cancellation of social activities	Incapacitating symptoms; required bed rest and/or resulted in loss of work or cancellation of social activities
Fatigue/malaise	Minimal symptoms; caused minimal or no interference with work, school or self-care activities	Notable symptoms; required modification in activity or use of medications; did not result in loss of work or cancellation of social activities	Incapacitating symptoms; required bed rest and/or resulted in loss of work or cancellation of social activities
Myalgia	Minimal symptoms; caused minimal or no interference with work, school or self-care activities	Notable symptoms; required modification in activity or use of medications; did not result in loss of work or cancellation of social activities	Incapacitating symptoms; required bed rest and/or resulted in loss of work or cancellation of social activities

[3] In addition to grading the measured local reaction at the greatest single diameter, the measurement should be recorded as a continuous variable.

[4] Induration/swelling should be evaluated and graded using the functional scale as well as the actual measurement.

Arthralgia	Minimal symptoms; caused minimal or no interference with work, school or self-care activities	Notable symptoms; required modification in activity or use of medications; did not result in loss of work or cancellation of social activities	Incapacitating symptoms; required bed rest and/or resulted in loss of work or cancellation of social activities
Chills	Minimal symptoms; caused minimal or no interference with work, school or self-care activities	Notable symptoms; required modification in activity or use of medications; did not result in loss of work or cancellation of social activities	Incapacitating symptoms; required bed rest and/or resulted in loss of work or cancellation of social activities

LABORATORY TOXICITY GRADING

Grading scale used for lab assessments is based on 'FDA's Toxicity Grading Scale for Healthy Adult and Adolescent Volunteers Enrolled in Preventive Vaccine Clinical Trials', but grade 3 and 4 are pooled below, consistent with the 3 scale toxicity grading used throughout the protocol. If a laboratory value falls within the grading as specified below but also within the local laboratory normal limits, the value is considered as normal. For hemoglobin only the change from reference is used for the grading. The FDA table does not include toxicity grading for hematocrit, RBC counts or INR.

Blood, Serum, or Plasma Chemistries[5]	LO/HI/N [6]	Mild (Grade 1)	Moderate (Grade 2)	Severe (Grade 3)
Sodium (mEq/L or mmol/L)	LO	132-134	130-131	≤129
	HI	144-145	146-147	≥148
Potassium (mEq/L or mmol/L)	LO	3.5-3.6	3.3-3.4	≤3.2
	HI	5.1-5.2	5.3-5.4	≥5.5
Glucose (mg/dL)	LO	65-69	55-64	≤54
	HI [7]	100-110	111-125	>125
	HI [8]	110-125	126-200	>200
Blood urea nitrogen	HI	23-26 (mg/dL) or 8.3-9.4 (mmol/L)	27-31 (mg/dL) or 9.5- 11.2 (mmol/L)	>31 (mg/dL) or >11.2 (mmol/L)
Creatinine	N	1.5-1.7 (mg/dL) or 133-151 (µmol/L)	1.8-2.0 (mg/dL) or 152-177 (µmol/L)	>2.0 (mg/dL) or > 177 (µmol/L)
Calcium (mg/dL)	LO	8.0-8.4	7.5-7.9	<7.5
	HI	10.5-11.0	11.1-11.5	>11.5
Magnesium (mg/dL)	LO	1.3-1.5	1.1-1.2	<1.1
Phosphorus (mg/dL)	LO	2.3-2.5	2.0-2.2	<2.0
CK (mg/dL)	N	1.25-1.5 x ULN	1.6-3.0 x ULN	≥3.1 x ULN
Albumin (g/dL)	LO	2.8-3.1	2.5-2.7	<2.5

[5] Depending upon the laboratory used, reference ranges, eligibility ranges and grading may be split out by sex and/or age.
[6] Low, High, Not Graded.
[7] Fasting.
[8] Non-fasting.

Total protein (g/dL)	LO	5.5-6.0	5.0-5.4	<5.0
Alkaline phosphatase (U/L)	N	1.1-2 x ULN	2.1-3 x ULN	>3 x ULN
AST (U/L)	HI	1.1-2.5 x ULN	2.6-5 x ULN	>5 x ULN
ALT (U/L)	HI	1.1-2.5 x ULN	2.6-5 x ULN	>5 x ULN
Bilirubin, serum total (mg/dL) – when accompanied by any increase in Liver Function Test		1.1–1.25 x ULN	1.2 –1.5 x ULN	>1.5 x ULN
Bilirubin, serum total (mg/dL) – when Liver Function Test is normal		1.1–1.5 x ULN	1.6–2.0 x ULN	>2.0 x ULN
Amylase (U/L)	N	1.1-1.5 x ULN	1.6-2.0 x ULN	>2.0 x ULN
Lipase (U/L)	N	1.1-1.5 x ULN	1.6-2.0 x ULN	>2.0 x ULN

**Hematology**	**LO/HI/N** [9]	**Mild (Grade 1)**	**Moderate (Grade 2)**	**Severe (Grade 3)**
Hemoglobin (women) change from baseline (g/dL)	LO	Any decrease-1.5	1.6-2.0	>2.0
Hemoglobin (men) change from baseline (g/dL)	LO	Any decrease-1.5	1.6-2.0	>2.0
White blood cell count (cell/mm^3)	HI	10,800-15,000	15,001-20,000	>20,000
	LO	2,500-3,500	1,500-2,499	<1,500
Lymphocytes (cell/mm^3)	LO	750-1,000	500-749	< 500
Neutrophils (cell/mm^3)*	LO	1,500-2,000	1,000-1,499	< 1000
Eosinophils (cell/mm^3)	HI	650-1500	1501-5000	> 5000
Platelets (cell/mm^3)	LO	125,000-140,000	100,000-124,000	<100,000
**Coagulation**				
PT (seconds)	HI	1.0-1.10 x ULN	1.11-1.20 x ULN	>1.20 x ULN
International Normalized Ratio (INR)[10]	HI	1.1-1.5 x ULN	1.6-2.0 x ULN	>2.0 x ULN
PTT or aPTT (seconds)	HI	1.0-1.2 x ULN	1.21-1.4 x ULN	>1.4 x ULN
Fibrinogen (mg/dL)	HI	400-500	501-600	>600
	LO	150-200	125-149	<125
**Urine**				
Protein (dipstick)	HI	Trace	1+	2+
Glucose (dipstick)	HI	Trace	1+	2+
Blood (microscopic) - red blood cells per high power field (RBC/HPF)	HI	1-10	11-50	>50 and/or gross blood

* For African subjects, the following absolute neutrophil counts should be used to determine the severity level: grade 1 = 750 – 999 cell/uL; grade 2 = 500-749 cells/uL; grade 3 <500 cells/uL

[9] Low, High, Not Graded.
[10] For INR, the values in the table are based on the Division of AIDS Table for Grading the Severity of Adult and Pediatric Adverse Events, 2009.

VITAL SIGNS TOXICITY GRADING

Grading scale used for vital signs is according to DMID Toxicity Tables (2014)

**Vital Signs**	**LO/HI/N** [11]	**Mild (Grade 1)** [12]	**Moderate (Grade 2)**	**Severe (Grade 3)**
Fever (°C)[13]	HI	38.0-38.4	38.5-38.9	>38.9
Fever (°F)	HI	100.4-101.1	101.2-102.0	>102.0
Tachycardia	HI	101-115 bpm	116-130 bpm	>130 bpm or ventricular dysrhythmias
Bradycardia	LO	50-54 or 45-50 bpm if baseline <60 bpm	45-49 or 40-44 bpm if baseline <60 bpm	<45 or <40 bpm if baseline <60 bpm
Hypertension (systolic) - mm Hg[14]	HI	141-150	151-160	>160
Hypertension (diastolic) - mm Hg	HI	91-95	96-100	>100
Hypotension (systolic) - mm Hg	LO	85-89	80-84	<80
Tachypnea - breaths per minute	HI	23-25	26-30	>30

[11] Low, High, Not Graded.

[12] If initial bound of grade 1 has gap from reference range or eligibility range, calculations based on the New England Journal of Medicine (NEJM) reference ranges.

[13] Axillary temperature. A protocol should select either °C or °F for inclusion.

[14] Assuming subject is awake, resting, and supine; for adverse events, 3 measurements on the same arm with concordant results.

Attachment 3:Hemoglobin Cut-off Values

Where no institutional normal reference ranges is available for hemoglobin, the following cut-off values are proposed. It is imperative to note that there are no standard accepted normative values for hemoglobin in most African countries and therefore, the following recommendations are based on the review of several published sources and in consultation with the sites involved with the study. In the table below, 'simplified' means that the number of cut-off categories has been reduced to decrease complexity and facilitate understanding about eligibility. Similarly, 'adjusted for safety' means that the references may quote lower average or -2 SD values for hemoglobin but these values are considered to be too low for these subjects.

Group	Value (g/dL)		Reference	Outcome
Adult and HIV+	Male 12.1	Female 9.5	LLN value for local sites in Kenya and Uganda, current values being used in Phase 1	Values kept for consistency
Adolescent 16-18 yrs	Male 12.1	Female 9.5	Robins reference 10.4 g/dL for girls and 12.4 g/dL for boys	Values simplified to correspond to adult cut-offs
Adolescent 11-15 yrs	Male 11.0	Female 9.5	Robins reference 11.0 g/dL for girls and boys	Value adjusted down for boys, no change for girls
Children 6-10 yrs	11.0		Robins reference 10.7 g/dL for girls and boys	Value simplified
Children 2-5 yrs	11.0		Robins reference 10.4 g/dL for girls and boys, LLN value for local lab in Kenya 11.5 g/dL for girls and 14.5 g/dL for boys, Schellenberg reference 8.2-9.3 g/dL average anemic defined as <11.0 g/dL	Value simplified and adjusted for safety to 11.0 g/dL
Children 1-2 yrs	11.0		Schellenberg reference 8.0 g/dL average anemic defined as <11.0 g/dL, DMID toxicity table 11.0 g/dL	Value simplified and adjusted for safety to 11.0 g/dL

Robins E and Blum S. Hematologic Reference Values for African American Children and Adolescents. American J Hematology. 2007:82, 611-614.

Schellenberg D, et al. The silent burden of anaemia in Tanzanian children: a community-based study. Bulletin of the World Health Organization 2003;81:581-590.

DMID US FDA Guidance document DIVISION OF MICROBIOLOGY AND INFECTIOUS DISEASES (DMID) PEDIATRIC TOXICITY TABLES NOVEMBER 2007

INVESTIGATOR AGREEMENT

I have read this document and agree that it contains all necessary details for carrying out this study. I will conduct the study as outlined herein and will complete the study within the time designated.

I will provide copies of the document and all pertinent information to all individuals responsible to me who assist in the conduct of this study. I will discuss this material with them to ensure that they are fully informed regarding the study vaccine, the conduct of the study, and the obligations of confidentiality.

Coordinating Investigator (where required):

Name (typed or printed): ____________________

Institution and Address: ____________________

____

____

____

Signature: ____________________ Date: ____________________

(Day Month Year)

Principal (Site) Investigator:

Name (typed or printed): ____________________

Institution and Address: ____________________

____

____

____

Telephone Number: ____________________

Signature: ____________________ Date: ____________________

(Day Month Year)

Sponsor's Responsible Medical Officer:

Name (typed or printed): [redacted]

Institution: [redacted]

Signature: [electronic signature appended at the end of the protocol] Date: ____________________

(Day Month Year)

Note: If the address or telephone number of the investigator changes during the course of the study, written notification will be provided by the investigator to the sponsor, and a protocol amendment will not be required.

LAST PAGE

SIGNATURES

Signed by	Date	Justification
[redacted]	[redacted]	Document Approval
