# Supplementary material for: Safety and Immunogenicity of an Accelerated Ebola Vaccination Schedule in People with and without Human Immunodeficiency Virus: A Randomized Clinical Trial
Source: Vaccines (Basel). 2024 May 4;12(5):497. doi: 10.3390/vaccines12050497 (PMC11125575; doi:10.3390/vaccines12050497)
Supplement: Supplementary file 1 [file vaccines-12-00497-s001.zip › Supplement/Supplement S2. Statistical analysis plan.pdf]

Janssen Vaccines & Prevention B.V.*

Statistical Analysis Plan
Amendment 1

A Randomized, Observer-blind, Placebo-controlled, Two-part, Phase 2 Study to Evaluate the Safety, Tolerability and Immunogenicity of Two Prime-boost Regimens of the Candidate Prophylactic Vaccines for Ebola Ad26.ZEBOV and MVA-BN-Filo

Protocol VAC52150EBL2003; Phase 2

Walter Reed Army Institute of Research and Janssen Vaccines & Prevention B.V.

IND Number: 16280

VAC52150 (Ad26.ZEBOV,MVA-BN-Filo [MVA-mBN226B])

* Janssen Vaccines & Prevention B.V. (formerly known as Crucell Holland B.V.) is a Janssen pharmaceutical company of Johnson & Johnson and is hereafter referred to as the sponsor of the study.

Status: Approved
Date: 16 April 2019
Prepared by: Walter Reed Army Institute of Research and Janssen Vaccines & Prevention B.V.
Document No.: EDMS-ERI-120337724_v2.0

Compliance: The study described in this report was performed according to the principles of Good Clinical Practice (GCP).

Confidentiality Statement
The information in this document contains trade secrets and commercial information that are privileged or confidential and may not be disclosed unless such disclosure is required by applicable law or regulations. In any event, persons to whom the information is disclosed must be informed that the information is privileged or confidential and may not be further disclosed by them. These restrictions on disclosure will apply equally to all future information supplied to you that is indicated as privileged or confidential.

1. TABLE OF CONTENTS

1. TABLE OF CONTENTS 2
2. AMENDMENT HISTORY 4
3. ABBREVIATIONS 5
1. INTRODUCTION 7
1.1. Trial Objectives 7
1.2. Statistical Hypotheses for Trial Objectives 7
1.3. Trial Design 7
1.4. Sample Size Justification 8
1.5. Randomization and Blinding 8
2. GENERAL ANALYSIS DEFINITIONS 9
2.1. Analysis Visit Windows and Periods 10
2.2. Pooling Algorithm for Analysis Centers 12
2.3. Analysis Sets 13
2.4. Definition of Subgroups 13
3. CHANGES TO THE PLANNED ANALYSIS 15
4. INTERIM ANALYSIS AND DATA MONITORING COMMITTEE REVIEW 15
5. SUBJECT INFORMATION 15
5.1. Disposition Information 16
5.2. Protocol Deviations 16
5.3. Demographics and Baseline Characteristics 16
5.4. Prior and Concomitant Medications 16
6. IMMUNOGENICITY 17
6.1. Endpoints 17
6.2. Immunogenicity Against the Insert 18
6.2.1. Humoral Immune Responses 18
6.2.1.1. Parameters 18
6.2.1.2. Data Handling Rules 19
6.2.1.3. Analysis Methods 19
6.2.2. Cellular Immune Responses 20
6.2.2.1. Parameters 20
6.2.2.2. Data Handling Rules 21
6.2.2.3. Analysis Methods 21
6.3. Immunogenicity Against the Vector 22
6.3.1. Humoral Immune Responses 22
6.3.1.1. Parameter 22
6.3.1.2. Data Handling Rules 22
6.3.1.3. Analysis Methods 22
7. SAFETY 22
7.1. Adverse Events 23
7.1.1. Definitions 23
7.1.2. Data Handling Rules 25
7.1.3. Analysis Methods 25
7.2. HIV Viral Load Assessment and CD4+ Cell Counts 26
7.3. Clinical Laboratory Tests 27
7.3.1. Definitions 27
7.3.2. Data Handling Rules 27
7.3.3. Analysis Methods 28
7.4. Vital Signs and Physical Examination Findings 28

7.5. Electrocardiogram 28

8. REFERENCES 29

9. ATTACHMENTS 30

1. PERIOD ALLOCATION OF ADVERSE EVENTS 30

2. TRANSFORMING ON-SITE ASSESSMENTS AND DIARIES OF SOLICITED ADVERSE EVENTS INTO AN ANALYSIS FORMAT 32

3. TOXICITY TABLES FOR USE IN TRIALS ENROLLING HEALTHY ADULTS 34

4. HEMOGLOBIN CUT-OFF VALUES 40

5. ADDITIONAL RULES FOR ICS ANALYSIS 40

2. AMENDMENT HISTORY

Version	Date	Description
1.0	19 March 2018	Initial version
2.0	14 February 2019	Amendment 1 (this document)

The overall rationale for Amendment 1: The purpose for this amendment is to align all the statistical analyses of Phase 2 and 3 Ebola studies and to address the remarks from the Food and Drug Administration (FDA). The changes made together with the rationale for each change are as follows:

Rationale: The analysis on the immunogenicity analysis set will only be included as a sensitivity analysis if more than 10% of the subjects are excluded from the per protocol analysis set.

2.3 Analysis Sets

Rationale: Use of antiretroviral medications may influence the VNA readout (ie, high background) and the observed titers may not represent the actual VNA titers in HIV-infected subjects. One possible explanation for these observations is that antiretroviral medications may interfere with pseudovirion expressions. This assay is therefore under investigation to determine which will be best for the HIV+ subjects. Only when such an assay is found, will the analysis of the VNA titers for the HIV-infected subjects be performed.

6.2.1.1 Parameters

6.2.1.3 Analysis Methods

Rationale: Dot plots will be generated for both ELISA and VNA. Information was added to clarify.

6.2.1.3 Analysis Methods

Rationale: Definitions for the sample interpretation and responder for cellular assays have been updated to include more details.

6.2.2.1 Parameters

6.2.2.3 Analysis Methods

Rationale: It is clarified that summary tabulations of SAEs, AEs with fatal outcome, AEs leading to permanent stop of study vaccination, Grade 3 AEs and IREs will be presented by System Organ Class (SOC) and Preferred Term (PT).

7.1.3 Analysis Methods

Rationale: It is clarified that imputation of missing end dates of ongoing AEs will only be used to derive the duration of the events; and that all missing AE end dates will be kept as unknown in the analysis dataset and listings.

Attachment 1: PERIOD ALLOCATION OF ADVERSE EVENTS

Rationale: It is clarified that summary tabulations of solicited AEs will count each event once (ie, assigning the highest grade and the relatedness that most implicates the vaccine) within an analysis period and that there will not be multiple listings of the same AE on the same day.

Attachment 2: TRANSFORMING ON-SITE ASSESSMENTS AND DIARIES OF SOLICITED ADVERSE EVENTS INTO AN ANALYSIS FORMAT

Rationale: Minor editorial changes and clarifications have been made throughout the document.

3. ABBREVIATIONS

Ad26	adenovirus serotype 26 (vector)
Ad26.ZEBOV	adenovirus serotype 26 expressing the Ebola virus Mayinga glycoprotein
AE	adverse event
aMLV	amphotropic Murine Leukemia Virus
BMI	body mass index
CI	confidence interval
(e)CRF	(electronic) case report form
CTP	clinical trial protocol
DMID	Division of Microbiology and Infectious Diseases
EBOV	Ebola virus
ELISA	enzyme-linked immunosorbent assay
ELISpot	enzyme-linked immunospot
EU	European Union
FANG	Filovirus Animal Nonclinical Group
FDA	Food and Drug Administration
GP	glycoprotein
HAART	highly active antiretroviral therapy
HIV	human immunodeficiency virus
IC_{50}	50% inhibitory concentration
IC_{90}	90% inhibitory concentration
ICF	informed consent form
ICH	International Council for Harmonisation
ICS	intracellular cytokine staining
IDMC	Independent Data Monitoring Committee
IFN-γ	interferon-γ
IL	interleukin
Inf.U	infectious units
IRE	immediate reportable event
LLOQ	lower limit of quantification
LOD	limit of detection
MedDRA	Medical Dictionary for Regulatory Activities
MVA	Modified Vaccinia Ankara
MVA-BN-Filo	Modified Vaccinia Ankara - Bavarian Nordic vector expressing the glycoproteins of Ebola virus, Sudan virus and Marburg virus and the nucleoprotein of Tai Forest virus (formally known as *Côte d'Ivoire ebolavirus*)
NSAID	non-steroidal anti-inflammatory drug
PBMC	peripheral blood mononuclear cells
SAE	serious adverse event
SAP	statistical analysis plan

SFU	spot-forming units
SSG	statistical support group
TAFV	Tai Forest virus
TNF-α	tumor necrosis factor-α
VNA	virus neutralization assay
vp	viral particles
WHO	World Health Organization
ZEBOV	Zaire ebolavirus

1. INTRODUCTION

There are 2 parts to the VAC52150EBL2003 study. Both parts of the study are being conducted in healthy adults and human immunodeficiency virus (HIV)-infected adults. Enrolment in both Parts 1 and 2 and follow-up are completed. This statistical analysis plan (SAP) describes the analyses that are planned for this study (as detailed in Section 4).

1.1. Trial Objectives

Primary Objectives

- To assess the safety and tolerability of different vaccination schedules of adenovirus serotype 26 expressing the Ebola virus Mayinga glycoprotein (Ad26.ZEBOV) and Modified Vaccinia Ankara Bavarian Nordic vector expressing multiple filovirus proteins (MVA-BN-Filo) administered intramuscularly as heterologous 2-dose regimens in healthy and in HIV-infected adults, with Ad26.ZEBOV (dose 1) and MVA-BN-Filo (dose 2) vaccination on Days 1 and 29, respectively, and MVA-BN-Filo (dose 1) and Ad26.ZEBOV (dose 2) vaccination on Days 1 and 15, respectively.
- To assess the immune responses to the Ebola virus (EBOV) glycoprotein (GP) (as measured by enzyme-linked immunosorbent assay [ELISA] antibody concentration) of different vaccination schedules of Ad26.ZEBOV and MVA-BN-Filo administered intramuscularly as heterologous 2-dose regimens in healthy and in HIV-infected adults, with Ad26.ZEBOV (dose 1) and MVA-BN-Filo (dose 2) vaccination on Days 1 and 29, respectively, and MVA-BN-Filo (dose 1) and Ad26.ZEBOV (dose 2) vaccination on Days 1 and 15, respectively.

Secondary Objective

To compare the safety and tolerability of both Ad26.ZEBOV,MVA-BN-Filo and MVA-BN-Filo,Ad26.ZEBOV regimens between healthy and HIV-infected adults.

Exploratory Objectives

Several exploratory objectives will be investigated, if the outcome measures are available. See the clinical trial protocol (CTP)[1] for the complete list of the exploratory objectives.

1.2. Statistical Hypotheses for Trial Objectives

No formal statistical hypothesis testing is planned for this study. This is because the primary purpose of the study is to provide descriptive information regarding safety and immunogenicity of the 2 vaccination regimens within cohorts of healthy and HIV-infected subjects.

1.3. Trial Design

This is a randomized, observer-blind, placebo-controlled, parallel-group, multicenter, 2-part, Phase 2 study to evaluate the safety, tolerability and immunogenicity of different vaccination regimens using Ad26.ZEBOV at a dose of $5x10^{10}$ viral particles (vp) and MVA-BN-Filo at a dose of $1x10^{8}$ infectious units (Inf.U, nominal titer), administered intramuscularly. The first regimen to be evaluated is a first vaccination with MVA-BN-Filo followed by a second vaccination with Ad26.ZEBOV 14 days later. The second regimen to be evaluated is a first vaccination with Ad26.ZEBOV followed by a second vaccination with MVA-BN-Filo 28 days

later. Both regimens are being evaluated in healthy and HIV-infected adults, aged 18 to 70 years, inclusive at screening. An overview of the study vaccination schedules is provided in Table 1.

A total of 575 subjects were planned to be enrolled, with approximately 300 healthy subjects and 275 HIV-infected subjects. Eligible subjects were those who had never received a candidate Ebola vaccine and had no prior exposure to EBOV (including travel to epidemic Ebola areas less than 1 month prior to screening) or a diagnosis of EBOV disease. See the CTP[1] for further details.

Table 1: Study Vaccination Schedules for Parts 1 and 2

**Part 1: United States of America (USA), N=75**			
**MVA-BN-Filo on Day 1, Ad26.ZEBOV on Day 15**			
Active		Placebo	
Healthy Adult<br>40	HIV+ Adult<br>20	Healthy Adult<br>10	HIV+ Adult<br>5
**Part 2: Africa, N=500**			
**Group 1: Ad26.ZEBOV on Day 1, MVA-BN-Filo on Day 29**			
Active		Placebo	
Healthy Adult<br>160	HIV+ Adult<br>160	Healthy Adult<br>40	HIV+ Adult<br>40
**Group 2: MVA-BN-Filo on Day 1, Ad26.ZEBOV on Day 15**			
Active		Placebo	
Healthy Adult<br>40	HIV+ Adult<br>40	Healthy Adult<br>10	HIV+ Adult<br>10

1.4. Sample Size Justification

An overall sample size of 575 subjects was expected and had to include 460 subjects who had to receive active 2-dose vaccination and 115 subjects who had to receive placebo. The data obtained in these subjects will substantially contribute to the overall safety database of the Ebola program. See Section 11.1 of the CTP[1] for details on the sample size justification.

1.5. Randomization and Blinding

Both Parts 1 and 2 of the study have completed randomization. Central randomization was implemented in this study. The randomization was used to minimize bias in the assignment of subjects to vaccination schedules (groups), to increase the likelihood that known and unknown subject attributes (eg, demographic and baseline characteristics) are evenly balanced across groups, and to enhance the validity of possible comparisons across groups. In addition, randomization was used to minimize bias in the assignment of subjects to study vaccine (active vaccine versus placebo). The randomization within each group was balanced by using randomly permuted blocks. There was stratification by age group (subjects ≥18 to ≤50 years versus subjects >50 years).

For Part 1, subjects were randomized in a 4:1 ratio to either receive MVA-BN-Filo or placebo on Day 1 followed by a second vaccination with Ad26.ZEBOV or placebo 14 days later. Randomization was done separately for healthy subjects (N=50) and HIV-infected subjects (N=25).

For Part 2, subjects were randomly enrolled in 1 of 2 groups at study entry to either receive Ad26.ZEBOV,MVA-BN-Filo at day 1 and day 29 or MVA-BN-Filo, Ad26.ZEBOV at day 1 and day 15 in a 4:1 ratio (ie, Group 1 versus Group 2). Within each group, subjects were randomized in a 4:1 ratio to receive active vaccine versus placebo. Randomization was done separately for healthy subjects (N=250) and HIV-infected subjects (N=250). The start of randomization of HIV-infected subjects in Part 2 was contingent upon acceptable safety data from HIV-infected subjects from Part 1.

For each part, study-site personnel (except for those with primary responsibility for study vaccine preparation and dispensation, and not involved in any other study-related procedure), the sponsor (except for specifically designated sponsor personnel who are independent of the study) and subjects will remain blinded to the study vaccine allocation until the last subject in that part has completed at least the 6-month post second vaccination visit or discontinued earlier and the database has been locked for that part.

Data that may potentially unblind the study vaccine assignment (ie, study vaccine preparation/accountability data, immunogenicity data or other specific laboratory data) will be handled with special care to ensure that the integrity of the data is maintained and the potential for bias is minimized. This can include making special provisions, such as segregating the data in question from view by the investigators, clinical team, or others as appropriate until the time of database lock and unblinding. The pharmacy and preparation of study vaccines was monitored by an independent study vaccine monitor.

2. GENERAL ANALYSIS DEFINITIONS

The type I error rate (α) is set to 0.05 and corresponding 2-sided 95% confidence intervals (CIs) will be calculated wherever applicable. Adjustment of the α (type I error) level due to multiplicity is not applicable for this study as no planned formal statistical hypothesis testing will be performed.

Analysis and evaluation are defined separately for each parameter later in this document together with the description of rules for handling missing or incomplete data. The analyses will include vaccinated subjects with respect to the actual vaccine administered. For instance, if a subject is randomized to the Ad26.ZEBOV followed by MVA-BN-Filo vaccination schedule but is inadvertently vaccinated according to the MVA-BN-Filo followed by Ad26.ZEBOV vaccination schedule, the subject will be included in the latter vaccination schedule. Also, subjects who receive only the first (dose 1) vaccination will be included in the vaccination schedule with the corresponding dose 1 vaccine. On the other hand, subjects who will receive a homologous regimen (ie, Ad26.ZEBOV followed by another Ad26.ZEBOV or MVA-BN-Filo followed by another MVA-BN-Filo) will be excluded from summary analyses (ie, tables and graphs) and listed separately.

In general, the study data will be analyzed as follows:

- Categorical variables will be summarized with a frequency table presenting counts and percentages.

- Continuous variables will be summarized using the following statistics, as appropriate: number of observations, arithmetic mean, geometric mean, corresponding 95% confidence interval (CI), standard deviation, standard error, median, quartiles (Q1 and Q3), minimum and maximum.

Baseline value will be defined as the value of the last available assessment performed prior to dose 1 administration, unless specified otherwise.

For safety assessments, the *baseline value* will be an assessment performed prior to or on the date (only if time of assessment is missing) of the dose 1 administration. The baseline value for immunogenicity assessments will be an assessment performed before or on the date of dose 1 administration. In case of multiple values, the value closest to the vaccination will be used as the baseline.

Reference value (only for immunogenicity) will be defined as the assessment performed on the date of the dose 2 vaccination. In case of multiple values (on the same day of vaccination), the value closest to the vaccination will be used as the reference value.

V*isit day* will be determined relative to the actual day of vaccination (date of dose 1 administration or date of dose 2 administration).

Repeated assessments of immunogenicity and safety (including HIV viral load and CD4+ cell counts in HIV-infected subjects) will be allocated to analysis windows and periods based on Table 2 and Table 3, as described below.

2.1. Analysis Visit Windows and Periods

Because subjects do not always adhere to the protocol visit schedule, the following rules will be applied to assign actual visits (immunogenicity and safety [ie, including only HIV viral load and CD4+ cell counts in HIV-infected subjects]) to analysis visits. The analysis visit windows and target days for each visit are displayed in Table 2. The reference day will be defined as:

– Day of first (dose 1) vaccination if the actual visit occurs prior to the second (dose 2) vaccination.
– Day of second vaccination (dose 2) if the actual visit occurs after the second vaccination.

Only the analysis time points and assays that are in scope for final statistical analysis are to be considered when assigning assessments to analysis visit windows. If a subject has 2 or more assessments within the same interval (analysis visit window), the one closest to the target day will be used for generating tables with descriptive statistics and graphical displays presenting data per time point. If 2 assessments are equidistance from the target day within the same interval, the latest assessment will be used. All assignments will be made in chronological order.

Because the analysis of adverse events (AEs), vital signs and laboratory abnormalities (except HIV viral load and CD4+ cell counts in HIV-infected subjects) will be presented per period (and not per time point), these will be assigned to the analysis period and/or phase based on Table 3.

For HIV viral loads and CD4+ cell counts in HIV-infected subjects, the electronic case report form (eCRF) visit schedules will be used for the post baseline assessments. If only unscheduled visits are present for a time point, then the one closest to the scheduled visit will be used. If distances of multiple assessments to the scheduled visit are equal, the measurement with the latest date will be used.

Table 2: Analysis Visit Windows

Time Interval (Label on Output)	Time Interval PP[a] (Day)	Time Interval IG[b] (Day)	Target Time Point (Day)
Prior to dose 2 vaccination			
Day 1 (Baseline)	≤ 1	≤ 1	1
Day 15 (14 days Post-dose 1)	[12; 18]	[2; 21]	15
Day 29 (28 days Post-dose 1)	[26; 32]	≥ 22	29
After dose 2 vaccination			
Day 36 (21 days Post-dose 2)<br>Day 50 (21 days Post-dose 2)	[19; 25]	[2; 32]	22
Day 57 (42 days Post-dose 2)<br>Day 71 (42 days Post-dose 2)	[40; 46]	[33; 112]	43
Day 195 (180 days Post-dose 2)<br>Day 209 (180 days Post-dose 2)	[166; 196]	[113; 273]	181
Day 380 (365 days Post-dose 2)<br>Day 394 (365 days Post-dose 2)	[336; 396]	≥ 274	366

[a] The analysis based on the per protocol (PP) analysis set will be restricted to data points that fall within this window (ie, protocol-defined window).

[b] The analysis based on the immunogenicity analysis set will be restricted to data points that fall within this window.

Note 1: The analysis windows and target days are based on the relative day (ie, with respect to reference day [day of dose 1 or dose 2 administration]).

Note 2: Day of dose 2 is also considered as Day 1 with respect to the second vaccination (ie, for calculating target days that fall after the second vaccination).

Note 3: Day 36, Day 57, Day 195 and Day 380 apply only to the 14-day interval vaccination schedule. While Day 50, Day 71, Day 209 and Day 394 apply only to the 28-day interval vaccination schedule.

Table 3: Analysis Periods

Phase	Period	Interval	
		From	To
Screening		00:00 on the date of signing the informed consent form	One minute prior to dose 1 administration on Day 1
Regimen*	Post-dose 1	Date and time of dose 1 administration	Minimum of:<br>a) 23:59 on the date of last contact (for early study discontinuation)<br>b) 23:59 on the date of relative Day 29 Post-dose 1<br>c) 23:59 on planned visit day of dose 2 administration (in case dose 2 is not administered)<br>d) one minute prior to dose 2 administration □<br>e) 23:59 on the date of database cut-off (in case of interim analysis)
Post-dose 1 FU		One minute after the end of the Post-dose 1 period	Minimum of:<br>a) 23:59 on the date of last contact (for early study discontinuation or completion)<br>b) one minute prior to dose 2 administration<br>c) 23:59 on the date of database cut-off (in case of interim analysis)
Regimen*	Post-dose 2	Date and time of dose 2 administration	Minimum of:<br>a) 23:59 on the date of last contact (for early study discontinuation or completion)<br>b) 23:59 on the date of relative Day 29 Post-dose 2<br>c) 23:59 on the date of database cut-off (in case of interim analysis)<br><br>**Note:** subjects who do not receive dose 2 will not have a Post-dose 2 period.
Post-dose 2 FU		One minute after the end of the Post-dose 2 period	Minimum of:<br>a) 23:59 on the date of database cut-off (in case of interim analysis)<br>b) 23:59 on the date of last contact (for early study discontinuation or completion)

□For dose 2 administered earlier than Day 29 Post-dose 1 visit.
Note 1: * Regimen period includes both the Post-dose 1 and Post-dose 2 periods.
Note 2: FU = follow-up.

2.2. Pooling Algorithm for Analysis Centers

There are 2 parts in this study and each part will primarily be evaluated separately. For each part, data from different study sites (if applicable) will be pooled for analysis. In addition, the study data will be analyzed based on the pooled (ie, 14-day interval vaccination schedule) data from

Parts 1 and 2 of the study, matching the cohorts (ie, healthy and HIV-infected subjects) and regimen (ie, vaccine sequence and interval).

2.3. Analysis Sets

Full Analysis Set

The full analysis set includes all subjects who were randomized and received at least 1 dose of study vaccine (Ad26.ZEBOV, MVA-BN-Filo or placebo), regardless of the occurrence of protocol deviations.

Immunogenicity Analysis Set

The immunogenicity analysis set includes all randomized and vaccinated subjects, who have at least 1 post-vaccination (ie, after the date of vaccination) evaluable immunogenicity sample.

Per Protocol Analysis Set

The per protocol analysis set includes all randomized and vaccinated subjects, who received both the dose 1 and dose 2 vaccinations (administered within the protocol-defined visit window), have at least 1 post-vaccination (ie, after the date of vaccination) evaluable immunogenicity sample, and have no major protocol violations influencing the immune responses.

The primary immunogenicity analysis will be performed on the per protocol analysis set. The analysis on the immunogenicity analysis set will be included as sensitivity analysis only if more than 10% of the subjects are excluded from the per protocol analysis set. For subjects who received dose 1 (ie, only first vaccination) but not dose 2 while continuing their planned visit schedule, the immune response measurements after the planned (ie, upper limit of the analysis visit window that covers the target day for the second vaccination) but not administered dose 2 will not be included in graphs and tables showing descriptive statistics. These measurements will however be shown in listings, together with the indication that they are not used in the analysis.

2.4. Definition of Subgroups

There are 2 parts (Part 1: USA, Part 2: Africa) in the study with each part having the healthy and HIV-infected cohort of subjects aged 18-70 years. The subgroups that will be used to summarize the data are shown in Table 4. Primarily, the analysis of subject information, safety and immunogenicity analyses will be performed by part and cohort. Within each combination of part, cohort and vaccination (dose 1 and dose 2) interval, subject information, safety and immunogenicity data will be presented for active vaccines (ie, vaccine sequence and interval) versus placebo.

Secondly, Part 2 data (ie, ELISA [units/mL] and VNA [IC_{50} titers]) will be presented by age stratum and on the pooled cohorts (ie, healthy and HIV-infected).

In addition, data from Parts 1 and 2 will be pooled (matching on cohort and vaccination schedule).

For each combination of cohort and vaccine sequence, the pooled data (subject information, ELISA [units/mL], VNA [IC_{50} titers] and safety) will be presented for active vaccine versus placebo. The pooled immunogenicity data (ELISA [units/mL] and VNA [IC_{50} titers]) will also be presented by age stratum (aged 18-50 years and aged 51-70 years) for active vaccine versus placebo. Furthermore, these data will be presented on the pooled cohorts (healthy and HIV-infected).

Table 4: Study Part, Cohort, Dose 1-Dose 2 Interval and Age Group Combinations and Parameters

Study Part	Dose 1-Dose 2 Interval	Cohort	Age Group	Analysis Parameters
Part 1	14-day interval	Healthy	18-70 years	- Subject information<br>- ELISA (units/mL)<br>- VNA (IC_{50} titers)<br>- ELISpot<br>- ICS<br>- Ad26 VNA (Part 2 only)<br>- Safety
		HIV-infected		
Part 2	14-day interval	Healthy	18-70 years	
		HIV-infected		
		Healthy	18-50 years	- ELISA (units/mL)<br>- VNA (IC_{50} titers)
			51-70 years	
		HIV-infected	18-50 years	
			51-70 years	
		Pooled Healthy and HIV-infected	18-70 years	
	28-day interval	Healthy	18-70 years	- Subject information<br>- ELISA (units/mL)<br>- VNA (IC_{50} titers)<br>- ELISpot<br>- ICS<br>- Ad26 VNA (Part 2 only)<br>- Safety
		HIV-infected		
		Healthy	18-50 years	- ELISA (units/mL)<br>- VNA (IC_{50} titers)
			51-70 years	
		HIV-infected	18-50 years	
			51-70 years	
		Pooled Healthy and HIV-infected	18-70 years	
Pooled Parts 1 and 2	14-day interval	Healthy	18-70 years	- Subject information<br>- ELISA (units/mL)<br>- VNA (IC_{50} titers)<br>- ELISpot<br>- ICS<br>- Ad26 VNA (Part 2 only)<br>- Safety
		HIV-infected		
		Healthy	18-50 years	- ELISA (units/mL)<br>- VNA (IC_{50} titers)
			51-70 years	
		HIV-infected	18-50 years	
			51-70 years	
		Pooled Healthy and HIV-infected	18-70 years	

Note: There is currently no assay for the VNA (IC_{50} titers) of HIV-infected subjects.

3. CHANGES TO THE PLANNED ANALYSIS

- Even in the absence of baseline immunogenicity samples, the post-baseline samples are of interest. Therefore, the immunogenicity analysis set is redefined to include subjects without baseline immunogenicity samples, provided that at least 1 post-vaccination (ie, after the date of vaccination) evaluable immunogenicity sample is available for the subjects.

- Because different levels of Post-dose 2 immune responses are expected for different vaccination schedules, the per protocol analysis set is redefined to exclude subjects whose second vaccination falls outside the protocol-defined window. Similar to the immunogenicity analysis set, the per protocol analysis set is also refined to include subjects without baseline immunogenicity samples

- Because of the lack of the necessary data to assess non-adherence, the planned additionally analysis restricted to HIV-infected subjects who adhere to antiretroviral therapy (ART) if more than 10% of the HIV-infected subjects exhibit non-adherence to ART will not be performed.

4. INTERIM ANALYSIS AND DATA MONITORING COMMITTEE REVIEW

An interim analysis was conducted when all subjects in Part 1 had completed the 21-day post second vaccination visit or discontinued earlier. The purpose of this interim analysis was to evaluate the effect of vaccination on the HIV viral load of HIV-infected subjects from a safety perspective. The independent statistical support group (SSG) performed this interim analysis and sent a blinded data package to the protocol safety review team for review. The SSG also prepared an unblinded data package for the independent data monitoring committee (IDMC) review and recommendations. The details of that interim analysis were provided in a separate IDMC charter[3] and the associated IDMC SAP[4]. The current SAP is applicable to the final analysis (ie, when all subjects in Parts 1 and 2 have completed the last study-related visit or discontinued earlier).

Ad hoc IDMC reviews could be requested by the sponsor for any single event or combination of multiple events which were considered to jeopardize the safety of the subjects. For all cases, the data package and analysis results that would have contained any piece of unblinding information would have been kept in a strictly confidential place, with access for IDMC and SSG members only until unblinding of the study by the sponsor.

5. SUBJECT INFORMATION

Subject information will be analyzed based on the full analysis set. In general, the analysis will be presented (see Table 4) by part, cohort and pooled (ie, Parts 1 and 2 data of the MVA-BN-Filo followed by Ad26.ZEBOV vaccination schedule).

5.1. Disposition Information

The number and percentage of subjects randomized, vaccinated and entered in each analysis period will be tabulated. Subject assignment to vaccine regimen will be provided in a data listing (including the assigned regimen and the actual regimen received).

Furthermore, the number and percentage of subjects in the full analysis set who completed and those who discontinued together with the reason(s) for discontinuation will be tabulated and listed. This will be done for completion/discontinuation from second vaccination and from the trial.

5.2. Protocol Deviations

Subjects with protocol deviations will be identified prior to the database lock. The major protocol deviations will be summarized by deviation category. A listing of the major protocol deviations will also be generated. The major protocol deviations that have the potential to influence immune responses will be flagged in the listing.

5.3. Demographics and Baseline Characteristics

The following demographic characteristics will be summarized.

- Sex (Female/Male)
- Age (years)
- Age group (18-50 years versus 51-70 years)
- Race
- Ethnicity
- Height (cm)
- Weight (kg)
- Body mass index (BMI, kg/m^2), calculated from baseline height and weight
- HIV type (HIV serotype 1, HIV serotype 2), only for HIV-infected subjects

5.4. Prior and Concomitant Medications

The analysis of pre-study and concomitant therapies will be based on the World Health Organization (WHO) drug coded terms as provided in the clinical database. If the coded term for a concomitant medication is missing, then the reported term will be used and flagged in the table. The concomitant therapies will be tabulated per period. Additionally, a listing of all pre-study and concomitant therapies will be provided. There will be special attention to analgesics/antipyretics (such as acetaminophen, non-steroidal anti-inflammatory drugs [NSAIDs] and aspirin) administered during the first 8 days (including the day of the vaccination) following each vaccination. Special attention will also be given to HIV-related concomitant therapies.

Based on their start and stop dates, concomitant therapies will be reported in each analysis period during which they were applied. For missing or partial start/stop dates the following allocation rules will be applied:

- In case of partial start or stop dates, the concomitant therapy records will be allocated to analysis periods using the available partial information, without imputations. If, for example, only month and year are available, these will be compared to the month and the year of the analysis periods, and the concomitant therapy record will be allocated to the period(s) where these date parts match.
- In case of a completely missing start date, the concomitant therapy will be considered as having started before the trial.
- In case of a completely missing end date, the concomitant therapy will be considered as ongoing at the end of the trial.

Remark: In addition to the date information, time information is considered to allocate concomitant therapies to periods and/or phases, if available.

6. IMMUNOGENICITY

6.1. Endpoints

Primary Endpoint:

Magnitude of humoral immune responses against EBOV GP (as measured by binding antibody levels using ELISA [in ELISA units/mL] until the 21-day Post-dose 2 visit).

Exploratory Outcomes:

Several exploratory objectives are specified in the CTP[1]. Those objectives will be investigated depending on the availability of the response outcome. The exploratory response outcomes could include but may not be limited to the following:

- Humoral immune responses against EBOV GP as measured by:
  - ELISA (in ELISA units/mL), collected at all timepoints (as specified in the CTP[1]).
  - Neutralizing antibody response in titers that inhibit viral infection by a certain percentage (IC_{50}), collected at all timepoints (as specified in the CTP[1]).
- Cellular immune responses against EBOV GP as measured by:
  - Number of interferon (IFN-γ) producing T cells (using enzyme-linked immunospot [ELISpot]), at all available timepoints.
  - Percentage of CD4+ T cells producing IFN-γ or interleukin (IL-2) (using intracellular cytokine staining [ICS]), at all available timepoints.
  - Percentage of CD8+ T cells producing IFN-γ or IL-2 (using ICS), at all available timepoints.
- Anti-vector neutralizing antibody responses as measured by Ad26 virus neutralization assay (VNA) (serum titers of neutralizing antibodies to the adenovirus serotype 26 [Ad26] vector 90% inhibitory concentration [IC_{90}]), collected at baseline.

6.2. Immunogenicity Against the Insert

6.2.1. Humoral Immune Responses

6.2.1.1. Parameters

Humoral immune responses, as measured by the following assay, will be analyzed:

- **Binding antibody responses using Filovirus Animal Nonclinical Group (FANG) ELISA:** Quantification of antibodies binding to EBOV GP using the ELISA units/mL readout.

In addition, the following will be defined for ELISA (ELISA units/mL) binding antibody responses:

  - **Sample interpretation:** A sample will be considered positive, if the value is above the lower limit of quantification (LLOQ).
  - **Responder:**
    - If sample interpretation is negative at baseline but positive post-baseline and the post-baseline value is greater than 2.5×LLOQ; OR
    - If sample interpretation is positive at both baseline and post-baseline and there is a greater than 2.5-fold increase from baseline (2.5-fold increase on the original scale).

- **Neutralizing antibody responses using virus neutralization assay (VNA):** titers of EBOV GP-specific neutralizing antibodies (unit: 50% inhibitory concentration [IC_{50}]).

For VNA (IC_{50} titer) responses, the following will also be defined:

  - **Sample interpretation:** A sample will be considered positive if the value is greater than both the assay-specific LLOQ and 3x(amphotropic murine leukemia virus [aMLV]) titer. Otherwise, the sample will be considered negative.
  - **Responder**:
    - If sample interpretation is negative at baseline but positive post-baseline and the post-baseline value is greater than 2×LLOQ; OR
    - If sample interpretation is positive at both baseline and post-baseline and there is a greater than 2-fold increase from baseline (2-fold increase on the original scale).

Important: The pseudovirions used in the pseudo VNA (psVNA) are constructed using an HIV-genomic luciferase vector. During assay validation, a high non-EBOV GP-specific background signal was observed for 1 out of 3 HIV-positive sera tested. This could be due to the subjects' antiretroviral therapy interfering with the HIV-genomic luciferase vector-based pseudovirion assay. There is currently an ongoing exploration of assay optimization to improve the sensitivity for the sera from HIV-infected subjects. If assay optimization would not be possible, then an alternative psVNA or a wild-type VNA may be considered for HIV-infected subjects' sera sample analysis. Therefore, the analysis of psVNA described in this SAP will not be applicable to the HIV-infected subjects. The analysis description of the psVNA of HIV-infected subjects will only be made when the optimum (or alternative) assay is found.

6.2.1.2. Data Handling Rules

For ELISA binding antibody responses, values below the LLOQ will be imputed with LLOQ/2. For the calculation of fold increases, the values below LLOQ will be imputed with the LLOQ. For VNA titers, values less than the assay-specific LLOQ or less than 3×aMLV will be imputed with half of the assay-specific LLOQ. For the calculation of fold increases, values that are less than the assay-specific LLOQ or less than 3×aMLV will be imputed with the assay-specific LLOQ.

Remark: If an aMLV titer (negative control) is a censored value (ie, <**40)**, then it will be imputed with **40** before proceeding with further computations.

6.2.1.3. Analysis Methods

The humoral immune responses (immunogenicity against EBOV GP) will be evaluated separately for each part of the study. Within each part, the data will primarily be presented by vaccination schedule (ie, vaccine sequence and interval) with a distinction between the healthy and HIV-infected cohorts. In addition, the data from Parts 1 and 2 will be pooled (matching cohort and vaccination schedule) and presented by vaccination schedule. Also, the pooled immunogenicity data (ie, ELISA units/mL and VNA (IC_{50})) will be presented by age stratum (aged 18-50 years and aged 51-70 years) for each vaccination schedule. Furthermore, the pooled immunogenicity data (ELISA units/mL and VNA (IC_{50})) will be presented by vaccination schedule without a distinction between the cohorts (ie, healthy and HIV-infected). See Section 2.4 (and Table 4) for details on the subgroup analyses that will be performed.

Except for VNA analysis of the HIV-infected subjects, the humoral immune responses will be analyzed as follows. Summary statistics (ie, geometric mean and corresponding 95% CIs) will be calculated and presented for ELISA binding antibody responses (ELISA units/mL) and VNA titers (IC_{50}) at each time point. The geometric mean fold increase (from Pre-dose and Pre-dose 2) with corresponding 95% CI will also be presented per time point for these parameters.

Graphical representations (on a $\log_{10}$-scale) of regimen profiles using the geometric mean concentrations with 95% CIs will be presented by vaccination schedule. Regimen profiles of the geometric mean concentrations with 95% CIs will also be generated by vaccination schedule and pre-existing antibody response against Ad26 vector (ie, pre-existing Ad26 versus not pre-existing Ad26). In addition, graphs of the reverse cumulative distributions (ie, percentage of subjects versus the magnitude of the antibody response levels) will be provided for the following time points, if available:

- Baseline and Pre-dose 2
- Baseline and 21 days Post-dose 2
- Baseline and 42 days Post-dose 2
- Baseline and 180 days Post-dose 2
- Baseline and 365 days Post-dose 2

For both ELISA (ELISA units/mL) and VNA (IC_{50} titer), additional graphical representations (on a log_{10}-scale) will be provided using dot plots (with distinction between positive and negative sample interpretations), by vaccination schedule.

Responder rates and positive sample interpretation will be summarized (ie, showing number, percentage and the exact 95% Clopper-Pearson CI) for ELISA (ELISA units/mL) antibody responses and VNA (IC_{50}) titers per time point.

Furthermore, response patterns over time for the humoral immune responses will be analyzed, taking into account the within-subject correlations, to describe differences between the vaccination schedules at the 21-day Post-dose 2, 42-day Post-dose 2, 6-month Post-dose 2 and 1-year Post-dose 2 visits. A linear mixed model with the unstructured variance-covariance and an unstructured mean (ie, including time as a categorical variable) will be used to estimate the geometric mean concentrations and the associated 95% CI at these timepoints. In case of model convergence problems, another variance-covariance structure (eg, heterogeneous compound symmetry, compound symmetry or simple) will be used.

6.2.2. Cellular Immune Responses

6.2.2.1. Parameters

Cellular immune responses, as measured by the following assays, may be analyzed:

- **Enzyme-linked immunospot (ELISpot):** EBOV GP-specific IFN-γ producing T cells, measured as the number of spot-forming units per million peripheral blood mononuclear cells (SFU/10^6 PBMC).

The following will be defined for ELISpot:

  - **Sample interpretation:** Sample positivity criteria will be defined by vendor based on the assay characteristics. A false positivity criterion of less than 10% for naïve samples (from non-vaccinated subjects) will be applied.
  - **Responder:**
    - If sample interpretation is negative at baseline but positive post-baseline and the post-baseline value is greater than 2× positivity threshold; OR
    - If sample interpretation is positive at both baseline and post-baseline and there is a greater than 2-fold increase from baseline.

- **Intracellular cytokine staining (ICS):** The following responses will be measured:
  - CD4+: IL-2+ and IFN-γ+ responses to EBOV GP.
  - CD8+: IL-2+ and IFN-γ+ responses to EBOV GP.

In addition, using IFN-γ+ and IL-2+ as present in the database for each T cell type, the following will be defined for the ICS:

- **Sample interpretation:** The sample positivity (interpretation) will be determined for each of the two peptide pools separately. If a sample is considered positive for at least one of the peptide pools the sample is considered positive. If a sample is positive for either IFN-γ+ or IL-2+, it is considered positive. A sample is considered positive when the EBOV peptide pool stimulated readout is greater than 3-fold the mock (unstimulated) readout and the mock-subtracted value is greater than the threshold (limit of detection (LOD)).
- **Responder:** The responder status of a subject will be determined based on IFN-γ+ and IL-2+ separately. This will be done on the mock subtracted percentages for the combined peptide pools (ie, sum of GP1 and GP2). For a given time point, if a responder status is achieved based on either IFN-γ+ or IL-2+, then the subject will be declared as a responder at that time point. For a given time point, a subject is considered as a responder when either of the following condition is satisfied:
  - If sample interpretation is negative at baseline but positive post-baseline and the post-baseline value is greater than 2×LOD; OR
  - If sample interpretation is positive at both baseline and post-baseline and there is a greater than 2-fold increase from baseline in background adjusted percentage of the combined peptide pools.

For additional rules for the ICS analysis refer to Attachment 52.5.

6.2.2.2. Data Handling Rules

For ELISpot, values below the positivity threshold will be imputed with half of the threshold.

For the calculation of fold increases, ELISpot values below the positivity threshold will be imputed with the threshold. Fold increases will not be calculated for ICS.

6.2.2.3. Analysis Methods

The cellular immune responses will be evaluated separately for each part of the study. Within each part, the data will primarily be presented by vaccination schedule with a distinction between the healthy and HIV-infected cohorts. The cellular data will not be presented by age stratum. Also, the cellular data for healthy and HIV-infected cohorts will not be combined for analysis. See Table 4 for details.

Summary statistics (ie, median, quartiles [Q1, Q3]) will be presented for all continuous cellular immunogenicity outcomes (ie, ELISpot and ICS) at each time point. Also, the median fold increases (from Pre-dose 1 and Pre-dose 2) with the corresponding quartiles (Q1 and Q3) will be presented for ELISpot only.

Graphical representations (on a $\log_{10}$-scale) will be provided using dot plots, by vaccination schedule. Also, regimen profiles of the vaccination schedules showing the median and the quartiles (Q1 and Q3) will be presented. Other profiles (median and quartiles) will be generated by pre-existing antibody response against Ad26 vector (ie, pre-existing Ad26 versus not pre-existing Ad26 vector). Responder and positive sample interpretation rates will be summarized

(ie, showing number, percentage and the corresponding exact 95% Clopper-Pearson CIs) per timepoint.

For ICS, the proportions of EBOV GP-specific CD4+ and CD8+ T cells (ie, cells producing at least 1 of the 3 investigated cytokines) will also be tabulated per timepoint and shown in a pie chart. The magnitude of each cytokine subset will be shown in a bar chart. Both pie chart and bar chart will be restricted to responders.

6.3. Immunogenicity Against the Vector

6.3.1. Humoral Immune Responses

6.3.1.1. Parameter

Immune responses, as measured by the Ad26 VNA (serum titers of neutralizing antibodies to the Ad26 vector [IC_{90}]) will be analyzed. The following will also be defined for Ad26 VNA titers:

Sample interpretation: a sample will be considered positive, if the value is above the assay-specific LLOQ.

6.3.1.2. Data Handling Rules

For the outcome (ie, Ad26 VNA), values below the LLOQ will be imputed with half of the LLOQ (LLOQ/2).

6.3.1.3. Analysis Methods

The humoral immune responses (ie, immunogenicity against Ad26 vector) will be evaluated separately for each part of the study. Within each part, the data will primarily be presented by cohort (ie, healthy and HIV-infected) and vaccination schedule.

The geometric mean and corresponding 95% CIs will be calculated. Positive sample interpretation will be summarized (ie, showing number, percentage and the exact 95% Clopper-Pearson CI). A data listing will also be generated.

7. SAFETY

The safety and tolerability data include the following:

- AEs (unsolicited AEs) collected from signing of the informed consent form (ICF) onwards until the 42-day Post-dose 2 visit.
- Serious adverse events (SAEs) and immediate reportable events (IREs) collected from signing of the ICF onwards until the end of the study.
- Solicited local and systemic AEs (reactogenicity) collected until 7 days after each administration of study vaccine.

The safety and tolerability data will be summarized based on the full analysis set. The analysis will be based on the actual dose (ie, dose 1 and dose 2 for the first and second vaccinations,

respectively) that the subjects received. For example, if dose 2 is not administered to a subject, then that subject will not be included in the analysis of AEs in the Post-dose 2 period. Focus will be on safety signals detected during the Post-dose 1 and Post-dose 2 periods, as well as the Regimen period (ie, combined Post-dose 1 and Post-dose 2 periods).

In general, safety data will be evaluated separately for each part of the study. Within each part, the data will primarily be presented by cohort (healthy and HIV-infected) and by vaccination schedule. Secondarily, the data from Parts 1 and 2 of the study will be pooled (ie, 14-day interval regimen) and presented separately for the healthy and HIV-infected cohorts. See Table 4 for a schematic overview the subgroups to be considered for the safety analysis.

7.1. Adverse Events

The analysis of AEs is based on the medical dictionary for regulatory activities (MedDRA) coded terms as provided in the clinical database. All reported AEs (solicited local, solicited systemic, and unsolicited) during the vaccination periods (Post-dose 1 and Post-dose 2) (ie, AEs following vaccination and AEs that have worsened since baseline) will be included in the analysis. Listings of AEs will include all reported AEs.

Note that the AEs include any occurrence that is new in onset or aggravated in severity, toxicity grade or frequency from the baseline condition, or clinically relevant abnormal results of diagnostic procedures, including clinically relevant laboratory test abnormalities.

7.1.1. Definitions

Solicited AEs are precisely defined events (local and systemic) that subjects are specifically asked about and which are noted by subjects in the diary. All other AEs are considered unsolicited. Refer to Sections 9.3 and 12.1.1 of the CTP[1] for further details.

Solicited Local (Injection Site) Reactions

The analysis of local solicited AEs will include:

- Pain/Tenderness
- Erythema
- Induration/Swelling
- Itching

Solicited Systemic Adverse Events

The analysis of systemic solicited AEs will include:

- Fever (defined as body temperature of 38°C or higher)
- Headache
- Fatigue/Malaise
- Myalgia

- Nausea/Vomiting
- Arthralgia
- Chills

Serious Adverse Events

SAEs will be collected from signing of the ICF until the end of the study. An SAE based on the International Council for Harmonisation (ICH) and the European Union (EU) Guidelines on Pharmacovigilance for Medicinal Products for Human Use is any untoward medical occurrence that at any dose:

- Results in death
- Is life-threatening (the subject was at risk of death at the time of the event. It does not refer to an event that hypothetically might have caused death if it were more severe.)
- Requires inpatient hospitalization or prolongation of existing hospitalization
- Results in persistent or significant disability/incapacity
- Is a congenital anomaly/birth defect
- Is a suspected transmission of any infectious agent via a medicinal product
- Is medically important*.

*Medical and scientific judgment should be exercised in deciding whether expedited reporting is also appropriate in other situations, such as important medical events that may not be immediately life-threatening or result in death or hospitalization but may jeopardize the subject or may require intervention to prevent one of the other outcomes listed in the definition above. These should usually be considered serious.

Immediate Reportable Events

The following list of neuroinflammatory disorders are categorized as IRE, and should be reported to the sponsor within 24 hours after becoming aware of the event using the IRE Form. Relevant data from the IRE form will be captured in the clinical database.

- Cranial nerve disorders, including paralyses/paresis (eg, Bell’s palsy)
- Optic neuritis
- Multiple sclerosis
- Transverse myelitis
- Guillain-Barré syndrome, including Miller Fisher syndrome, Bickerstaff’s encephalitis and other variants
- Acute disseminated encephalomyelitis, including site specific variants: eg, non-infectious encephalitis, encephalomyelitis, myelitis, myeloradiculomyelitis
- Myasthenia gravis and Lambert-Eaton myasthenic syndrome

– Immune-mediated peripheral neuropathies and plexopathies (including chronic inflammatory demyelinating polyneuropathy, multifocal motor neuropathy and polyneuropathies associated with monoclonal gammopathy)
– Narcolepsy
– Isolated paresthesia of >7 days duration

Causality

The solicited local AEs will be considered as related to the study vaccine, by definition. An unsolicited and solicited systemic AEs will be considered related to the use of the study vaccine if the attribution is possible, probable or very likely. An AE will be considered not related with the use of the study vaccine if the attribution is not related or doubtful. Refer to Section 12.1.2 of the CTP[1], for further details.

Severity Criteria

The severity of the AEs is classified by the investigator as mild, moderate or severe using the Division of Microbiology and Infectious Diseases (DMID) Toxicity Table for Use in Trials Enrolling Healthy Adults (Attachment 3).

Solicited events that are graded less than mild, are not considered AEs. For some solicited events (eg, induration/swelling), the diameter and grading (as reported by the investigator [ie, functional grade]) are collected in the electronic case report form (eCRF). The diameter will be used to derive the toxicity grading. The worst grade should be used when both the diameter derived grade and the functional grade are available. If only the functional grade is available, then this should be used

7.1.2. Data Handling Rules

Missing data will not be imputed. If severity or relationship of AEs to the study vaccine could not be derived (ie, missing or unknown), it will be considered as unknown, for analysis purposes. Local solicited AEs will be considered as related with the use of the study vaccine, by definition.

Solicited events will always be allocated to the analysis period (Section 2.1). For analysis purpose, the AEs will be allocated to periods and/or phases as described in Attachment 2.1.

7.1.3. Analysis Methods

In general, the AEs following vaccination will be summarized (ie, tables of descriptive statistics) by vaccination schedule and presented per period/phase. Similar summary tables will also be provided pooled by vaccine (dose).

Furthermore, unsolicited AEs will be summarized (showing number and percentage) by System Organ Class (SOC) and Preferred Term (PT). Solicited AEs (recorded by day) will be converted into the analysis format of unsolicited AEs (recorded by event), as detailed in Attachment 2. These solicited AEs will be summarized by class (local, systemic) and Preferred Term. For

solicited as well as unsolicited AEs, tables focusing on severity will be created. Focus will also be on the relationship (to the study vaccine) of the solicited systemic and unsolicited AEs.

The SAEs, AEs with fatal outcome, AEs leading to permanent stop of study vaccination, Grade 3 AEs and IREs will also be listed. A table summarizing all those parameters will further be created and presented per analysis period and vaccination schedule. Summary tabulations by SOC and PT for each of the category of events (ie, SAEs, AEs with fatal outcome, AEs leading to permanent stop of study vaccination, Grade 3 AEs and immediate reportable events) will also be generated on the entire reporting period. Subject narratives will be generated for these events, except Grade 3 AEs.

For the most frequent (at least 10% of subjects in any vaccination schedule) solicited local and systemic AEs, the duration and time to first onset of the events will also be summarized. If a subject experiences more than 1 occurrence of a solicited event, the maximum duration of the events will be used. The time to first onset is defined as:

$$[date\ of\ first\ onset - reference\ date + 1]$$

The reference date is the start date of each vaccination period (ie, Post-dose 1 or Post-dose 2). Duration and time to onset of AEs will be expressed in days.

7.2. HIV Viral Load Assessment and CD4+ Cell Counts

HIV viral load and CD4+ cell counts will be evaluated for HIV-infected subjects. For analysis, the HIV viral load assessment at each time point will be categorized as follows:

- **<20 copies/mL:** Both undetectable (ie, values below the limit of detection ["NOT DETECTED"]) and non-quantifiable (ie, <20 copies/mL) HIV viral load.
- **20-200 copies/mL:** HIV viral loads ranging from 20 to 200 copies/mL.
- **>200 copies/mL:** More than 200 copies/mL of HIV viral load.

A summary table will be generated based on the above categorization of the HIV viral loads. The table will show the number and percentage of subjects under each category at baseline and post-baseline time points and by vaccination schedule. A cross tabulation will also be generated for those categories. The cross tabulation will show the number and percentage of subjects in each category at baseline against other time points.

Subject-specific profiles will be generated for the HIV viral load and CD4+ cell counts. The graphs will be generated separately for those subjects whose baseline HIV viral load falls within a category (ie, <20 copies/mL, 20-200 copies/mL and >200 copies/mL).

In addition, a listing (also showing all the HIV viral loads and CD4+ cell count assessments) will be generated.

7.3. Clinical Laboratory Tests

This section concerns the clinical laboratory test data (except HIV viral loads and CD4+ cell counts in HIV-infected subjects). The analysis of the laboratory assessments will be based on the Food and Drug Administration (FDA)'s Toxicity Grading Scale for Healthy Adult and Adolescent Volunteers Enrolled in Preventive Vaccine Clinical Trials (Attachments 3 and 4). In case no toxicity grades are defined for a test, the abnormalities above or below the normal range will be used.

It is important to note that clinically relevant changes occurring during the study are recorded in the AE section of the eCRF up to 42 days post dose 2 visit.

7.3.1. Definitions

In determining toxicity grades, the following rules will be applied:

- Worst grades/abnormalities are determined over the entire period (eg, Post-dose 1 or Post-dose 2) separately, including all post-baseline measurements of the corresponding period.
- The abnormalities "abnormally low" and "abnormally high" are considered equally important and both abnormalities are shown in the tables. (This means that the sum of the percentages can exceed 100%).
- If a laboratory value falls within the grading as specified in the grading table but also within the local laboratory normal limits, the value is considered as normal or Grade 0.
- Laboratory results falling between the grading scales will be allocated to the adjacent worst-case grade (because the scale for some parameters in the grading table is not continuous as there may be zones where toxicity grade definitions do not exist).

An abnormality (toxicity grade or abnormality based on normal ranges) will be considered as following vaccination in a period and/or phase if it is worse than the corresponding baseline record. If the baseline value is missing, the abnormality is always considered as following vaccination. A shift from "abnormally low" at baseline to "abnormally high" post-baseline (or vice versa) is also considered as an abnormality following vaccination.

7.3.2. Data Handling Rules

In case a laboratory test result is *censored* (no numeric value is available, but only a verbatim term) then a numeric value will be imputed:

- For integer x:
  - If the value is $<x$ then impute with x-1.
  - If the value is $>x$ then impute with x+1.
- For mantissa (decimal part) x:
  - If the value is $<y.x$ then impute with $y.x$-0.1 (if x has 1 decimal place of precision).
  - If the value is $>y.x$ then impute with $y.x$+0.1 (if x has 1 decimal place of precision).

Remark: The value added or subtracted from the mantissa should follow from its mantissa.

Example:

If value is <5 or >10 impute with 4 and 11 respectively.<br>If value is <5.3 or >10.7 then impute with 5.2 and 10.8 respectively.<br>If value is <5.32 or >10.73 then impute with 5.31 and 10.74 respectively.

7.3.3. Analysis Methods

Laboratory abnormalities will be determined in accordance with the toxicity grading tables (Attachments 3 and 4), and in accordance with the normal ranges of the clinical laboratory. The worst abnormalities following vaccination will be summarized (ie, showing number and percentage) by vaccination schedule and presented per period/phase, with special attention to Grade 3 toxicities. Focus will be on clinical abnormalities that occur during Post-dose 1 and Post-dose 2 periods, as well as the Regimen period (ie, combined Post-dose 1 and Post-dose 2 periods). Similar tables for worst abnormalities will be provided pooled by vaccine (dose). A listing will also be provided for subjects with clinically significant abnormal laboratory findings.

7.4. Vital Signs and Physical Examination Findings

Vital sign abnormalities will be determined in accordance with the DMID Vital Signs Toxicity Grading (Attachment 3). The worst abnormalities (following vaccination) of vital signs will be tabulated (ie, showing number and percentage) and listed. Focus will be on the abnormalities that occur during the Post-dose 1 and Post-dose 2 periods, as well as the Regimen period (ie, combined Post-dose 1 and Post-dose 2 periods).

It is important to note that a full physical examination is only conducted at screening. At other visits, only abbreviated, symptom-directed examinations are performed per the investigator’s discretion. Therefore, only a listing of subjects with clinically significant physical examination findings (ie, abnormalities) will be provided.

Also, any clinically relevant vital signs or physical examination abnormalities occurring from signing of the ICF onwards until 42-day post dose 2 vaccination are recorded on the AE page of the eCRF and will be analyzed as AE.

7.5. Electrocardiogram

Note that a single, 12-lead electrocardiogram will be performed at screening for subjects >50 years and interpreted locally. Additional ECG monitoring could be done at other time points during the study only if clinically indicated based on signs and symptoms. Therefore, only a listing of subjects with clinically significant electrocardiogram abnormality will be generated.

8. REFERENCES

1. Clinical Protocol VAC52150EBL2003 Amendment 5: A Randomized, Observer-blind, Placebo-controlled, Two-part, Phase 2 Study to Evaluate the Safety, Tolerability and Immunogenicity of Two Prime-boost Regimens of the Candidate Prophylactic Vaccines for Ebola Ad26.ZEBOV and MVA-BN-Filo. Walter Reed Army Institute of Research and Janssen Vaccines & Prevention B.V. (September 2017).
2. Horton H, Thomas EP, Stucky JA, et al. Optimization and Validation of an 8-Color Intracellular Cytokine Staining (ICS) Assay to Quantify Antigen-Specific T Cells Induced by Vaccination. J Immunol Methods. 2007;323(1):39–54.
3. VAC52150EBL2003-Independent Data Monitoring Committee Charter. Walter Reed Army Institute of Research and Crucell Holland B.V. (November 2015).
4. VAC52150EBL2003-Independent Data Monitoring Committee Statistical Analysis Plan Amendment 1. Walter Reed Army Institute of Research and Janssen Vaccines & Prevention B.V. (February 2017).

9. ATTACHMENTS

1. PERIOD ALLOCATION OF ADVERSE EVENTS

Solicited events will always be allocated to the Post-dose 1 or Post-dose 2 period, as appropriate.

Unsolicited AEs will be allocated to the different periods per the following rules:

<u>Step 1</u>: Allocation of unsolicited events to the periods/phases:

The AEs present in the database are allocated to periods/phases based on their start date/time. If the start date/time of an event falls between (or on) the start and stop date/time of a period/phase, the AE is attributed to that period/phase (ie, AEs following vaccination).

Incomplete dates (ie, time and/or day and/or month and/or year missing):

1) In case of partial start or stop dates, the events are allocated to the periods/phases using the available partial information on start and end date; no imputation will be done. If, for instance, for the AE start date only month and year are available, these data are compared to the month and year information of the periods/phases. This rule may lead to multiplication of the event because of its assignment to multiple periods/phases (see below example).
2) In case of a completely missing start date, the event will be allocated to the appropriate period/phase (eg, Post-dose 1 or Post-dose 2) and consequently the Regimen period; except if the end date of the AE falls before the start of the Post-dose 1 or Post-dose 2 period.

3) In case of a completely missing end date (ie, only for the calculation of duration):
    - In case the AE is flagged as ongoing the date is imputed by the cut-off date of the analysis for subjects still ongoing in the study, and by the end date of the last period/phase for subjects who discontinued or completed the trial.
    - In case the AE is not flagged as ongoing, the end date is considered as unknown, and the date will remain missing.

Examples:

Screening Phase: start date: 14JUN2016 - stop date: 28JUN2016<br>Post-dose 1 period: start date: 28JUN2016 - stop date: 19JUL2016<br><br>1) Adverse event: start date: JUN2016- stop date: 15JUL2016<br>As the start date only has information about month and year, only this information will be used from the periods/phases (ie, assuming any day of Jun is possible) and therefore the AE will be assigned to the Screening Phase as well as to the Post-dose 1 period.<br><br>2) Adverse event: start date: JUL2016- stop date 14JUL2016<br>As the AE starts after the Screening Phase and after the start of the Post-dose 1 period, it is only assigned to the Post-dose 1 period.

Remarks:

- In addition to the date information, time information is considered to allocate AEs to periods, if available.

- The imputation of missing end dates of ongoing AEs will only be used to derive the duration of the event (ie, to give an indication of the minimum duration). The imputed end dates will not be shown in the data listings.

***Step 2*: Combination of events:**

Overlapping/consecutive events are defined as events of the same subject with the same preferred term which have at least 1 day overlap or for which the start date of an event is 1 day after the end date of the preceding event. Overlapping/consecutive events may be combined into one AE or not, according to the following rules:

1) If overlapping/consecutive events start in a non-active phase (Screening or any of Post-dose FU phases) followed by an AE in an active (Post-dose 1 or Post-dose 2) period, they are allocated to their respective periods/phases and are considered as separate events.
2) In case overlapping/consecutive events start within a single period/phase, they are considered as one and the same AE. The individual events which contribute to this AE are retained as individual records in the Analysis Data Model (ADaM) database but are assigned the same onset, period/phase, and total duration.
3) In case overlapping/consecutive events start in an active period followed by a non-active phase, they are allocated only to the active period and are considered as one and the same AE. The individual events which contribute to this AE are retained as individual records in the ADaM database but are assigned the same onset, period, and total duration.
4) In case an active period is followed by another active period, and the overlapping/consecutive events start in both periods, they are allocated to their respective period and are considered as separate AEs. The same rule applies for 2 non-active phases.
   - Remarks:
     1. Time is not considered when determining overlap of events.
     2. Events can only be combined into one and the same AE if their start and stop dates are known.
     3. In case the completely missing end date is imputed (for calculation of duration), this date is also considered as a complete date.

   Examples:
   Screening phase: start date: 14JUN2016 - stop date: 28JUN2016
   Post-dose 1 period: start date: 28JUN2016 - stop date: 26JUL2016
   Post-dose 1 FU phase: start date: 27JUL2016 - stop date: 15AUG2016

   Example for the above Scenario 1
   AE1: start date: 20JUN2016- stop date: 10JUL2016
   AE2: start date: 08JUL2016- stop date: 18JUL2016
   AE1 will be attributed to the Screening Phase and AE2 to the Post-dose 1 period.

   Example for the above Scenario 3
   AE1: start date: 18JUL2016- stop date: 28JUL2016
   AE2: start date: 28JUL2016- stop date: 08AUG2016

As AE1 starts in the active period (Post-dose 1) and overlaps with AE2 which starts in a non-active phase (Post-dose 1 FU), this AE is considered as a single AE in the AE analysis starting on 18JUL2016 and ending on 08AUG2016 and is attributed to the Post-dose 1 period.

2. TRANSFORMING ON-SITE ASSESSMENTS AND DIARIES OF SOLICITED ADVERSE EVENTS INTO AN ANALYSIS FORMAT

When creating the analysis dataset for solicited AEs, solicited AEs (recorded by day) need to be converted into the format of unsolicited AEs (recorded by event). All diary data will be considered, as well as any post-dose on-site assessment (scheduled as well as unscheduled) within (including the day of vaccination) 8 days after vaccination. For solicited local AEs for which a diameter is measured, the maximum of diameter derived grade and functional severity grading (if available) will be used. The start date of the AE will be considered as the date of first occurrence of the solicited AE (both local and systemic). If on subsequent day(s), the same grade is reported, the last reported date is used as the end date of the AE. A new record is created in case the grade of the event changes. If there is a time gap of at least one day between two (or more) occurrences of the same type of the solicited AE, then the second (and/or next) occurrence will be considered as a new AE. In case no data is reported for a day, this is analyzed as no event reported. If the on-site assessment differs in grade or relatedness (if collected) with the corresponding diary data, only the highest grade and relatedness assessment indicating the highest relatedness to study vaccination per AE will be kept in the analysis database and used in the tables and listings. The following example shows how the solicited AE should be converted into a format of unsolicited AEs:

<u>Data from the Subject Diary</u>
Subject: 0001
Solicited systemic AE: Headache

	**On-site Assessment**	**Diary Data**							
Solicited AE	Day 1 01Jan16	Day 1 01Jan16	Day 2 02Jan16	Day 3 03Jan16	Day 4 04Jan16	Day 5 05Jan16	Day 6 06Jan16	Day 7 07Jan16	Day 8 08Jan16
Grade	2	1	1	0	3	3	1	0	0
Relatedness	Doubtful	Probable							

The data should be converted and stored in the AE dataset as follows:

**Subject No.**	**AE**	**Start Date (Char)**	**Stop Date (Char)**	**Severity**	**Relatedness**	**AEID**
0001	Headache	01Jan16	02Jan16	2	Probable	1
0001	Headache	04Jan16	05Jan16	3	Probable	1
0001	Headache	06Jan16	06Jan16	1	Probable	1

If a solicited AE ends after Day 8:

The stop date of the event is the "Date of last day of symptom" as recorded in the eCRF and the "maximum severity" after Day 8 as recorded in the CRF. A separate record is created for this, in case this severity deviates from the previous record.

Note: To complete the start and end-date based on diary data, the date will be calculated based on the day the AE is reported relative to vaccination and not on the reported date. For example, if the vaccination is on 01-JAN-2016, and the AE starts on Day 3, the start date will be set to the 03-JAN-2016, independent of the reported actual date.

For the calculation of duration, the first and last day is used, irrespective of whether interruptions occurred in between by missing reporting days or Grade 0 events. In the above example, the 4 records contribute to the same AE, therefore AEID is set to the same value and the duration of the AE is set to 6 for all records.

It is important to note that the occurrence of solicited systemic adverse events considered to be related to the study vaccine and persisting for at least 3 days and in at least 3 subjects will result in a study pause. However, the above rule for calculating duration of AEs may incorrectly indicate that the pausing rule is met. Therefore, a listing of subjects with Grade 3 (severe) AEs will be generated with an indication that interruption between AE start and AE end are not considered in the calculation of the duration of the AEs.

3. TOXICITY TABLES FOR USE IN TRIALS ENROLLING HEALTHY ADULTS

The abbreviations used in the following tables are:

ALT: alanine aminotransferase; aPTT: activated partial thromboplastin time; AST: aspartate aminotransferase; AV block: atrioventricular block; bpm: beats per minute; CK: creatine kinase; FEV_1: forced expiratory volume in 1 second; g: gram; HI: high; HPF: high power field; INR: international normalized ratio; IV: intravenous; LO: low; mEq: milliequivalent; mm Hg: millimeter of mercury; N: not graded; PT: prothrombin time; PTT: partial thromboplastin time; QTc: QT-interval corrected for heart rate; QTcB: Bazett's corrected QT interval; QTcF: Fridericia's corrected QT interval; RBC: red blood cell; Rx: therapy; ULN: upper limit of normal

CLINICAL ADVERSE EVENTS

Grading scale used for clinical adverse events is adapted from the Division of Microbiology and Infectious Diseases (DMID) Toxicity Tables (2014). For adverse events not included in the tables below, refer to the severity criteria guidelines in Section 12.1.3 of the CTP[1].

**Cardiovascular**	**Grade 1**	**Grade 2**	**Grade 3**
Arrhythmia		Asymptomatic, transient signs, no Rx required	Recurrent/persistent; symptomatic Rx required
Hemorrhage, blood loss	Estimated blood loss ≤100 mL	Estimated blood loss >100 mL, no transfusion required	Transfusion required
QTcF (Fridericia's correction)[a] or QTcB (Bazett's correction)	Asymptomatic, QTc interval 450-479 ms, *OR* Increase in interval <30 ms above baseline	Asymptomatic, QTc interval 480-499 ms, *OR* Increase in interval 30-60 ms above baseline[b]	Asymptomatic, QTc interval ≥500 ms, *OR* Increase in interval ≥60 ms above baseline
PR interval (prolonged)	PR interval 0.21-0.25 s	PR interval >0.25 s	Type II 2nd degree AV block *OR* Ventricular pause >3.0 s
**Respiratory**	**Grade 1**	**Grade 2**	**Grade 3**
Cough	Transient; no treatment	Persistent cough	Interferes with daily activities
Bronchospasm, acute	Transient; no treatment; FEV_1 71%-80% of peak flow	Requires treatment; normalizes with bronchodilator; FEV_1 60%-70% (of peak flow)	No normalization with bronchodilator; FEV_1 <60% of peak flow
Dyspnea	Does not interfere with usual and social activities	Interferes with usual and social activities, no treatment	Prevents daily and usual social activity or requires treatment
**Gastrointestinal**	**Grade 1**	**Grade 2**	**Grade 3**
Nausea/vomiting	Minimal symptoms; caused minimal or no interference with work,	Notable symptoms; required modification in activity or use of	Incapacitating symptoms; required bed rest and/or resulted in loss of work or

[a] Inclusion dependent upon protocol requirements.

[b] The Grade 2 increase in interval is changed from 30-50 ms to 30-60 ms since the original DMID Toxicity Tables (2014) did not cover the increase in interval between 50 and 60 ms.

	school or self-care activities	medications; did not result in loss of work or cancellation of social activities	cancellation of social activities
Diarrhea	2-3 loose or watery stools or <400 g/24 hours	4-5 loose or watery stools or 400-800 g/24 hours	6 or more loose or watery stools or >800 g/24 hours or requires IV hydration
**Reactogenicity**	**Grade 1**	**Grade 2**	**Grade 3**
Local reactions			
Pain/tenderness at injection site	Aware of symptoms but easily tolerated; does not interfere with activity; discomfort only to touch	Notable symptoms; required modification in activity or use of medications; discomfort with movement	Incapacitating symptoms; inability to do work or usual activities; significant discomfort at rest
Erythema/redness[a]	2.5-5 cm	5.1-10 cm	>10 cm
Induration/swelling[b]	2.5-5 cm and does not interfere with activity	5.1-10 cm or interferes with activity	>10 cm or prevents daily activity
Itching at the injection site	Minimal symptoms; caused minimal or no interference with work, school or self-care activities	Notable symptoms; required modification in activity or use of medications; did not result in loss of work or cancellation of social activities	Incapacitating symptoms; required bed rest and/or resulted in loss of work or cancellation of social activities
Systemic reactions			
Allergic reaction	Pruritus without rash	Localized urticaria	Generalized urticaria; angioedema or anaphylaxis
Headache	Minimal symptoms; caused minimal or no interference with work, school or self-care activities	Notable symptoms; required modification in activity or use of medications; did not result in loss of work or cancellation of social activities	Incapacitating symptoms; required bed rest and/or resulted in loss of work or cancellation of social activities
Fatigue/malaise	Minimal symptoms; caused minimal or no interference with work, school or self-care activities	Notable symptoms; required modification in activity or use of medications; did not result in loss of work or cancellation of social activities	Incapacitating symptoms; required bed rest and/or resulted in loss of work or cancellation of social activities
Myalgia	Minimal symptoms; caused minimal or no interference with work, school or self-care activities	Notable symptoms; required modification in activity or use of medications; did not result in loss of work or cancellation of social activities	Incapacitating symptoms; required bed rest and/or resulted in loss of work or cancellation of social activities

[a] In addition to grading the measured local reaction at the greatest single diameter, the measurement should be recorded as a continuous variable.

[b] Induration/swelling should be evaluated and graded using the functional scale as well as the actual measurement.

Arthralgia	Minimal symptoms; caused minimal or no interference with work, school or self-care activities	Notable symptoms; required modification in activity or use of medications; did not result in loss of work or cancellation of social activities	Incapacitating symptoms; required bed rest and/or resulted in loss of work or cancellation of social activities
**Reactogenicity**	**Grade 1**	**Grade 2**	**Grade 3**
Chills	Minimal symptoms; caused minimal or no interference with work, school or self-care activities	Notable symptoms; required modification in activity or use of medications; did not result in loss of work or cancellation of social activities	Incapacitating symptoms; required bed rest and/or resulted in loss of work or cancellation of social activities

LABORATORY TOXICITY GRADING

Grading scale used for lab assessments is based on 'FDA's Toxicity Grading Scale for Healthy Adult and Adolescent Volunteers Enrolled in Preventive Vaccine Clinical Trials', but grade 3 and 4 are pooled below, consistent with the 3 scale toxicity grading used throughout the protocol. If a laboratory value falls within the grading as specified below but also within the local laboratory normal limits, the value is considered as normal. For hemoglobin only the change from reference is used for the grading. The FDA table does not include toxicity grading for hematocrit, RBC counts or INR.

Blood, Serum, or Plasma Chemistries[a]	LO/HI/N [b]	Mild (Grade 1)	Moderate (Grade 2)	Severe (Grade 3)
Sodium (mEq/L or mmol/L)	LO	132-134	130-131	≤129
	HI	144-145	146-147	≥148
Potassium (mEq/L or mmol/L)	LO	3.5-3.6	3.3-3.4	≤3.2
	HI	5.1-5.2	5.3-5.4	≥5.5
Glucose (mg/dL)	LO	65-69	55-64	≤54
	HI [c]	100-110	111-125	>125
	HI [d]	110-125	126-200	>200
Blood urea nitrogen	HI	23-26 (mg/dL)	27-31 (mg/dL)	>31 (mg/dL)
Creatinine	HI	1.5-1.7 (mg/dL)	1.8-2.0 (mg/dL)	>2.0 (mg/dL)
Calcium (mg/dL)	LO	8.0-8.4	7.5-7.9	<7.5
	HI	10.5-11.0	11.1-11.5	>11.5
Magnesium (mg/dL)	LO	1.3-1.5	1.1-1.2	<1.1
Phosphorus (mg/dL)	LO	2.3-2.5	2.0-2.2	<2.0
CK (mg/dL)	N	1.25-1.5 x ULN	1.6-3.0 x ULN	≥3.1 x ULN
Albumin (g/dL)	LO	2.8-3.1	2.5-2.7	<2.5
Total protein (g/dL)	LO	5.5-6.0	5.0-5.4	<5.0
Alkaline phosphatase (U/L)	N	1.1-2 x ULN	2.1-3 x ULN	>3 x ULN
AST (U/L)	HI	1.1-2.5 x ULN	2.6-5 x ULN	>5 x ULN
ALT (U/L)	HI	1.1-2.5 x ULN	2.6-5 x ULN	>5 x ULN
Bilirubin, serum total (mg/dL) – when accompanied by any increase in Liver Function Test		1.1–1.25 x ULN	1.26 –1.5 x ULN	>1.5 x ULN
Bilirubin, serum total (mg/dL) – when Liver Function Test is normal		1.1–1.5 x ULN	1.6–2.0 x ULN	>2.0 x ULN
Amylase (U/L)	N	1.1-1.5 x ULN	1.6-2.0 x ULN	>2.0 x ULN
Lipase (U/L)	N	1.1-1.5 x ULN	1.6-2.0 x ULN	>2.0 x ULN

Hematology	LO/HI/N [e]	Mild (Grade 1)	Moderate (Grade 2)	Severe (Grade 3)
Hemoglobin (women) change from baseline (g/dL)	LO	Any decrease-1.5	1.6-2.0	>2.0
Hemoglobin (men) change from	LO	Any decrease-	1.6-2.0	>2.0

[a] Depending upon the laboratory used, reference ranges, eligibility ranges and grading may be split out by sex and/or age.
[b] Low, High, Not Graded.
[c] Fasting.
[d] Non-fasting.
[e] Low, High, Not Graded.

baseline (g/dL)		1.5		
White blood cell count (cell/mm^3)	HI	10,800-15,000	15,001-20,000	>20,000
	LO	2,500-3,500	1,500-2,499	<1,500
Lymphocytes (cell/mm^3)	LO	750-1,000	500-749	< 500
Neutrophils (cell/mm^3)*	LO	1,500-2,000	1,000-1,499	< 1000
Eosinophils (cell/mm^3)	HI	650-1500	1501-5000	> 5000
Platelets (cell/mm^3)	LO	125,000-140,000	100,000-124,999	<100,000
**Coagulation**				
PT (seconds)	HI	1.0-1.10 x ULN	1.11-1.20 x ULN	>1.20 x ULN
International Normalized Ratio (INR)[a]	HI	1.1-1.5 x ULN	1.6-2.0 x ULN	>2.0 x ULN
PTT or aPTT (seconds)	HI	1.0-1.2 x ULN	1.21-1.4 x ULN	>1.4 x ULN
Fibrinogen (mg/dL)	HI	400-500	501-600	>600
	LO	150-200	125-149	<125
**Urine**				
Protein (dipstick)	HI	Trace	1+	2+
Glucose (dipstick)	HI	Trace	1+	2+
Blood (microscopic) - red blood cells per high power field (RBC/HPF)	HI	1-10	11-50	>50 and/or gross blood

[*] For African subjects, the following absolute neutrophil counts should be used to determine the severity level: grade 1 = 750 – 999 cell/uL; grade 2 = 500-749 cells/uL; grade 3 <500 cells/uL

[a] For INR, the values in the table are based on the Division of AIDS Table for Grading the Severity of Adult and Pediatric Adverse Events, 2009.

RANGES TO CONVERT FDA SCALE (mg/dL) TO SI UNITS

**Blood, Serum, or Plasma Chemistries**[a]	**LO/HI/N**[b]	**Mild (Grade 1)**	**Moderate (Grade 2)**	**Severe (Grade 3)**
Glucose (mmol/L)	LO	3.61-3.38	3.05-3.60	≤3.04
	HI[c]	5.55-6.11	6.12-6.94	>6.94
	HI[d]	6.11-6.94	6.95-11.10	>11.10
Blood urea nitrogen (mmol/L)	HI	8.2-9.3	9.4-11.1	>11.1
Creatinine (µmol/L)	HI	133-150	151-177	>177
Calcium (mmol/L)	LO	2.00-2.10	1.87-1.99	<1.87
	HI	2.62-2.74	2.75-2.87	>2.87
Magnesium (mmol/L)	LO	0.53-0.62	0.45-0.52	<0.45
Phosphorus (mmol/L)	LO	0.74-0.81	0.65-0.73	<0.65
Cholesterol (mmol/L)	HI	5.20-5.43	5.44-5.82	>5.82
**Coagulation**				
Fibrinogen (µmol/L)	HI	11.76-14.70	14.71-17.65	>17.65
	LO	4.41-5.88	3.68-4.40	<3.68

VITAL SIGNS TOXICITY GRADING

Grading scale used for vital signs is according to DMID Toxicity Tables (2014)

**Vital Signs**	**LO/HI/N** [e]	**Mild (Grade 1)** [f]	**Moderate (Grade 2)**	**Severe (Grade 3)**
Fever (°C)[g]	HI	38.0-38.4	38.5-38.9	>38.9
Fever (°F)	HI	100.4-101.1	101.2-102.0	>102.0
Tachycardia	HI	101-115 bpm	116-130 bpm	>130 bpm or ventricular dysrhythmias
Bradycardia	LO	50-54 or 45-50 bpm if baseline <60 bpm	45-49 or 40-44 bpm if baseline <60 bpm	<45 or <40 bpm if baseline <60 bpm
Hypertension (systolic) - mm Hg[h]	HI	141-150	151-160	>160
Hypertension (diastolic) - mm Hg	HI	91-95	96-100	>100
Hypotension (systolic) - mm Hg	LO	85-89	80-84	<80
Tachypnea - breaths per minute	HI	23-25	26-30	>30

---

[a] Depending upon the laboratory used, reference ranges, eligibility ranges and grading may be split out by sex and/or age.
[b] Low, High, Not Graded.
[c] Fasting.
[d] Non-fasting.
[e] Low, High, Not Graded.
[f] If initial bound of grade 1 has gap from reference range or eligibility range, calculations based on the New England Journal of Medicine (NEJM) reference ranges.
[g] Oral or axillary body temperature. A protocol should select either °C or °F for inclusion.
[h] Assuming subject is awake, resting, and supine or sitting; for adverse events, 3 measurements on the same arm with concordant results.

4. HEMOGLOBIN CUT-OFF VALUES

Where no institutional normal reference ranges are available for hemoglobin, the following cut-off values are proposed. It is imperative to note that there is no standard accepted normative values for hemoglobin in most African countries and therefore, the following recommendations are based on the review of several published sources and in consultation with the sites involved with the study. In the table below, 'simplified' means that the number of cut-off categories has been reduced to decrease complexity and facilitate understanding about eligibility. Similarly, 'adjusted for safety' means that the references may quote lower average or -2 SD values for hemoglobin but these values are considered to be too low for these subjects.

Group	Value (g/dL)		Reference	Outcome
Adult and HIV+	Male 12.1	Female 9.5	LLN value for local sites in Kenya and Uganda, current values being used in Phase 1	Values kept for consistency
Adolescent 16-18 yrs	Male 12.1	Female 9.5	Robins reference 10.4 g/dL for girls and 12.4 g/dL for boys	Values simplified to correspond to adult cut-offs
Adolescent 11-15 yrs	Male 11.0	Female 9.5	Robins reference 11.0 g/dL for girls and boys	Value adjusted down for boys, no change for girls
Children 6-10 yrs	11.0		Robins reference 10.7 g/dL for girls and boys	Value simplified
Children 2-5 yrs	11.0		Robins reference 10.4 g/dL for girls and boys, LLN value for local lab in Kenya 11.5 g/dL for girls and 14.5 g/dL for boys, Schellenberg reference 8.2-9.3 g/dL average anemic defined as <11.0 g/dL	Value simplified and adjusted for safety to 11.0 g/dL
Children 1-2 yrs	11.0		Schellenberg reference 8.0 g/dL average anemic defined as <11.0 g/dL, DMID toxicity table 11.0 g/dL	Value simplified and adjusted for safety to 11.0 g/dL

Robins E and Blum S. Hematologic Reference Values for African American Children and Adolescents. American J Hematology. 2007:82, 611-614.

Schellenberg D, et al. The silent burden of anaemia in Tanzanian children: a community-based study. Bulletin of the World Health Organization 2003;81:581-590.

DMID US FDA Guidance document DIVISION OF MICROBIOLOGY AND INFECTIOUS DISEASES (DMID) PEDIATRIC TOXICITY TABLES NOVEMBER 2007

5. ADDITIONAL RULES FOR ICS ANALYSIS

Cellular immune responses, as measured by the following assay, may be analyzed:
Intracellular cytokine staining (ICS): The following responses are measured:

- CD4+: IL-2+ and IFN-γ+ responses to EBOV GP.
- CD8+: IL-2+ and IFN-γ+ responses to EBOV GP.

Per the data transfer agreement (DTA), the key variables shown in Table 5 will be available in the clinical database.

Table 5: List of variables that uniquely define a record in the clinical data

Variable	Comment
SUBJID	Subject Identifier
DTC	Date and time of collection of the sample. Format: YYYY-MM-DDTHH:MM
Visit	Visit Label from Shipping List
**Cytokine**	IFN-γ+, IFN-γ+ IL-2+, IL-2+, IFN-γ+_or_IL-2+, Total
Antigen	EBOV GP pep pool 1, EBOV GP pep pool 2, Negative Control
tcellsub	CD4+, CD8+

Important: For the responder definition, each cytokine (IFN-γ+ or IL-2+) readout should be used separately with the corresponding LOD. Also, both "sample interpretation" and "responder" should be defined separately for T cell type (ie CD4+ and CD8+).

Computation of sample interpretation (positivity)

For each type of cytokine expressing T cells (CD4+ or CD8+), each peptide pool (GP1 or GP2) and negative control (mock [unstimulated] readout), the database will contain:

- Total number of cells (***nsub***)
- Marginal cytokine expressing T cells subsets (***cytnum***)
- Total cell count of cytokine expressing T cells (any of IL-2+ or IFN-γ+ present)

The sample positivity (interpretation) will be determined for each of the 2 peptide pools separately as outlined in Table 6, where ***cytnum_neg*** and ***nsub_neg*** respectively denote the positive T cell counts and total T cell counts of the negative control (unstimulated readout).

Table 6: Derivation steps for ICS sample interpretation

Step	Computation
1	Per T cell type (CD4+ or CD8+), compute the percentage positive T cell counts for each antigen peptide pool (GP1, GP2) separately for each cytokine (IFN-γ+, IL-2+) based on Equations (1) and (2). The T cell counts identified in the database as IFN-γ+ (eg this is captured on the record where ISCAT= CD4+ IFN-γ+ for CD4+ T cell type) and IL-2+ (eg this is captured on the record where ISCAT= CD4+IL-2+ for CD4+ T cell type) should be used. Let $\%\boldsymbol{EBOV\ GPx\ results\_pos}$ and $\%\boldsymbol{Negative\ control\_pos}$ denote EBOV peptide pool stimulated readout (percentage positive T cell) and the mock (unstimulated) readout (percentage positive T cell), respectively. Then for each GP$x(x = 1, 2)$, $$\%EBOV\ GPx\ results\_pos = \frac{cytnum_x}{nsub_x} \times 100 \quad (1)$$ $$\%Negative\ control\_pos = \frac{cytnum\_neg}{nsub\_neg} \times 100 \quad (2)$$ If replicate negative control samples are available for a subject at one timepoint, the average of the negative controls should be used.
2	Per T cell type (CD4+ or CD8+), compute the mock subtracted percentages for each antigen peptide pool (GP1, GP2) separately for each cytokine (IFN-γ+, IL-2+) using Equation (3). $$\%Background\ subtracted\ EBOV\ GPx = Max(\%EBOV\ GPx\ results\_pos - \%Negative\ control\_pos, 0) \quad (3)$$
3	Compute the fold over mock values as ratio of the EBOV stimulated readout to the negative control readout as shown in Equation (4). To avoid computational problems (e.g. division by zero) all values of the $\%Negative\ control\_pos$ that are less than 0.001% should be imputed with 0.001% in the Equation. $$Fold\ over\ mock\ EBOV\ GPx = \frac{\%EBOV\ GPx\ results\_pos}{\%Negative\ control\_pos}, \quad (4)$$
4	**Sample interpretation per T cell type and antigen peptide pool combination:** For each T cell type (CD4+, CD8+) and peptide pool (GP1, GP2) determine sample positivity per cytokine (IFN-γ+, IL-2+). That is, a sample is considered positive for a given T cell type and peptide pool when either cytokine (IFN-γ or IL-2) satisfies the following:<br>- EBOV stimulated readout is greater than 3-fold the mock readout (i.e. $Fold\ over\ mock\ EBOV\ GPx > 3$); AND<br>- Mock-subtracted percentage ($\%background\ subtracted\ EBOV\ GPx$) is greater than the LOD.
5	**Sample interpretation per T cell type:** For a given T cell type, the sample is considered positive if positivity is declared for either peptide pool GP1 or GP2 in Step 4.

Pure cytokine combinations

In addition to the sample interpretation, the background adjusted percentage of any subset (GP1 and GP2 combined) for the total cytokine response (captured on the records IFN-γ+ or IL-2+) and any of the "*pure*" cytokine combinations (Table 7 below) should also be computed. The steps below should be followed.

Table 7: Pure cytokine combinations computation formulae

**Formula for "*pure*" cytokine counts**		
**IFN-γ**	**IL-2**	
+	+	(IFN-γ+IL-2+)
-	+	(IL-2+) - (IFN-γ+IL-2+)
+	-	(IFN-γ+) - (IFN-γ+IL-2+)

1. Apply the formula from the Table 7 above with the marginal cytokine counts (*cytnum* [positive T cell counts]) for each "*pure*" cytokine combination (*c_cytnum*)

   For each cytokine combination apply the formula also for the negative control(s) to obtain *c_cytnum_neg* ("pure" cytokine combination of unstimulated readout).

2. The total background adjusted EBOV GP values (captured on the records IFN-γ+ or IL-2+) will be presented in tables, listings, dot plots and regimen profiles. The graphical displays should be on the log10-scale. For ICS, values below or equal to zero, will be imputed to 0.0001. Fold increase will not be calculated for the total background adjusted EBOV GP values in the analysis. If a value is negative or zero, "0.0001" will be shown in tables and listings instead of "<LOD", as the LOD are meant for individual cytokines. The imputation value of 0.0001 is chosen, because an imputation to zero would cause issues with the log-scale. The imputation value of 0.0001 for the percentage corresponds to 1 positive cell out of the 1 million cells that went into the ICS assay. This is therefore the lowest possible value observable above zero in the ICS assay.

3. The total background adjusted percentage of any subset (GP1 and GP2 combined) for the total cytokine response (captured on the records IFN-γ+ or IL-2+) and any of the "*pure*" cytokine combinations is the sum of non-negative background adjusted percentages from peptide pools GP1 and GP2. It is expressed as

$$\mathrm{Max}\left(\mathrm{c\_pctpos}_{\mathrm{adj}_1}, 0\right) + \mathrm{Max}\left(\mathrm{c\_pctpos}_{\mathrm{adj}_2}, 0\right)$$

Important:

1. The total background adjusted "*pure*" percentages will be used for the pie charts and bar charts without any further imputation.

2. The total background adjusted EBOV GP values (captured on the records IFN-γ+ or IL-2+) will be presented in tables, dot plots and regimen profiles. The LOD of IFN-γ+ will be used as the general LOD for the total background adjusted EBOV GP values captured on the records IFN-γ+ or IL-2+.
