# Supplementary material for: Safety and Immunogenicity of an Accelerated Ebola Vaccination Schedule in People with and without Human Immunodeficiency Virus: A Randomized Clinical Trial
Source: Vaccines (Basel). 2024 May 4;12(5):497. doi: 10.3390/vaccines12050497 (PMC11125575; doi:10.3390/vaccines12050497)
Supplement: Supplementary file 1 [file vaccines-12-00497-s001.zip › Supplement/Supplement S3. Supplementary material.doc]

**Supplementary Material**

Supplement S1. Study protocol
Supplement S2. Statistical analysis plan
Supplement S3. Supplementary material

Supplementary methods

Supplementary results

Supplementary Table S1. Solicited local AEs by worst severity grade (full analysis set)

Supplementary Table S2. Solicited systemic AEs by worst severity grade (full analysis set)

Supplementary Table S3. Unsolicited AEs (full analysis set)

Supplementary Table S4. SAEs (full analysis set)

Supplementary Table S5. HIV viral load: categorization of HIV viral loads in PLWH

Supplementary Table S6. EBOV GP-specific binding antibody responses in each study group, from baseline to study completion as measured by FANG anti-EBOV GP IgG ELISA

Supplementary Figure S1. Correlation between HIV viral load at baseline (panel A) and CD4+ T-cells at baseline (panel B) and EBOV GP binding antibody in active vaccine recipients at Day 36

Supplementary Figure S2. Correlation between EBOV GP binding antibody (Kikwit) and neutralizing antibody responses at Day 36 in PWOH and PLWH (active vaccine recipients)

Supplementary Figure S3. Cross-strain EBOV GP-specific neutralizing antibody responses as measured by psVNA

Supplementary Figure S4. Cross-strain EBOV GP neutralizing antibody responses as measured by psVNA

Supplementary Figure S5. Correlation between cross-strain neutralizing antibody responses and EBOV GP-specific binding antibody responses at Day 36 in active vaccine recipients

Supplementary Figure S6. EBOV GP-specific ADCP responses

Supplementary Figure S7. EBOV GP-specific antibody-dependent natural killer cell activation (ADNKA) responses

Supplementary Figure S8. EBOV GP-specific ADCD responses

Supplementary Figure S9. Antibody effector polyfunctionality

Supplementary Figure S10. Combinatorial polyfunctionality analysis of antigen-specific T-cell subsets (COMPASS)

Members of the VAC52150/EBL2003/RV456 study team (in addition to authors)

**Supplement S3.**

**Supplementary methods**

**Viral Loads and CD4+ Counts in People Living With HIV (PLWH)**

HIV viral loads (Roche COBAS AmpliPrep/COBAS TaqMan HIV-1 v2.0) and CD4+ cell counts were measured at screening, baseline, Day 15 prior to MVA-BN-Filo vaccination, Days 21 and 42 post-dose 2, and at 6 and 12 months using standard methodology. Post-hoc correlation analyses were performed in PLWH to evaluate the potential impact of HIV viral load and CD4+ T-cell count prior to dose 1 and dose 2 vaccinations on the Ebola virus (EBOV) glycoprotein (GP)-specific binding antibody concentrations at Day 36 at an individual level.

**Methodology of Filovirus Animal Non-Clinical Group (FANG) Anti-EBOV GP Immunoglobulin G (IgG) Enzyme-Linked Immunosorbent Assay (ELISA)**

Participants were considered EBOV GP binding antibody responders if a) the postvaccination concentration was more than 2.5-fold higher than the lower limit of quantification (LLOQ; 36.11 ELISA units [EU]/mL) for participants negative at baseline, or b) more than 2.5-fold higher than the baseline value in those participants who were positive prevaccination.

**Methodology of Pseudovirion Neutralization Assay (psVNA) at U.S. Army Research Institute of Infectious Diseases (USAMRIID)**

All specimens were evaluated for the presence of neutralizing antibodies using a psVNA. The USAMRIID psVNA is an assay that has been used previously in clinical and nonclinical research [1–3]. The psVNA utilizes engineered vesicular stomatitis virus (VSV) that expresses a luciferase reporter gene in the place of the virus G envelope glycoprotein genes. Filovirus pseudovirions (PsV) were generated for Kikwit-95, Makona-14, and Bundibugyo and were then purified and quantified as described previously [2]. To perform the neutralization assay, each individual filovirus PsV (4000 focus forming units) was combined with serum (1:20–1:1,562,500 dilution range) in the presence of a human complement (5%; Sigma, St. Louis, Missouri, USA) and incubated overnight at 2–8°C. The PsV plus serum mixture was then added to Vero-76 cell monolayers in clear bottom black-walled 96-well microtiter plates. The plates were incubated for 18–24 hours and then media removed, lysis luciferase reagent (Promega, Madison, Wisconsin, USA) added, and flash luminescence data acquired using a luminometer (Tecan M200 Pro microplate reader, Mannedorf, CH, Switzerland). If sera contain antibodies that prevent the PsV from attaching to and/or entering cells, then the reporter activity is neutralized. Neutralization titers are interpolated from 4-parameter curves using GraphPad Prism (GraphPad, San Diego, California, USA). The reciprocal of the interpolated dilution that results in a 50% decrease in luciferase activity is the psVNA_50_. Participants were considered as responders for psVNA if a sample was negative at baseline (50% inhibitory concentration [IC_50_] titer <20) and positive postbaseline, and the postbaseline titer was greater than two times the assay limit (IC_50_ titer of 20), or samples were positive both at baseline and postbaseline but with a greater than four-fold increase from baseline.

**Methodology for Fc-mediated Antibody Effector Functions**

For all Fc-mediated antibody effector functions, a responder was defined as having a response above the positivity threshold (the median plus two times the standard deviation of plasma samples from five normal healthy donors).

**Methodology for Antibody-dependent Activation of Natural Killer Cells (ADNKA)**

Ninety-six well plates were coated with Ebola GP antigen at 150 μg/mL in phosphate-buffered saline (PBS) over night at 4°C. Plates were then extensively washed with PBS and diluted plasma samples were incubated at room temperature (RT) for 2 hours. After washing, cryopreserved peripheral blood mononuclear cells (PBMCs) were added to each well and incubated for 6 hours at 37°C in presence of monensin (BD Bioscience) and brefeldin (eBiosceince). After stimulation, cells were washed and surface stained at RT for 10 minutes in the dark. Samples were then washed and fixed using Fix & Perm Medium A (Life Technologies). Intracellular cytokine staining (ICS) was performed in Fix & Perm Medium B (Life Technologies). Commercial monoclonal antibodies used in flow cytometry included: anti-CD3 AF700 (clone UCHT1), anti-CD56 PE-Cy7 (clone B159), anti-CD16 APC-Cy7 (clone 3G8), anti-MIP-1β PE (clone D21-1351) and anti-IFN-γ V450 (clone B27; all from BD Biosciences) and anti-CD14 V510 (clone M5E2), anti-CD19 V510 (clone HIB19), anti-TNF FITC (clone MAb11; all from BioLegend). Data were acquired on a BD LSRII instrument and analyzed using FlowJo Version 9.8.5 software.

**Methodology for Antibody-dependent Cellular Phagocytosis (ADCP)**

Briefly, Ebola GP was biotinylated at a biotin to protein ratio of 1:50 following manufacturer’s instructions (Thermo Scientific) and incubated with yellow-green strepavidin-fluorescent beads (Molecular Probes) for 2 hours at 37°C. Ten microliters of a 100-fold dilution of beads-protein was incubated 2 hours at 37°C with 100 μl diluted plasma samples before addition of THP-1 cells (MilliporeSigma, Burlington, Massachusetts, USA). After a 19-hour incubation at 37°C, the cells were fixed with 4% formaldehyde solution (Tousimis, Rockville, Maryland, USA) and fluorescence was evaluated on a LSRII (BD Bioscience). The phagocytic score was calculated by multiplying the percentage of bead-positive cells by the geometric mean fluorescence intensity of the bead-positive cells and dividing by 10^4^.

**Methodology for Antibody-mediated Complement Deposition (ADCD)**

Biotinylated GP-coated red Neutravidin beads (Thermo Fisher) were incubated with heat-inactivated samples as described for ADCP above. Guinea pig complement (Cedarlane Labs) diluted in veronal buffer containing calcium and magnesium (Boston Bioproducts) was incubated with antibody-bead complexes for 20 minutes, and C3 deposition onto beads was detected using an anti–guinea pig C3 antibody (MP Biomedicals) and measured by flow cytometry.

**ICS Methodology**

Briefly, cryopreserved PBMCs were thawed, washed, counted, and resuspended in 10% fetal bovine serum–Roswell Park Memorial Institute (RPMI) at 2×10^6^ cells/mL in a 50 mL conical tube and placed in a 37ºC/5% CO_2_ incubator overnight. Post–overnight incubation, cells were washed and recounted to determine cell yield and viability <66%. Any samples that did not meet cell yield and viability criteria were excluded from analysis. 100 μL of resuspended cells at 10×10^6^ cells/mL and 25 μL of each stimulant, co-stimulant, and transport blockers (dimethyl sulfoxide [DMSO], EBOV1 and EBOV2 peptide pools, SEB, CD28/49d, Brefeldin A, and monensin) were plated in a 96-well plate and incubated for 6 hours at 37ºC/5% CO_2_. Post–6-hour incubation, 20 mM ethylenediaminetetraacetic acid was added to each well, and plates were wrapped in foil and stored overnight at 4°C. The following day, plates were centrifuged at 750 ×g for 3 minutes and washed three times with PBS. Cells were then resuspended in 50 μL of Live/Dead Aqua stain or PBS and incubated in the dark, at RT for 20 minutes. 150 μL PBS was added to each well, and plate was centrifuged 750 ×g for 3 minutes. Cells were then resuspended in 50 μL of surface staining cocktail or fluorescence-activated cell sorting (FACS) Wash Buffer and incubated in the dark, at RT for 20 minutes. 150 μL FACS Wash Buffer was added to each well and washed three times. Cells were resuspended in each well with 100 μL of 1X BD FACS Lyse Working Solution and incubated in the dark at RT for 10 minutes. 100 μL of FACS Wash Buffer was added to each well. Plates were then centrifuged at 750 ×g/3 minutes, and supernatant flicked from the wells. Cells were resuspended in 200 μL of FACS Perm II Working Solution (BD Biosciences), and the plates were incubated for 10 minutes in the dark at RT. Plates were then centrifuged at 750 ×g for 3 minutes and supernatant flicked from the wells. Cells were resuspended in 200 μL of FACS Wash Buffer (BD Biosciences) and washed three times. Cells were resuspended in 50 μL ICS antibody cocktail and incubated in the dark, at RT for 30 minutes. 150 μL FACS Wash Buffer was added to each well and washed three times. Cells were incubated in 1% paraformaldehyde in the dark, at RT for 15 minutes. Cells were washed three times, resuspended in 200 μL FACS Wash Buffer and run in plate mode on LSRII (BD Biosciences). Commercial antibodies included: CD4 BUV395 (clone SK3), IFN-γ V450 (clone B27), AViD AQUA viability dye, CD14 BV510 (clone M5E2), CD3 BV570 (clone UCHT1), CD154 BV605 (clone 24-31), CD56 BV650 (clone HCD56), CD8 BV711 (cloneRPA-T8), CCR7 BV785 (clone G043H7), TNF-α FITC (clone MAb11), IL-4 PerCP-Cy5.5 (cloneMP4-25D2), IL-2 PE (clone MQ1-17H12), CXCR5 PE-eFluor610 (clone MU5UBEE), PD-1 PE-Cy7 (clone eBioJ105), IL-21 APC (clone 3A3-N2), and CD45RA APC-H7 (clone HI100). Data was analyzed using FlowJo Software. In addition to the qualified markers IFN-γ and IL-2, the exploratory markers IL-4, IL-21, TNF-α, and CD154 were also measured by ICS at the same time points and used for combinatorial polyfunctionality analysis of antigen-specific T-cell subsets using the computational software COMPASS. This software models all observed T-cell subsets and selects those most likely to have antigen-specific responses [4].

For cell-mediated immune responses assessed by ICS, the proportion of T-cells producing IFN-γ, IL-2, or both, at each time point, were quantified separately and used to calculate the sum of the T-cell response to the two EBOV GP peptide pools. For a given time point, if a responder status was achieved based on either IFN-γ or IL-2, the participant was declared as a responder at that time point. A participant was a responder if the sample interpretation was negative at baseline and positive postbaseline and the postbaseline value was greater than two times the limit of detection [0.0221% CD4+ IFN-γ, 0.035% CD4+ IL-2, 0.0705% CD8+ IFN-γ, and 0.041% CD8+ IL-2], or the sample interpretation was positive both at baseline and postbaseline and there was a greater than two-fold increase from baseline in the background-adjusted percentage of the combined peptide pools.

**Supplementary References**

1.	Grant-Klein RJ, Altamura LA, Badger CV, et al. Codon-optimized filovirus DNA vaccines delivered by intramuscular electroporation protect cynomolgus macaques from lethal Ebola and Marburg virus challenges. Hum Vaccin Immunother, **2015** ; 11: 1991–2004.

2.	Kwilas S, Kishimori JM, Josleyn M, et al. A hantavirus pulmonary syndrome (HPS) DNA vaccine delivered using a spring-powered jet injector elicits a potent neutralizing antibody response in rabbits and nonhuman primates. Curr Gene Ther, **2014**; 14: 200–210.

3.	Regules JA, Beigel JH, Paolino KM, et al. A recombinant vesicular stomatitis virus Ebola vaccine. N Engl J Med, **2017**; 376: 330–341.

4.	Lin L, Finak G, Ushey K, et al. COMPASS identifies T-cell subsets correlated with clinical outcomes. Nat Biotechnol, **2015**; 33: 610–616.

**Supplementary results**

**COMPASS Posterior Probabilities for EBOV-specific CD4+ and CD8+ T-cells**

In both people without HIV (PWOH) and people living with HIV (PLWH), the posterior probability heatmaps indicate the robust presence of an EBOV GP-specific CD4+ polyfunctional T-cell subset co-expressing IL-2, TNF-α, IFN-γ, and CD154. (Supplementary Figure 9A, **Supplement 3**). For EBOV GP-specific CD8+ T-cells, the subset co-expressing CD154, TNF-α, and IFN-γ comprised the greatest contribution to polyfunctionality (Supplementary Figure 9B, **Supplement 3**).

**Impact of HIV Parameters on EBOV GP Binding Antibody Responses**

A low negative correlation (Spearman correlation factor: –0.344) was observed between the baseline HIV viral load and vaccine-induced binding antibody concentrations at Day 36 (Supplementary Figure 1A, **Supplement 3**). A negligible positive correlation (Spearman correlation coefficient: 0.298) was observed between the baseline CD4+ T-cell count and the EBOV GP-specific binding antibody concentrations at Day 36 at an individual level (Supplementary Figure 1B, **Supplement 3**).

**Supplementary Table S1. Solicited local AEs by worst severity grade (full analysis set)**

|  |  | **PWOH** | | **PLWH** | |
| --- | --- | --- | --- | --- | --- |
| **Dictionary-derived Term** | **Grade** | **MVA, Ad26** | **Placebo, Placebo** | **MVA, Ad26** | **Placebo, Placebo** |
| **Post-dose 1, N** |  | **40** | **10** | **20** | **5** |
| Any solicited local AE, n (%) | Any | 24 (60) | 3 (30) | 10 (50) | 0 |
|  | Grade 1 | 19 (48) | 3 (30) | 9 (45) | 0 |
|  | Grade 2 | 5 (13) | 0 | 1 (5) | 0 |
| Injection-site erythema | Any | 2 (5) | 0 | 1 (5) | 0 |
|  | Grade 1 | 1 (3) | 0 | 0 | 0 |
|  | Grade 2 | 1 (3) | 0 | 1 (5) | 0 |
| Injection-site pain | Any | 22 (55) | 3 (30) | 9 (45) | 0 |
|  | Grade 1 | 18 (45) | 3 (30) | 9 (45) | 0 |
|  | Grade 2 | 4 (10) | 0 | 0 | 0 |
| Injection-site pruritus | Any | 3 (8) | 0 | 3 (15) | 0 |
|  | Grade 1 | 3 (8) | 0 | 3 (15) | 0 |
| Injection-site swelling | Any | 4 (10) | 0 | 4 (20) | 0 |
|  | Grade 1 | 2 (5) | 0 | 3 (15) | 0 |
|  | Grade 2 | 2 (5) | 0 | 1 (5) | 0 |
| **Post-dose 2, N** |  | **39** | **10** | **20** | **5** |
| Any solicited local AE, n (%) | Any | 27 (69) | 1 (10) | 11 (55) | 0 |
|  | Grade 1 | 15 (39) | 1 (10) | 7 (35) | 0 |
|  | Grade 2 | 6 (15) | 0 | 2 (10) | 0 |
|  | Grade 3 | 6 (15) | 0 | 2 (10) | 0 |
| Injection-site erythema | Any | 9 (23) | 0 | 2 (10) | 0 |
|  | Grade 1 | 3 (8) | 0 | 0 | 0 |
|  | Grade 2 | 1 (3) | 0 | 0 | 0 |
|  | Grade 3 | 5 (13) | 0 | 2 (10) | 0 |
| Injection-site pain | Any | 23 (59) | 1 (10) | 11 (55) | 0 |
|  | Grade 1 | 17 (44) | 1 (10) | 8 (40) | 0 |
|  | Grade 2 | 5 (13) | 0 | 3 (15) | 0 |
|  | Grade 3 | 1 (3) | 0 | 0 | 0 |
| Injection-site pruritus | Any | 4 (10) | 0 | 4 (20) | 0 |
|  | Grade 1 | 4 (10) | 0 | 4 (20) | 0 |
| Injection-site swelling | Any | 9 (23) | 0 | 6 (30) | 0 |
|  | Grade 1 | 6 (15) | 0 | 5 (25) | 0 |
|  | Grade 2 | 2 (5) | 0 | 1 (5) | 0 |
|  | Grade 3 | 1 (3) | 0 | 0 | 0 |
| **Regimen, N** |  | **40** | **10** | **20** | **5** |
| Any solicited local AE, n (%) | Any | 32 (80) | 4 (40) | 15 (75) | 0 |
|  | Grade 1 | 16 (40) | 4 (40) | 10 (50) | 0 |
|  | Grade 2 | 10 (25) | 0 | 3 (15) | 0 |
|  | Grade 3 | 6 (15) | 0 | 2 (10) | 0 |
| Injection-site erythema | Any | 10 (25) | 0 | 3 (15) | 0 |
|  | Grade 1 | 3 (8) | 0 | 0 | 0 |
|  | Grade 2 | 2 (5) | 0 | 1 (5) | 0 |
|  | Grade 3 | 5 (13) | 0 | 2 (10) | 0 |
| Injection-site pain | Any | 30 (75) | 4 (40) | 14 (70) | 0 |
|  | Grade 1 | 20 (50) | 4 (40) | 11 (55) | 0 |
|  | Grade 2 | 9 (23) | 0 | 3 (15) | 0 |
|  | Grade 3 | 1 (3) | 0 | 0 | 0 |
| Injection-site pruritus | Any | 7 (18) | 0 | 6 (30) | 0 |
|  | Grade 1 | 7 (18) | 0 | 6 (30) | 0 |
| Injection-site swelling | Any | 11 (28) | 0 | 9 (45) | 0 |
|  | Grade 1 | 6 (15) | 0 | 7 (35) | 0 |
|  | Grade 2 | 4 (10) | 0 | 2 (10) | 0 |
|  | Grade 3 | 1 (3) | 0 | 0 | 0 |

The denominator is the number of participants with available reactogenicity data after the given dose.

n (%): number (percentage) of participants with one or more event.

Ad26, Ad26.ZEBOV; AE, adverse event; MVA, MVA-BN-Filo; N, number of participants; PLWH, people living with HIV; PWOH, people without HIV.

**Supplementary Table S2. Solicited systemic AEs by worst severity grade (full analysis set)**

|  |  | **PWOH** | | **PLWH** | |
| --- | --- | --- | --- | --- | --- |
| **Dictionary-derived Term** | **Grade** | **MVA, Ad26** | **Placebo, Placebo** | **MVA, Ad26** | **Placebo, Placebo** |
| **Post-dose 1, N** |  | 40 | 10 | 20 | 5 |
| Any solicited systemic AE, n (%) | Any | 12 (30) | 4 (40) | 8 (40) | 3 (60) |
|  | Grade 1 | 7 (18) | 3 (30) | 7 (35) | 2 (40) |
|  | Grade 2 | 5 (13) | 1 (10) | 1 (5) | 1 (20) |
| Arthralgia | Any | 4 (10) | 2 (20) | 1 (5) | 0 |
|  | Grade 1 | 3 (8) | 1 (10) | 1 (5) | 0 |
|  | Grade 2 | 1 (3) | 1 (10) | 0 | 0 |
| Chills | Any | 5 (13) | 1 (10) | 2 (10) | 1 (20) |
|  | Grade 1 | 5 (13) | 1 (10) | 2 (10) | 0 |
|  | Grade 2 | 0 | 0 | 0 | 1 (20) |
| Fatigue | Any | 10 (25) | 2 (20) | 6 (30) | 3 (60) |
|  | Grade 1 | 6 (15) | 2 (20) | 6 (30) | 2 (40) |
|  | Grade 2 | 4 (10) | 0 | 0 | 1 (20) |
| Headache | Any | 7 (18) | 1 (10) | 6 (30) | 2 (40) |
|  | Grade 1 | 3 (8) | 1 (10) | 5 (25) | 1 (20) |
|  | Grade 2 | 4 (10) | 0 | 1 (5) | 1 (20) |
| Myalgia | Any | 9 (23) | 3 (30) | 5 (25) | 0 |
|  | Grade 1 | 6 (15) | 3 (30) | 5 (25) | 0 |
|  | Grade 2 | 3 (8) | 0 | 0 | 0 |
| Nausea | Any | 2 (5) | 0 | 1 (5) | 0 |
|  | Grade 1 | 2 (5) | 0 | 1 (5) | 0 |
| Pyrexia | Any | 0 | 0 | 0 | 0 |
| **Post-dose 2, N** |  | 39 | 10 | 20 | 5 |
| Any solicited systemic AE, n (%) | Any | 24 (62) | 2 (20) | 10 (50) | 2 (40) |
|  | Grade 1 | 12 (31) | 2 (20) | 5 (25) | 2 (40) |
|  | Grade 2 | 9 (23) | 0 | 5 (25) | 0 |
|  | Grade 3 | 3 (8) | 0 | 0 | 0 |
| Arthralgia | Any | 8 (21) | 0 | 1 (5) | 0 |
|  | Grade 1 | 4 (10) | 0 | 1 (5) | 0 |
|  | Grade 2 | 4 (10) | 0 | 0 | 0 |
| Chills | Any | 11 (28) | 0 | 5 (25) | 0 |
|  | Grade 1 | 6 (15) | 0 | 2 (10) | 0 |
|  | Grade 2 | 3 (8) | 0 | 3 (15) | 0 |
|  | Grade 3 | 2 (5) | 0 | 0 | 0 |
| Fatigue | Any | 20 (51) | 2 (20) | 8 (40) | 2 (40) |
|  | Grade 1 | 10 (26) | 2 (20) | 6 (30) | 2 (40) |
|  | Grade 2 | 7 (18) | 0 | 2 (10) | 0 |
|  | Grade 3 | 3 (8) | 0 | 0 | 0 |
| Headache | Any | 16 (41) | 0 | 4 (20) | 2 (40) |
|  | Grade 1 | 10 (26) | 0 | 2 (10) | 2 (40) |
|  | Grade 2 | 4 (10) | 0 | 2 (10) | 0 |
|  | Grade 3 | 2 (5) | 0 | 0 | 0 |
| Myalgia | Any | 11 (28) | 0 | 3 (15) | 0 |
|  | Grade 1 | 6 (15) | 0 | 2 (10) | 0 |
|  | Grade 2 | 5 (13) | 0 | 1 (5) | 0 |
| Nausea | Any | 6 (15) | 0 | 4 (20) | 0 |
|  | Grade 1 | 3 (8) | 0 | 2 (10) | 0 |
|  | Grade 2 | 3 (8) | 0 | 2 (10) | 0 |
| Pyrexia | Any | 4 (10) | 0 | 2 (10) | 0 |
|  | Grade 1 | 4 (10) | 0 | 1 (5) | 0 |
|  | Grade 2 | 0 | 0 | 1 (5) | 0 |
| **Regimen, N** |  | 40 | 10 | 20 | 5 |
| Any solicited systemic AE, n (%) | Any | 28 (70) | 5 (50.0%) | 12 (60.0%) | 3 (60.0%) |
|  | Grade 1 | 14 (35) | 4 (40) | 7 (35) | 2 (40) |
|  | Grade 2 | 11 (28) | 1 (10) | 5 (25) | 1 (20) |
|  | Grade 3 | 3 (8) | 0 | 0 | 0 |
| Arthralgia | Any | 10 (25) | 2 (20) | 2 (10) | 0 |
|  | Grade 1 | 5 (13) | 1 (10) | 2 (10) | 0 |
|  | Grade 2 | 5 (13) | 1 (10) | 0 | 0 |
| Chills | Any | 16 (40) | 1 (10) | 6 (30) | 1 (20) |
|  | Grade 1 | 11 (28) | 1 (10) | 3 (15) | 0 |
|  | Grade 2 | 3 (8) | 0 | 3 (15) | 1 (20) |
|  | Grade 3 | 2 (5) | 0 | 0 | 0 |
| Fatigue | Any | 23 (58) | 3 (30) | 12 (60) | 3 (60) |
|  | Grade 1 | 11 (28) | 3 (30) | 10 (50) | 2 (40) |
|  | Grade 2 | 9 (23) | 0 | 2 (10) | 1 (20) |
|  | Grade 3 | 3 (8) | 0 | 0 | 0 |
| Headache | Any | 20 (50) | 1 (10) | 8 (40) | 2 (40) |
|  | Grade 1 | 11 (28) | 1 (10) | 5 (25) | 1 (20) |
|  | Grade 2 | 7 (18) | 0 | 3 (15) | 1 (20) |
|  | Grade 3 | 2 (5) | 0 | 0 | 0 |
| Myalgia | Any | 15 (38) | 3 (30) | 7 (35) | 0 |
|  | Grade 1 | 8 (20) | 3 (30) | 6 (30) | 0 |
|  | Grade 2 | 7 (18) | 0 | 1 (5) | 0 |
| Nausea | Any | 8 (20) | 0 | 4 (20) | 0 |
|  | Grade 1 | 5 (13) | 0 | 2 (10) | 0 |
|  | Grade 2 | 3 (8) | 0 | 2 (10) | 0 |
| Pyrexia | Any | 4 (10) | 0 | 2 (10) | 0 |
|  | Grade 1 | 4 (10) | 0 | 1 (5) | 0 |
|  | Grade 2 | 0 | 0 | 1 (5) | 0 |

The denominator is the number of participants with available reactogenicity data after the given dose.

n (%): number (percentage) of participants with one or more event.

Ad26, Ad26.ZEBOV; AE, adverse event; MVA, MVA-BN-Filo; N, number of participants; PLWH, people living with HIV; PWOH, people without HIV.

**Supplementary Table S3.** **Unsolicited AEs (full analysis set)**

|  |  | **PWOH** | | **PLWH** | |
| --- | --- | --- | --- | --- | --- |
| **Participants with at least one unsolicited AE** | **Grade** | **MVA, Ad26** | **Placebo, Placebo** | **MVA, Ad26** | **Placebo, Placebo** |
| **Post-dose 1, N** |  | 40 | 10 | 20 | 5 |
| Unsolicited AE, n (%) | Any | 11 (28) | 2 (20) | 8 (40) | 0 |
|  | Grade 1 | 6 (15) | 1 (10) | 5 (25) | 0 |
|  | Grade 2 | 4 (10) | 1 (10) | 3 (15) | 0 |
|  | Grade 3 | 1 (3) | 0 | 0 | 0 |
| Unsolicited AE that is related to vaccine | Any | 3 (8) | 1 (10) | 1 (5) | 0 |
| AE leading to permanent stop of vaccine | – | 1 (3) | 0 | 0 | 0 |
| SAE | – | 0 | 0 | 0 | 0 |
| **Post-dose 2, N** |  | 39 | 10 | 20 | 5 |
| Unsolicited AE, n (%) | Any | 9 (23) | 4 (40) | 6 (30) | 0 |
|  | Grade 1 | 6 (15) | 3 (30) | 3 (15) | 0 |
|  | Grade 2 | 3 (8) | 1 (10) | 3 (15) | 0 |
|  | Grade 3 | 0 | 0 | 0 | 0 |
| Unsolicited AE that is related to vaccine | Any | 4 (10) | 0 | 3 (15) | 0 |
| AE leading to permanent stop of vaccine | – | 0 | 0 | 0 | 0 |
| SAE | – | 0 | 0 | 0 | 0 |
| **Regimen, N** |  | 40 | 10 | 20 | 5 |
| Unsolicited AE, n (%) | Any | 16 (40) | 5 (50) | 11 (55) | 0 |
|  | Grade 1 | 10 (25) | 3 (30) | 8 (40) | 0 |
|  | Grade 2 | 5 (13) | 2 (20) | 3 (15) | 0 |
|  | Grade 3 | 1 (3) | 0 | 0 | 0 |
| Unsolicited AE that is related to vaccine | Any | 7 (18) | 1 (10) | 4 (20) | 0 |
| AE leading to permanent stop of vaccine | – | 1 (3) | 0 | 0 | 0 |
| SAE | – | 0 | 0 | 0 | 0 |

Note that vaccine relates to MVA-BN-Filo, Ad26.ZEBOV, or placebo.

This table only includes AEs that were reported between the dose 1 vaccination and 14 days post-dose 1, and between the dose 2 vaccination and 28 days post-dose 2.

The denominator is the number of participants with available reactogenicity data after the given dose.

n (%): number (percentage) of participants with one or more event.

Ad26, Ad26.ZEBOV; AE, adverse event; MVA, MVA-BN-Filo; N, number of participants; PLWH, people living with HIV; PWOH, people without HIV; SAE, serious adverse event.

**Supplementary Table S4.** **SAEs (full analysis set)**

| **ID^a^** | **HIV status** | **Regimen** | **Age**  **(years)** | **Sex** | **Preferred Term / Verbatim** | **Onset 1^b^/ Onset 2^c^/ Duration** | **Causality** | **CM Taken** | **Outcome of AE** |
| --- | --- | --- | --- | --- | --- | --- | --- | --- | --- |
| 1 | PWOH | MVA, Ad26 | 54 | F | Deep vein thrombosis / Deep vein thrombosis - left lower extremity | D 131 / D 117 / Unknown | Not related | Yes | Recovered |
| 2 | PWOH | Pbo, pbo | 34 | F | Anaphylactic reaction / Idiopathic anaphylactic reaction | D 188 / D 174 / 1 day | Not related | Yes | Recovered |
| 3 | PLWH | MVA, Ad26 | 52 | M | Panic attack / Intermittent panic attack | D 58 / D 44 / 308 days | Not related | Yes | Not recovered |
| 4 | PLWH | Pbo, pbo | 53 | M | Cervical cord compression / C5C6 acute cord compression | D 163 / D 149 / 4 days | Not related | No | Recovered |
| 4 | PLWH | Pbo, pbo | 53 | M | Staphylococcal osteomyelitis / Methicillin resistant staphylococcus aureus osteomyelitis | D 163 / D 149 / 217 days | Not related | Yes | Not recovered |

All SAEs were reported through study end.

Ad26, Ad26.ZEBOV at a dose of 5×10^10^ viral particles; AE, adverse event; CM, concomitant medication; D, day; F, female; M, male; MVA, MVA-BN-Filo at a dose of 1×10^8^ Inf.U; Pbo, placebo; PLWH, people living with HIV; PWOH, people without HIV; SAE, serious adverse event.

^a^ID: scrambled identifier, not tied to the actual participant ID in the clinical database. This scrambled ID allows determination of which participants reported multiple grade 3 SAEs.

^b^Onset 1: onset versus dose 1 vaccination.

^c^Onset 2: onset versus dose 2 vaccination.

**Supplementary Table S5. HIV viral load: categorization of HIV viral loads in PLWH**

|  | | **14-day Interval** | |
| --- | --- | --- | --- |
|  | | **MVA, Ad26** | **Placebo, Placebo** |
| Day 1 (baseline) | N | 20 | 5 |
|  | <20 copies/mL, n (%) | 16 (80) | 5 (100) |
|  | 20–200 copies/mL, n (%) | 4 (20) | 0 |
|  | >200 copies/mL, n (%) | 0 | 0 |
| Day 15 | N | 20 | 5 |
|  | <20 copies/mL, n (%) | 18 (90) | 3 (60) |
|  | 20–200 copies/mL, n (%) | 1 (5) | 2 (40) |
|  | >200 copies/mL, n (%) | 1 (5) | 0 |
| Day 36 | N | 20 | 5 |
|  | <20 copies/mL, n (%) | 18 (90) | 3 (60) |
|  | 20–200 copies/mL, n (%) | 1 (5) | 2 (40) |
|  | >200 copies/mL, n (%) | 1 (5) | 0 |
| Day 57 | N | 20 | 5 |
|  | <20 copies/mL, n (%) | 18 (90) | 4 (80) |
|  | 20–200 copies/mL, n (%) | 1 (5) | 1 (20) |
|  | >200 copies/mL, n (%) | 1 (5) | 0 |
| Day 195 | N | 19 | 5 |
|  | <20 copies/mL, n (%) | 19 (100) | 4 (80) |
|  | 20–200 copies/mL, n (%) | 0 | 1 (20) |
|  | >200 copies/mL, n (%) | 0 | 0 |
| Day 380 | N | 18 | 5 |
|  | <20 copies/mL, n (%) | 18 (100) | 5 (100) |
|  | 20–200 copies/mL, n (%) | 0 | 0 |
|  | >200 copies/mL, n (%) | 0 | 0 |
| Worst postbaseline | N | 20 | 5 |
|  | <20 copies/mL, n (%) | 16 (80) | 3 (60) |
|  | 20–200 copies/mL, n (%) | 3 (15) | 2 (40) |
|  | >200 copies/mL, n (%) | 1 (5) | 0 |

The denominator for the percentage calculation is the total number of participants with data at that time point.

Table is restricted to PLWH.

Ad26, Ad26.ZEBOV; MVA, MVA-BN-Filo; N, number of participants; PLWH, people living with HIV.

**Supplementary Table S6. EBOV GP-specific binding antibody responses in each study group, from baseline to study completion as measured by FANG anti-EBOV GP IgG ELISA**

|  | **PWOH** | | **PLWH** | |
| --- | --- | --- | --- | --- |
|  | **MVA, Ad26** | **Placebo, Placebo** | **MVA, Ad26** | **Placebo, Placebo** |
| Day 1 (baseline) |  |  |  |  |
| N | 38 | 10 | 19 | 5 |
| GMC (95% CI) | <LLOQ (<LLOQ–<LLOQ) | <LLOQ (<LLOQ–49) | <LLOQ (<LLOQ–<LLOQ) | <LLOQ |
| Day 15 |  |  |  |  |
| N | 38 | 10 | 19 | 5 |
| GMC (95% CI) | <LLOQ (<LLOQ–48) | <LLOQ (<LLOQ–45) | <LLOQ (<LLOQ–38) | <LLOQ |
| Responder, n/N* (%) | 4/38 (11) | 0/10 (0) | 2/19 (11) | 0/5 (0) |
| 95% CI | (3–25) | (0–31) | (1–33) | (0–52) |
| Day 36 |  |  |  |  |
| N | 36 | 9 | 19 | 5 |
| GMC (95% CI) | 6286 (4730–8355) | 39 (<LLOQ–229) | 2005 (923–4353) | <LLOQ |
| Responder, n/N* (%) | 36/36 (100) | 1/9 (11) | 18/19 (95) | 0/5 (0) |
| 95% CI | (90–100) | (0–48) | (74–100) | (0–52) |
| Day 57 |  |  |  |  |
| N | 36 | 10 | 19 | 5 |
| GMC (95% CI) | 4800 (3772–6110) | 36 (<LLOQ–178) | 1557 (802–3023) | <LLOQ |
| Responder, n/N* (%) | 36/36 (100) | 1/10 (10) | 19/19 (100) | 0/5 (0) |
| 95% CI | (90–100) | (0–44.5) | (82–100) | (0–52) |
| Day 195 |  |  |  |  |
| N | 36 | 10 | 18 | 5 |
| GMC (95% CI) | 1705 (1274–2282) | <LLOQ (<LLOQ; 75) | 655 (384–1117) | <LLOQ (<LLOQ; <LLOQ) |
| Responder, n/N* (%) | 36/36 (100) | 1/10 (10) | 17/18 (94) | 0/5 (0) |
| 95% CI | (90–100) | (0–44.5) | (73–100) | (0–52) |
| Day 380 |  |  |  |  |
| N | 37 | 10 | 17 | 5 |
| GMC (95% CI) | 1436 (1059–1946) | <LLOQ (<LLOQ; 65) | 531 (285–989) | <LLOQ |
| Responder, n/N* (%) | 37/37 (100) | 0/10 (0) | 16/17 (94) | 0/5 (0) |
| 95% CI | (90.5–100) | (0–31) | (71–100) | (0–52) |

N: number of participants with data at that time point; N*: number of participants with data at baseline and at that time point.

GMCs and corresponding CIs are shown as EU/mL.

For the responder rates, Exact Clopper-Pearson CI are shown.

Ad26, Ad26.ZEBOV; CI, confidence interval; EBOV, Ebola virus; ELISA, enzyme-linked immunosorbent assay; EU, ELISA units; FANG, Filovirus Animal Non-Clinical Group; GMC, geometric mean concentration; GP glycoprotein; IgG, immunoglobulin G; LLOQ, lower limit of quantification; MVA, MVA-BN-Filo; PLWH, people living with HIV; PWOH, people without HIV.

**Supplementary Figure S1. Correlation between HIV viral load at baseline (panel A) and CD4+ T-cells at baseline (panel B) and EBOV GP binding antibody in active vaccine recipients at Day 36**

EBOV, Ebola virus; ELISA, enzyme-linked immunosorbent assay; GP, glycoprotein; LLOQ, lower limit of quantification.

**Supplementary Figure S2. Correlation between EBOV GP binding antibody (Kikwit) and neutralizing antibody responses at Day 36 in PWOH and PLWH (active vaccine recipients)**

Correlation analysis comparing binding antibody responses as measured by FANG anti-EBOV GP IgG ELISA (y-axis) with psVNA IC_50_ titers for EBOV Zaire 95 Kikwit virus strain. Lower assay limits are shown as dotted lines.

EBOV, Ebola virus; ELISA, enzyme-linked immunosorbent assay; FANG, Filovirus Animal Non-Clinical Group; GP, glycoprotein; IC_50_, 50% inhibitory concentration; IgG, immunoglobulin G; LLOQ, lower limit of quantification; PLWH, people living with HIV; psVNA, pseudovirion neutralization assay; PWOH, people without HIV.

**Supplementary Figure S3. Cross-strain EBOV GP-specific neutralizing antibody responses as measured by psVNA**

Specimens from PWOH (A–C) or PLWH (D–F) who were vaccinated with MVA, Ad26 or a placebo were evaluated by psVNA to measure the cross-neutralizing antibody response against A,D) EBOV Zaire 95 Kikwit strain, B,E) EBOV Zaire 14 Makona strain or C,F) Bundibugyo virus. Blood was collected on Days 0, 15, 36, 57, 195, and 360 and run in the assays. Geometric mean IC_50_ titers (GMTs) ± SD were plotted. Numbers of participants, % responders, and GMT are reported. The lower assay limit was 20 (dotted line). Participants with titers <20 were given a value of 14.1 for analysis.

Ad26, Ad26.ZEBOV; EBOV, Ebola virus; IC_50_, 50% inhibitory concentration; GP, glycoprotein; IC_50_, 50% inhibitory concentration; MVA, MVA-BN-Filo; N, number of participants; PLWH, people living with HIV; psVNA, pseudovirion neutralization assay; PWOH, people without HIV; SD, standard deviation.

**Supplementary Figure S4. Cross-strain EBOV GP neutralizing antibody responses as measured by psVNA**

Specimens from PLWH or PWOH who were vaccinated with MVA, Ad26 or a placebo were evaluated by psVNA to measure the cross-neutralizing antibody response against A) EBOV Zaire 14 Makona virus strain or B) Bundibugyo virus strain. Blood was collected on Days 0, 15, 36, 57, 195, and 360 and run in the assays. Geometric mean IC_50_ titers ± SD were plotted. The lower assay limit was 20 (dotted line).

Ad26, Ad26.ZEBOV; EBOV, Ebola virus; GP, glycoprotein; IC_50_, 50% inhibitory concentration; MVA, MVA-BN-Filo; PLWH, people living with HIV; psVNA, pseudovirion neutralization assay; PWOH, people without HIV; SD, standard deviation.

**Supplementary Figure S5. Correlation between cross-strain neutralizing antibody responses and EBOV GP-specific binding antibody responses at Day 36 in active vaccine recipients**

Correlation analysis comparing binding antibody responses as measured by FANG anti-EBOV GP IgG ELISA (y-axis) with psVNA IC_50_ titers for (A) EBOV Z14 Makona virus strain and (B) Bundibugyo (BUN) virus strain. Lower assay limits are shown as dotted lines.

EBOV, Ebola virus; ELISA, enzyme-linked immunosorbent assay; FANG, Filovirus Animal Non-Clinical Group; GP, glycoprotein; IC_50_, 50% inhibitory concentration; IgG, immunoglobulin G; LLOQ, lower limit of quantification; psVNA, pseudovirion neutralization assay.

**Supplementary Figure S6. EBOV GP-specific ADCP responses**

ADCP was evaluated at Days 0, 36, and 360. (A) PWOH vaccinated with MVA, Ad26, (B) PLWH participants vaccinated with MVA, Ad26, (C) PWOH who received placebo, placebo, (D) PLWH participants who received placebo, placebo. Data are plotted as individual geometric mean ADCP scores ± 95% CI were plotted. The threshold for positivity was determined using plasma from controls (dotted line).
Ad26, Ad26.ZEBOV; ADCP, antibody-dependent cellular phagocytosis; CI, confidence interval; EBOV, Ebola virus; Geo, geometric; GP, glycoprotein; MVA, MVA-BN-Filo; N, number of participants; PLWH, people living with HIV; PWOH, people without HIV.

**Supplementary Figure S7. EBOV GP-specific antibody-dependent natural killer cell activation (ADNKA) responses**

ADNKA was evaluated by ICS at Days 0, 36, and 360. (A) PWOH vaccinated with MVA, Ad26, (B) PLWH participants vaccinated with MVA, Ad26, (C) PWOH participants who received placebo, (D) PLWH participants who received placebo. Data are plotted as individual geometric means ± 95% CI. The threshold for positivity was determined using plasma from controls (dotted line).
Ad26, Ad26.ZEBOV; ADNKA, antibody-dependent natural killer cell activation; CI, confidence interval; EBOV, Ebola virus; Geo, geometric; GP, glycoprotein; IFNγ, interferon γ; MIP1β, macrophage inflammatory protein 1 β; MVA, MVA-BN-Filo; ICS, intracellular cytokine staining; N, number of participants; NK, natural killer; PLWH, people living with HIV; PWOH, people without HIV; TNF, tumor necrosis factor.

**Supplementary Figure S8. EBOV GP-specific ADCD responses**

ADCD was evaluated at Days 0, 36, and 360. (A) PWOH vaccinated with MVA, Ad26, (B) PLWH participants vaccinated with MVA, Ad26, (C) PWOH who received placebo, (D) PLWH participants who received placebo. Data are plotted as individual geometric mean ADCD scores ± 95% CI. The threshold for positivity was determined using plasma from controls (dotted line).

Ad26, Ad26.ZEBOV; ADCD, antibody-dependent complement deposition; CI, confidence interval; EBOV, Ebola virus; Geo, geometric; GP, glycoprotein; MVA, MVA-BN-Filo; N, number of participants; PLWH, people living with HIV; PWOH, people without HIV.

**Supplementary Figure S9. Antibody effector polyfunctionality**

Percent of participants per group displaying positive (psVNA, ADCD, ADCP, ADNKA) antibody functions at Day 36 and Day 360. ADCD, antibody-dependent complement deposition; ADCP, antibody-dependent cellular phagocytosis; ADNKA, antibody-dependent natural killer cell activation; PLWH, people living with HIV; psVNA, pseudovirion neutralization assay; PWOH, people without HIV.

**Supplementary Figure S10. Combinatorial polyfunctionality analysis of antigen specific T-cell subsets (COMPASS)**

Heatmap of COMPASS posterior probabilities for (A) EBOV-specific CD4+ T-cells and (B) EBOV-specific CD8+ T-cells. In each case, columns in the lower part of the figure correspond to the different cell subsets modeled by COMPASS, color-coded by the cytokines (IL-21, IL-2, TNF-α, IL-4, IFN-γ, CD154) they express (white = “off,” shaded = “on,” color = “degree of functionality”), and ordered by degree of functionality from one function on the left to six functions on the right. In the upper part of each figure, rows correspond to individual participants. Each cell of the heatmap shows the probability that a given cell subset (columns from lower part of figure) has an antigen-specific response in the corresponding participant (columns in upper part of figure), where the probability is color-coded from white (zero) to purple (one).

EBOV, Ebola virus; IL, interleukin; INF-γ, interferon γ; PLWH, people living with HIV; PWOH, people without HIV; TNF-α, tumor necrosis factor α.

**Members of the VAC52150/EBL2003/RV456 study team (in addition to authors)**

**MHRP:** Aljawharah Alrubayyi, Siriwat Akapirat, Michael Barrera, Jacob Boeckelman, Shayla Duncan, Gina Griffith, Dohoon Kim, Kayvon Modjarrad, Amber Moodley, Vicky Polonis, Christina Polyak, Morgane Rolland, Pallavi Thapa, Rasmi Thomas, Lindsay Wieczorek

**WRAIR-CTC:** Anthony Cardile, Elizabeth P. Harausz, Michael Koren, Elena Kwon, Leyi Lin, Jeffrey Livezey, Kristin Mills, James Moon, Anne Preston, Shon Remich, Jason Regules, Donna Tosh, Patrick Twomey

**U.S. DOD JPEO-CBRND, JPM CBRN Medical:** Rachel Overman, Andrew Glenn

**Contract support for U.S. DOD JPEO-CBRND, JPM CBRN Medical:** Ronald Reisler, Aaron Short

**Janssen:** Leen Kwanten, Melissa van Alst, Griet van Roey, Daniel Splinter

**Battelle Memorial Institute:** Jeremy Smith
